# Leveraging Ligand Affinity and Properties: Discovery of Novel Benzamide-Type Cereblon Binders for the Design of PROTACs

**DOI:** 10.1021/acs.jmedchem.3c00851

**Published:** 2023-10-30

**Authors:** Christian Steinebach, Aleša Bricelj, Arunima Murgai, Izidor Sosič, Luca Bischof, Yuen Lam Dora Ng, Christopher Heim, Samuel Maiwald, Matic Proj, Rabea Voget, Felix Feller, Janez Košmrlj, Valeriia Sapozhnikova, Annika Schmidt, Maximilian Rudolf Zuleeg, Patricia Lemnitzer, Philipp Mertins, Finn K. Hansen, Michael Gütschow, Jan Krönke, Marcus D. Hartmann

**Affiliations:** † Pharmaceutical Institute, University of Bonn, D-53121 Bonn, Germany; ‡ Faculty of Pharmacy, University of Ljubljana, SI-1000 Ljubljana, Slovenia; § Department of Hematology, Oncology, and Cancer Immunology, Charité - Universitätsmedizin Berlin, corporate member of Freie Universität Berlin, Humboldt-Universität zu Berlin, D-12203 Berlin, Germany; ∥ Max Planck Institute for Biology Tübingen, D-72076 Tübingen, Germany; ⊥ Faculty of Chemistry and Chemical Technology, University of Ljubljana, SI 1000 Ljubljana, Slovenia; # Max Delbrück Center for Molecular Medicine, D-13125 Berlin, Germany; ∇ German Cancer Consortium (DKTK), Partner Site Berlin, DKFZ, D-69120 Heidelberg, Germany; ¶ Berlin Institute of Health, D-10178 Berlin, Germany; ● Interfaculty Institute of Biochemistry, University of Tübingen, D-72076 Tübingen, Germany

## Abstract

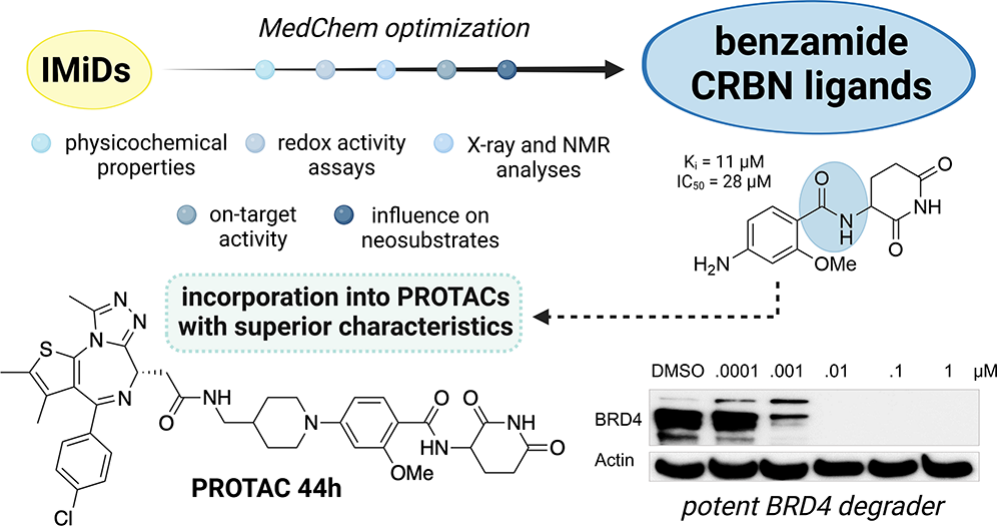

Immunomodulatory imide drugs (IMiDs) such as thalidomide, pomalidomide, and lenalidomide are the most common cereblon (CRBN) recruiters in proteolysis-targeting chimera (PROTAC) design. However, these CRBN ligands induce the degradation of IMiD neosubstrates and are inherently unstable, degrading hydrolytically under moderate conditions. In this work, we simultaneously optimized physiochemical properties, stability, on-target affinity, and off-target neosubstrate modulation features to develop novel nonphthalimide CRBN binders. These efforts led to the discovery of conformationally locked benzamide-type derivatives that replicate the interactions of the natural CRBN degron, exhibit enhanced chemical stability, and display a favorable selectivity profile in terms of neosubstrate recruitment. The utility of the most potent ligands was demonstrated by their transformation into potent degraders of BRD4 and HDAC6 that outperform previously described reference PROTACs. Together with their significantly decreased neomorphic ligase activity on IKZF1/3 and SALL4, these ligands provide opportunities for the design of highly selective and potent chemically inert proximity-inducing compounds.

## Introduction

**Figure 1:**
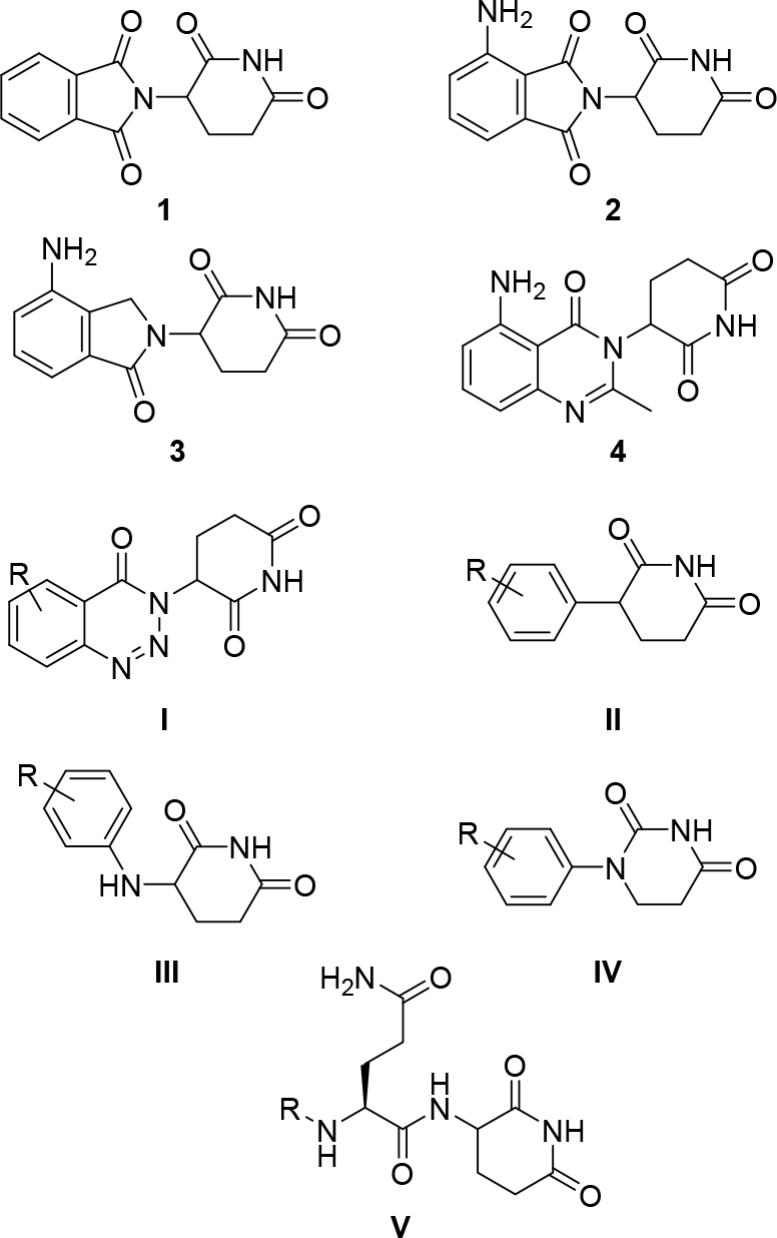
CRBN ligand landscape. Established CRBN modulators (**1**–**4**), novel scaffolds (**I**–**IV**) used in degrader design, and the terminal residues (QcQ) of a recently discovered natural degron (**V**).

Deciphering thalidomide’s mechanism of action in 2010 by Ito et al. ignited a transformative process in drug discovery toward the development of proximity-induced pharmacology as a new therapeutic modality.^1,2^ The potential and benefits of targeted protein degradation (TPD) as a cutting-edge paradigm are thoroughly demonstrated by molecular glues (MGs) and proteolysis-targeting chimeras (PROTACs).^3,4^ Nowadays, the E3 ligase substrate receptor cereblon (CRBN), thalidomide’s primary target,^1,5^ is of particular importance in the fields of MGs and PROTACs.^6,7^ The immunomodulatory imide drugs (IMiDs) thalidomide (**1**, Figure 1), pomalidomide (**2**), lenalidomide (**3**), and avadomide (**4**) represent classical CRBN recruiters that are frequently exploited for PROTAC design.^2,6,8^ One feature, but also a potential caveat of this class of ligase binders, is their potential to promote the degradation of lymphoid transcription factors such as IKZF1, IKZF3, and SALL4, the last being responsible for severe teratogenicity caused by thalidomide analogs.^9−13^ Furthermore, the typical IMiD scaffolds are prone to hydrolytic and enzymatic degradation, rendering them less attractive for pharmacological application.^14−16^

These observations led to the development of a series of new CRBN binders that could potentially overcome the limitations above. For instance, benzotriazino glutarimides (scaffold **I**, Figure 1) have been successfully employed to generate BRD4-targeting PROTACs. Rankovic and colleagues recently reported on phenyl glutarimides (**II**), which retained CRBN affinity and significantly improved chemical stability over phthalimide-based recruiters.^16^ Phenyl glutarimides were incorporated in degraders targeting BRD4 and cancer-related kinases.^16,17^ Anilino glutarimides (**III**) and related aryl glutarimides were utilized to generate USP7-targeting PROTACs and PDE6D-directed MGs.^18,19^ We noted significant improvements in the solubility of anilinic ligands compared to classical scaffolds, which could pave the way for *in vivo* applications of such molecules. Recently, three groups reported on phenyl dihydrouracil derivatives (**IV**) as CRBN-engaging agents.^20−22^ These ligands combined several desirable features, including improved resistance to hydrolytic degradation. Furthermore, replacing the C-3 carbon of the glutarimide ring with nitrogen addressed commonly challenged racemization issues of IMiD-based degraders.

Our laboratories are interested in advancing E3 ligase ligands and have contributed in various ways to explore their structure–activity relationships within PROTAC design.^14,18,23−25^ CRBN ligands rely on only a small set of interactions that are classically limited to H-bonds of the glutarimide moiety within the conserved tri-tryptophan pocket and a further H-bond between one of the phthalimide carbonyls and a conserved asparagine (Asn351) in the sensor loop.^26−28^ We and others have suggested that simplifying classical phthalimide-based ligands can further improve the suitability of CRBN-hijacking probes. For instance, in 2016, we described a novel FRET reporter for the characterization of CRBN ligands based on a minimal uracil scaffold.^29^ Later, we utilized this recruiter to develop a BODIPY derivative suitable for microscale thermophoresis (MST) assays.^30^ Similar to the newly reported phenyl glutarimide- and phenyl dihydrouracil-based ligands, these reporters rely mainly on interactions within the tri-tryptophan pocket, but do not interact with Asn351 as phthalimide-based ligands do. However, the interplay with Asn351 can be restored by attaching an amide group to the core binding moieties of aminosuccinimide or aminoglutarimides, as exemplified by thalidomide hydrolysis products that maintained CRBN affinity.^23,24^ Intriguingly, we and others recently reported this particular minimal binding motif to be identical to a natural degron that interacts with CRBN (**V**, Figure 1).^31−33^ Based on these studies on minimal CRBN ligands and the medicinal chemistry-driven optimization of a benzamide-type screening hit, we have obtained attractive nonphthalimide CRBN binders for PROTAC design. Our design strategies build on the analysis of intramolecular bonds, cocrystal structures, physicochemical characteristics, stability data, and on-target activity.

## Results and Discussion

### Fluorinated Benzamide Derivatives Exhibit Increased CRBN Binding Affinity

**Scheme 1:**
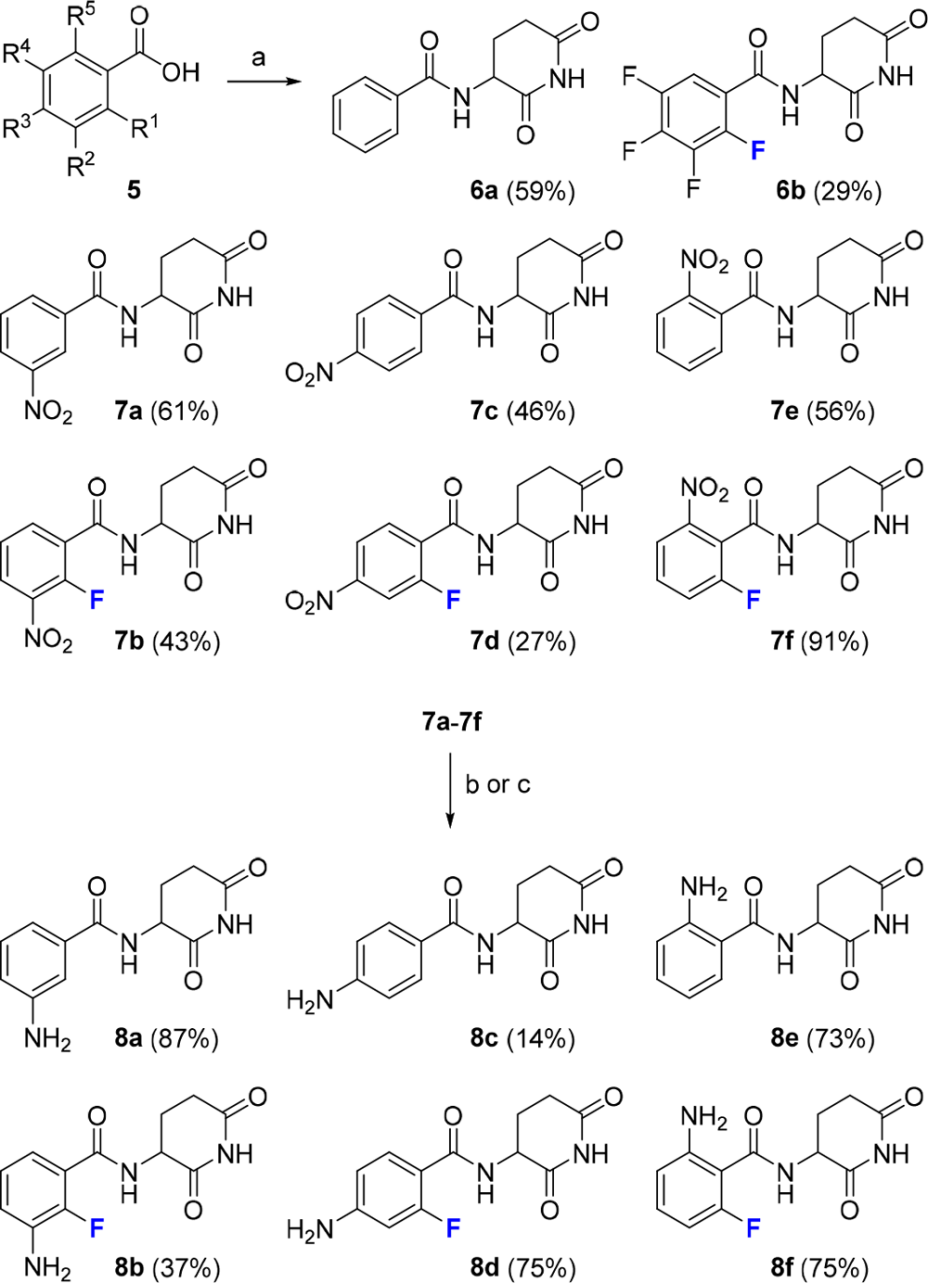
Synthesis of Substituted Benzamido Glutarimides Reagents and conditions: (a) (i) (COCl)_2_, DMF (cat.), CH_2_Cl_2_, 0 °C, 2 h; (ii) ArCOCl, Et_3_N, 3-aminopiperidine-2,6-dione hydrochloride, CH_2_Cl_2_, 0 °C, to rt, 18 h; (b) Pd/C, H_2_, DMF, rt, 18 h; (c) Fe, EtOH, AcOH, H_2_O, 110 °C, 30 min.

In previous studies, we discovered that the introduction of fluorine atoms improved the biological properties of thalidomide derivatives.^34−36^ Specifically, the perfluorination of benzamides increased the binding affinity compared to its nonfluorinated analog (**6b** vs **6a**, Scheme 1).^34^ Fluoro-substituted compounds enjoy particular success in medicinal chemistry, and a high proportion of drugs in the pharmaceutical pipeline contain at least one fluorine atom.^37^ The incorporation of fluorine can affect several essential properties in drug design. For instance, lipophilicity, metabolic stability, membrane permeation, and binding affinity can be modulated by incorporating an electronegative fluorine atom.^38^ However, fluorine is also considered a conformation-controlling element through hyperconjugative electron donation of vicinal groups and, potentially, also through intramolecular hydrogen bonds (IMHBs) of the C–F···H–N type.^39,40^ Interestingly, fluorine-containing HBs were reported for ortho-fluorobenzamides.^41^ Accordingly, we proposed an amphiphilic character of the fluorine in **6b**, i.e., as a hydrogen-bond acceptor and a hydrophobic moiety. To further clarify the effect of the substituent at the ortho position, we synthesized a series of six related compounds, having either a hydrogen or fluorine atom attached to the aromatic moiety (**8a**–**8f**, Scheme 1). In these molecules, an amino group was installed at the different unsubstituted positions to scan potential fragment growing and/or linker exit vectors.

Next, we used our previously established MST assay to measure binding to the human CRBN thalidomide binding domain (hTBD).^30^ In three out of four cases, the fluorinated derivative displayed lower IC_50_ values than the nonhalogenated counterpart (Table 1). Additional IMHBs due to the second ortho substituent could explain the deviation from this trend in **8f**. The fluorine-containing compound **8d** exhibited the highest affinity in this series with an IC_50_ value of 63 ± 16 μM. In addition to the *in vitro* binding values, data on critical physiochemical properties were also collected. As expected, the introduction of fluorine increased the lipophilicity (log *D*) of the compounds. In contrast, plasma protein binding was less affected by these minor chemical modifications. Substituted derivatives had higher chromatographic hydrophobicity index (CHI) values, indicating less pronounced permeability obstacles.

**Table 1:**
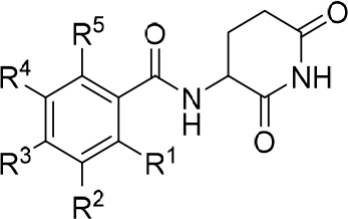
Chemical Structures, Binding Data, Distribution Coefficients, Plasma Protein Binding Properties, and Phospholipid Interaction Capabilities of Benzamides 6 and 8

Ligand	R^1^	R^2^	R^3^	R^4^	R^5^	IC_50_ (μM)^a^	*K*_i_ (μM)^a^	elog *D*_7.4_^b^	PPB (%)^c^	CHI_IAM_^d^
**2**						13 ± 2.7	3.3 ± 1.4	0.5	51	–4.0
**3**						19 ± 1.5	6.4 ± 0.8	–0.4	12	–2.6
**6a**	H	H	H	H	H	127 ± 40	63 ± 21	–0.3	10	6.3
**6b**	F	F	F	F	H	65 ± 26	30 ± 14	0.9	17	16.7
**8a**	H	NH_2_	H	H	H	107 ± 45	53 ± 24	–1.2	4	–1.1
**8b**	F	NH_2_	H	H	H	93 ± 19	45 ± 9.9	–1.2	4	0.7
**8c**	H	H	NH_2_	H	H	86 ± 21	41 ± 11	–2.0	3	–1.1
**8d**	F	H	NH_2_	H	H	63 ± 16	29 ± 8.2	–0.7	5	5.2
**8e**	H	H	H	H	NH_2_	74 ± 14	35 ± 7.0	–0.5	9	6.2
**8f**	F	H	H	H	NH_2_	114 ± 60	56 ± 31	–0.1	12	8.1

a Affinity values determined in a competitive MST assay as described in the methods sections. For comparison, pomalidomide (**2**) and lenalidomide (**3**) were included, for which data is from ref (30).

b Distribution coefficients at pH 7.4 were estimated by an HPLC-based method.

c Plasma protein binding; experimentally determined percentage of compound bound to human serum albumin.

d Chromatographic hydrophobicity index values referring to IAM chromatography (CHI_IAM_ values), an estimate for drug–membrane interactions and permeability.

### Intramolecular Hydrogen Bonds Predetermine Ligand Conformations

Inspired by the positive results of placing a fluorine atom at the ortho position of the benzamide scaffold, we sought to investigate the structure–activity relationships in more detail. Accordingly, analogous compounds were synthesized, allowing for IMHBs that pertain to motifs of the type C–O···H–N or C-X···H–N (with X = halogen). When appropriately substituting molecule **8c** from R^1^ = H to R^1^ = X, the aromatic ring becomes more electron deficient, the amide NH group more acidic, and the entire molecule displays increased lipophilicity. Such features could favorably affect the biological activity of the CRBN ligands. To pinpoint whether the ortho substituents may improve binding affinity *via* IMHBs or electronic effects, we designed a series of 7 related compounds. Halo derivatives **8d** (R = F) and **11a** (R = Cl) could benefit from both mechanisms, haloalkyl compound **11b** (R = CF_3_) displays strong deactivating properties, whereas compounds **11c** (R = CH_3_), **11d** (R = OMe), and **11e** (R = OH) have ascending electron donating features. In addition, **11d** and **11e** could contribute to IMHBs. In **11f**, the ortho-fluorine may constitute the major design element, but additional decorations at the benzamide scaffold could prove beneficial. Due to the rather tight spatial restraints of the thalidomide binding site,^28^ more spacious polar substituents (e.g., nitro, amide, ester) were not considered in our compound design.

**Scheme 2:**
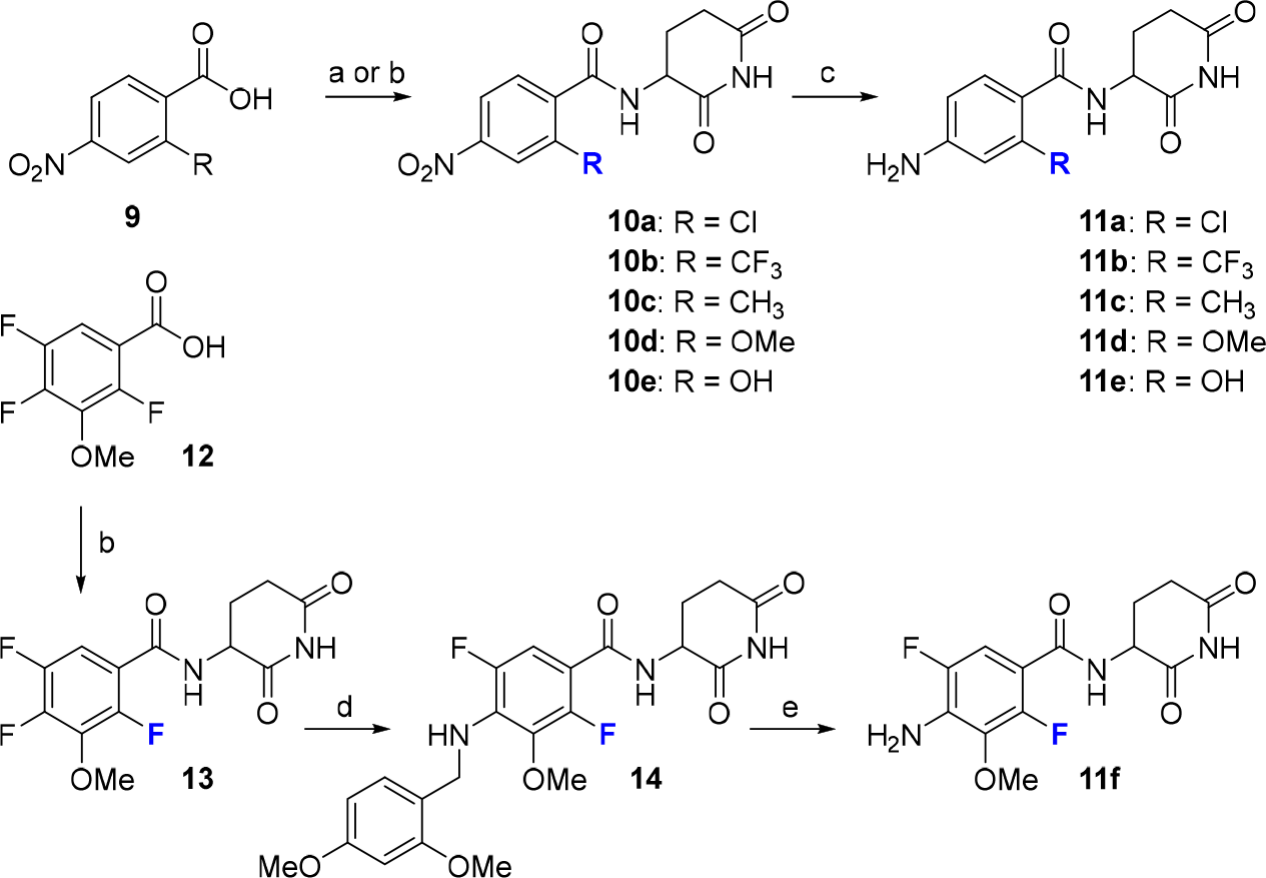
Synthesis of Different Ortho-Substituted Para-Aminobenzoates Reagents and conditions: (a) (i) oxalyl dichloride, DMF (cat.), CH_2_Cl_2_, 0 °C to rt, 2 h; (ii) 3-aminopiperidine-2,6-dione hydrochloride, Et_3_N, CH_2_Cl_2_, rt, 16 h, 80–83%; (b) EDC × HCl, HOBt, 3-aminopiperidine-2,6-dione hydrochloride, DIPEA, DMF, rt, 16 h, 35–74%; (c) Pd/C, H_2_, DMF, rt, 18 h, 11–74%; (d) (2,4-dimethoxyphenyl)methanamine, DIPEA, DMSO, 90 °C, 16 h, 35%; (e) TFA, CH_2_Cl_2_, rt, 2 h, 77%.

The syntheses of compounds **11** are outlined in Scheme 2 and proceeded in most cases *via* the established route of EDC/HOBt-mediated couplings between benzoic acid derivatives of type **9** and the aminoglutarimide building block.^42^ Subsequent reduction of the nitro group afforded the desired anilines **11a**–**11e.** Derivative **11f** was obtained from **12** after amide bond formation, S_N_Ar at position 4 (Figure S1) with 2,4-dimethoxybenzylamine, and cleavage of the dimethoxybenzyl C–N bond with TFA in CH_2_Cl_2_.^43^

**Figure 2:**
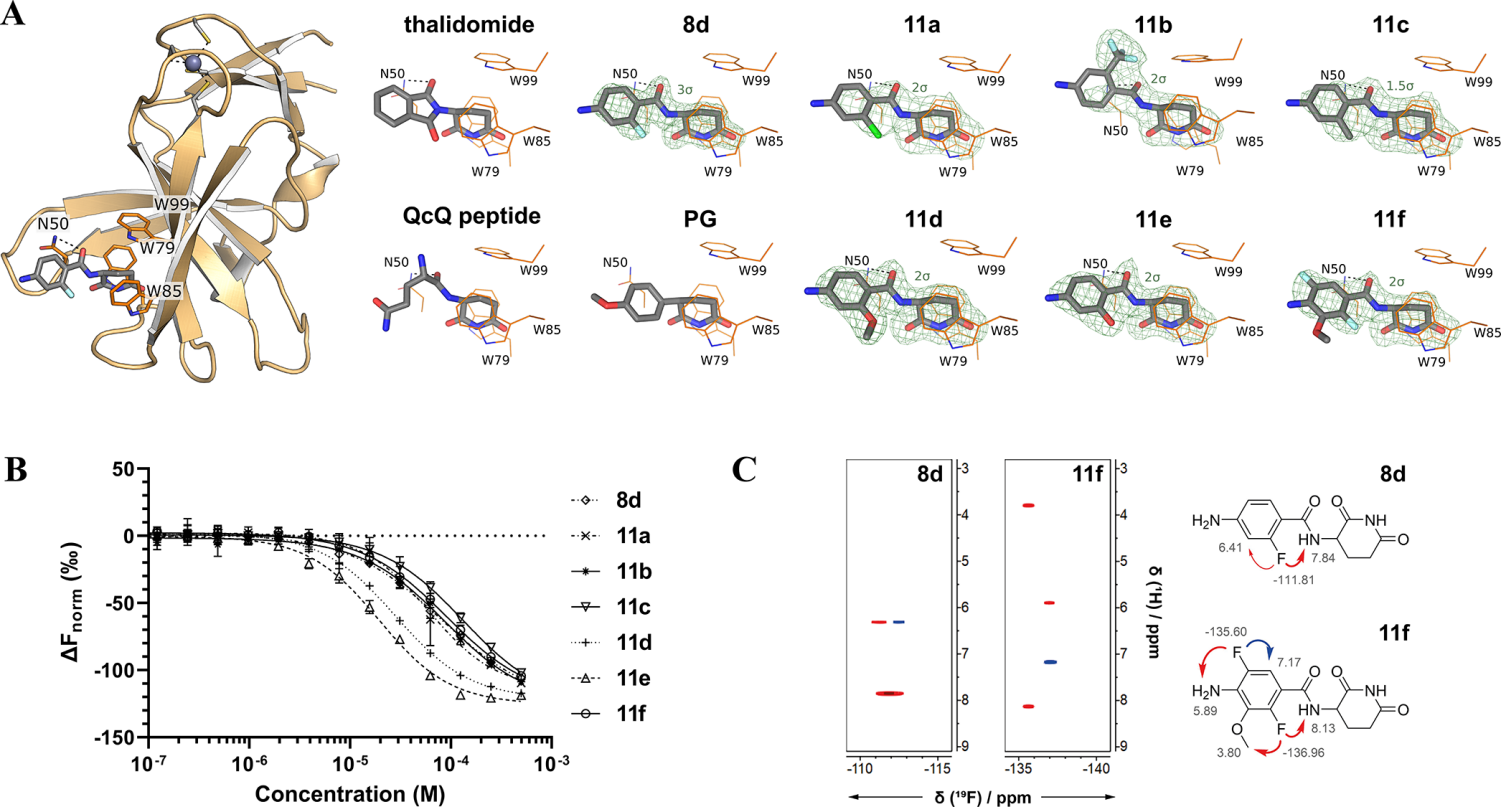
(A) Difference electron density (*F*_o_ – *F*_c_) maps, contoured at the indicated sigma level, of ortho-substituted benzamide compounds bound to crystallized MSCI4. The full-size crystal structure is exemplary shown for **8d** on the left. For comparison, thalidomide, a QcQ peptide (the two last amino acids of a natural degron peptide), and a phenyl glutarimide (PG) are shown (PDB identifiers: 4V2Y, 8BC7, and 7SHH, respectively). Nitrogen is shown in blue, carbon in gray, oxygen in red, fluorine in light blue, and chlorine in chartreuse. (B) Dose–response curves for compounds **8d** and **11a**–**11f** were obtained in competitive MST measurements with BODIPY-uracil and hTBD (*n* = 3). (C) Sections of ^19^F,^1^H-HOESY NMR spectra of **8d** and **11f**, respectively. Spatial proximity between F and the amide NH provides evidence for intramolecular interactions. NOE with residual water is not displayed.

The inherent conformational flexibility of benzamides **11** along the amide bond was investigated using a force field-based method implemented in Schrödinger MacroModel. Global minimum conformations are depicted in Figure S2. As expected, IMHBs were observed in low-energy conformers, except for **11a**–**11c**. Fortunately, we could study all compounds generated in this iterative step through cocrystal structures (Figure 2) in complex with the hTBD homolog MsCI4.^44^ These data confirmed that the preferred orientation of the ortho substituent toward the amide NH was preserved in the bound state of all ligands except for **11b**. However, the intrinsic preference of these atoms for the formation of H-bonds through van der Waals forces may facilitate mimicking the bonded pose as in isoindoles of types **2** and **3**. Sub van der Waals distances ranging from 1.3 to 2.0 Å were seen in **8d**, **11a**, **11c**, **11d**, **11e**, and **11f** (Table S1). Overall, compounds with C–O···H–N or C-X···H–N patterns achieved a higher affinity to the hTBD (Table 2). To investigate whether such intramolecular H-bonding may even persist in the polar environment of a solvated compound, we performed 2D NMR experiments in DMSO. We determined that F and the NH proton are closely spaced by observing correlation peaks between the NH proton and the F signals in the ^19^F,^1^H-HOESY NMR spectra of **8d** and **11f** (Figure 2C). The results, therefore, strongly suggest that IMHBs predetermine the ligand conformation in solution. Overall, the decreased conformational freedom may be attractive in terms of increased bioavailability and reduced entropic penalty upon binding with CRBN.^45^

**Table 2:**
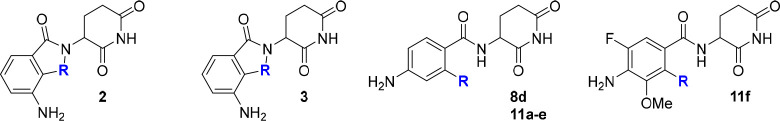
Chemical Structures, Binding Data, Physicochemical Properties, and Cellular Activities of Ortho-Substituted Benzamides

										neosubstrate deg (%)^h^,^i^	
Ligand	R	IC_50_ (μM)^a^	*K*_i_ (μM)^a^	elog *D*_7.4_^b^	PPB (%)^c^	CHI_IAM_^d^	log(*S*)^e^	Redox activity^f^	UV/vis stability^g^	IKZF3	SALL4
**2**	C=O	13 ± 2.7	3.3 ± 1.4	0.5	51	–4.0	–4.0	n.a.^j^	<pH 9	86	95
**3**	CH_2_	19 ± 1.5	6.4 ± 0.8	–0.4	12	–2.6	–2.6	not active	stable	57	70
**8d**	F	63 ± 16	29 ± 8.2	–0.7	5	5.2	–3.1	not active	stable	39	14
**11a**	Cl	60 ± 13	28 ± 6.6	–0.9	10	2.6	–2.6	not active	stable	13	<5
**11b**	CF_3_	87 ± 25	42 ± 13	–0.3	6	6.7	–3.5	not active	stable	69	45
**11c**	CH_3_	132 ± 55	65 ± 29	–1.2	2	–1.1	–2.5	not active	stable	57	<5
**11d**	OMe	28 ± 2.6	11 ± 1.4	0.0	17	6.5	–2.9	not active	stable	14	14
**11e**	OH	20 ± 2.0	6.8 ± 1.0	–0.3	18	8.8	–3.0	active	<pH 9	41	<5
**11f**	F	90 ± 17	44 ± 9.0	–0.9	14	2.6	–3.0	not active	stable	63	42

a Affinity values determined in a competitive MST assay as described in the method sections (see Supporting Information).

b Distribution coefficients at pH 7.4 were estimated by an HPLC-based method.

c Plasma protein binding; experimentally determined percentage of compound bound to human serum albumin.

d Chromatographic hydrophobicity index values referring to IAM chromatography (CHI_IAM_ values), an estimate for drug–membrane interactions and permeability.

e Logarithm of the solubility measured in mol/L at pH 6.8 by an HPLC-based method.

f Redox activity assays for the detection of compounds that react with reducing agents in redox cycles by forming ROS (H_2_DCFDA assay) or free radicals (resazurin assay); see ref (49).

g UV–vis-based assay for the evaluation of aqueous stability in phosphate buffer at pH 7.0, 8.0, and 9.0 after 4 h of incubation at 37 °C.

h Percentage of degraded IKZF3 protein after 16 h treatment of MM.1S cells with 0.1 μM of each compound.

i Percentage of degraded SALL4 protein after 16 h treatment of HuH6 cells with 0.1 μM of each compound. Western blots were analyzed by densitometric methods, and values were normalized to the respective loading controls and to DMSO-treated conditions. IKZF3/SALL4 degradation data represent the average of at least two independent biological experiments.

j Not available due to spectral interference.

Next, ligands **11** were evaluated concerning their physicochemical properties, stability, intrinsic reactivity, and neosubstrate modulation features. For the former, relevant properties such as lipophilicity (determined by an HPLC method), plasma protein binding, permeability (using immobilized artificial membrane chromatography), and solubility (log *S*) were determined (Table 2). As expected, introducing hydrophobic substituents at the ortho position increased the lipophilicity compared to the unsubstituted derivative **8c** (log *D* = −2.0), e.g., by up to two log units in compound **11d**. As commonly accepted, increasing lipophilicity by adding halogen atoms is detrimental to aqueous solubility.^46^ Although a negative trend was observed (Figure S3A), this correlation was less pronounced in our series of compounds. In contrast, more lipophilic compounds increased the CHI value, which may indicate improved passive permeability (Figure S3B). When plotting the percentage of human serum albumin (HSA) binding or the IC_50_ versus log *D*_7.4_, the correlation of the linear regressions improved when only conformationally locked compounds were considered (Figure S3C,D). The latter observation is consistent with the enhanced CRBN binding affinities of hydrophobically decorated ligands.^5,47,48^

To detect a potential oxidative liability of the aniline moiety, we employed our previously optimized redox activity assays^49^ using the reagents resazurin and 2′,7′-dichloro-dihydrofluorescein diacetate (H_2_DCFDA), which are capable of detecting compounds that react with reducing agents in redox cycles (Table 2). Hit compound **11e** had to be excluded from further development due to the formation of reactive oxygen species (ROS) in the presence of the reducing agent tris(2-carboxyethyl)phosphine (TCEP). Subsequently, the aqueous stability was determined spectrophotometrically by following the changes in the absorption spectra of the compounds at pH 7.0, 8.0, and 9.0. These data confirmed the known hydrolytic susceptibility of compound **2**,^14,24^ and revealed poor stability of **11e** at higher pH values.

Benzamides **11** were assessed in MM.1S cells for their abilities to induce degradation of the CRBN neosubstrate IKZF3 (Figure S4A).^10,50^ Indeed, the substituted benzamides were less active recruiters of this transcription factor than pomalidomide (**2**) and lenalidomide (**3**). Nevertheless, neosubstrate degradation varied, although compounds of type **11** share the same anilinic degron motif and differ only in the ortho substituent. We hypothesized that the substituent is responsible for slight conformational changes (see Figure 2A), which could affect the recruitment efficiency. Among these compounds, **8d**, **11a**, and **11d** represent exceptionally selective CRBN binders for PROTAC development. In contrast, compound **11b** (R = CF_3_) led to the most pronounced degradation of IKZF3 (69%) at 0.1 μM (Table 2). As IMiDs such as **1** can induce the degradation of the spalt-like transcription factor 4 (SALL4), an oncofetal protein involved in limb development during embryogenesis, particular attention should be paid to potential teratogenic effects.^12,13,51^ We determined SALL4 degradation mediated by classical IMiDs and benzamides **11** in HuH6 hepatoblastoma cells (Figure S4B). Encouragingly, even compounds with CRBN binding affinity similar to lenalidomide, such as **11d** and **11e**, did not trigger substantial degradation of SALL4 (Table 2). In addition, our investigations did not reveal any discernible impact of benzamides on the activity of lenalidomide-selective neosubstrate CK1α or GSPT1, both of which are known to be targeted for degradation by IMiD-based PROTACs (Figure S4A). These proteins play crucial roles in several cell types and are implicated in toxicity-related processes. The entire set of available data (i.e., *in vitro* binding, physicochemical properties, and cellular neosubstrate attenuation) guided further development of bifunctional molecules based on the benzamide scaffold **11**.

### Evaluating the Effectiveness of Benzamides in the Design of MGs and PROTACs

In the next step, we sought to challenge linker-connected CRBN ligands for neosubstrate degradation. The attachment of a small linker could serve as a minimal degron to modulate substrate recognition of CRBN and could predict the scope of neosubstrate recruitment after the binding of benzamide-based PROTACs. In addition to the transcription factors IKZF1, IKZF3, and SALL4, we considered the degradation of GSPT1 as a problematic off-target, since their depletion causes profound effects in a variety of healthy tissues.^52−54^ Accordingly, evaluating CRBN ligands for their impact on attenuating these proteins is of great interest for MG and PROTAC development.

**Scheme 3:**
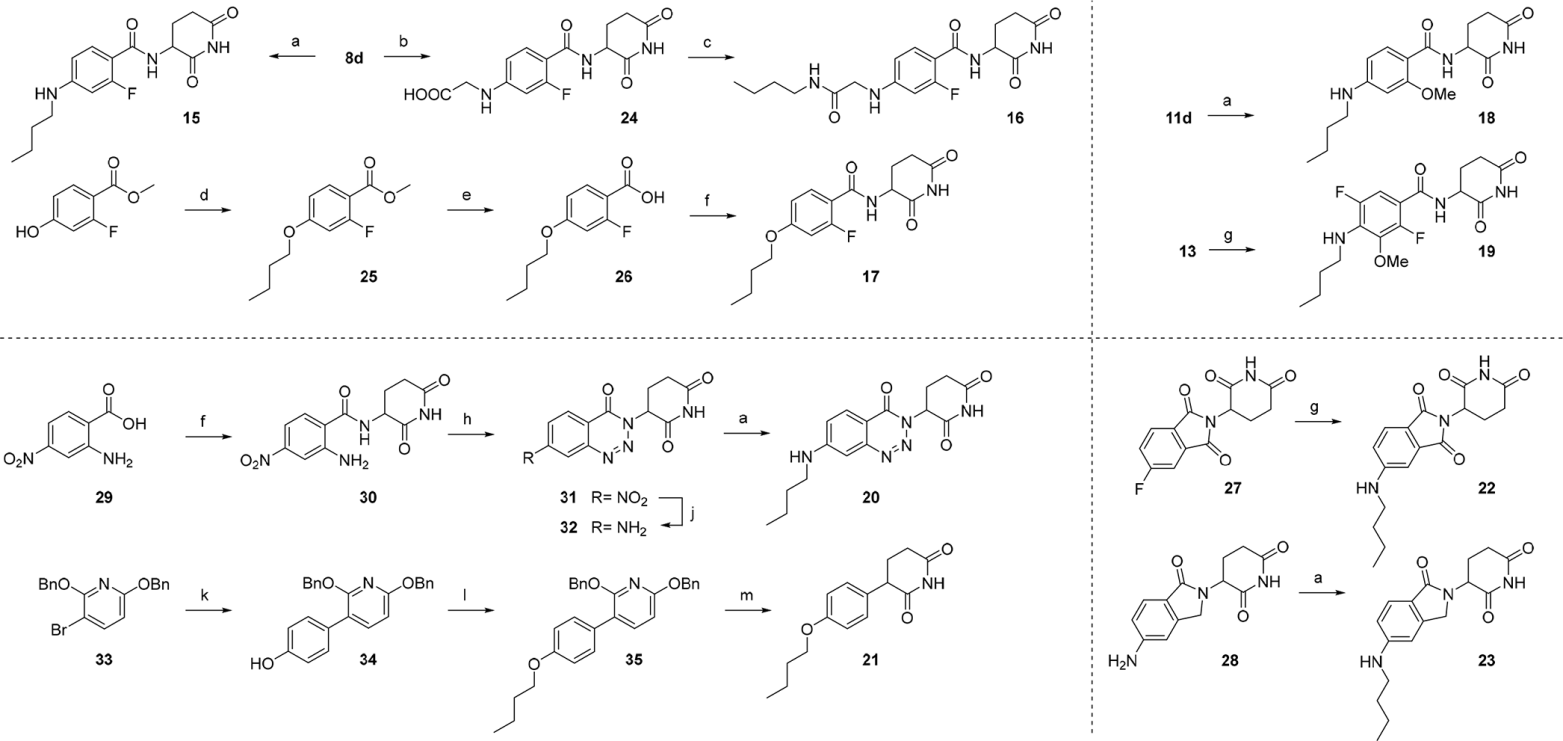
Synthesis of Linker-Functionalized CRBN Ligands Reagents and conditions: (a) (i) butyraldehyde, AcOH, DMF, rt, 1 h; (ii) STAB, 0 °C to rt, 16 h, 26–67%; (b) (i) glyoxylic acid monohydrate, AcOH, DMF, rt, 10 min; (ii) NaCNBH_3_, rt, 2 h, 20%; (c) *n*-butylamine, HATU, DIPEA, DMF, rt, 16 h, 25%; (d) *n*-butanol, PPh_3_, DIAD, THF, 0 °C to rt, 16 h; (e) NaOH, H_2_O, EtOH, rt, 1 h, 30% (2 steps); (f) EDC × HCl, HOBt, DIPEA, DMF, rt, 16 h, 35–43%; (g) *n*-butylamine, DMSO, DIPEA, 90 °C, 16 h, 40–43%; (h) NaNO_2_, AcOH, rt, 4 h, 80%; (j) Fe, THF, H_2_O, rt, 18 h, 44%; (k) (4-hydroxyphenyl)boronic acid, PdCl_2_(dppf) × CH_2_Cl_2_, K_3_PO_4_, dioxane, H_2_O, 110 °C, 18 h, 31%; (l) 1-bromobutane, K_2_CO_3_, DMF, 70 °C, 18 h, 79%; (m) Pd/C, H_2_, THF, rt, 18 h, 26%.

In compound **15** (Scheme 3), a short aliphatic linker was attached to the fluoro-substituted compound **8d** by reductive amination with butyraldehyde. A two-carbon spacer was introduced in **8d** with glycolic acid and coupled with *n*-butylamine to give **16**. Such functional groups are frequently used in degrader design and are known to influence neosubstrate degradation capabilities significantly.^6,14^ A chemically different exit vector was realized in phenol ether **17**. In addition to the prominent scaffold derived from **8d**, we investigated two other hits, **11d** and **11f**. Corresponding linker conjugates were obtained via reductive amination or S_N_Ar, respectively. The same linkers were realized in benzotriazino glutarimide **20**, phenyl glutarimide **21**, pomalidomide-type ligand **22**, and isoindolinone **23**.

*In vitro* testing of the hTBD affinity of compounds **15**–**23** confirmed that linker attachment was well tolerated in all cases (Table 3). Notably, nonbenzamide scaffolds displayed slightly better binding values, with lenalidomide-derived compound **23** having the best CRBN binding. All compounds, except **16**, were moderately hydrophobic with log *D* values between 1.8 and 2.6. In the case of **16**, the additional amide group increases the polar surface area of the molecule, which is also detrimental to plasma protein binding and membrane interactions, as suggested by the significantly lower CHI_IAM_ value. However, the reduced hydrophobicity of **16** is responsible for better lipophilic ligand efficiency (LLE).^55^ Equipping the benzamide scaffold with several hydrophobic substituents (as realized in **19**) did not improve the binding affinity, which is also reflected by its lower LLE. None of the compounds were redox-active, and only **22** was unstable at pH above 8.0, as our high-throughput plate reader assay determined.^49,56^ However, refinement of the stability assessment by using an HPLC-based method and extending the treatment to 24 h at a pH of 7.4 revealed that most binders, with the exception of **21**, were compromised through hydrolytic degradation. These results indicate that both the phthalimide and glutarimide rings are prone to hydrolysis.

**Table 3:** Physicochemical Properties, Binding Affinities, and Cellular Activities of Linker-Connected CRBN Ligands 15–23

Ligand	IC_50_ (μM)^a^	*K*_i_ (μM)^a^	elog *D*_7.4_^b^	LLE^c^	PPB (%)^d^	CHI_IAM_^e^	Redox activity^f^	UV/vis Stability^g^	Buffer stability (%)^h^	IKZF3 deg (%)^i^	SALL4 deg (%)^j^
**15**	62 ± 29	29 ± 15	2.0	2.2	85	29.8	not active	stable	67	<5	14
**16**	55 ± 16	25 ± 8.5	0.7	3.6	30	15.1	not active	stable	56	<5	21
**17**	65 ± 18	31 ± 9.2	2.2	2.0	89	31.2	not active	stable	47	<5	37
**18**	n.d.^k^	n.d.	2.3	n.d.	90	30.3	not active	stable	n.d.	<5	19
**19**	75 ± 30	35 ± 16	2.5	1.6	88	32.4	not active	stable	48	10	26
**20**	16 ± 3.6	4.9 ± 1.9	2.2	2.6	88	31.5	not active	stable	68	12	67
**21**	14 ± 1.5	3.9 ± 0.8	2.6	2.3	89	33.3	not active	n.a.^l^	98	<5	55
**22**	n.d.	n.d.	2.4	2.4	89	32.8	n.a.^l^	<pH 8	65	<5	73
**23**	7.4 ± 1.3	0.43 ± 0.68	1.8	3.3	83	26.4	not active	stable	76	<5	52

a Affinity values determined in a competitive MST assay as described in the method sections (see Supporting Information).

b Distribution coefficients at pH 7.4 were estimated by an HPLC-based method.

c Lipophilic ligand efficiency, LLE = pIC_50_ – elog *D*.

d Plasma protein binding; experimentally determined percentage of compound bound to human serum albumin.

e Chromatographic hydrophobicity index values referring to IAM chromatography (CHI_IAM_ values), an estimate for drug–membrane interactions and permeability.

f Redox activity assays for the detection of compounds that react with reducing agents in redox cycles by forming ROS (H_2_DCFDA assay) or free radicals (resazurin assay); see ref (49).

g UV–vis-based assay for the evaluation of aqueous stability in phosphate buffer at pH 7.0, 8.0, and 9.0 after 4 h of incubation at 37 °C.

h Percentage stability refers to the remaining starting material as determined by HPLC after incubation for 24 h in 50 mM PBS buffer at pH 7.4. Values represent the mean of three independent repeats.

i Percentage of degraded IKZF3 protein after 24 h treatment of MM.1S cells with 0.1 μM of each compound.

j Percentage of degraded SALL4 protein after 24 h treatment of HuH6 cells with 0.1 μM of each compound. Values are normalized to respective loading controls and to DMSO-treated conditions. IKZF3/SALL4 degradation data represent the average of at least two independent biological experiments.

k Not determined.

l Not available due to spectral interference or low absorbance.

Next, effects on the crucial CRBN neosubstrates were evaluated in cellular models (Figure S5). Table 3 summarizes the percentage of IKZF3 degradation after treatment of MM.1S cells with MGs **15**–**23** and SALL4 attenuation in HuH6 cells. These experiments revealed an important caveat of classical degrader scaffolds (as in **22** and **23**), i.e., the potential teratogenic risk through SALL4 degradation. Comparison between **15** and **17** suggests that neosubstrate recruitment is guided by chemical motifs rather than binding affinity or physicochemical properties. Linkages in compounds **15** and **18** were particularly promising in sparing SALL4 degradation.

### Synthesis of Linker Conjugates for PROTAC Development

**Scheme 4:**
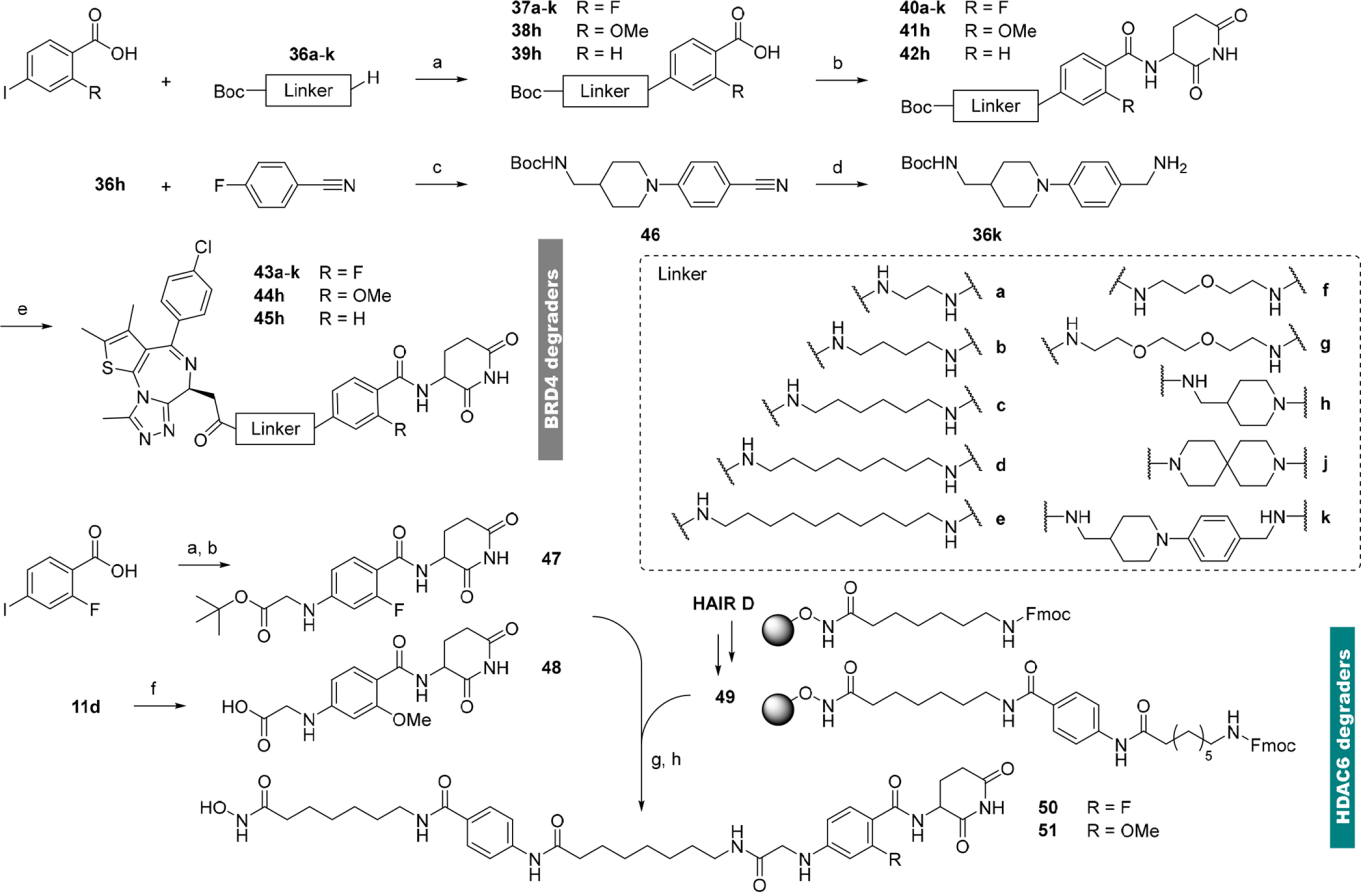
Synthesis of Benzamide-Type PROTACs Targeting BRD4 or HDAC6 Reagents and conditions: (a) (i) aryl iodide, CuI, l-proline, DMSO, 5 min; (ii) primary or secondary amines, DMSO, rt, 3 d, 19–79%; (b) EDC × HCl, HOBt, 3-aminopiperidine-2,6-dione hydrochloride, DIPEA, DMF, rt, 16 h, 27–82%; (c) DIPEA, DMSO, 90 °C, 16 h, 51%; (d) CoCl_2_, NaBH_4_, MeOH, 0 °C, 2 h, 22%; (e) (i) TFA, CH_2_Cl_2_, rt, 2 h; (ii) (+)-JQ1 carboxylic acid, HATU, DIPEA, DMF, rt, 16 h, 31–92%; (f) (i) glyoxylic acid monohydrate, NaOAc, AcOH, MeOH, 0 °C; (ii) NaCNBH_3_, 0 °C, 1 h, 29%; (g) (i) **47**, TFA, CH_2_Cl_2_, rt, 2 h or **48**; (ii) **49**, 20% piperidine, DMF, rt, 2 × 5 min; (iii) **47**-COOH or **48**-COOH, **49**-NH_2_, HATU/EDC × HCl, HOBt × H_2_O, DIPEA, DMF, rt, 18 h; (h) TFA, triisopropylsilane, CH_2_Cl_2_, rt, 1 h, 28–33% (7 steps).

The final goal of this study was the incorporation of newly developed CRBN ligands into prototypic PROTAC molecules. In previous efforts (Scheme 3), linkers were attached via a reductive amination sequence. Although this technique was able to generate certain benzamide-linker conjugates (e.g., **40d**), we found several limitations of this method: the *in situ* generation of aldehydes from PEG-type linkers was unsuccessful, and secondary amine-aryl conjugates could not be realized via this method. Due to the presence of the fluorine atom at the *ortho* position, S_N_Ar reactions had to be omitted. Of note, S_N_Ar reactions also failed in the case of 4-halo-2-methoxy analogs under various conditions.^57,58^ Despite several tested palladium-catalyzed transformations, only a recent variant of an Ullmann reaction was successfully employed to generate linker conjugates (Scheme 4).^59^ The copper-catalyzed amination was carried out starting from 2-fluoro-4-iodobenzoic acid and ω-Boc-protected primary or secondary amines (**36a**–**36k**) under mild conditions. The linker was first attached to the benzoic acid, as aryl iodides led to significant side products in the EDC/HOBt-mediated formation of amide bonds. Applying the Ullmann procedure and subsequent amide bond formation enabled synthesizing a diverse library of linker-functionalized benzamides. These derivatives comprised aliphatic linkers (**40a**–**40e**), PEGylated derivatives (**40f** and **40g**), and rigidified analogs (**40h**–**40k**). Cleavage of the Boc protecting group under acidic conditions and subsequent HATU-mediated coupling with JQ1-acid culminated in 10 different BRD4-targeting PROTACs (**43a**–**43k**). Two more derivatives bearing a methoxy group (**44h**), or an H atom (**45h**) were realized to investigate the influence of the ortho substituent. A second target protein was addressed with **50** and **51**, PROTACs based on a vorinostat-derived histone deacetylase 6 (HDAC6) ligand.^60^ A CRBN recruiter based on type **16** (Table 3) was selected due to its high LLE, similarity to the linker exit vector in A6, and the correct functional carboxylic acid handle required for the assembly of the final degrader via solid-phase synthesis. All HDAC6 degraders were synthesized according to our previously published approach using the preloaded resin **49** as key building block.^60,61^ After Fmoc-deprotection and coupling with the acids (liberated from **11d** or **47**), the final PROTAC **50** and **51** were released from the preloaded resin with trifluoroacetic acid and triisopropylsilane in CH_2_Cl_2_.

### Cellular Evaluations of Novel Benzamide-Type PROTACs

**Figure 3:**
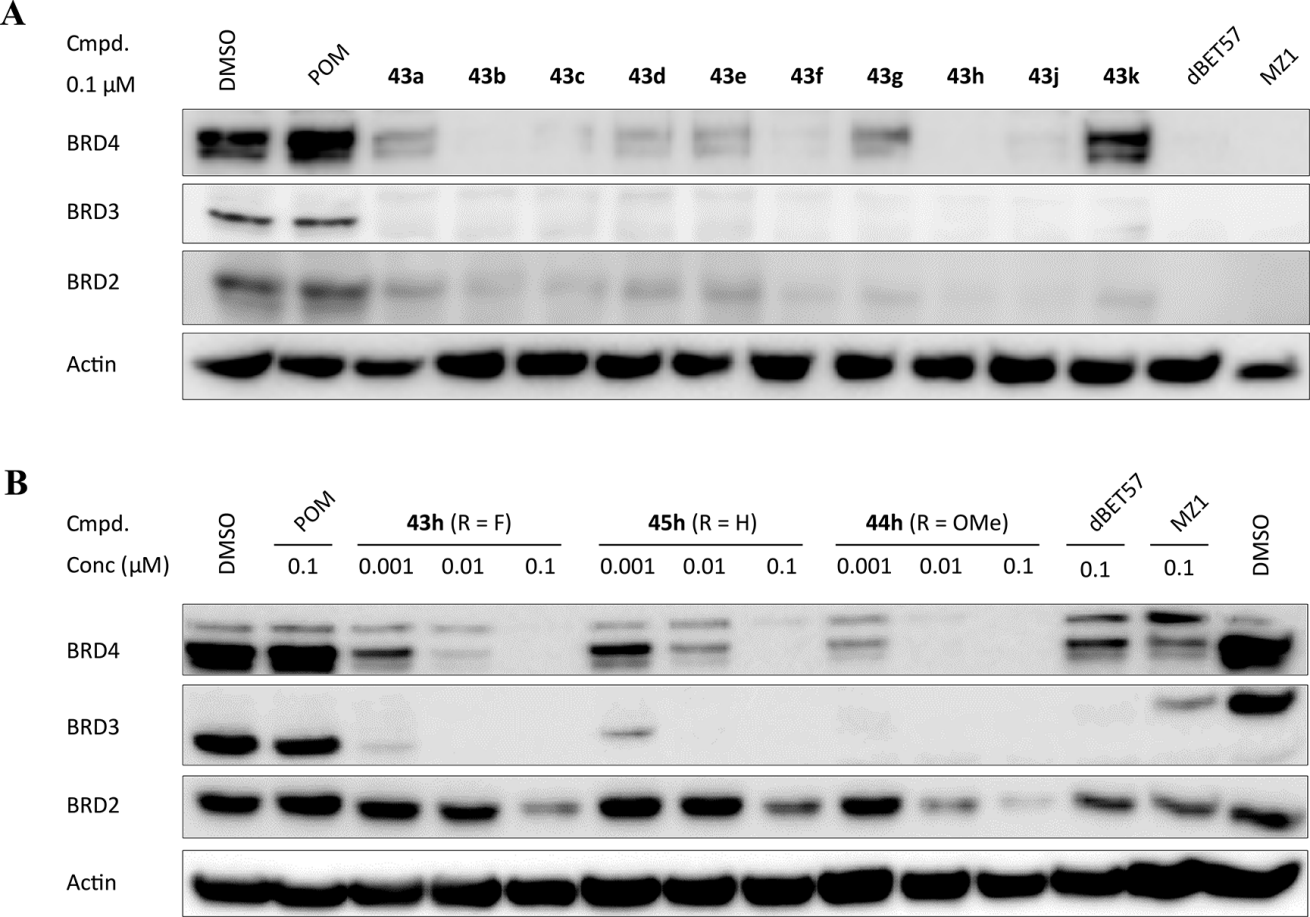
Evaluation of BRD4 PROTACs **43**–**45** in MOLT4 cells. (A) Western blot analyses of BRD4, BRD3, BRD2, and actin protein levels in MOLT4 cells treated with pomalidomide (POM), PROTACs **43** or the CRBN-recruiting reference dBET57 or the VHL-recruiting reference MZ1 for 24 h at 0.1 μM. (B) Western blot analyses of the homologous series **43h**, **44h**, and **45h** at three different concentrations after treatment of MOLT4 cells for 24 h.

The 12 novel BRD4-targeting PROTACs were evaluated in the cell lines MOLT4 (Figure 3) and MV4;11 (Figure S6). For comparison, the established CRBN-recruiting PROTAC dBET57 and the VHL-addressing degrader MZ1 were included in the study.^62,63^ Degradation capabilities (% target degraded) and critical physicochemical properties are summarized in Table 4. All PROTACs, except for **43k**, resulted in a pronounced reduction of BRD4 levels after a 24 h treatment with 0.1 μM of the PROTACs. Protein attenuation was comparable to dBET57 or MZ1 treatments. Surprisingly, even strongly rigidified compounds such as **43j** triggered significant BRD4 degradation. The lipophilicity of PROTACs can be tuned by the choice of the linker, as exemplified by the increase in the log *D* value of homologs **43a**–**43e**. Throughout the series, HSA binding values ranged from 90 to 96% but could be reduced by incorporating PEG-type linkers.^18^ Most PROTACs achieved CHI_IAM_ values between 30 and 50, a previously defined optimal range to achieve good membrane permeability.^64^ Of note, potent BRD4 degraders with molecular weights below 700 g/mol (**43a**) or TPSA as low as 139 Å^2^ (**43j**) were obtained. If intramolecular HBs can lock the bonded isoindole conformation, a superior outcome for derived PROTACs can be expected. Furthermore, the shielding of one HBD could lead to improved cell permeability. Direct comparison between **43h** (R = F), **44h** (R = OMe), and **45h** (R = H) revealed that a more pronounced target degradation was observed for **44h** (Table 4 and Figure 3B). In a concentration-dependent experiment, DC_50,24h_ (BRD4) values of 11, 4.7, and 0.59 nM were determined for dBET57, **43h**, and **44h**, respectively (Figure S7A,B). Next, we assessed the solubility of the BRD4 PROTACs (Table 4) and analyzed structure–property relationships. As expected, solubility of compounds possessing a PEG-type linker had improved solubilities (e.g., **43f** and **43g**), whereas highly hydrophobic compounds such as **43e** bearing alkyl linkers displayed very poor solubility (<1 μg/mL). However, rigidified compounds including lead PROTAC **44h** were poorly soluble in buffer. We expect that the incorporation of protonable nitrogen into the linker moiety could further optimize this feature.

**Table 4:**
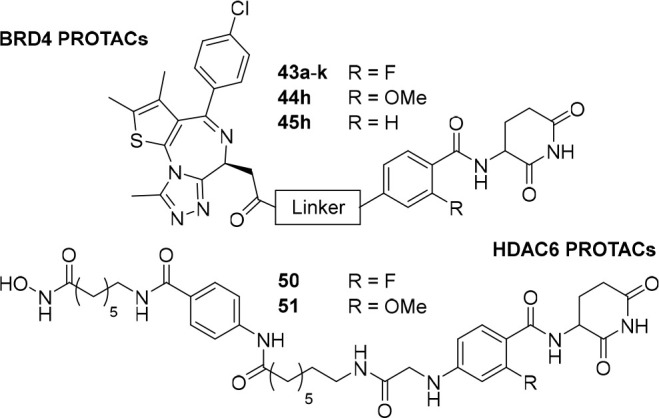
Physicochemical Properties and Target Degradation Capabilities of BRD4-Targeting PROTACs 43–45 and the HDAC6 Degraders 50 and 51

PROTAC	elog *D*_7.4_^a^	PPB (%)^b^	CHI^c^	log(S)^d^	Target deg (%)^e^
dBET57	2.7	93	32.3	–5.3	92
MZ1	2.7	92	31.5	–4.9	94
**43a**	2.1	91	30.4	–4.8	70
**43b**	2.3	92	30.7	–4.5	85
**43c**	2.7	94	34.8	n.a.^g^	80
**43d**	3.3	95	38.7	n.a.	69
**43e**	3.8	96	43.9	n.a.	65
**43f**	2.2	90	29.6	–4.3	81
**43g**	2.3	90	28.8	–3.9	60
**43h**	2.5	93	32.4	–5.8	91
**43j**	3.1	95	36.0	n.a.	83
**43k**	2.9	95	36.7	n.a.	37
**44h**	2.4	92	31.6	–5.6	96
**45h**	2.2	91	30.6	–5.5	83
**50**	1.0	90	27.5	n.d.^f^	81
**51**	0.9	90	28.5	n.d.	87
A6	1.3	91	29.2	n.d.	72

a Distribution coefficients at pH 7.4 were estimated by an HPLC-based method.

b Plasma protein binding; experimentally determined percentage of compound bound to human serum albumin.

c Chromatographic hydrophobicity index values referring to IAM chromatography (CHI_IAM_ values).

d Logarithm of the solubility measured in mol/L at pH 6.8 by an HPLC-based method.

e Percentage of degraded target protein (BRD4 after 24 h treatment of MOLT4 cells with 0.1 μM of PROTACs **43**–**45** or HDAC6 after 24 h treatment of MM.1S cells with 0.1 μM of PROTACs **50**, **51**, or A6). Values are normalized to respective loading controls and to DMSO-treated conditions. BRD4/HDAC6 degradation data represent the average of at least two independent biological experiments.

f Not determined.

g Not available (<1 μg/mL).

**Figure 4:**
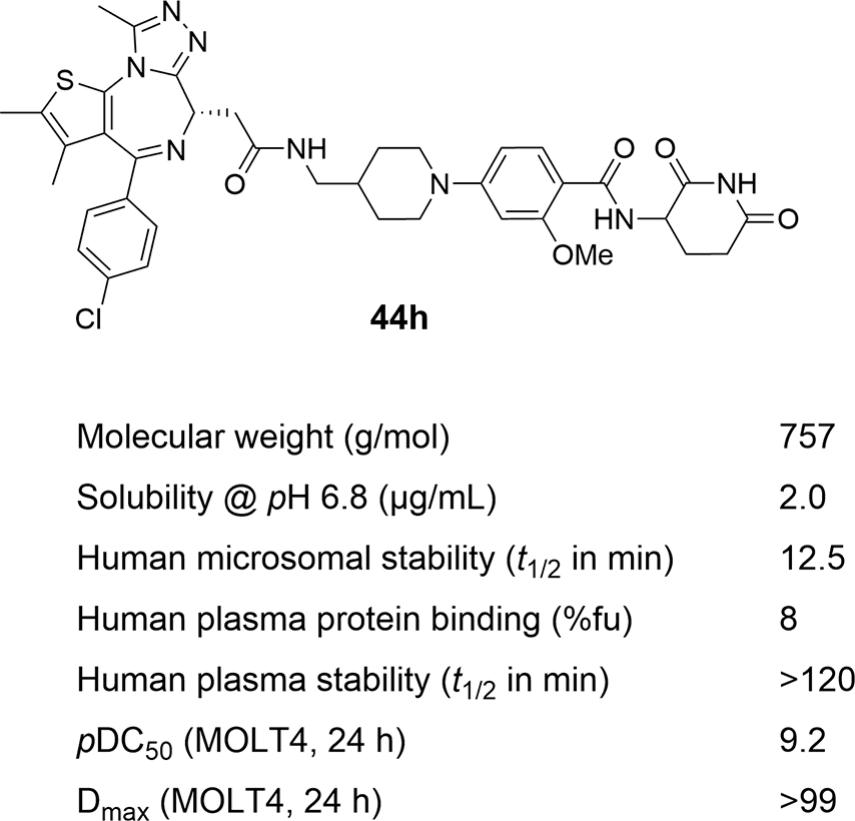
Physicochemical and biochemical profile of PROTAC **44h**.

To evaluate the suitability of leading PROTACs for future *in vivo* applications, we conducted assessments of numerous parameters including chemical solubility and human plasma protein binding, as well as stability in human plasma, human liver microsomes, and aqueous buffer systems. Figure S8 and Figure 4 present physicochemical profiles of compounds **43h** and **44h**, respectively. Noteworthy, benzamide-type PROTACs exhibit exceptional stability in human plasma and demonstrate enhanced water stability compared to phthalimide-based drugs like dBET1 (Figure S8A). The observed moderate stability in human liver microsomes could possibly be attributed to JQ1’s vulnerability to metabolic processes.^65^ This notion is reinforced by the considerable stability shown by benzamide MGs, namely compounds **15** and **18**, across all tested systems (Figure S8B).

**Figure 5:**
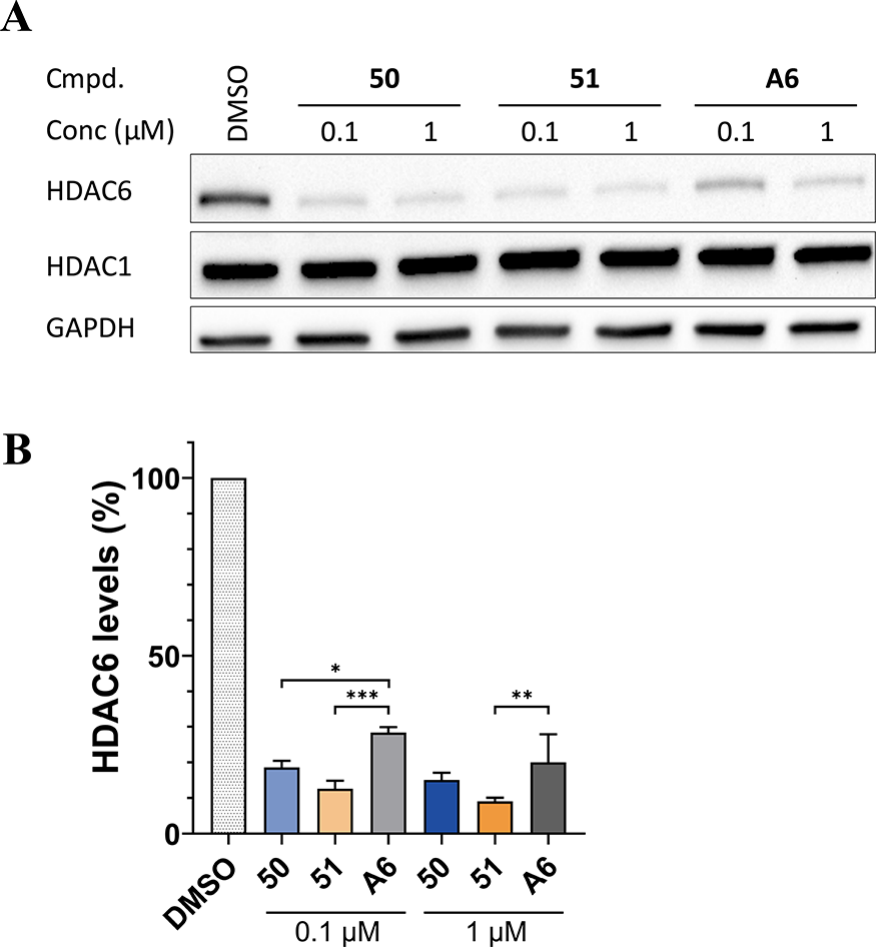
HDAC6 degraders **50**, **51**, and A6 induce degradation of HDAC6. (A) Western blot analyses of HDAC6, HDAC1, and GAPDH protein levels in MM.1S cells treated with compounds **50**, **51**, and A6 for 24 h at the indicated dose. (B) Densitometric analysis of the Western blot assays. Data are normalized to loading control and are shown as mean ± s.d. (*n* = 3, independent experiments). * *p* < 0.05; ** *p* < 0.01; *** *p* < 0.001.

To demonstrate the general utility of novel CRBN ligands described in this study, we sought to degrade a second protein with benzamide-type PROTACs. The HDAC6-targeting degraders **50** and **51** were investigated in the multiple myeloma cell line MM.1S and compared to our previous hit compound A6 (Table 4 and Figure 5A).^60^ Compound **50** or **51** performed well and resulted in even more pronounced degradation of HDAC6 (85% and 91%, respectively) compared to treatment with A6 at 1 μM (80% degradation). Both compounds significantly outperformed this reference compound when tested at concentrations as low as 100 nM (Table 4 and Figure 5B). As expected, compound **51**, bearing the methoxy substituent at the ortho position, performed slightly better than its fluorinated analog **50**. Concentration-dependent experiments revealed DC_50,24h_ (HDAC6) values of 6.5 and 5.8 nM for A6 and **51**, respectively (Figure S7C,D). The control HDAC isoform HDAC1 (class I) was not affected by either compound (Figure 5A).

**Figure 6:**
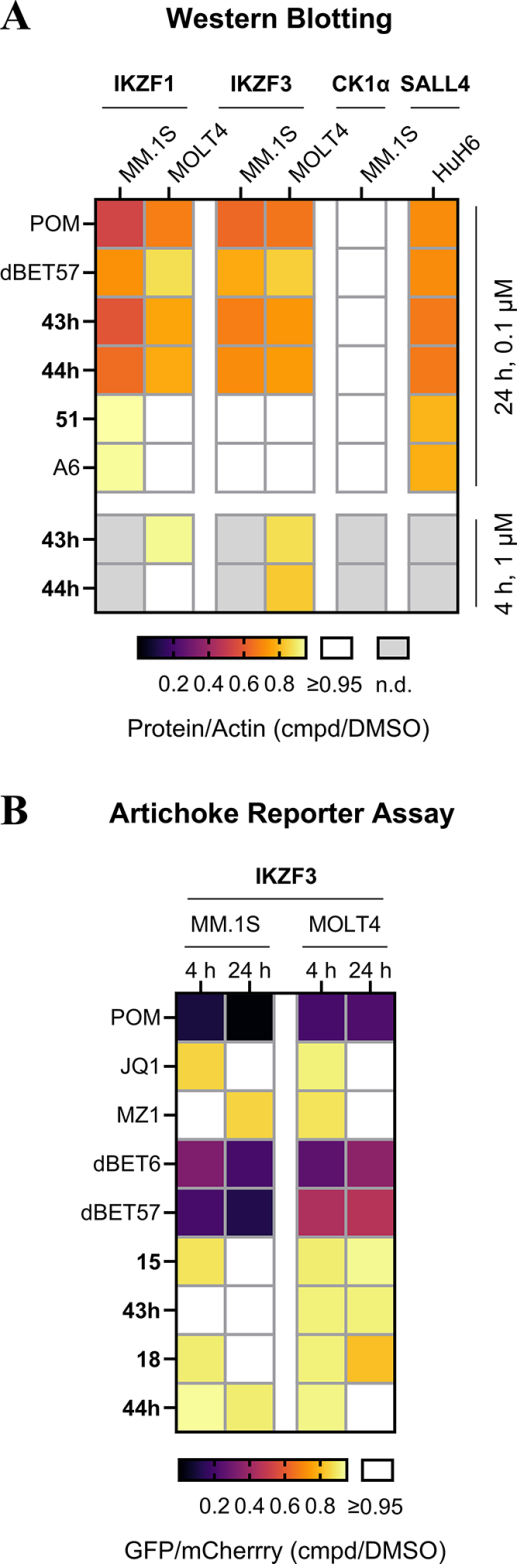
Investigation of neomorphic activities of BRD4 and HDAC6 PROTACs as compared to reference compounds. (A) Quantification of Western blot band intensities after treating the respective cell line with compound at the indicated conditions. Heatmap colors refer to the remaining protein levels after cell treatment. Values are normalized to respective loading controls and to DMSO-treated conditions and represent an average of at least two independent experiments. POM: pomalidomide. dBET57: BRD4 PROTAC. A6: HDAC6 PROTAC. (B) Influence on the pomalidomide-sensitive zinc-finger protein IKZF3 in cells by IMiDs, benzamide-type CRBN ligands, and BRD4 degraders. Cells stably expressing IKZF3-GFP were treated with PROTACs followed by flow cytometry to assess IKZF3 degradation. The graph shows the average of normalized GFP/mCherry ratios for drug-treated versus untreated cells (*n* = 3).

In the final analysis, we aimed to verify that the characteristic prevention of neosubstrate degradation of our benzamide-type ligands is also preserved in their corresponding PROTACs. We subjected MM.1S, MOLT4, or HuH6 cells to treatment with BRD4 PROTACs or pomalidomide for a period of 24 h and assessed protein levels by Western blot (Figure 6A). Remarkably, after treatment with **43h** and **44h**, as well as the reference CRBN-recruiting PROTAC dBET57, a significant reduction in the levels of the lymphoid transcription factors IKZF1 and IKZF3 was seen in the two examined cell lines. The degradation of IKZF1 and IKZF3 was less significant when MM.1S and MOLT4 cells were exposed to PROTACs **43h** and **44h** at a dose of 1 μM for a duration of 4 h. In contrast, the HDAC6-targeting PROTACs **51** and its related PROTAC A6 had negligible effects on the aforementioned transcription factors. Given the role of BRD4 in epigenetic regulation we hypothesized that downregulation of IKZF1 and IKZF3 may arise on the transcriptional level as a consequence of BRD4 inactivation.^66^ In order to distinguish direct effects on protein degradation from transcriptional RNA expression regulation, we applied the Artichoke lentiviral reporter vector that ectopically expresses an IKZF3-GFP fusion protein independent of cellular transcriptional control.^67^ In fact, the abundance of IKZF3-GFP was not significantly altered by either PROTACs **43h** and **44h** (Figure 6B) or their MG precursors **15** and **18**, respectively. In contrast, significant decrease of IKZF3-GFP was observed with PROTACs generated from traditional IMiD-scaffolds (i.e., dBET6 and dBET57), while the BRD4 inhibitor JQ1 or the VHL-hijacking PROTAC MZ1 did not induce such changes. These results confirmed the absence of IMiD neosubstrate degradation and selectivity of benzamide-based PROTACs.

**Figure 7:**
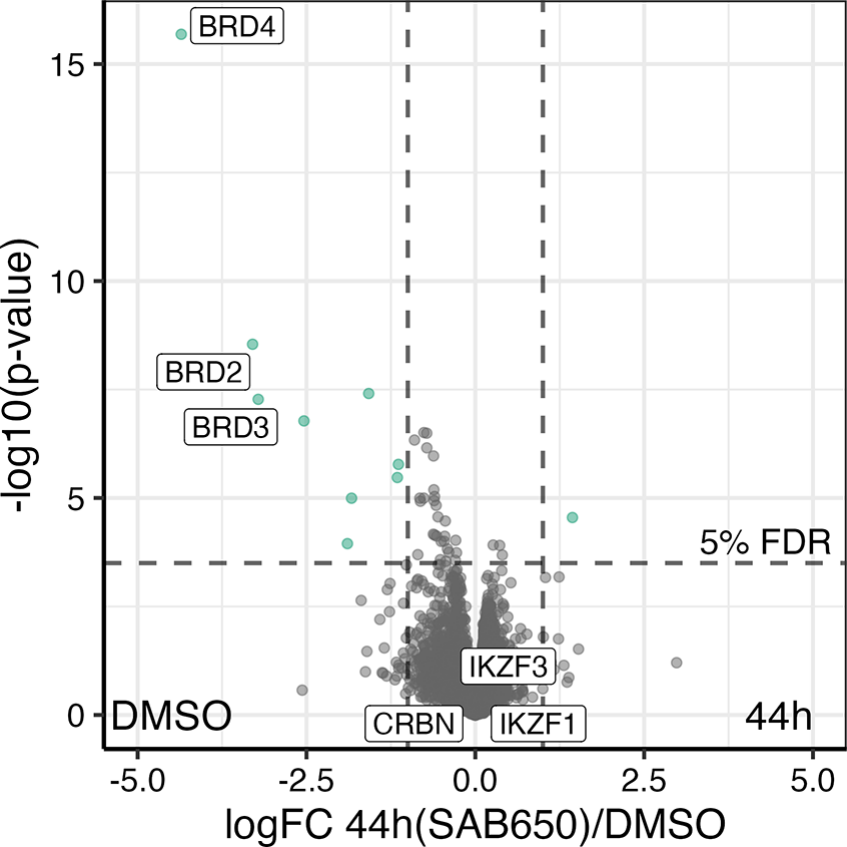
Quantitative proteomics for BRD4 PROTAC **44h**. Total proteome of the MOLT4 cells treated with compound **44h** at 1 μM for 4 h was compared to the proteome of the control cells (DMSO) using the moderated 2-sided 2-sample *t* test (*n* = 5). For each protein the log 10 *p*-value (*y*-axis) is plotted against the log 2 log FC 44h(SAB650)/DMSO. *p*-values were adjusted using the Benjamini–Hochberg procedure. The regulated proteins (5% FDR and absolute log FC > 1) are indicated by color.

Quantitative proteomics (Figure 7) in MOLT4 cells endorsed the slight preference of PROTAC **44h** for BRD4 down-regulation over BRD2 and BRD3, which are all targeted by the POI ligand JQ1. The IMiD neosubstrates IKZF1 and IKZF3, CK1a and GSPT1 remained unaffected by **44h** at a concentration of 1 μM, which is in line with the results from Western blot analyses (Figure 6A) and ectopically expressed IKZF3 (Figure 6B). Consistent with previous findings on other BRD4 degraders such as dBET1 and MZ1, the administration of **44h** resulted in the transcriptional repression of the oncogene MYC (Figure S9).^68^

## Conclusions

**Figure 8:**
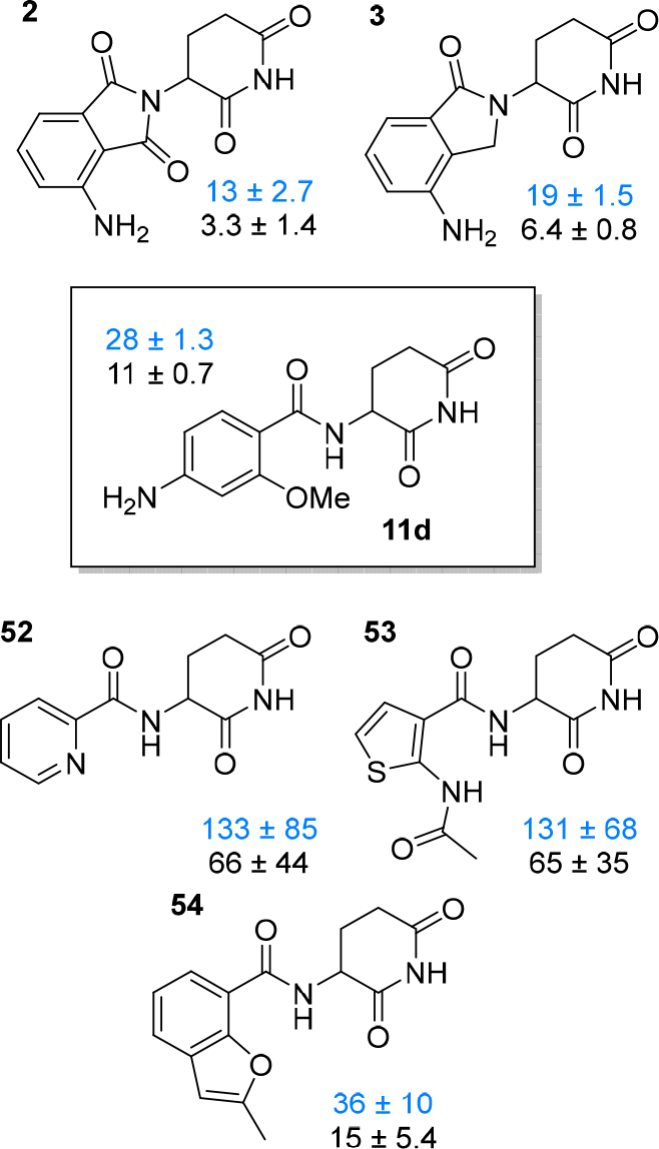
Affinity ranking of ligand **11d** among the established CRBN ligands pomalidomide (**2**) and lenalidomide (**3**), as well as novel chemotypes (**52**–**54**) extracted from patent literature. IC_50_ and derived *K*_i_ values (both in μM) are shown in blue and black, respectively, together with their confidence intervals.

In this study, we systematically investigated an alternative CRBN recruiting scaffold that could find use in various TPD applications. Our results show that ortho-substituted benzamide derivatives represent very attractive derivatives for PROTAC development. Starting from an initial weak CRBN ligand, we rationalized nonbonded interactions between the chemical entities and their receptors.^69^ Part of the medicinal chemistry-driven optimization strategy was the analysis of van der Waals interactions, hydrogen bonds, and hydrophobicity. The compound refinement was accompanied by a critical assessment of important physicochemical and biologically relevant properties. The affinity of the lead compound **11d** to the hTBD was close to those of classical scaffolds such as **2** or **3** (Figure 8) or the natural CRBN degron,^32^ but higher than those of other aryl amides **52**–**54** taken from the recent patent literature (Figure S10).^70−73^ We were able to translate conformationally locked benzamide-type ligands into potent degraders of BRD4 and HDAC6 that possessed advantages compared to IMiD-based prototypes such as dBET57, particularly in terms of stability and neosubstrate selectivity. Our efforts highlight the importance of medicinal chemistry optimization of E3 ligase ligands and PROTAC development to be balanced and multidimensional.

## Experimental Section

### Chemistry

#### General Synthetic Methods and Materials

Preparative column chromatography (CC) was performed using Merck silica gel 60 (0.063–0.200 mm) or using an automated flash chromatography (FC) system puriFlash XS 520Plus. Solid phase synthesis was processed in PP-reactors equipped with a PE frit (5 mL, 25 μm pore size, MultiSyn Tech GmbH). The synthesis was carried out at room temperature on an orbital shaker (RS-OS 5, Phoenix Instruments GmbH). Melting points were determined on a Büchi 510 oil bath apparatus or on a Reichelt hot-stage apparatus and were uncorrected. ^1^H NMR and ^13^C NMR spectra were recorded on a Bruker Avance 400 MHz NMR spectrometer, Bruker Avance 500 MHz NMR spectrometer or on a Bruker Avance III 600 MHz NMR spectrometer, respectively. NMR spectra were processed and analyzed in MestReNova. Chemical shifts are given in parts per million (ppm), coupling constants *J* are given in Hertz, and spin multiplicities are given as s (singlet), d (doublet), t (triplet), q (quartet), or m (multiplet). In the case of overlapping extraneous solvent peaks, multiplet analyses in ^1^H NMR spectra were performed using qGSD (quantitative Global Spectral Deconvolution). Resonance assignments were made based on one- and two-dimensional NMR techniques which include ^1^H, ^13^C, DEPT, HSQC, and HMBC experiments. The purity and identity of the compounds were determined by HPLC-UV obtained on an LC–MS instrument (Agilent Infinity Lab LC/MSD-system with ESI-source coupled with an Agilent HPLC 1260 Infinity II) or separately on an LC instrument (Dionex UltiMate 3000 UHPLC modular system) and High-Resolution Mass Spectrometer (Thermo Scientific Q Exactive Plus). For the former, an EC50/2 Nucleodur C18 Gravity 3 μm column (Macherey-Nagel) was used. The column temperature was 40 °C. HPLC conditions started with 90% H_2_O containing 2 mM NH_4_Ac. The gradient ramped up to 100% MeCN in 10 min, followed by further flushing with 100% MeCN for 5 min. The flow rate was 0.5 mL/min. The samples were dissolved in H_2_O, MeOH, or MeCN (approximately 1 mg/mL), and 2 μL sample solution was injected. Positive total ion scans were observed from 100 to 1000 *m*/*z* (or more if necessary), and UV absorption was detected from 190–600 nm using a diode array detector (DAD). The purity was determined at 220–600 nm, unless indicated otherwise. For the latter, a Waters Acquity UPLC HSS C18 SB column (1.8 μm, 2.1 mm × 50 mm) was used, thermostated at 40 °C. The mobile phase consisted of 0.1% TFA in H_2_O and MeCN, employing the following gradient: 5% to 95% MeCN in 10 min, then 95% MeCN for 4 min, with flow rate of 0.3 mL/min and injection volume of 5 μL. For PROTACs **50** and **51**, the following HPLC systems were used: A Thermo Fisher Scientific Ulti-MateTM 3000 UHPLC system with a Nucleodur 100-5 C18 (250 × 4.6 mm, Macherey Nagel) with a flow rate of 1 mL/min and a temperature of 25 °C with an appropriate gradient was used. For preparative purposes, an AZURA Prep. 500/1000 gradient system with a Nucleodur 110-5 C18 HTec (150 × 32 mm, Macherey Nagel) column and a flow rate of 20 mL/min was used. Detection was implemented with UV absorption measurement at wavelengths of λ = 220 nm and λ = 250 nm. If not stated otherwise, the indicated purity was determined at a wavelength of 250 nm. H_2_O_dd_ with an addition of 0.1% TFA (A) and MeCN (B) were used. The following gradient was used for purification via preparative HPLC: A 95% for 5 min equilibration, in 35 min to B 95% and 8 min isocratic. All compounds that were evaluated in biological assays are >95% pure by LC/MS or UPLC analysis, respectively.

#### General Procedure A. Preparation of Benzamido Glutarimides by Acylation

3-Aminopiperidine-2,6-dione hydrochloride (0.33 g, 2.0 mmol) was suspended in dry CH_2_Cl_2_, and it was cooled to 0 °C. Subsequently, Et_3_N (0.56 mL, 4.0 mmol) and the corresponding *in situ*-generated benzoyl chloride (2.0 mmol) were added. After stirring the mixture for 18 h at rt, it was quenched by the addition of half-saturated NH_4_Cl solution (100 mL), and then extracted with 10% MeOH in EtOAc (2 × 100 mL). The combined organic layers were washed with H_2_O (100 mL) and brine (100 mL), dried over Na_2_SO_4_, filtered, and concentrated *in vacuo*.

#### General Procedure B. Preparation of Benzamido Glutarimides with EDC/HOBt^42^

The corresponding benzoic acid derivative (5.0 mmol), 3-aminopiperidine-2,6-dione hydrochloride (3.29 g, 20 mmol), and DIPEA (3.48 mL, 20 mmol) were suspended in dry DMF (20 mL). Subsequently, EDC × HCl (1.05 g, 5.5 mmol) and HOBt × H_2_O (0.84 g, 5.5 mmol) were added. After stirring the mixture for 18 h at rt, it was quenched by the addition of half-saturated NH_4_Cl solution (100 mL) and then extracted with 10% MeOH in EtOAc (2 × 100 mL). The combined organic layers were washed with H_2_O and brine (each 100 mL), dried over Na_2_SO_4_, filtered, and concentrated *in vacuo*.

#### General Procedure C. Reduction of Nitro Group

The corresponding nitro-containing compound (1.0 mmol) was dissolved in dry DMF (10 mL), and 10% Pd/C (20% by mass) was added under an argon atmosphere. The reaction mixture was stirred under H_2_ (1 atm, balloon) for 18 h at rt. The mixture was filtered through Celite and washed with MeOH (2 × 20 mL), and the filtrate was concentrated.

#### General Procedure D. Copper-Catalyzed Ullmann-Type Coupling

The corresponding 4-iodobenzoic acid derivative (3.0 mmol), anhydrous CuI (114 mg, 0.6 mmol), and l-(−)-proline (138 mg, 1.2 mmol) were placed in a Schlenk tube, and it was evacuated and refilled with argon gas. The solids were suspended in dry DMSO (5 mL) and then stirred under an argon atmosphere for 5 min. Subsequently, a solution of the corresponding amine (9.0 mmol) in dry DMSO (5 mL) was added, and it was mixed for 3 d at rt and protected from light. The mixture was portioned between EtOAc (50 mL) and 10% KHSO_4_ solution (50 mL). The aqueous layer was extracted again with EtOAc (50 mL), and the combined organic layers were washed with 5% LiCl solution and brine (each 50 mL), dried over Na_2_SO_4_, filtered, and concentrated *in vacuo*.

#### General Procedure E. Assembly of BRD4-Targeting PROTACs

The corresponding Boc-protected linker-ligand derivative **40**, **41**, or **42** (0.1 mmol) was dissolved in dry CH_2_Cl_2_ (8 mL) and treated with TFA (2 mL). The mixture was stirred at rt for 2 h, after which it was concentrated *in vacuo*, coevaporated with dry CH_2_Cl_2_ (2 × 5 mL), and dried under high vacuum. Subsequently, (+)-JQ1 carboxylic acid (40 mg, 0.1 mmol), DIPEA (35 μL, 0.2 mmol), and HATU (42 mg, 0.11 mmol) were dissolved in dry DMF (4 mL) and stirred for 5 min at rt. The deprotected amine, dissolved in dry DMF (4 mL) and DIPEA (70 μL, 0.4 mmol), was added, and the combined mixture was stirred at rt for 16 h. It was quenched by the addition of half-saturated NH_4_Cl solution (50 mL) and then extracted with EtOAc (2 × 50 mL). The combined organic layers were washed with 5% LiCl solution and brine (each 50 mL), dried over Na_2_SO_4_, filtered, and concentrated *in vacuo*.

#### Synthetic Details for the Synthesis of Compounds **6** to **53**. *N*-(2,6-Dioxo-3-piperidyl)benzamide (**6a**)

This compound was synthesized as described previously.^34^

#### *N*-(2,6-Dioxo-3-piperidyl)-2,3,4,5-tetrafluorobenzamide (**6b**)

This compound was synthesized as described previously.^34^

#### *N*-(2,6-Dioxo-3-piperidyl)-3-nitrobenzamide (**7a**)

This compound was prepared using the General Procedure A and 3-nitrobenzoyl chloride (0.37 g). The crude product was purified by CC (gradient of petroleum ether/EtOAc 1:2 to EtOAc) to give a colorless solid. Yield 0.34 g (61%); mp 206–210 °C; *R*_f_ = 0.54 (EtOAc); ^1^H NMR (600 MHz, DMSO-*d*_6_) δ 1.98–2.05 (m, 1H), 2.08–2.19 (m, 1H), 2.52–2.60 (m, 1H), 2.77–2.86 (m, 1H), 4.80–4.87 (m, 1H), 7.81 (t, *J* = 8.0 Hz, 1H), 8.29–8.33 (m, 1H), 8.38–8.43 (m, 1H), 8.71 (t, *J* = 2.0 Hz, 1H), 9.17 (d, *J* = 8.3 Hz, 1H), 10.89 (s, 1H); ^13^C NMR (151 MHz, DMSO-*d*_6_) δ 24.22 (C-4′), 31.12 (C-5′), 49.92 (C-3′), 122.13, 126.34, 130.45 (C-2, C-4, C-5), 133.98, 135.42 (C-1, C-6), 148.00 (C-3), 164.25 (CO), 172.11, 173.15 (C-2′, C-6′); LC–MS (ESI) (90% H_2_O to 100% MeCN in 10 min, then 100% MeCN to 20 min, DAD 200–600 nm), *t*_R_ = 6.84 min, 99% purity. *m*/*z* [M + H]^+^ calcd for C_12_H_11_N_3_O_5_, 278.08; found, 278.0.

#### *N*-(2,6-Dioxo-3-piperidyl)-2-fluoro-3-nitrobenzamide (**7b**)

This compound was prepared using the General Procedure A and 2-fluoro-3-nitrobenzoyl chloride (0.41 g). The crude product was recrystallized from 90% EtOAc/*n*-hexanes give a colorless solid. Yield 0.25 g (43%); mp 184–186 °C; *R*_f_ = 0.57 (EtOAc); ^1^H NMR (500 MHz, DMSO-*d*_6_) δ 1.99–2.07 (m, 1H), 2.04–2.14 (m, 1H), 2.50–2.59 (m, 1H), 2.74–2.84 (m, 1H), 4.74–4.83 (m, 1H), 7.53 (t, *J* = 8.0 Hz, 1H), 7.93–7.99 (m, 1H), 8.22–8.29 (m, 1H), 8.93 (d, *J* = 8.2 Hz, 1H), 10.87 (s, 1H); ^13^C NMR (126 MHz, DMSO-*d*_6_) δ 24.08, 30.98, 49.92, 125.26 (d, *J* = 4.7 Hz), 126.64 (d, *J* = 14.5 Hz), 128.12 (d, *J* = 2.2 Hz), 135.66 (d, *J* = 3.7 Hz), 137.70 (d, *J* = 8.5 Hz), 151.93 (d, *J* = 266.2 Hz), 162.31, 171.63, 172.98; LC–MS (ESI) (90% H_2_O to 100% MeCN in 10 min, then 100% MeCN to 20 min, DAD 220–400 nm), *t*_R_ = 2.69 min, 99% purity. *m*/*z* [M + H]^+^ calcd for C_12_H_11_FN_3_O_5_, 296.07; found, 296.1. HRMS (ESI) *m*/*z* [M + H]^+^ calcd for C_12_H_11_FN_3_O_5_, 296.0677; found, 296.0665.

#### *N*-(2,6-Dioxo-3-piperidyl)-4-nitrobenzamide (**7c**)

This compound was prepared using the General Procedure A and 4-nitrobenzoyl chloride (0.37 g). The crude product was purified by FC (40 g, 30 μm, gradient from 60 to 100% EtOAc in *n*-hexanes) to give a colorless solid. Yield 0.26 g (46%); mp 238–240 °C; *R*_f_ = 0.51 (EtOAc); ^1^H NMR (500 MHz, DMSO-*d*_6_) δ 1.96–2.05 (m, 1H), 2.07–2.19 (m, 1H), 2.51–2.60 (m, 1H), 2.75–2.86 (m, 1H), 4.81 (ddd, *J* = 5.1, 8.1, 12.9 Hz, 1H), 8.07–8.13 (m, 2H), 8.31–8.37 (m, 2H), 9.09 (d, *J* = 8.2 Hz, 1H), 10.87 (s, 1H); ^13^C NMR (126 MHz, DMSO-*d*_6_) δ 24.16, 31.08, 49.90, 123.77, 128.96, 139.65, 149.34, 164.73, 172.00, 173.07; LC–MS (ESI) (90% H_2_O to 100% MeCN in 10 min, then 100% MeCN to 20 min, DAD 200–600 nm), *t*_R_ = 2.93 min, 99% purity. *m*/*z* [M + H]^+^ calcd for C_12_H_11_N_3_O_5_, 278.08; found, 278.1. HRMS (ESI) *m*/*z* [M + H]^+^ calcd for C_12_H_11_N_3_O_5_, 278.0772; found, 278.0766.

#### *N*-(2,6-Dioxo-3-piperidyl)-2-fluoro-4-nitrobenzamide (**7d**)

This compound was prepared using the General Procedure A and 2-fluoro-4-nitrobenzoyl chloride (0.41 g). The crude product was recrystallized from 80% EtOAc/*n*-hexanes give a colorless solid. Yield 0.16 g (27%); mp 196–200 °C; *R*_f_ = 0.60 (EtOAc); ^1^H NMR (500 MHz, DMSO-*d*_6_) δ 1.99–2.18 (m, 2H), 2.50–2.59 (m, 1H), 2.74–2.85 (m, 1H), 4.79 (ddd, *J* = 5.7, 8.2, 12.1 Hz, 1H), 7.87 (t, *J* = 7.7 Hz, 1H), 8.16 (dd, *J* = 2.2, 8.5 Hz, 1H), 8.22 (dd, *J* = 2.2, 9.8 Hz, 1H), 8.94 (d, *J* = 8.2 Hz, 1H), 10.87 (s, 1H); ^13^C NMR (126 MHz, DMSO-*d*_6_) δ 24.05, 30.98, 49.89, 112.32 (d, *J* = 27.8 Hz), 119.81 (d, *J* = 3.6 Hz), 129.81 (d, *J* = 15.5 Hz), 131.41 (d, *J* = 3.4 Hz), 149.48 (d, *J* = 8.9 Hz), 158.70 (d, *J* = 253.8 Hz), 162.48, 171.62, 172.98; LC–MS (ESI) (90% H_2_O to 100% MeCN in 10 min, then 100% MeCN to 20 min, DAD 200–600 nm), *t*_R_ = 2.98 min, 99% purity. *m*/*z* [M + H]^+^ calcd for C_12_H_11_FN_3_O_5_, 296.07; found, 296.1. HRMS (ESI) *m*/*z* [M + H]^+^ calcd for C_12_H_11_FN_3_O_5_, 296.0677; found, 296.0667.

#### *N*-(2,6-Dioxo-3-piperidyl)-2-nitrobenzamide (**7e**)

This compound was prepared using the General Procedure A and 2-nitrobenzoyl chloride (0.37 g). The crude product was purified by FC (40 g, 30 μm, gradient from 60 to 100% EtOAc in *n*-hexanes) to give a beige solid. Yield 0.31 g (56%); mp 174–176 °C; *R*_f_ = 0.53 (EtOAc); ^1^H NMR (500 MHz, DMSO-*d*_6_) δ 2.00–2.10 (m, 2H), 2.50–2.59 (m, 1H), 2.73–2.84 (m, 1H), 4.69–4.77 (m, 1H), 7.64 (dd, *J* = 1.5, 7.6 Hz, 1H), 7.67–7.74 (m, 1H), 7.78–7.85 (m, 1H), 8.05 (dd, *J* = 1.2, 8.2 Hz, 1H), 9.01 (d, *J* = 8.2 Hz, 1H), 10.85 (s, 1H); ^13^C NMR (126 MHz, DMSO-*d*_6_) δ 24.01, 30.85, 49.63, 124.25, 129.23, 131.10, 132.06, 133.79, 147.14, 165.53, 171.76, 173.01; LC–MS (ESI) (90% H_2_O to 100% MeCN in 10 min, then 100% MeCN to 20 min, DAD 220–400 nm), *t*_R_ = 1.29 min, 99% purity. *m*/*z* [M + H]^+^ calcd for C_12_H_11_N_3_O_5_, 278.08; found, 278.1. HRMS (ESI) *m*/*z* [M + H]^+^ calcd for C_12_H_11_N_3_O_5_, 278.0772; found, 278.0765.

#### *N*-(2,6-Dioxo-3-piperidyl)-2-fluoro-6-nitrobenzamide (**7f**)

This compound was prepared using the General Procedure A and 2-fluoro-6-nitrobenzoyl chloride (0.41 g). The crude product was purified by FC (40 g, 30 μm, gradient from 60 to 100% EtOAc in *n*-hexanes) to give a gray solid. Yield 0.54 g (91%); mp >240 °C; *R*_f_ = 0.64 (EtOAc); ^1^H NMR (500 MHz, DMSO-*d*_6_) δ 1.88–2.00 (m, 1H), 2.07–2.16 (m, 1H), 2.48–2.59 (m, 1H), 2.71–2.82 (m, 1H), 4.73–4.82 (m, 1H), 7.70–7.81 (m, 2H), 8.01 (dd, *J* = 1.8, 7.5 Hz, 1H), 9.15 (d, *J* = 7.9 Hz, 1H), 10.85 (s, 1H); ^13^C NMR (126 MHz, DMSO-*d*_6_) δ 24.04, 30.67, 49.83, 120.53 (d, *J* = 3.1 Hz), 121.38 (d, *J* = 24.2 Hz), 122.18 (d, *J* = 22.3 Hz), 131.78 (d, *J* = 8.8 Hz), 146.79 (d, *J* = 5.2 Hz), 158.57 (d, *J* = 249.8 Hz); LC–MS (ESI) (90% H_2_O to 100% MeCN in 10 min, then 100% MeCN to 20 min, DAD 200–600 nm), *t*_R_ = 1.33 min, 98% purity. *m*/*z* [M + H]^+^ calcd for C_12_H_11_FN_3_O_5_, 296.07; found, 296.1. HRMS (ESI) *m*/*z* [M + H]^+^ calcd for C_12_H_11_FN_3_O_5_, 296.0677; found, 296.0669.

#### 3-Amino-*N*-(2,6-dioxo-3-piperidyl)benzamide (**8a**)

This compound was prepared using the General Procedure C and nitro compound **7a** (0.28 g). The crude product was purified by CC (gradient of EtOAc 10% EtOH/EtOAc) to give a colorless solid. Yield 0.54 g (87%); mp 196–198 °C; *R*_f_ = 0.49 (10% EtOH/EtOAc); ^1^H NMR (600 MHz, DMSO-*d*_6_) δ 1.90–2.01 (m, 1H), 2.01–2.14 (m, 1H), 2.49–2.59 (m, 1H), 2.65–2.87 (m, 1H), 4.73 (ddd, *J* = 5.3, 8.3, 12.4 Hz, 1H), 5.23 (s, 2H), 6.63–6.76 (m, 1H), 6.90–7.00 (m, 1H), 7.04 (t, *J* = 2.0 Hz, 1H), 7.08 (t, *J* = 7.8 Hz, 1H), 8.47 (d, *J* = 8.4 Hz, 1H), 10.80 (s, 1H); ^13^C NMR (151 MHz, DMSO-*d*_6_) δ 24.39, 31.16, 49.56, 113.02, 114.54, 116.82, 128.83, 135.05, 148.88, 167.01, 172.48, 173.21; LC–MS (ESI) (90% H_2_O to 100% MeCN in 10 min, then 100% MeCN to 20 min, DAD 200–400 nm), *t*_R_ = 3.02 min, 99% purity. *m*/*z* [M + H]^+^ calcd for C_12_H_14_N_3_O_3_, 248.10; found, 247.9. HRMS (ESI) *m*/*z* [M + H]^+^ calcd for C_12_H_14_N_3_O_3_, 248.1030; found, 248.1022.

#### 3-Amino-*N*-(2,6-dioxo-3-piperidyl)-2-fluorobenzamide (**8b**)

This compound was prepared using the General Procedure C and nitro compound **7b** (0.30 g). The crude product was recrystallized from 80% EtOAc/*n*-hexanes give a beige solid. Yield 98 mg (37%); mp 186–188 °C; *R*_f_ = 0.47 (EtOAc); ^1^H NMR (500 MHz, DMSO-*d*_6_) δ 1.96–2.13 (m, 2H), 2.51–2.56 (m, 1H), 2.70–2.82 (m, 1H), 4.68–4.77 (m, 1H), 5.30 (s, 2H), 6.74 (t, *J* = 6.8 Hz, 1H), 6.86 (t, *J* = 8.2 Hz, 1H), 6.92 (t, *J* = 7.6 Hz, 1H), 8.38 (dd, *J* = 2.8, 8.5 Hz, 1H), 10.80 (s, 1H); ^13^C NMR (126 MHz, DMSO-*d*_6_) δ 24.22, 31.02, 49.67, 115.91, 118.16 (d, *J* = 5.2 Hz), 123.44 (d, *J* = 11.4 Hz), 124.16 (d, *J* = 3.7 Hz), 137.14 (d, *J* = 13.4 Hz), 147.79 (d, *J* = 243.0 Hz), 164.34, 172.04, 173.06; LC–MS (ESI) (90% H_2_O to 100% MeCN in 10 min, then 100% MeCN to 20 min, DAD 200–600 nm), *t*_R_ = 0.82 min, 99% purity. *m*/*z* [M + H]^+^ calcd for C_12_H_13_FN_3_O_3_, 266.09; found, 266.1. HRMS (ESI) *m*/*z* [M + H]^+^ calcd for C_12_H_13_FN_3_O_3_, 266.0936; found, 266.0926.

#### 4-Amino-*N*-(2,6-dioxo-3-piperidyl)benzamide (**8c**)

Nitro precursor **7c** (0.28 g, 1.0 mmol) was dissolved in EtOH (10 mL) and AcOH (2.5 mL). A mixture of Fe (0.28 g, 5.0 mmol) in H_2_O (10 mL) and AcOH (10 mL) was added, and the combined mixture was stirred at 110 °C for 30 min. After cooling, it was diluted with EtOAc (100 mL) and carefully quenched with saturated NaHCO_3_ solution (100 mL). The organic layer was separated, washed with brine (100 mL), dried over Na_2_SO_4_, filtered, and concentrated *in vacuo*. The crude product was purified by FC (40 g, 30 μm, gradient from 60 to 100% EtOAc in *n*-hexanes) to give a beige solid. Yield 30 mg (14%); mp 234–236 °C; *R*_f_ = 0.32 (EtOAc); ^1^H NMR (500 MHz, DMSO-*d*_6_) δ 1.88–1.97 (m, 1H), 2.02–2.14 (m, 1H), 2.53 (t, *J* = 3.8 Hz, 1H), 2.70–2.81 (m, 1H), 4.70 (ddd, *J* = 5.3, 8.4, 13.1 Hz, 1H), 5.61 (s, 2H), 6.52–6.57 (m, 2H), 7.55–7.60 (m, 2H), 8.23 (d, *J* = 8.3 Hz, 1H), 10.75 (s, 1H); ^13^C NMR (126 MHz, DMSO-*d*_6_) δ 24.59, 31.17, 49.43, 112.66, 120.69, 129.02, 151.98, 166.17, 172.75, 173.20; LC–MS (ESI) (90% H_2_O to 100% MeCN in 10 min, then 100% MeCN to 20 min, DAD 200–600 nm), *t*_R_ = 0.56 min, 98% purity. *m*/*z* [M + H]^+^ calcd for C_12_H_14_N_3_O_3_, 248.10; found, 248.1. HRMS (ESI) *m*/*z* [M + H]^+^ calcd for C_12_H_14_N_3_O_3_, 248.1030; found, 248.1037.

#### 4-Amino-*N*-(2,6-dioxo-3-piperidyl)-2-fluorobenzamide (**8d**)

This compound was prepared using the General Procedure C and nitro compound **7d** (0.30 g). The crude product was purified by FC (40 g, 30 μm, gradient from 60 to 100% EtOAc in *n*-hexanes) to give a beige solid. Yield 0.20 g (75%); mp 216–218 °C; *R*_f_ = 0.41 (EtOAc); ^1^H NMR (500 MHz, DMSO-*d*_6_) δ 1.95–2.15 (m, 2H), 2.50–2.56 (m, 1H), 2.69–2.81 (m, 1H), 4.65–4.75 (m, 1H), 5.97 (s, 2H), 6.30 (dd, *J* = 2.1, 14.5 Hz, 1H), 6.41 (dd, *J* = 2.1, 8.6 Hz, 1H), 7.49 (t, *J* = 8.8 Hz, 1H), 7.81 (t, *J* = 7.6 Hz, 1H), 10.78 (s, 1H); ^13^C NMR (126 MHz, DMSO-*d*_6_) δ 24.35, 31.13, 49.87, 99.29 (d, *J* = 26.5 Hz), 108.04 (d, *J* = 12.4 Hz), 109.72, 132.09 (d, *J* = 4.7 Hz), 153.98 (d, *J* = 12.7 Hz), 161.78 (d, *J* = 245.8 Hz), 163.36 (d, *J* = 2.8 Hz), 172.50, 173.08; LC–MS (ESI) (90% H_2_O to 100% MeCN in 10 min, then 100% MeCN to 20 min, DAD 200–600 nm), *t*_R_ = 1.01 min, 95% purity. *m*/*z* [M + H]^+^ calcd for C_12_H_13_FN_3_O_3_, 266.09; found, 266.1. HRMS (ESI) *m*/*z* [M + H]^+^ calcd for C_12_H_13_FN_3_O_3_, 266.0936; found, 266.0925.

#### 2-Amino-*N*-(2,6-dioxo-3-piperidyl)benzamide (**8e**)

This compound was synthesized by analogy with **8c**, but using precursor **7e** (0.28 g, 1.0 mmol). The crude product was recrystallized from 20% EtOH/EtOAc give a colorless solid. Yield 0.18 g (73%); mp 244–248 °C; *R*_f_ = 0.52 (EtOAc); ^1^H NMR (600 MHz, DMSO-*d*_6_) δ 1.90–1.99 (m, 1H), 2.06–2.16 (m, 1H), 2.50–2.57 (m, 1H), 2.73–2.82 (m, 1H), 4.68–4.75 (m, 1H), 6.39 (s, 2H), 6.49–6.55 (m, 1H), 6.70 (dd, *J* = 1.2, 8.3 Hz, 1H), 7.12–7.18 (m, 1H), 7.50 (dd, *J* = 1.6, 8.0 Hz, 1H), 8.44 (d, *J* = 8.3 Hz, 1H), 10.80 (s, 1H); ^13^C NMR (151 MHz, DMSO-*d*_6_) δ 24.33, 31.15, 49.22, 114.17, 114.67, 116.55, 128.25, 132.09, 149.89, 168.80, 172.57, 173.17; LC–MS (ESI) (90% H_2_O to 100% MeCN in 10 min, then 100% MeCN to 20 min, DAD 200–600 nm), *t*_R_ = 1.46 min, 99% purity. *m*/*z* [M + H]^+^ calcd for C_12_H_14_N_3_O_3_, 248.10; found, 248.1. HRMS (ESI) *m*/*z* [M + H]^+^ calcd for C_12_H_14_N_3_O_3_, 248.1030; found, 248.1020.

#### 2-Amino-*N*-(2,6-dioxo-3-piperidyl)-6-fluorobenzamide (**8f**)

This compound was prepared using the General Procedure C and nitro compound **7f** (0.30 g). The crude product was triturated with EtOAc and dried to give a beige solid. Yield 0.20 g (75%); mp 234–236 °C; *R*_f_ = 0.60 (EtOAc); ^1^H NMR (600 MHz, DMSO-*d*_6_) δ 1.94–2.02 (m, 1H), 2.04–2.14 (m, 1H), 2.50–2.56 (m, 1H), 2.71–2.82 (m, 1H), 4.69–4.77 (m, 1H), 5.98 (s, 2H), 6.28–6.34 (m, 1H), 6.50 (d, *J* = 8.2 Hz, 1H), 7.04–7.11 (m, 1H), 8.50 (dd, *J* = 2.8, 8.3 Hz, 1H), 10.87 (s, 1H); ^13^C NMR (151 MHz, DMSO-*d*_6_) δ 23.99, 31.11, 49.61, 101.69 (d, *J* = 23.0 Hz), 107.44 (d, *J* = 18.8 Hz), 111.42, 131.36 (d, *J* = 11.3 Hz), 149.53 (d, *J* = 6.4 Hz), 160.43 (d, *J* = 243.2 Hz), 164.59, 172.83 (d, *J* = 84.6 Hz); LC–MS (ESI) (90% H_2_O to 100% MeCN in 10 min, then 100% MeCN to 20 min, DAD 200–600 nm), *t*_R_ = 2.02 min, 99% purity. *m*/*z* [M + H]^+^ calcd for C_12_H_13_FN_3_O_3_, 266.09; found, 266.0. HRMS (ESI) *m*/*z* [M + H]^+^ calcd for C_12_H_13_FN_3_O_3_, 266.0936; found, 266.0927.

#### 2-Chloro-*N*-(2,6-dioxo-3-piperidyl)-4-nitrobenzamide (**10a**)

This compound was prepared using the General Procedure B and 2-chloro-4-nitrobenzoic acid (1.00 g). The crude product was purified by CC (CH_2_Cl_2_/MeOH 20:1) to give a gray solid. Yield 1.12 g (74%); mp 204–207 °C; *R*_f_ = 0.20 (CH_2_Cl_2_/MeOH 20:1); ^1^H NMR (400 MHz, DMSO-*d*_6_) δ 2.01–2.10 (m, 2H), 2.55–2.61 (m, 1H), 2.74–2.87 (m, 1H), 4.75–4.84 (m, 1H), 7.73 (d, *J* = 8.4 Hz, 1H), 8.28 (dd, *J* = 8.4, 2.2 Hz, 1H), 8.36 (d, *J* = 2.2 Hz, 1H), 9.08 (d, *J* = 8.3 Hz, 1H), 10.92 (s, 1H); ^13^C NMR (101 MHz, DMSO-*d*_6_) δ 23.99, 30.78, 49.47, 122.46, 124.68, 130.07, 131.12, 141.93, 148.33, 164.92, 171.50, 172.91; UPLC-retention time, 3.51 min; purity 90%. HRMS (ESI) *m*/*z* [M + H]^+^ calcd for C_12_H_9_N_3_O_5_Cl, 310.0236; found, 310.0237.

#### *N*-(2,6-Dioxo-3-piperidyl)-4-nitro-2-(trifluoromethyl)benzamide (**10b**)

This compound was prepared using the General Procedure A (5 mmol scale) and 4-nitro-2-(trifluoromethyl)benzoyl chloride (1.27 g). The crude product was purified by FC (80 g, 30 μm, gradient from 10 to 100% EtOAc in cyclohexane) to give a beige solid. Yield 1.03 g (60%); mp 210–212 °C; *R*_f_ = 0.64 (EtOAc); ^1^H NMR (500 MHz, DMSO-*d*_6_) δ 1.96–2.04 (m, 1H), 2.01–2.10 (m, 1H), 2.50–2.59 (m, 1H), 2.70–2.85 (m, 1H), 4.73–4.81 (m, 1H), 7.85 (d, *J* = 8.4 Hz, 1H), 8.50 (d, *J* = 2.3 Hz, 1H), 8.60 (dd, *J* = 2.3, 8.4 Hz, 1H), 9.13 (d, *J* = 8.2 Hz, 1H), 10.88 (s, 1H); ^13^C NMR (126 MHz, DMSO-*d*_6_) δ 23.96, 30.91, 49.67, 121.82 (d, *J* = 5.1 Hz), 122.64 (q, *J* = 274.4 Hz), 127.49 (q, *J* = 33.1 Hz), 127.78, 130.85, 141.29, 148.02, 165.58, 171.58, 172.98; LC–MS (ESI) (90% H_2_O to 100% MeCN in 10 min, then 100% MeCN to 20 min, DAD 220–600 nm), *t*_R_ = 4.08 min, 99% purity. *m*/*z* [M – H]^−^ calcd for C_13_H_9_F_3_N_3_O_5_, 344.05; found, 344.1. HRMS (ESI) *m*/*z* [M – H]^−^ calcd for C_13_H_9_F_3_N_3_O_5_, 344.0500; found, 344.0499.

#### *N*-(2,6-Dioxo-3-piperidyl)-2-methyl-4-nitrobenzamide (**10c**)

This compound was prepared using the General Procedure A (5 mmol scale) and 2-methyl-4-nitrobenzoyl chloride (1.00 g). The crude product was suspended in 1 M HCl (aq), and the solid product was collected by filtration, washed with H_2_O (2 × 10 mL), Et_2_O (2 × 10 mL), and dried *in vacuo* to give a gray solid. Yield 1.17 g (80%); mp 233–236 °C; *R*_f_ = 0.25 (CH_2_Cl_2_/MeOH 20:1); ^1^H NMR (400 MHz, DMSO-*d*_6_) δ 1.98–2.14 (m, 2H), 2.49 (s, 3H), 2.53–2.60 (m, 1H), 2.75–2.86 (m, 1H), 4.73–4.83 (m, 1H), 7.59 (d, *J* = 8.3 Hz, 1H), 8.12 (dd, *J* = 8.4, 2.4 Hz, 1H), 8.16 (d, *J* = 2.3 Hz, 1H), 8.87 (d, *J* = 8.4 Hz, 1H), 10.90 (s, 1H); ^13^C NMR (101 MHz, DMSO-*d*_6_) δ 19.00, 23.99, 30.96, 49.37, 120.86, 124.98, 128.38, 137.69, 142.76, 147.70, 167.59, 171.84, 173.00; UPLC-retention time, 3.44 min; purity 89%. HRMS (ESI) *m*/*z* [M + H]^+^ calcd for C_13_H_14_N_3_O_5_, 292.0928; found, 292.0927.

#### *N*-(2,6-Dioxo-3-piperidyl)-2-methoxy-4-nitrobenzamide (**10d**)

This compound was prepared using the General Procedure A (5 mmol scale) and 2-methoxy-4-nitrobenzoyl chloride (1.08 g). The crude product was suspended in 1 M HCl (aq), and the solid product was collected by filtration, washed with H_2_O (2 × 10 mL), Et_2_O (2 × 10 mL), and dried *in vacuo* to give a gray solid. Yield 1.27 g (83%); mp 179–181 °C; *R*_f_ = 0.22 (CH_2_Cl_2_/MeOH 9:1); ^1^H NMR (400 MHz, DMSO-*d*_6_) δ 2.03–2.19 (m, 2H), 2.53–2.58 (m, 1H), 2.74–2.85 (m, 1H), 4.01 (s, 3H), 4.74–4.84 (m, 1H), 7.89 (s, 1H), 7.91–7.96 (m, 2H), 8.76 (d, *J* = 7.8 Hz, 1H), 10.92 (s, 1H); ^13^C NMR (101 MHz, DMSO-*d*_6_) δ 23.99, 30.90, 49.92, 56.76, 107.13, 115.42, 128.88, 131.43, 149.69, 157.31, 163.58, 171.92, 172.95; UPLC-retention time, 3.80 min; purity 90%. HRMS (ESI) *m*/*z* [M + H]^+^ calcd for C_13_H_14_N_3_O, 308.0877; found, 308.0876.

#### *N*-(2,6-Dioxo-3-piperidyl)-2-hydroxy-4-nitrobenzamide (**10e**)

This compound was prepared using the General Procedure B and 2-hydroxy-4-nitrobenzoic acid (0.92 g). The crude product was purified by CC (CH_2_Cl_2_/MeOH 20:1) to give an off-white solid. Yield 0.77 g (53%); mp 195–198 °C; *R*_f_ = 0.18 (CH_2_Cl_2_/MeOH 20:1); ^1^H NMR (400 MHz, DMSO-*d*_6_) δ 2.07–2.18 (m, 2H), 2.54–2.59 (m, 1H), 2.74–2.86 (m, 1H), 4.79–4.87 (m, 1H), 7.74 (d, *J* = 2.2 Hz, 1H), 7.77 (dd, *J* = 8.6, 2.3 Hz, 1H), 8.09 (d, *J* = 8.6 Hz, 1H), 9.18 (d, *J* = 7.5 Hz, 1H), 10.96 (s, 1H), 12.43 (s, 1H); ^13^C NMR (101 MHz, DMSO-*d*_6_) δ 23.87, 30.95, 50.03, 111.71, 113.57, 123.05, 131.00, 150.01, 158.20, 165.27, 171.89, 172.92; UPLC-retention time, 3.91 min; purity 95%. HRMS (ESI) *m*/*z* [M – H]^−^ calcd for C_12_H_10_N_3_O_6_, 292.0575; found, 292.0573.

#### 4-Amino-2-chloro-*N*-(2,6-dioxo-3-piperidyl)benzamide (**11a**)

Compound **10a** (0.52 g, 1.69 mmol) and 1,2-dichlorobenzene (3.80 mL, 4.97 g, 33.8 mmol) were dissolved in dry DMF (15 mL) under an argon atmosphere. Pd/C (0.10 g, 20% by mass) was added and the mixture was stirred under a hydrogen atmosphere for 18 h at rt. The suspension was filtered through Celite and washed with MeOH (50 mL), and the volatiles were evaporated. The crude product was purified by CC (CH_2_Cl_2_/MeOH/NH_4_OH 9:1:0.1) to obtain a colorless solid. Yield 50 mg (11%); mp 182–186 °C; *R*_f_ = 0.22 (CH_2_Cl_2_/MeOH/NH_4_OH 9:1:0.1); ^1^H NMR (400 MHz, DMSO-*d*_6_) δ 1.93–2.10 (m, 2H), 2.55 (s, 1H), 2.69–2.83 (m, 1H), 4.62–4.73 (m, 1H), 5.76 (s, 2H), 6.50 (dd, *J* = 8.4, 2.2 Hz, 1H), 6.59 (d, *J* = 2.2 Hz, 1H), 7.24 (d, *J* = 8.4 Hz, 1H), 8.28 (d, *J* = 8.3 Hz, 1H), 10.82 (s, 1H); ^13^C NMR (101 MHz, DMSO-*d*_6_) δ 24.26, 30.82, 49.45, 111.59, 113.84, 121.71, 130.76, 131.50, 151.47, 166.25, 172.19, 173.09; UPLC-retention time, 1.81 min; purity 97%. HRMS (ESI) *m*/*z* [M + H]^+^ calcd for C_12_H_13_N_3_O_3_Cl, 282.0640; found, 282.0636.

#### 4-Amino-*N*-(2,6-dioxo-3-piperidyl)-2-(trifluoromethyl)benzamide (**11b**)

This compound was prepared using the General Procedure C and compound **10b** (0.35 g). The crude product was purified by FC (25 g, 30 μm, gradient from 50 to 100% EtOAc in cyclohexane) to give a colorless solid. Yield 0.23 g (74%); mp 216–218 °C; *R*_f_ = 0.39 (EtOAc); ^1^H NMR (500 MHz, DMSO-*d*_6_) δ 1.89–2.07 (m, 2H), 2.49–2.56 (m, 1H), 2.70–2.81 (m, 1H), 4.64 (ddd, *J* = 5.5, 8.4, 12.0 Hz, 1H), 5.82 (s, 2H), 6.76 (dd, *J* = 2.2, 8.3 Hz, 1H), 6.90 (d, *J* = 2.2 Hz, 1H), 7.27 (d, *J* = 8.2 Hz, 1H), 8.40 (d, *J* = 8.4 Hz, 1H), 10.77 (s, 1H); ^13^C NMR (126 MHz, DMSO-*d*_6_) δ 24.23, 30.97, 49.44, 110.84 (d, *J* = 5.3 Hz), 115.46, 122.33, 124.02 (q, *J* = 274.6 Hz), 127.71 (q, *J* = 31.0 Hz), 130.28, 150.38, 167.51, 172.15, 173.11; LC–MS (ESI) (90% H_2_O to 100% MeCN in 10 min, then 100% MeCN to 20 min, DAD 220–600 nm), *t*_R_ = 1.81 min, 98% purity. *m*/*z* [M + H]^+^ calcd for C_13_H_13_F_3_N_3_O_3_, 316.09; found, 316.1. HRMS (ESI) *m*/*z* [M + H]^+^ calcd for C_13_H_13_F_3_N_3_O_3_, 316.0904; found, 316.0895.

#### 4-Amino-*N*-(2,6-dioxo-3-piperidyl)-2-methylbenzamide (**11c**)

This compound was prepared using the General Procedure C and compound **10c** (1.15 g, 3.94 mmol). The crude product was purified by CC (CH_2_Cl_2_/MeOH/NH_4_OH 9:1:0.1) to obtain a colorless solid. Yield 0.21 g (21%); mp 146–150 °C; *R*_f_ = 0.30 (CH_2_Cl_2_/MeOH/NH_4_OH 9:1:0.1); ^1^H NMR (400 MHz, DMSO-*d*_6_) δ 1.89–2.12 (m, 2H), 2.28 (s, 3H), 2.52–2.56 (m, 1H), 2.71–2.82 (m, 1H), 4.62–4.70 (m, 1H), 5.38 (s, 2H), 6.34–6.39 (m, 2H), 7.16–7.21 (m, 1H), 8.08 (d, *J* = 8.4 Hz, 1H), 10.79 (s, 1H); ^13^C NMR (101 MHz, DMSO-*d*_6_) δ 20.43, 24.36, 31.02, 49.23, 110.21, 115.53, 122.72, 129.15, 137.82, 150.23, 168.85, 172.54, 173.12; UPLC-retention time, 0.92 min; purity 95%. HRMS (ESI) *m*/*z* [M + H]^+^ calcd for C_13_H_16_N_3_O_3_, 262.1186; found, 262.1182.

#### 4-Amino-*N*-(2,6-dioxo-3-piperidyl)-2-methoxybenzamide (**11d**)

This compound was prepared using the General Procedure C and compound **10d** (0.61 g, 3.94 mmol). The crude product was purified by CC (CH_2_Cl_2_/MeOH/NH_4_OH 9:1:0.1) to obtain a colorless solid. Yield 80 mg (14%); mp 174–179 °C; *R*_f_ = 0.35 (CH_2_Cl_2_/MeOH/NH_4_OH 9:1:0.1); ^1^H NMR (400 MHz, DMSO-*d*_6_) δ 1.97–2.17 (m, 2H), 2.52–2.53 (m, 1H), 2.69–2.81 (m, 1H), 3.83 (s, 3H), 4.64–4.73 (m, 1H), 5.80 (s, 2H), 6.20 (dd, *J* = 8.5, 2.0 Hz, 1H), 6.24 (d, *J* = 2.0 Hz, 1H), 7.65 (d, *J* = 8.5 Hz, 1H), 8.34 (d, *J* = 6.9 Hz, 1H), 10.86 (s, 1H); ^13^C NMR (101 MHz, DMSO-*d*_6_) δ 24.53, 31.10, 50.06, 55.43, 95.90, 106.18, 108.08, 132.76, 153.68, 159.24, 164.62, 172.86, 173.02; UPLC-retention time, 2.28 min; purity 96%. HRMS (ESI) *m*/*z* [M + H]^+^ calcd for C_13_H_16_N_3_O_4_, 278.1135; found, 278.1129.

#### 4-Amino-*N*-(2,6-dioxo-3-piperidyl)-2-hydroxybenzamide (**11e**)

This compound was prepared using the General Procedure C and compound **10e** (0.42 g, 3.94 mmol). The crude product was purified by CC (CH_2_Cl_2_/MeOH/NH_4_OH 9:1:0.1) to obtain a colorless solid. Yield 0.23 g (44%); mp 199–202 °C; *R*_f_ = 0.26 (CH_2_Cl_2_/MeOH/NH_4_OH 9:1:0.1); ^1^H NMR (400 MHz, DMSO-*d*_6_) δ 1.92–2.01 (m, 1H), 2.02–2.17 (m, 1H), 2.53–2.57 (m, 1H), 2.72–2.84 (m, 1H), 4.69–4.79 (m, 1H), 5.80 (s, 2H), 5.96 (d, *J* = 2.2 Hz, 1H), 6.07 (dd, *J* = 8.7, 2.2 Hz, 1H), 7.50 (d, *J* = 8.7 Hz, 1H), 8.55 (d, *J* = 8.1 Hz, 1H), 10.87 (s, 1H), 12.52 (s, 1H); ^13^C NMR (101 MHz, DMSO-*d*_6_) δ 24.19, 31.03, 49.11, 100.19, 106.15, 128.93, 162.25, 169.32, 172.32, 173.02; UPLC-retention time, 1.97 min; purity 97%. HRMS (ESI) *m*/*z* [M + H]^+^ calcd for C_12_H_14_N_3_O_4_, 264.0979; found, 264.0973.

#### 4-Amino-*N*-(2,6-dioxo-3-piperidyl)-2,5-difluoro-3-methoxybenzamide (**11f**)

Compound **14** (0.46 g, 1.0 mmol) was dissolved in dry CH_2_Cl_2_, treated with TFA (0.23 mL, 3.0 mmol), and stirred at rt for 4 h. The brown suspension was filtrated, and the filtrate was subjected to FC (50 g, 30 μm, gradient from 30 to 100% EtOAc in cyclohexane) to give a slightly yellow solid. Yield 0.24 g (77%); mp 182–184 °C; *R*_f_ = 0.60 (80% EtOAc in cyclohexane); ^1^H NMR (500 MHz, DMSO-*d*_6_) δ 1.95–2.04 (m, 1H), 2.04–2.15 (m, 1H), 2.50–2.55 (m, 1H), 2.70–2.81 (m, 1H), 3.79 (s, 3H), 4.70 (ddd, *J* = 5.4, 7.9, 12.8 Hz, 1H), 5.83 (s, 2H), 7.16 (dd, *J* = 6.2, 11.6 Hz, 1H), 8.09 (dd, *J* = 5.5, 7.9 Hz, 1H), 10.75 (s, 1H); ^13^C NMR (126 MHz, DMSO-*d*_6_) δ 24.21, 31.08, 49.93, 60.96 (d, *J* = 3.7 Hz), 107.50 (dd, *J* = 7.2, 13.2 Hz), 110.46 (dd, *J* = 4.0, 21.8 Hz), 134.26 (dd, *J* = 7.4, 16.5 Hz), 135.02 (dd, *J* = 5.2, 15.4 Hz), 145.95 (d, *J* = 234.9 Hz), 151.27 (d, *J* = 244.6 Hz), 162.54, 172.23, 173.05; LC–MS (ESI) (90% H_2_O to 100% MeCN in 10 min, then 100% MeCN to 20 min, DAD 200–600 nm), *t*_R_ = 2.62 min, 99% purity. *m*/*z* [M + H]^+^ calcd for C_13_H_14_F_2_N_3_O_4_, 314.09; found, 314.1. HRMS (ESI) *m*/*z* [M + H]^+^ calcd for C_13_H_14_F_2_N_3_O_4_, 314.0947; found, 314.0948.

#### *N*-(2,6-Dioxo-3-piperidyl)-2,4,5-trifluoro-3-methoxybenzamide (**13**)

This compound was prepared using the General Procedure A (4 mmol scale) and 2,4,5-trifluoro-3-methoxybenzoyl chloride (0.90 g). The crude product was purified by FC (40 g, 30 μm, gradient from 50 to 80% EtOAc in cyclohexane) to give a beige solid. Yield 0.53 g (42%); mp 174–176 °C; *R*_f_ = 0.69 (EtOAc); ^1^H NMR (500 MHz, DMSO-*d*_6_) δ 1.96–2.14 (m, 2H), 2.48–2.57 (m, 1H), 2.72–2.83 (m, 1H), 4.01 (s, 3H), 4.74 (ddd, *J* = 5.5, 8.2, 13.0 Hz, 1H), 7.35–7.44 (m, 1H), 8.67–8.72 (m, 1H), 10.85 (s, 1H); ^13^C NMR (126 MHz, DMSO-*d*_6_) δ 24.06, 30.97, 49.94, 62.41 (t, *J* = 3.3 Hz), 110.43 (dd, *J* = 3.4, 20.5 Hz), 119.79 (d, *J* = 14.4 Hz), 137.64 (d, *J* = 14.5 Hz), 144.81 (dd, *J* = 10.5, 175.9 Hz), 147.45 (d, *J* = 9.1 Hz), 149.28 (d, *J* = 249.6 Hz), 161.77, 171.69, 172.98; LC–MS (ESI) (90% H_2_O to 100% MeCN in 10 min, then 100% MeCN to 20 min, DAD 200–600 nm), *t*_R_ = 4.17 min, 99% purity. *m*/*z* [M + H]^+^ calcd for C_13_H_12_F_3_N_2_O_4_, 317.07; found, 317.1. HRMS (ESI) *m*/*z* [M + H]^+^ calcd for C_13_H_12_F_3_N_2_O_4_, 317.0744; found, 317.0732.

#### 4-[(2,4-Dimethoxyphenyl)methylamino]-*N*-(2,6-dioxo-3-piperidyl)-2,5-difluoro-3-methoxybenzamide (**14**)

Compound **13** (0.88 g, 2.8 mmol) was dissolved in dry DMSO (28 mL). 2,4-Dimethoxybenzylamine (0.46 g, 2.8 mmol) and DIPEA (0.97 mL, 5.6 mmol) were added, and the mixture was stirred at 90 °C for 18 h. After cooling, it was diluted with EtOAc (100 mL) and washed with half-saturated NH_4_Cl solution (100 mL). The aqueous layer was extracted EtOAc (100 mL) again, and the combined organic layers were washed with 5% LiCl solution and brine (each 100 mL), dried over Na_2_SO_4_, filtered, and concentrated *in vacuo*. The crude product was purified by FC (80 g, 30 μm, gradient from 50 to 80% EtOAc in cyclohexane) to give a colorless solid. Yield 0.45 g (35%); mp 102–104 °C; *R*_f_ = 0.55 (80% EtOAc in cyclohexane); ^1^H NMR (600 MHz, DMSO-*d*_6_) δ 1.93–2.01 (m, 1H), 2.02–2.12 (m, 1H), 2.70–2.79 (m, 1H), 3.72 (d, *J* = 17.3 Hz, 6H), 3.78 (s, 3H), 4.41 (dd, *J* = 1.7, 7.0 Hz, 2H), 4.69 (ddd, *J* = 5.4, 8.0, 12.8 Hz, 1H), 5.74–5.79 (m, 1H), 6.42 (dd, *J* = 2.4, 8.3 Hz, 1H), 6.53 (d, *J* = 2.3 Hz, 1H), 7.03 (d, *J* = 8.3 Hz, 1H), 7.11 (dd, *J* = 6.3, 13.3 Hz, 1H), 8.18 (dd, *J* = 4.5, 8.1 Hz, 1H), 10.81 (s, 1H); ^13^C NMR (151 MHz, DMSO-*d*_6_) δ 24.18, 31.07, 49.89, 55.27, 55.54, 61.43, 98.53, 104.33, 109.91 (dd, *J* = 7.8, 13.7 Hz), 111.49 (dd, *J* = 4.1, 24.2 Hz), 120.21, 128.48, 134.48 (dd, *J* = 4.2, 11.2 Hz), 136.29 (dd, *J* = 8.1, 16.1 Hz), 146.90 (d, *J* = 236.6 Hz), 150.82 (d, *J* = 245.8 Hz), 157.84, 159.85, 162.33, 172.15, 173.08; LC–MS (ESI) (90% H_2_O to 100% MeCN in 10 min, then 100% MeCN to 20 min, DAD 220–600 nm), *t*_R_ = 6.15 min, 99% purity. *m*/*z* [M + H]^+^ calcd for C_22_H_24_F_2_N_3_O_6_, 464.16; found, 464.3. HRMS (ESI) *m*/*z* [M + H]^+^ calcd for C_22_H_24_F_2_N_3_O_6_, 464.1628; found, 464.1628.

#### 4-(Butylamino)-*N*-(2,6-dioxo-3-piperidyl)-2-fluorobenzamide (**15**)

A solution of **8d** (0.27 g, 1.0 mmol) in dry DMF (20 mL) was added to a mixture of butyraldehyde (0.14 g, 2.0 mmol) in dry DMF (2 mL) and AcOH (1 mL) under an argon atmosphere. After stirring the solution for 1 h at rt, the mixture was cooled to 0 °C, and STAB (1.27 g, 6.0 mmol) was added portionwise. The mixture was stirred at rt for 18 h, after which MeOH (10 mL) was added, and the volatiles were then evaporated. The crude product was purified by FC (40 g, 30 μm, gradient from 30 to 100% EtOAc in cyclohexane) to give a blueish solid. Yield 0.22 g (68%); mp 150–152 °C; *R*_f_ = 0.53 (80% EtOAc in cyclohexane); ^1^H NMR (500 MHz, DMSO-*d*_6_) δ 0.90 (t, *J* = 7.4 Hz, 3H), 1.31–1.42 (m, 2H), 1.47–1.56 (m, 2H), 1.95–2.15 (m, 2H), 2.46–2.54 (m, 1H), 2.69–2.80 (m, 1H), 3.04 (td, *J* = 5.4, 7.0 Hz, 2H), 4.65–4.74 (m, 1H), 6.31 (dd, *J* = 2.2, 15.1 Hz, 1H), 6.44 (dd, *J* = 2.2, 8.6 Hz, 1H), 6.46–6.52 (m, 1H), 7.54 (t, *J* = 8.9 Hz, 1H), 7.81 (t, *J* = 7.6 Hz, 1H), 10.79 (s, 1H); ^13^C NMR (126 MHz, DMSO-*d*_6_) δ 13.86, 19.84, 24.36, 30.67, 31.14, 42.26, 49.89, 97.17 (d, *J* = 27.5 Hz), 107.54 (d, *J* = 12.7 Hz), 108.22, 131.92 (d, *J* = 4.8 Hz), 153.64 (d, *J* = 12.4 Hz), 162.01 (d, *J* = 245.8 Hz), 163.36 (d, *J* = 2.9 Hz), 172.52, 173.08; LC–MS (ESI) (90% H_2_O to 100% MeCN in 10 min, then 100% MeCN to 20 min, DAD 220–600 nm), *t*_R_ = 5.37 min, 99% purity. *m*/*z* [M + H]^+^ calcd for C_16_H_21_FN_3_O_3_, 322.16; found, 322.3. HRMS (ESI) *m*/*z* [M + H]^+^ calcd for C_16_H_21_FN_3_O_3_, 322.1562; found, 322.1563.

#### 4-[[2-(Butylamino)-2-oxo-ethyl]amino]-*N*-(2,6-dioxo-3-piperidyl)-2-fluorobenzamide

Compound **24** (0.15 g, 0.4 mmol) was dissolved in dry DMF (8 mL), and DIPEA (0.35 mL, 2.0 mmol), *n*-butylamine (40 μL, 0.4 mmol), and HATU (0.17 g, 0.44 mmol) were added. After stirring this mixture for 16 h at rt, it was diluted with EtOAc (50 mL), washed with half-saturated NH_4_Cl solution (50 mL), and extracted again with EtOAc (50 mL). The combined organic layers were washed with 5% LiCl and brine (each 50 mL), dried over Na_2_SO_4_, filtered, and concentrated *in vacuo*. The crude product was purified by FC (25 g, 15 μm, gradient from 60 to 100% EtOAc in cyclohexane) to give a colorless solid. Yield 39 mg (25%); mp 208–210 °C; *R*_f_ = 0.31 (EtOAc); ^1^H NMR (500 MHz, DMSO-*d*_6_) δ 0.85 (t, *J* = 7.3 Hz, 3H), 1.13–1.30 (m, 2H), 1.32–1.42 (m, 2H), 1.96–2.15 (m, 2H), 2.49–2.55 (m, 1H), 2.69–2.80 (m, 1H), 3.07 (q, *J* = 6.8 Hz, 2H), 3.69 (d, *J* = 5.9 Hz, 2H), 4.70 (ddd, *J* = 5.8, 7.8, 12.6 Hz, 1H), 6.30 (dd, *J* = 2.2, 14.8 Hz, 1H), 6.44 (dd, *J* = 2.2, 8.6 Hz, 1H), 6.77 (t, *J* = 5.8 Hz, 1H), 7.54 (t, *J* = 8.8 Hz, 1H), 7.84–7.96 (m, 2H), 10.79 (s, 1H); ^13^C NMR (126 MHz, DMSO-*d*_6_) δ 13.74, 19.59, 24.33, 31.12, 31.31, 38.27, 46.20, 49.88, 97.99 (d, *J* = 27.4 Hz), 108.69 (d, *J* = 15.7 Hz), 131.77, 153.13 (d, *J* = 12.2 Hz), 161.70 (d, *J* = 246.1 Hz), 163.31 (d, *J* = 2.7 Hz), 169.02, 172.45, 173.07; LC–MS (ESI) (90% H_2_O to 100% MeCN in 10 min, then 100% MeCN to 20 min, DAD 220–600 nm), *t*_R_ = 3.72 min, 99% purity. *m*/*z* [M + H]^+^ calcd for C_18_H_24_FN_4_O_4_, 379.18; found, 379.2. HRMS (ESI) *m*/*z* [M + H]^+^ calcd for C_18_H_24_FN_4_O_4_, 379.1776; found, 379.1771.

#### 4-Butoxy-*N*-(2,6-dioxo-3-piperidyl)-2-fluorobenzamide (**17**)

This compound was prepared using the General Procedure B (2 mmol scale) and precursor **26** (0.42 g). The crude product was purified by FC (40 g, 30 μm, gradient from 10 to 50% EtOAc in cyclohexane) to give a gray solid. Yield 0.28 g (43%); mp 156–158 °C; *R*_f_ = 0.32 (50% EtOAc in cyclohexane); ^1^H NMR (600 MHz, DMSO-*d*_6_) δ 0.92 (t, *J* = 7.4 Hz, 3H), 1.37–1.47 (m, 2H), 1.65–1.73 (m, 2H), 1.96–2.04 (m, 1H), 2.05–2.15 (m, 1H), 2.48–2.55 (m, 1H), 2.72–2.81 (m, 1H), 4.03 (t, *J* = 6.5 Hz, 2H), 4.73 (ddd, *J* = 5.3, 8.0, 12.8 Hz, 1H), 6.86 (dd, *J* = 2.4, 8.7 Hz, 1H), 6.89 (dd, *J* = 2.4, 13.2 Hz, 1H), 7.67 (t, *J* = 8.8 Hz, 1H), 8.26 (dd, *J* = 4.7, 8.0 Hz, 1H), 10.82 (s, 1H); ^13^C NMR (151 MHz, DMSO-*d*_6_) δ 13.79, 18.77, 24.21, 30.62, 31.09, 49.86, 68.20, 102.31 (d, *J* = 26.5 Hz), 111.20, 114.63 (d, *J* = 13.2 Hz), 131.78 (d, *J* = 4.4 Hz), 160.90 (d, *J* = 249.0 Hz), 162.39 (d, *J* = 11.1 Hz), 163.14, 172.19, 173.10; LC–MS (ESI) (90% H_2_O to 100% MeCN in 10 min, then 100% MeCN to 20 min, DAD 220–600 nm), *t*_R_ = 5.94 min, 99% purity. *m*/*z* [M + H]^+^ calcd for C_16_H_20_FN_2_O_4_, 323.14; found, 323.2. HRMS (ESI) *m*/*z* [M + H]^+^ calcd for C_16_H_20_FN_2_O_4_, 323.1402; found, 323.1402.

#### 4-(Butylamino)-*N*-(2,6-dioxo-3-piperidyl)-2-methoxybenzamide (**18**)

To a mixture of butyraldehyde (83 mg, 1.15 mmol), dry DMF (1 mL), and AcOH (0.5 mL) was added under an argon atmosphere a solution of **11d** (0.18 g, 0.57 mmol) in dry DMF (10 mL). After stirring the solution for 1 h at rt, the mixture was cooled to 0 °C and STAB (0.73 g, 3.44 mmol) was added. The mixture was stirred at rt for 18 h, after which MeOH (10 mL) was added, and the volatiles were then evaporated. The crude product was purified by CC (CH_2_Cl_2_/MeOH 20:1) to obtain an off-white solid. Yield 89 mg (47%); mp 251–253 °C; *R*_f_ = 0.28 (CH_2_Cl_2_/MeOH 20:1); ^1^H NMR (400 MHz, DMSO-*d*_6_) δ 1.01 (t, *J* = 7.4 Hz, 3H), 1.27 (t, *J* = 7.5 Hz, 2H), 1.75–1.86 (m, 2H), 2.09–2.18 (m, 2H), 2.73–2.85 (m, 3H), 2.87–2.93 (m, 2H), 4.02 (s, 3H), 4.78–4.87 (m, 1H), 7.44 (s, 1H), 8.12 (s, 1H), 8.34 (s, 1H), 8.75 (d, *J* = 7.6 Hz, 1H), 10.92 (s, 1H); ^13^C NMR (101 MHz, DMSO-*d*_6_) δ 14.17, 21.54, 24.15, 24.24, 30.96, 36.80, 50.02, 56.16, 107.46, 123.39, 130.72, 134.28, 147.98, 156.67, 164.39, 172.24, 173.00; UPLC-retention time, 2.93 min; purity 99%. HRMS (ESI) *m*/*z* [M + H]^+^ calcd for C_17_H_24_N_3_O_4_, 334.1761; found, 334.1757.

#### 4-(Butylamino)-*N*-(2,6-dioxo-3-piperidyl)-2,5-difluoro-3-methoxybenzamide (**19**)

Compound **13** (0.32 g, 1.0 mmol) was dissolved in dry DMSO (10 mL). *N*-Butylamine (99 μL, 2.8 mmol) and DIPEA (0.35 mL, 2.0 mmol) were added, and the mixture was stirred at 90 °C for 18 h. After cooling, it was diluted with EtOAc (50 mL) and washed with half-saturated NH_4_Cl solution (50 mL). The aqueous layer was extracted EtOAc (50 mL) again, and the combined organic layers were washed with 5% LiCl solution and brine (each 50 mL), dried over Na_2_SO_4_, filtered, and concentrated *in vacuo*. The crude product was purified by FC (40 g, 30 μm, gradient from 30 to 80% EtOAc in cyclohexane) to give a colorless solid. Yield 0.15 g (40%); mp 146–148 °C; *R*_f_ = 0.43 (60% EtOAc in cyclohexane); ^1^H NMR (500 MHz, DMSO-*d*_6_) δ 0.87 (t, *J* = 7.4 Hz, 3H), 1.30 (h, *J* = 7.3 Hz, 2H), 1.43–1.53 (m, 2H), 1.95–2.04 (m, 1H), 2.04–2.15 (m, 1H), 2.50–2.59 (m, 1H), 2.70–2.81 (m, 1H), 3.32 (dd, *J* = 5.9, 7.5 Hz, 2H), 3.78 (s, 3H), 4.71 (ddd, *J* = 5.4, 7.9, 12.7 Hz, 1H), 5.62–5.68 (m, 1H), 7.16 (dd, *J* = 8.2, 11.9 Hz, 1H), 8.13 (dd, *J* = 4.1, 8.5 Hz, 1H), 10.81 (s, 1H); ^13^C NMR (126 MHz, DMSO-*d*_6_) δ 13.85, 19.49, 24.20, 31.07, 32.65, 44.07, 49.92, 61.34, 108.72 (dd, *J* = 8.4, 12.8 Hz), 110.75–112.18 (m), 134.51–135.15 (m), 135.75 (dd, *J* = 9.2, 15.6 Hz), 146.45 (d, *J* = 235.9 Hz), 151.08 (d, *J* = 245.0 Hz), 162.34, 172.18, 173.04; LC–MS (ESI) (90% H_2_O to 100% MeCN in 10 min, then 100% MeCN to 20 min, DAD 220–600 nm), *t*_R_ = 6.01 min, 99% purity. *m*/*z* [M + H]^+^ calcd for C_17_H_22_F_2_N_3_O_4_, 370.16; found, 370.1. HRMS (ESI) *m*/*z* [M + H]^+^ calcd for C_17_H_22_F_2_N_3_O_4_, 370.1573; found, 370.1574.

#### 3-[7-(Butylamino)-4-oxo-1,2,3-benzotriazin-3-yl]piperidine-2,6-dione (**20**)

To a mixture of butyraldehyde (0.15 g, 2.0 mmol), dry DMF (2 mL), and AcOH (1.6 mL) was added under an argon atmosphere a solution of compound **32** (0.28 g, 1.0 mmol) in dry DMF (10 mL). After stirring the solution for 1 h at rt, the mixture was cooled to 0 °C and STAB (1.28 g, 6.0 mmol) was added. The mixture was stirred for 18 h at rt, after which MeOH (10 mL) was added, and the volatiles were then evaporated. The crude product was purified by CC (CH_2_Cl_2_/MeOH 30:1) to obtain an off-white solid. Yield 85 mg (26%); mp 201–205 °C; *R*_f_ = 0.16 (CH_2_Cl_2_/MeOH 30:1); ^1^H NMR (400 MHz, DMSO-*d*_6_) δ 0.93 (t, *J* = 7.3 Hz, 3H), 1.36–1.47 (m, 2H), 1.54–1.64 (m, 2H), 2.17–2.26 (m, 1H), 2.59–2.72 (m, 2H), 2.85–3.00 (m, 1H), 3.14–3.23 (m, 2H), 5.87 (dd, *J* = 12.3, 5.4 Hz, 1H), 7.00 (d, *J* = 2.3 Hz, 1H), 7.14 (dd, *J* = 8.8, 2.4 Hz, 1H), 7.18 (t, *J* = 5.5 Hz, 1H), 7.89 (d, *J* = 8.7 Hz, 1H), 11.14 (s, 1H); ^13^C NMR (101 MHz, DMSO-*d*_6_) δ 13.75, 19.75, 22.89, 30.25, 30.78, 42.06, 57.98, 107.07, 125.65, 146.18, 154.16, 154.63, 170.03, 172.74; UPLC-retention time, 5.42 min; purity 99%. HRMS (ESI) *m*/*z* [M + H]^+^ calcd for C_16_H_20_N_5_O_3_, 330.1561; found, 330.1552.

#### 3-(4-Butoxyphenyl)piperidine-2,6-dione (**21**)^16^

Compound **35** (0.44 g, 1.0 mmol) was dissolved in dry THF (8 mL) under an argon atmosphere. Pd/C (89 mg, 20% by mass) was added, and the mixture was stirred under a H_2_ atmosphere for 18 h at rt. The suspension was filtered through Celite, washed with MeOH (20 mL) and the volatiles were evaporated. The crude product was purified by CC (*n*-hexanes/EtOAc 2:1) to obtain a colorless solid. Yield 0.12 g (26%); mp 146–148 °C; *R*_f_ = 0.40 (*n*-hexanes/EtOAc 2:1); ^1^H NMR (400 MHz, CDCl_3_) δ 0.97 (t, *J* = 7.4 Hz, 3H), 1.43–1.54 (m, 2H), 1.72–1.81 (m, 2H), 2.14–2.32 (m, 2H), 2.59–2.77 (m, 2H), 3.73 (dd, *J* = 9.5, 5.3 Hz, 1H), 3.95 (t, *J* = 6.5 Hz, 2H), 6.86–6.92 (m, 2H), 7.08–7.14 (m, 2H), 8.01 (s, 1H); ^13^C NMR (101 MHz, CDCl_3_) δ 13.97, 19.37, 26.55, 31.03, 31.41, 47.30, 67.86, 115.05, 128.79, 129.17, 158.82, 172.46, 173.54; UPLC-retention time, 5.84 min; purity 99%. HRMS (ESI) *m*/*z* [M + H]^+^ calcd for C_15_H_20_NO_3_, 262.1438; found, 262.1434.

#### 5-(Butylamino)-2-(2,6-dioxo-3-piperidyl)isoindoline-1,3-dione (**22**)

Compound **27** (0.14 g, 0.5 mmol) was dissolved in dry DMSO (5 mL). *N*-Butylamine (37 mg, 0.5 mmol) and DIPEA (0.17 mL, 1.0 mmol) were added, and the mixture was stirred at 90 °C for 18 h. After cooling, it was diluted with EtOAc (50 mL) and washed with half-saturated NH_4_Cl solution (50 mL). The aqueous layer was extracted EtOAc (50 mL) again, and the combined organic layers were washed with 5% LiCl solution and brine (each 50 mL), dried over Na_2_SO_4_, filtered, and concentrated *in vacuo*. The crude product was purified by FC (25 g, 15 μm, gradient from 20 to 60% EtOAc in cyclohexane) to give a yellow solid. Yield 71 mg (43%); mp 124–126 °C; *R*_f_ = 0.45 (50% EtOAc/petroleum ether); ^1^H NMR (500 MHz, DMSO-*d*_6_) δ 0.91 (t, *J* = 7.3 Hz, 3H), 1.33–1.44 (m, 2H), 1.50–1.60 (m, 2H), 1.94–2.03 (m, 1H), 2.45–2.61 (m, 2H), 2.80–2.92 (m, 1H), 3.11–3.18 (m, 2H), 5.01 (dd, *J* = 5.4, 12.7 Hz, 1H), 6.83 (dd, *J* = 2.2, 8.3 Hz, 1H), 6.93 (d, *J* = 2.1 Hz, 1H), 7.05 (t, *J* = 5.4 Hz, 1H), 7.54 (d, *J* = 8.4 Hz, 1H), 11.01 (s, 1H); ^13^C NMR (126 MHz, DMSO-*d*_6_) δ 13.82, 19.82, 22.38, 30.49, 31.12, 42.31, 48.77, 115.95, 125.21, 134.35, 154.63, 167.28, 167.83, 170.27, 172.90; LC–MS (ESI) (90% H_2_O to 100% MeCN in 10 min, then 100% MeCN to 20 min, DAD 220–600 nm), *t*_R_ = 5.77 min, 98% purity. *m*/*z* [M + H]^+^ calcd for C_17_H_20_N_3_O_4_, 330.14; found, 330.2. HRMS (ESI) *m*/*z* [M + H]^+^ calcd for C_17_H_20_N_3_O_4_, 330.1448; found, 330.1444.

#### 3-[5-(Butylamino)-1-oxo-isoindolin-2-yl]piperidine-2,6-dione (**23**)

To a mixture of butyraldehyde (28 mg, 0.39 mmol), dry DMF (1 mL) and AcOH (0.3 mL) was added under an argon atmosphere a solution of compound **28** (50 mg, 0.19 mmol) in dry DMF (3 mL). After stirring the solution for 1 h at rt, the mixture was cooled to 0 °C and STAB (0.25 g, 1.16 mmol) was added. The mixture was stirred for 18 h at rt, after which MeOH (10 mL) was added, and the volatiles were then evaporated. The crude product was purified by CC (CH_2_Cl_2_/MeOH 20:1) to obtain an off-white solid. Yield 41 mg (67%); mp 135–138 °C; *R*_f_ = 0.18 (CH_2_Cl_2_/MeOH 20:1); ^1^H NMR (400 MHz, DMSO-*d*_6_) δ 0.91 (t, *J* = 7.3 Hz, 3H), 1.33–1.44 (m, 2H), 1.50–1.59 (m, 2H), 1.89–1.99 (m, 1H), 2.25–2.40 (m, 1H), 2.54–2.61 (m, 1H), 2.89 (ddd, *J* = 17.2, 13.7, 5.4 Hz, 1H), 3.01–3.10 (m, 2H), 4.10–4.29 (m, 2H), 5.01 (dd, *J* = 13.3, 5.1 Hz, 1H), 6.35 (t, *J* = 5.4 Hz, 1H), 6.61 (d, *J* = 1.9 Hz, 1H), 6.65 (dd, *J* = 8.4, 2.0 Hz, 1H), 7.37 (d, *J* = 8.3 Hz, 1H), 10.93 (s, 1H); ^13^C NMR (101 MHz, DMSO-*d*_6_) δ 13.79, 19.80, 22.66, 30.57, 31.30, 42.27, 46.76, 51.27, 104.06, 112.43, 118.59, 123.89, 144.52, 152.37, 168.76, 171.46, 172.98; UPLC-retention time, 4.56 min; purity 97%. HRMS (ESI) *m*/*z* [M + H]^+^ calcd for C_17_H_22_N_3_O_3_, 316.1656; found, 316.1650.

#### 2-[4-[(2,6-Dioxo-3-piperidyl)carbamoyl]-3-fluoroanilino]acetic Acid (**24**)

Compound **8d** (0.53 g, 2.0 mmol) was dissolved in dry DMF (10 mL) and cooled to 0 °C. AcOH (0.46 mL, 8.0 mmol, NaOAc (0.33 g, 4.0 mmol), glycolic acid monohydrate (0.28 g, 3.0 mmol), and NaCNBH_3_ (0.14 g, 2.2 mmol) were added, and the mixture was stirred for 2 h at rt. The dark mixture was filtered through a Celite pad, and washed with EtOAc + 1% AcOH (200 mL). The organic layer was washed with 5% LiCl solution and brine (each 100 mL), dried over Na_2_SO_4_, filtered, and concentrated *in vacuo*. The crude product was purified by FC (80 g, 30 μm, gradient from 30 to 80% EtOAc in cyclohexane, each containing 1% AcOH) to give a colorless solid. Yield 0.13 g (20%); mp 226–230 °C; *R*_f_ = 0.26 (EtOAc + 1% AcOH); ^1^H NMR (600 MHz, DMSO-*d*_6_) δ 1.96–2.04 (m, 1H), 2.04–2.14 (m, 1H), 2.49–2.54 (m, 1H), 2.69–2.79 (m, 1H), 3.87 (d, *J* = 4.6 Hz, 2H), 4.70 (ddd, *J* = 5.4, 7.8, 12.6 Hz, 1H), 6.36 (dd, *J* = 2.2, 14.7 Hz, 1H), 6.47 (dd, *J* = 2.2, 8.6 Hz, 1H), 6.74 (t, *J* = 6.1 Hz, 1H), 7.53 (t, *J* = 8.8 Hz, 1H), 7.89 (t, *J* = 7.4 Hz, 1H), 10.80 (s, 1H), 12.61 (s, 1H); ^13^C NMR (151 MHz, DMSO-*d*_6_) δ 24.35, 31.15, 44.40, 49.88, 98.05 (d, *J* = 27.6 Hz), 108.55, 108.73 (d, *J* = 12.3 Hz), 131.82 (d, *J* = 4.7 Hz), 153.06 (d, *J* = 12.1 Hz), 160.92, 162.55, 163.36, 171.97, 172.49, 173.13; LC–MS (ESI) (90% H_2_O to 100% MeCN in 10 min, then 100% MeCN to 20 min, DAD 220–600 nm), *t*_R_ = 0.30 min, 98% purity. *m*/*z* [M – H]^−^ calcd for C_14_H_15_FN_3_O_5_, 322.08; found, 322.1, HRMS (ESI) *m*/*z* [M – H]^−^ calcd for C_14_H_15_FN_3_O_5_, 322.0845; found, 322.0847.

#### Methyl 4-Butoxy-2-fluorobenzoate (**25**)

Methyl 2-fluoro-4-hydroxybenzoate (1.70 g, 10 mmol) and *n*-butanol (0.92 mL, 10 mmol) were dissolved in dry THF (100 mL). The mixture was degassed in an ultrasonic bath and purged with argon gas. Subsequently, triphenylphosphine (3.93 g, 15 mmol) and DIAD (2.94 mL, 15 mmol) were added at 0 °C. After stirring the mixture for 18 h at rt, the volatiles were removed. The product was used in the next step without further purification and characterization.

#### 4-Butoxy-2-fluorobenzoic Acid (**26**)

The red residue **25** was dissolved in H_2_O/EtOH (100 mL), and NaOH (0.40 g, 10 mmol) was added. One hour later, it was diluted with EtOAc (100 mL) and acidified with 2 M HCl until pH = 2. The aqueous layer was extracted again with EtOAc (2 × 50 mL), and the combined organic layers were washed with H_2_O and brine (each 50 mL), dried over Na_2_SO_4_, filtered, and concentrated *in vacuo*. The crude product was purified by FC (80 g, 30 μm, gradient from 5 to 20% EtOAc in cyclohexane) to give a colorless solid. Yield 0.64 g (30%); mp 100–102 °C; *R*_f_ = 0.62 (EtOAc); ^1^H NMR (600 MHz, DMSO-*d*_6_) δ 0.92 (t, *J* = 7.4 Hz, 3H), 1.37–1.46 (m, 2H), 1.65–1.73 (m, 2H), 4.04 (t, *J* = 6.5 Hz, 2H), 6.80–6.89 (m, 2H), 7.79 (t, *J* = 8.7 Hz, 1H), 12.81 (s, 1H); ^13^C NMR (151 MHz, DMSO-*d*_6_) δ 13.76, 18.75, 30.57, 68.27, 102.86 (d, *J* = 25.5 Hz), 111.09 (q, *J* = 3.9 Hz), 133.44 (d, *J* = 3.3 Hz), 162.88 (d, *J* = 241.1 Hz), 163.78 (d, *J* = 4.3 Hz), 164.82 (d, *J* = 3.3 Hz); LC–MS (ESI) (90% H_2_O to 100% MeCN in 10 min, then 100% MeCN to 20 min, DAD 220–600 nm), *t*_R_ = 3.12 min, 99% purity. *m*/*z* [M + H]^+^ calcd for C_11_H_14_FO_3_, 213.09; found, 213.1.

#### 5-Fluorothalidomide (**27**)

This compound was synthesized as described previously.^18^

#### 3-(5-Amino-1-oxo-isoindolin-2-yl)piperidine-2,6-dione (**28**)

This compound was obtained from Enamine (Kyiv, Ukraine).

#### 2-Amino-*N*-(2,6-dioxo-3-piperidyl)-4-nitrobenzamide (**30**)

This compound was prepared using the General Procedure B and 2-Amino-4-nitrobenzoic acid (0.91 g). The crude product was purified by CC (CH_2_Cl_2_/MeOH 20:1) to give a green solid. Yield 0.52 g (35%); mp 175–180 °C; *R*_f_ = 0.18 (CH_2_Cl_2_/MeOH 20:1); ^1^H NMR (400 MHz, DMSO-*d*_6_) δ 1.93–2.02 (m, 1H), 2.05–2.18 (m, 1H), 2.55–2.60 (m, 1H), 2.74–2.87 (m, 1H), 4.71–4.82 (m, 1H), 6.86 (s, 2H), 7.32 (dd, *J* = 8.6, 2.4 Hz, 1H), 7.60 (d, *J* = 2.4 Hz, 1H), 7.70 (d, *J* = 8.7 Hz, 1H), 8.83 (d, *J* = 8.3 Hz, 1H), 10.90 (s, 1H); ^13^C NMR (101 MHz, DMSO-*d*_6_) δ 24.00, 31.01, 49.30, 108.36, 110.19, 119.37, 129.76, 149.61, 150.17, 167.30, 172.21, 173.07; UPLC-retention time, 3.72 min; purity 90%. HRMS (ESI) *m*/*z* [M – H]^−^ calcd for C_12_H_11_N_4_O_5_, 291.0735; found, 291.0736.

#### 3-(7-Nitro-4-oxo-1,2,3-benzotriazin-3-yl)piperidine-2,6-dione (**31**)

2.5 mmol) was added. The mixture was stirred for 3.5 h at rt, then H_2_O (30 mL) was added, and the precipitate was collected by filtration, washed with H_2_O (2 × 10 mL), Et_2_O (2 × 10 mL), and dried *in vacuo* to give a pale-yellow solid. Yield 0.77 g (80%); mp 188–192 °C; *R*_f_ = 0.70 (CH_2_Cl_2_/MeOH 9:1); ^1^H NMR (400 MHz, DMSO-*d*_6_) δ 2.27–2.37 (m, 1H), 2.64–2.78 (m, 2H), 2.91–3.05 (m, 1H), 6.01–6.09 (m, 1H), 8.51 (d, *J* = 8.7 Hz, 1H), 8.66 (dd, *J* = 8.7, 2.2 Hz, 1H), 9.00 (d, *J* = 2.1 Hz, 1H), 11.26 (s, 1H); ^13^C NMR (101 MHz, DMSO-*d*_6_) δ 22.63, 30.77, 48.68, 123.35, 123.70, 127.03, 127.49, 143.50, 151.78, 153.75, 169.54, 172.76; UPLC-retention time, 3.80 min; purity 90%. HRMS (ESI) *m*/*z* [M – H]^−^ calcd for C_12_H_8_N_5_O_5_, 302.0531; found, 302.0533.

#### 3-(7-Amino-4-oxo-1,2,3-benzotriazin-3-yl)piperidine-2,6-dione (**32**)

To a solution of compound **31** (0.77 g, 2.5 mmol) in THF/H_2_O 1:1 (20 mL) was added iron (0.77 g). The mixture was stirred for 18 h at rt. The suspension was filtered and washed with EtOAc (50 mL). The organic phase was washed with saturated NaHCO_3_ (100 mL) and brine (100 mL), dried over Na_2_SO_4_, filtered, and concentrated *in vacuo*, and the volatiles were evaporated. The crude product was purified by CC (CH_2_Cl_2_/MeOH 20:1) to obtain an off-white solid. Yield 0.30 g (44%); mp 262–265 °C; *R*_f_ = 0.35 (CH_2_Cl_2_/MeOH 20:1); ^1^H NMR (400 MHz, DMSO-*d*_6_) δ 2.16–2.26 (m, 1H), 2.58–2.71 (m, 2H), 2.87–3.00 (m, 1H), 5.81–5.90 (m, 1H), 6.65 (s, 2H), 7.06 (d, *J* = 2.1 Hz, 1H), 7.09 (dd, *J* = 8.6, 2.2 Hz, 1H), 7.89 (d, *J* = 8.6 Hz, 1H), 11.13 (s, 1H); ^13^C NMR (101 MHz, DMSO-*d*_6_) δ 22.89, 30.79, 57.97, 106.89, 107.27, 120.24, 126.17, 145.94, 154.19, 155.49, 170.06, 172.78; UPLC-retention time, 2.53 min; purity 93%. HRMS (ESI) *m*/*z* [M – H]^−^ calcd for C_12_H_10_N_5_O_3_, 272.0789; found, 272.0790.

#### 4-(2,6-Dibenzyloxy-3-pyridyl)phenol (**34**)^16^

2,6-Bis(benzyloxy)-3-bromopyridine (0.21 g, 0.57 mmol), (4-hydroxyphenyl)boronic acid (0.16 g, 1.13 mmol) and K_3_PO_4_ (0.27 g, 1.25 mmol) were dissolved in dioxane (10 mL) and H_2_O (1 mL). The reaction mixture was then flushed with argon and PdCl_2_(dppf) × CH_2_Cl_2_ (46 mg, 56.7 μmol) was added. After stirring for 18 h at 110 °C, the mixture was cooled and filtered through Celite, washed with EtOAc (50 mL) and the volatiles were evaporated. The crude product was purified by CC

(*n*-hexanes/EtOAc 4:1) to obtain a white solid. Yield 68 mg (31%); mp 134–137 °C; *R*_*f*_ = 0.30 (*n*-hexanes/EtOAc 4:1); ^1^H NMR (400 MHz, CDCl_3_) δ 4.73 (s, 1H), 5.36 (s, 2H), 5.42 (s, 2H), 6.46 (d, *J* = 8.1 Hz, 1H), 6.83–6.88 (m, 2H), 7.27–7.40 (m, 8H), 7.41–7.47 (m, 4H), 7.56 (d, *J* = 8.1 Hz, 1H); ^13^C NMR (101 MHz, CDCl_3_) δ 67.71, 67.98, 102.49, 115.19, 115.77, 127.35, 127.56, 127.92, 128.48, 128.62, 129.57, 130.44, 137.77, 138.07, 141.59, 154.54, 158.34, 161.12; UPLC-retention time, 8.70 min; purity 93%. HRMS (ESI) *m*/*z* [M + H]^+^ calcd for C_25_H_22_NO_3_, 384.1594; found, 384.1586.

#### 2,6-Dibenzyloxy-3-(4-butoxyphenyl)pyridine (**35**)^16^

To a solution of compound **34** (0.51 g, 1.3 mmol) and K_2_CO_3_ (0.28 g, 2.0 mmol) in dry DMF (5 mL) was added under an argon atmosphere a solution of 1-bromobutane (0.22 g, 1.6 mmol) in dry DMF (5 mL). The mixture was stirred for 18 h at 70 °C and cooled, and the volatiles were evaporated. The crude product was purified by CC (*n*-hexanes/EtOAc 20:1) to obtain a colorless solid. Yield 0.46 g (79%); mp 50–52 °C; *R*_*f*_ = 0.35 (*n*-hexanes/EtOAc 20:1); ^1^H NMR (400 MHz, CDCl_3_) δ 0.99 (t, *J* = 7.4 Hz, 3H), 1.46–1.53 (m, 2H), 1.74–1.84 (m, 2H), 3.99 (t, *J* = 6.5 Hz, 2H), 5.36 (s, 2H), 5.43 (s, 2H), 6.46 (d, *J* = 8.0 Hz, 1H), 6.90–6.95 (m, 2H), 7.27–7.41 (m, 8H), 7.41–7.46 (m, 2H), 7.46–7.51 (m, 2H), 7.58 (d, *J* = 8.0 Hz, 1H); ^13^C NMR (101 MHz, CDCl_3_) δ 14.02, 19.42, 31.52, 67.68, 67.82, 67.96, 102.45, 114.31, 115.94, 127.36, 127.52, 127.91, 128.47, 128.61, 129.09, 130.16, 137.80, 138.13, 141.60, 158.23, 158.34, 161.03; UPLC-retention time, 10.88 min; purity 99%. HRMS (ESI) *m*/*z* [M + H]^+^ calcd for C_29_H_30_NO_3_, 440.2220; found, 440.2213.

#### *tert*-Butyl *N*-(2-Aminoethyl)carbamate (**36a**)

This compound was used as commercially supplied (BLDPharm Germany).

#### *tert*-Butyl *N*-(4-Aminobutyl)carbamate (**36b**)

This compound was used as commercially supplied (BLDPharm Germany).

#### *tert*-Butyl *N*-(6-Aminohexyl)carbamate (**36c**)

This compound was used as commercially supplied (BLDPharm Germany).

#### *tert*-Butyl *N*-(8-Aminooctyl)carbamate (**36d**)

This compound was used as commercially supplied (BLDPharm Germany).

#### *tert*-Butyl *N*-(10-Aminodecyl)carbamate (**36e**)

This compound was used as commercially supplied (BLDPharm Germany).

#### *tert*-Butyl *N*-[2-(2-Aminoethoxy)ethyl]carbamate (**36f**)

This compound was synthesized as described previously.^57^

#### *tert*-Butyl *N*-[2-[2-(2-Aminoethoxy)ethoxy]ethyl]carbamate (**36g**)

This compound was synthesized as described previously.^57^

#### *tert*-Butyl *N*-(4-Piperidylmethyl)carbamate (**36h**)

This compound was used as commercially supplied (BLDPharm Germany).

#### *tert*-Butyl 3,9-Diazaspiro[5.5]undecane-3-carboxylate (**36j**)

This compound was used as commercially supplied (BLDPharm Germany).

#### *tert*-Butyl *N*-[[1-[4-(Aminomethyl)phenyl]-4-piperidyl]methyl]carbamate (**36k**)

Compound **46** (0.95 g, 3 mmol) was dissolved in dry MeOH (30 mL) and anhydrous CoCl_2_ (0.58 g, 4.5 mmol) was added, followed by portion wise addition of NaBH_4_ (0.57 g, 15 mmol) at 0 °C. The resulting mixture was stirred for 2 h, after which the black suspension was quenched with 1N NaOH (30 mL) and the mixture was transferred to centrifuge tubes. After centrifugation, the supernatant was decanted, and the sediment washed with more of the same solvent. The combined supernatants were extracted with CHCl_3_ (3 × 50 mL), washed with brine (50 mL), dried over Na_2_SO_4_, filtered, and concentrated. The crude product was purified by FC (40 g, 30 μm, gradient from 0 to 40% MeOH in EtOAc + 1% Et_3_N) to give a colorless oil. Yield 0.21 g (22%); *R*_f_ = 0.12 (20% MeOH in EtOAc + 1% Et_3_N); ^1^H NMR (500 MHz, DMSO-*d*_6_) δ 1.12–1.26 (m, 2H), 1.37 (s, 9H), 1.42–1.53 (m, 1H), 1.63–1.70 (m, 2H), 2.51–2.61 (m, 2H), 2.83 (t, *J* = 6.3 Hz, 2H), 3.59 (s, 3H), 3.56–3.65 (m, 4H), 6.84 (d, *J* = 8.6 Hz, 2H), 7.12 (d, *J* = 8.6 Hz, 2H); ^13^C NMR (126 MHz, DMSO-*d*_6_) δ 28.41, 29.37, 36.23, 45.19, 45.58, 49.16, 77.48, 115.94, 127.82, 134.22, 150.15, 155.91; LC–MS (ESI) (90% H_2_O to 100% MeCN in 10 min, then 100% MeCN to 20 min, DAD 220–600 nm), *t*_R_ = 4.88 min, 91% purity. *m*/*z* [M + H]^+^ calcd for C_18_H_30_N_3_O_2_, 320.23; found, 320.2. HRMS (ESI) *m*/*z* [M + H]^+^ calcd for C_29_H_30_NO_3_, 320.2333; found, 320.2332.

#### 4-[2-(*tert*-Butoxycarbonylamino)ethylamino]-2-fluorobenzoic Acid (**37a**)

This compound was prepared using the General Procedure D, 2-fluoro-4-iodobenzoic acid (0.80 g), and **36a** (1.44 g) as the linker. The crude product was purified by FC (50 g, 15 μm, gradient from 0 to 10% MeOH in CH_2_Cl_2_) to give a beige solid. Yield 0.53 g (60%); mp 202–204 °C; *R*_f_ = 0.45 (10% MeOH in CH_2_Cl_2_); ^1^H NMR (500 MHz, DMSO-*d*_6_) δ 1.36 (s, 9H), 3.02–3.15 (m, 4H), 6.30 (dd, *J* = 2.3, 14.5 Hz, 1H), 6.40 (dd, *J* = 2.3, 8.8 Hz, 1H), 6.68 (t, *J* = 5.4 Hz, 1H), 6.84 (t, *J* = 5.6 Hz, 1H), 7.57 (t, *J* = 8.8 Hz, 1H), 12.14 (s, 1H); ^13^C NMR (126 MHz, DMSO) δ 28.35, 42.26, 77.90, 97.79 (d, *J* = 26.2 Hz), 104.72 (d, *J* = 10.1 Hz), 108.00, 133.35 (d, *J* = 3.4 Hz), 154.48 (d, *J* = 12.3 Hz), 155.86, 163.85 (d, *J* = 254.5 Hz), 165.18 (d, *J* = 3.6 Hz); LC–MS (ESI) (90% H_2_O to 100% MeCN in 10 min, then 100% MeCN to 20 min, DAD 220–600 nm), *t*_R_ = 1.66 min, 98% purity. *m*/*z* [M + H]^+^ calcd for C_14_H_20_FN_2_O_4_, 299.14; found, 299.1. HRMS (ESI) *m*/*z* [M + H]^+^ calcd for C_14_H_20_FN_2_O_4_, 299.1402; found, 299.1398.

#### 4-[4-(*tert*-Butoxycarbonylamino)butylamino]-2-fluorobenzoic Acid (**37b**)

This compound was prepared using the General Procedure D, 2-fluoro-4-iodobenzoic acid (0.80 g), and **36b** (1.69 g) as the linker. The crude product was purified by FC (50 g, 15 μm, gradient from 0 to 10% MeOH in CH_2_Cl_2_) to give a colorless solid. Yield 0.77 g (79%); mp 120–122 °C; *R*_f_ = 0.52 (10% MeOH in CH_2_Cl_2_); ^1^H NMR (600 MHz, DMSO-*d*_6_) δ 1.36 (s, 9H), 1.40–1.53 (m, 4H), 2.93 (q, *J* = 6.4 Hz, 2H), 3.03 (q, *J* = 6.5 Hz, 2H), 6.26 (dd, *J* = 2.2, 14.5 Hz, 1H), 6.38 (dd, *J* = 2.2, 8.8 Hz, 1H), 6.68 (t, *J* = 5.5 Hz, 1H), 6.78 (t, *J* = 5.8 Hz, 1H), 7.57 (t, *J* = 8.8 Hz, 1H), 12.12 (s, 1H); ^13^C NMR (151 MHz, DMSO) δ 25.89, 27.21, 28.43, 40.23, 42.22, 77.52, 97.72 (d, *J* = 25.7 Hz), 104.34 (d, *J* = 9.7 Hz), 107.90, 133.36, 154.66 (d, *J* = 12.2 Hz), 155.78, 163.88 (d, *J* = 254.5 Hz), 165.24; LC–MS (ESI) (90% H_2_O to 100% MeCN in 10 min, then 100% MeCN to 20 min, DAD 220–600 nm), *t*_R_ = 3.52 min, 98% purity. *m*/*z* [M – H]^−^ calcd for C_16_H_22_FN_2_O_4_, 325.16; found, 325.2. HRMS (ESI) *m*/*z* [M + H]^+^ calcd for C_16_H_24_FN_2_O_4_, 327.1715; found, 327.1715.

#### 4-[6-(*tert*-butoxycarbonylamino)hexylamino]-2-fluorobenzoic Acid (**37c**)

This compound was prepared using the General Procedure D, 2-fluoro-4-iodobenzoic acid (0.80 g), and **36c** (1.95 g) as the linker. The crude product was purified by FC (80 g, 30 μm, gradient from 0 to 10% MeOH in CH_2_Cl_2_) to give a colorless solid. Yield 0.63 g (59%); mp 68–70 °C; *R*_f_ = 0.50 (10% MeOH in CH_2_Cl_2_); ^1^H NMR (600 MHz, DMSO-*d*_6_) δ 1.21–1.35 (m, 6H), 1.36 (s, 9H), 1.44–1.54 (m, 2H), 2.89 (q, *J* = 6.6 Hz, 2H), 2.99–3.05 (m, 2H), 6.25 (dd, *J* = 2.2, 14.5 Hz, 1H), 6.38 (dd, *J* = 2.2, 8.8 Hz, 1H), 6.66 (t, *J* = 5.4 Hz, 1H), 6.73 (t, *J* = 5.7 Hz, 1H), 7.57 (t, *J* = 8.8 Hz, 1H), 12.12 (s, 1H); ^13^C NMR (151 MHz, DMSO) δ 26.18, 26.37, 28.43, 28.48, 29.61, 40.24, 42.42, 77.45, 97.70 (d, *J* = 26.0 Hz), 104.34 (d, *J* = 9.9 Hz), 107.84, 133.37, 154.64, 154.72, 155.75, 163.87 (d, *J* = 254.7 Hz), 165.24, 165.27; LC–MS (ESI) (90% H_2_O to 100% MeCN in 10 min, then 100% MeCN to 20 min, DAD 220–600 nm), *t*_R_ = 4.78 min, 99% purity. *m*/*z* [M – H]^−^ calcd for C_18_H_22_FN_2_O_4_, 353.19; found, 353.2. HRMS (ESI) *m*/*z* [M + H]^+^ calcd for C_18_H_28_FN_2_O_4_, 355.2028; found, 355.2028.

#### 4-[8-(*tert*-Butoxycarbonylamino)octylamino]-2-fluorobenzoic Acid (**37d**)

This compound was prepared using the General Procedure D, 2-fluoro-4-iodobenzoic acid (0.80 g), and **36d** (2.20 g) as the linker. The crude product was purified by FC (50 g, 15 μm, gradient from 0 to 10% MeOH in CH_2_Cl_2_) to give a colorless solid. Yield 0.22 g (19%); mp 104–106 °C; *R*_f_ = 0.62 (10% MeOH in CH_2_Cl_2_); ^1^H NMR (500 MHz, DMSO-*d*_6_) δ 1.19–1.35 (m, 10H), 1.36 (s, 9H), 1.46–1.56 (m, 2H), 2.88 (q, *J* = 6.7 Hz, 2H), 2.99–3.06 (m, 2H), 6.25 (dd, *J* = 2.2, 14.5 Hz, 1H), 6.38 (dd, *J* = 2.2, 8.8 Hz, 1H), 6.64 (t, *J* = 5.4 Hz, 1H), 6.70 (t, *J* = 5.7 Hz, 1H), 7.57 (t, *J* = 8.8 Hz, 1H), 12.10 (s, 1H); ^13^C NMR (126 MHz, DMSO) δ 26.35, 26.61, 28.40, 28.50, 28.82, 28.89, 29.58, 40.29, 42.47, 77.39, 97.68 (d, *J* = 26.5 Hz), 104.33 (d, *J* = 9.8 Hz), 107.80, 133.33, 154.66 (d, *J* = 12.5 Hz), 155.70, 163.83 (d, *J* = 254.2 Hz), 165.21; LC–MS (ESI) (90% H_2_O to 100% MeCN in 10 min, then 100% MeCN to 20 min, DAD 220–600 nm), *t*_R_ = 5.80 min, 98% purity. *m*/*z* [M – H]^−^ calcd for C_20_H_30_FN_2_O_4_, 381.22; found, 381.2. HRMS (ESI) *m*/*z* [M + H]^+^ calcd for C_20_H_32_FN_2_O_4_, 383.2341; found, 383.2342.

#### 4-[10-(*tert*-Butoxycarbonylamino)decylamino]-2-fluorobenzoic Acid (**37e**)

This compound was prepared using the General Procedure D, 2-fluoro-4-iodobenzoic acid (0.80 g), and **36e** (2.45 g) as the linker. The crude product was purified by FC (80 g, 30 μm, gradient from 0 to 10% MeOH in CH_2_Cl_2_) to give a colorless solid. Yield 0.38 g (31%); mp 120–122 °C; *R*_f_ = 0.58 (10% MeOH in CH_2_Cl_2_); ^1^H NMR (600 MHz, DMSO-*d*_6_) δ 1.16–1.34 (m, 14H), 1.35 (s, 9H), 1.51 (p, *J* = 7.1 Hz, 2H), 2.87 (q, *J* = 6.6 Hz, 2H), 3.02 (td, *J* = 5.4, 7.0 Hz, 2H), 6.25 (dd, *J* = 2.3, 14.5 Hz, 1H), 6.38 (dd, *J* = 2.2, 8.8 Hz, 1H), 6.66 (t, *J* = 5.4 Hz, 1H), 6.71 (t, *J* = 5.8 Hz, 1H), 7.56 (t, *J* = 8.8 Hz, 1H), 12.11 (s, 1H); ^13^C NMR (151 MHz, DMSO) δ 26.40, 26.69, 28.42, 28.53, 28.85, 28.94, 29.10, 29.61, 40.23, 42.48, 77.41, 97.69 (d, *J* = 26.1 Hz), 104.31 (d, *J* = 9.8 Hz), 107.84, 133.36, 154.69 (d, *J* = 12.7 Hz), 155.72, 163.86 (d, *J* = 254.5 Hz), 165.26; LC–MS (ESI) (90% H_2_O to 100% MeCN in 10 min, then 100% MeCN to 20 min, DAD 220–600 nm), *t*_R_ = 6.85 min, 97% purity. *m*/*z* [M – H]^−^ calcd for C_22_H_36_FN_2_O_4_, 409.25; found, 409.3. HRMS (ESI) *m*/*z* [M + H]^+^ calcd for C_22_H_36_FN_2_O_4_, 411.2654; found, 411.2653.

#### 4-[2-[2-(*tert*-Butoxycarbonylamino)ethoxy]ethylamino]-2-fluorobenzoic Acid (**37f**)

This compound was prepared using the General Procedure D, 2-fluoro-4-iodobenzoic acid (0.80 g), and **36f** (1.84 g) as the linker. The crude product was purified by FC (80 g, 30 μm, gradient from 0 to 10% MeOH in CH_2_Cl_2_) to give a colorless semisolid. Yield 0.56 g (55%); *R*_f_ = 0.50 (10% MeOH in CH_2_Cl_2_); ^1^H NMR (500 MHz, DMSO-*d*_6_) δ 1.36 (s, 9H), 3.08 (q, *J* = 5.9 Hz, 2H), 3.22 (q, *J* = 5.6 Hz, 2H), 3.40 (t, *J* = 6.0 Hz, 2H), 3.52 (t, *J* = 5.6 Hz, 2H), 6.33 (dd, *J* = 2.2, 14.5 Hz, 1H), 6.44 (dd, *J* = 2.2, 8.8 Hz, 1H), 6.67 (t, *J* = 5.6 Hz, 1H), 6.74 (d, *J* = 6.3 Hz, 1H), 7.58 (t, *J* = 8.8 Hz, 1H), 12.13 (s, 1H); ^13^C NMR (126 MHz, DMSO) δ 28.36, 42.41, 68.52, 69.30, 77.77, 98.03 (d, *J* = 25.6 Hz), 104.71 (d, *J* = 10.0 Hz), 107.98, 133.33, 154.53 (d, *J* = 12.4 Hz), 155.77, 163.78 (d, *J* = 254.4 Hz), 165.20 (d, *J* = 3.5 Hz); LC–MS (ESI) (90% H_2_O to 100% MeCN in 10 min, then 100% MeCN to 20 min, DAD 220–600 nm), *t*_R_ = 2.90 min, 98% purity. *m*/*z* [M – H]^−^ calcd for C_16_H_22_FN_2_O_5_, 341.15; found, 341.1. HRMS (ESI) *m*/*z* [M + H]^+^ calcd for C_16_H_24_FN_2_O_5_, 343.1664; found, 343.1661.

#### 4-[2-[2-[2-(*tert*-Butoxycarbonylamino)ethoxy]ethoxy]ethylamino]-2-fluorobenzoic Acid (**37g**)

This compound was prepared using the General Procedure D, 2-fluoro-4-iodobenzoic acid (0.80 g), and **36g** (2.23 g) as the linker. The crude product was purified by FC (80 g, 30 μm, gradient from 0 to 5% MeOH in CH_2_Cl_2_) to give a brownish oil. Yield 0.44 g (38%); *R*_f_ = 0.57 (10% MeOH in CH_2_Cl_2_); ^1^H NMR (600 MHz, DMSO-*d*_6_) δ 1.36 (s, 9H), 3.05 (q, *J* = 6.1 Hz, 2H), 3.24 (q, *J* = 5.6 Hz, 2H), 3.37 (t, *J* = 6.1 Hz, 2H), 3.46–3.56 (m, 6H), 6.33 (dd, *J* = 2.3, 14.5 Hz, 1H), 6.43 (dd, *J* = 2.3, 8.8 Hz, 1H), 6.71 (q, *J* = 6.4 Hz, 2H), 7.57 (t, *J* = 8.7 Hz, 1H), 12.15 (s, 1H); ^13^C NMR (151 MHz, DMSO) δ 28.38, 40.24, 40.61, 42.43, 68.89, 69.34, 69.64, 69.84, 77.74, 97.92, 98.08, 104.67, 108.00, 128.29, 128.71, 133.36, 154.51, 154.59, 155.74, 162.96, 164.65, 165.23; LC–MS (ESI) (90% H_2_O to 100% MeCN in 10 min, then 100% MeCN to 20 min, DAD 220–600 nm), *t*_R_ = 3.35 min, 99% purity. *m*/*z* [M + H]^+^ calcd for C_18_H_28_FN_2_O_6_, 387.19; found, 387.2. HRMS (ESI) *m*/*z* [M + H]^+^ calcd for C_18_H_28_FN_2_O_6_, 387.1926; found, 387.1914.

#### 4-[4-[(*tert*-Butoxycarbonylamino)methyl]-1-piperidyl]-2-fluorobenzoic Acid (**37h**)

This compound was prepared using the General Procedure D, 2-fluoro-4-iodobenzoic acid (0.80 g), and **36h** (1.45 g) as the linker. The crude product was purified by FC (80 g, 30 μm, gradient from 0 to 10% MeOH in CH_2_Cl_2_) to give a colorless solid. Yield 0.22 g (20%); mp 172–174 °C; *R*_f_ = 0.44 (10% MeOH in CH_2_Cl_2_); ^1^H NMR (600 MHz, DMSO-*d*_6_) δ 1.05–1.15 (m, 2H), 1.36 (s, 9H), 1.55–1.65 (m, 2H), 1.66 (d, *J* = 3.6 Hz, 1H), 2.76–2.81 (m, 1H), 2.82 (t, *J* = 6.3 Hz, 3H), 3.85–3.91 (m, 2H), 6.67 (dd, *J* = 2.5, 15.4 Hz, 1H), 6.74 (dd, *J* = 2.5, 9.0 Hz, 1H), 6.86 (t, *J* = 6.0 Hz, 1H), 7.65 (t, *J* = 9.1 Hz, 1H), 12.34 (s, 1H); ^13^C NMR (151 MHz, DMSO) δ 28.42, 28.85, 36.22, 45.37, 46.82, 77.55, 100.72 (d, *J* = 26.5 Hz), 106.01 (d, *J* = 10.0 Hz), 109.29, 133.31, 155.13 (d, *J* = 11.9 Hz), 155.93, 163.56 (d, *J* = 254.1 Hz), 165.03 (d, *J* = 3.6 Hz); LC–MS (ESI) (90% H_2_O to 100% MeCN in 10 min, then 100% MeCN to 20 min, DAD 220–600 nm), *t*_R_ = 3.76 min, 98% purity. *m*/*z* [M + H]^+^ calcd for C_18_H_26_FN_2_O_4_, 353.19; found, 353.2. HRMS (ESI) *m*/*z* [M + H]^+^ calcd for C_18_H_26_FN_2_O_4_, 353.1871; found, 353.1866.

#### 4-(3-*tert*-Butoxycarbonyl-3,9-diazaspiro[5.5]undecan-9-yl)-2-fluorobenzoic Acid (**37j**)

This compound was prepared using the General Procedure D, 2-fluoro-4-iodobenzoic acid (0.80 g), and **36j** (2.29 g) as the linker. The crude product was purified by FC (80 g, 30 μm, gradient from 0 to 10% MeOH in CH_2_Cl_2_) to give a colorless solid. Yield 0.43 g (36%); mp 240–242 °C; *R*_f_ = 0.49 (10% MeOH in CH_2_Cl_2_); ^1^H NMR (500 MHz, DMSO-*d*_6_) δ 1.38 (s, 9H), 1.33–1.47 (m, 4H), 1.50 (dd, *J* = 3.9, 7.8 Hz, 4H), 3.30–3.34 (m, 8H), 6.66 (dd, *J* = 2.5, 15.4 Hz, 1H), 6.73 (dd, *J* = 2.5, 8.9 Hz, 1H), 7.66 (t, *J* = 9.0 Hz, 1H), 12.32 (s, 1H); ^13^C NMR (126 MHz, DMSO) δ 28.26, 29.64, 34.17, 34.80, 40.20, 42.42, 78.56, 100.48 (d, *J* = 26.9 Hz), 106.01 (d, *J* = 10.3 Hz), 109.04, 133.25 (d, *J* = 3.2 Hz), 154.11, 155.17 (d, *J* = 11.5 Hz), 163.51 (d, *J* = 254.2 Hz), 165.02 (d, *J* = 3.6 Hz); LC–MS (ESI) (90% H_2_O to 100% MeCN in 10 min, then 100% MeCN to 20 min, DAD 220–600 nm), *t*_R_ = 4.90 min, 98% purity. *m*/*z* [M + H]^+^ calcd for C_21_H_30_FN_2_O_4_, 393.22; found, 393.2. HRMS (ESI) *m*/*z* [M + H]^+^ calcd for C_21_H_30_FN_2_O_4_, 393.2184; found, 393.2176.

#### 4-[[4-[4-[(*tert*-Butoxycarbonylamino)methyl]-1-piperidyl]phenyl]methylamino]-2-fluorobenzoic Acid (**37k**)

This compound was prepared using the General Procedure D, 2-fluoro-4-iodobenzoic acid (0.80 g), and **36k** (2.88 g) as the linker. The crude product was purified by FC (80 g, 30 μm, gradient from 0 to 10% MeOH in CH_2_Cl_2_) to give a red solid. Yield 233 mg (17%); *R*_f_ = 0.42 (10% MeOH in CH_2_Cl_2_). Due to its low chemical stability, the material was directly used in the next step without further characterization.

#### 4-[4-[(tert-Butoxycarbonylamino)methyl]-1-piperidyl]-2-methoxybenzoic acid (**38h**)

This compound was prepared using the General Procedure D, 4-iodo-2-methoxybenzoic acid (1.39 g), and **36h** (3.21 g) as the linker. The crude product was purified by CC (gradient from 0 to 10% MeOH in CH_2_Cl_2_) to give a gray solid. Yield 0.84 g (46%). The product was used in the next step without further purification and characterization.

#### 4-[4-[(*tert*-Butoxycarbonylamino)methyl]-1-piperidyl]benzoic Acid (**39h**)

This compound was prepared using the General Procedure D, 4-iodobenzoic acid (0.74 g), and **36h** (1.45 g) as the linker. The crude product was purified by FC (80 g, 30 μm, gradient from 0 to 10% MeOH in CH_2_Cl_2_) to give a colorless semisolid. Yield 0.64 g (64%); *R*_f_ = 0.43 (7% MeOH in CH_2_Cl_2_); ^1^H NMR (600 MHz, DMSO-*d*_6_) δ 1.08–1.18 (m, 2H), 1.37 (s, 9H), 1.53–1.63 (m, 1H), 1.67 (dd, *J* = 3.5, 13.8 Hz, 2H), 2.73–2.80 (m, 2H), 2.82 (t, *J* = 6.3 Hz, 2H), 3.83–3.90 (m, 2H), 6.85 (t, *J* = 6.0 Hz, 1H), 6.92 (d, *J* = 8.9 Hz, 2H), 7.70–7.75 (m, 2H), 12.16 (s, 1H); ^13^C NMR (151 MHz, DMSO) δ 28.43, 28.97, 36.30, 45.46, 47.12, 77.54, 113.48, 118.75, 131.07, 153.77, 155.93, 167.42; LC–MS (ESI) (90% H_2_O to 100% MeCN in 10 min, then 100% MeCN to 20 min, DAD 220–600 nm), *t*_R_ = 4.19 min, 96% purity. *m*/*z* [M – H]^−^ calcd for C_18_H_25_N_2_O_4_, 333.18; found, 333.2. HRMS (ESI) *m*/*z* [M + H]^+^ calcd for C_18_H_27_N_2_O_4_, 335.1965; found, 335.1967.

#### *tert*-Butyl *N*-[2-[4-[(2,6-Dioxo-3-piperidyl)carbamoyl]-3-fluoroanilino]ethyl]carbamate (**40a**)

This compound was prepared using the General Procedure B (0.5 mmol scale) and acid **37a** (149 mg). The crude product was purified by FC (40 g, 30 μm, gradient from 0 to 10% MeOH in EtOAc) to give a light blue solid. Yield 55 mg (27%); mp 234–236 °C; *R*_f_ = 0.31 (80% EtOAc in cyclohexane); ^1^H NMR (500 MHz, DMSO-*d*_6_) δ 1.37 (s, 9H), 1.96–2.17 (m, 2H), 2.50–2.55 (m, 1H), 2.69–2.80 (m, 1H), 3.05–3.13 (m, 4H), 4.65–4.74 (m, 1H), 6.35 (dd, *J* = 2.2, 14.9 Hz, 1H), 6.45 (dd, *J* = 2.2, 8.8 Hz, 1H), 6.53 (s, 1H), 6.85 (d, *J* = 6.5 Hz, 1H), 7.54 (t, *J* = 8.9 Hz, 1H), 7.83 (t, *J* = 7.5 Hz, 1H), 10.79 (s, 1H); ^13^C NMR (126 MHz, DMSO) δ 24.34, 28.36, 31.12, 40.29, 42.40, 49.88, 77.90, 97.28 (d, *J* = 27.8 Hz), 107.97 (d, *J* = 12.8 Hz), 108.36, 131.93 (d, *J* = 4.8 Hz), 153.35 (d, *J* = 12.2 Hz), 155.86, 162.00 (d, *J* = 245.9 Hz), 163.30 (d, *J* = 2.8 Hz), 172.48, 173.06; LC–MS (ESI) (90% H_2_O to 100% MeCN in 10 min, then 100% MeCN to 20 min, DAD 220–600 nm), *t*_R_ = 4.74 min, 99% purity. *m*/*z* [M + H]^+^ calcd for C_19_H_26_FN_4_O_5_, 409.19; found, 409.2. HRMS (ESI) *m*/*z* [M + H]^+^ calcd for C_19_H_26_FN_4_O_5_, 409.1882; found, 409.1875.

#### *tert*-Butyl *N*-[4-[4-[(2,6-Dioxo-3-piperidyl)carbamoyl]-3-fluoroanilino]butyl]carbamate (**40b**)

This compound was prepared using the General Procedure B (0.5 mmol scale) and acid **37b** (163 mg). The crude product was purified by FC (40 g, 30 μm, gradient from 20 to 100% EtOAc in cyclohexane) to give a colorless solid. Yield 157 mg (72%); mp 154–156 °C; *R*_f_ = 0.38 (80% EtOAc in cyclohexane); ^1^H NMR (500 MHz, DMSO-*d*_6_) δ 1.36 (s, 9H), 1.39–1.55 (m, 4H), 1.96–2.15 (m, 2H), 2.46–2.54 (m, 1H), 2.69–2.80 (m, 1H), 2.93 (q, *J* = 6.5 Hz, 2H), 3.03 (q, *J* = 6.5 Hz, 2H), 4.65–4.74 (m, 1H), 6.31 (dd, *J* = 2.2, 15.2 Hz, 1H), 6.43 (dd, *J* = 2.2, 8.8 Hz, 1H), 6.50 (t, *J* = 5.5 Hz, 1H), 6.77 (t, *J* = 5.7 Hz, 1H), 7.53 (t, *J* = 8.9 Hz, 1H), 7.81 (t, *J* = 7.7 Hz, 1H), 10.79 (s, 1H); ^13^C NMR (126 MHz, DMSO) δ 24.36, 25.91, 27.22, 28.42, 31.14, 40.20, 42.32, 49.89, 77.49, 97.19 (d, *J* = 27.5 Hz), 107.57 (d, *J* = 12.7 Hz), 108.25, 131.91 (d, *J* = 4.7 Hz), 153.58 (d, *J* = 12.5 Hz), 155.76, 162.01 (d, *J* = 245.7 Hz), 163.34 (d, *J* = 2.9 Hz), 172.51, 173.07; LC–MS (ESI) (90% H_2_O to 100% MeCN in 10 min, then 100% MeCN to 20 min, DAD 220–600 nm), *t*_R_ = 5.46 min, 99% purity. *m*/*z* [M + H]^+^ calcd for C_21_H_30_FN_4_O_5_, 437.22; found, 437.3. HRMS (ESI) *m*/*z* [M + H]^+^ calcd for C_21_H_30_FN_4_O_5_, 437.2195; found, 437.2196.

#### *tert*-Butyl *N*-[6-[4-[(2,6-Dioxo-3-piperidyl)carbamoyl]-3-fluoroanilino]hexyl]carbamate (**40c**)

This compound was prepared using the General Procedure B (0.5 mmol scale) and acid **37c** (177 mg). The crude product was purified by FC (40 g, 30 μm, gradient from 30 to 100% EtOAc in cyclohexane) to give a colorless solid. Yield 179 mg (77%); mp 138–140 °C; *R*_f_ = 0.50 (80% EtOAc in cyclohexane); ^1^H NMR (600 MHz, DMSO-*d*_6_) δ 1.22–1.35 (m, 6H), 1.36 (s, 9H), 1.45–1.55 (m, 2H), 1.96–2.04 (m, 1H), 2.04–2.14 (m, 1H), 2.50–2.54 (m, 1H), 2.70–2.79 (m, 1H), 2.89 (q, *J* = 6.6 Hz, 2H), 2.99–3.05 (m, 2H), 4.66–4.73 (m, 1H), 6.30 (dd, *J* = 2.2, 15.1 Hz, 1H), 6.43 (dd, *J* = 2.2, 8.8 Hz, 1H), 6.50 (t, *J* = 5.4 Hz, 1H), 6.73 (t, *J* = 5.8 Hz, 1H), 7.53 (t, *J* = 8.9 Hz, 1H), 7.82 (t, *J* = 7.7 Hz, 1H), 10.80 (s, 1H); ^13^C NMR (151 MHz, DMSO) δ 24.37, 26.21, 26.39, 28.43, 28.51, 29.62, 31.16, 40.24, 42.53, 49.89, 77.45, 97.17 (d, *J* = 27.3 Hz), 107.54 (d, *J* = 12.7 Hz), 108.24, 131.94 (d, *J* = 4.8 Hz), 153.62 (d, *J* = 12.1 Hz), 155.75, 162.03 (d, *J* = 245.6 Hz), 163.37, 172.55, 173.12; LC–MS (ESI) (90% H_2_O to 100% MeCN in 10 min, then 100% MeCN to 20 min, DAD 220–600 nm), *t*_R_ = 6.24 min, 99% purity. *m*/*z* [M + H]^+^ calcd for C_23_H_34_FN_4_O_5_, 465.25; found, 465.3. HRMS (ESI) *m*/*z* [M + H]^+^ calcd for C_23_H_34_FN_4_O_5_, 465.2508; found, 465.2512.

#### *tert*-Butyl *N*-[8-[4-[(2,6-Dioxo-3-piperidyl)carbamoyl]-3-fluoroanilino]octyl]carbamate (**40d**)

This compound was prepared using the General Procedure B (0.5 mmol scale) and acid **37d** (191 mg). The crude product was purified by FC (25 g, 15 μm, gradient from 30 to 100% EtOAc in cyclohexane) to give a colorless solid. Yield 148 mg (60%); mp 136–138 °C; *R*_f_ = 0.55 (80% EtOAc in cyclohexane); ^1^H NMR (600 MHz, DMSO-*d*_6_) δ 1.17–1.35 (m, 10H), 1.35 (s, 9H), 1.48–1.56 (m, 2H), 1.97–2.04 (m, 1H), 2.04–2.14 (m, 1H), 2.51 (dd, *J* = 2.8, 4.5 Hz, 1H), 2.70–2.79 (m, 1H), 2.88 (q, *J* = 6.6 Hz, 2H), 2.99–3.05 (m, 2H), 4.66–4.73 (m, 1H), 6.30 (dd, *J* = 2.2, 15.2 Hz, 1H), 6.43 (dd, *J* = 2.2, 8.8 Hz, 1H), 6.50 (t, *J* = 5.3 Hz, 1H), 6.71 (t, *J* = 5.8 Hz, 1H), 7.53 (t, *J* = 8.9 Hz, 1H), 7.81 (t, *J* = 7.7 Hz, 1H), 10.79 (s, 1H); ^13^C NMR (151 MHz, DMSO) δ 24.37, 26.39, 26.66, 28.43, 28.55, 28.86, 28.95, 29.60, 31.16, 40.23, 42.59, 77.42, 97.16 (d, *J* = 27.4 Hz), 107.53 (d, *J* = 13.0 Hz), 108.23, 131.94 (d, *J* = 4.5 Hz), 153.63 (d, *J* = 12.2 Hz), 155.73, 162.03 (d, *J* = 245.5 Hz), 163.36, 172.55, 173.11; LC–MS (ESI) (90% H_2_O to 100% MeCN in 10 min, then 100% MeCN to 20 min, DAD 220–600 nm), *t*_R_ = 7.00 min, 99% purity. *m*/*z* [M + H]^+^ calcd for C_25_H_38_FN_4_O_5_, 493.28; found, 493.4. HRMS (ESI) *m*/*z* [M + H]^+^ calcd for C_25_H_38_FN_4_O_5_, 493.2821; found, 493.2822.

#### *tert*-Butyl *N*-[10-[4-[(2,6-Dioxo-3-piperidyl)carbamoyl]-3-fluoroanilino]decyl]carbamate (**40e**)

This compound was prepared using the General Procedure B (0.5 mmol scale) and acid **37e** (205 mg). The crude product was purified by FC (25 g, 15 μm, gradient from 20 to 100% EtOAc in cyclohexane) to give a colorless solid. Yield 182 mg (70%); mp 144–146 °C; *R*_f_ = 0.59 (80% EtOAc in cyclohexane); ^1^H NMR (500 MHz, DMSO-*d*_6_) δ 1.17–1.35 (m, 14H), 1.36 (s, 9H), 1.46–1.57 (m, 2H), 1.96–2.15 (m, 2H), 2.48–2.54 (m, 1H), 2.69–2.80 (m, 1H), 2.87 (q, *J* = 6.7 Hz, 2H), 2.99–3.06 (m, 2H), 4.65–4.74 (m, 1H), 6.30 (dd, *J* = 2.2, 15.1 Hz, 1H), 6.43 (dd, *J* = 2.2, 8.8 Hz, 1H), 6.49 (t, *J* = 5.4 Hz, 1H), 6.69 (t, *J* = 5.5 Hz, 1H), 7.53 (t, *J* = 8.9 Hz, 1H), 7.80 (t, *J* = 7.7 Hz, 1H), 10.79 (s, 1H); ^13^C NMR (126 MHz, DMSO) δ 24.35, 26.38, 26.68, 28.40, 28.54, 28.83, 28.94, 29.08, 29.59, 31.13, 40.12, 42.57, 49.88, 77.39, 97.15 (d, *J* = 27.2 Hz), 107.52 (d, *J* = 12.7 Hz), 108.21, 131.91 (d, *J* = 4.7 Hz), 153.61 (d, *J* = 12.5 Hz), 155.70, 162.00 (d, *J* = 245.8 Hz), 163.34 (d, *J* = 2.9 Hz), 172.51, 173.06; LC–MS (ESI) (90% H_2_O to 100% MeCN in 10 min, then 100% MeCN to 20 min, DAD 220–600 nm), *t*_R_ = 7.84 min, 98% purity. *m*/*z* [M + H]^+^ calcd for C_27_H_42_FN_4_O_5_, 521.31; found, 521.5. HRMS (ESI) *m*/*z* [M + H]^+^ calcd for C_27_H_42_FN_4_O_5_, 521.3134; found, 521.3138.

#### *tert*-Butyl *N*-[2-[2-[4-[(2,6-Dioxo-3-piperidyl)carbamoyl]-3-fluoroanilino]ethoxy]ethyl]carbamate (**40f**)

This compound was prepared using the General Procedure B (0.5 mmol scale) and acid **37f** (171 mg). The crude product was purified by FC (25 g, 15 μm, gradient from 50 to 100% EtOAc in cyclohexane) to give a colorless solid. Yield 154 mg (68%); mp 96–98 °C; *R*_f_ = 0.47 (EtOAc); ^1^H NMR (600 MHz, DMSO-*d*_6_) δ 1.17–1.35 (m, 10H), 1.35 (s, 9H), 1.48–1.56 (m, 2H), 1.97–2.04 (m, 1H), 2.04–2.14 (m, 1H), 2.51 (dd, *J* = 2.8, 4.5 Hz, 1H), 2.70–2.79 (m, 1H), 2.88 (q, *J* = 6.6 Hz, 2H), 2.99–3.05 (m, 2H), 4.66–4.73 (m, 1H), 6.30 (dd, *J* = 2.2, 15.2 Hz, 1H), 6.43 (dd, *J* = 2.2, 8.8 Hz, 1H), 6.50 (t, *J* = 5.3 Hz, 1H), 6.71 (t, *J* = 5.8 Hz, 1H), 7.53 (t, *J* = 8.9 Hz, 1H), 7.81 (t, *J* = 7.7 Hz, 1H), 10.79 (s, 1H); ^13^C NMR (151 MHz, DMSO) δ 24.37, 26.39, 26.66, 28.43, 28.55, 28.86, 28.95, 29.60, 31.16, 40.23, 42.59, 77.42, 97.16 (d, *J* = 27.4 Hz), 107.53 (d, *J* = 13.0 Hz), 108.23, 131.94 (d, *J* = 4.5 Hz), 153.63 (d, *J* = 12.2 Hz), 155.73, 162.03 (d, *J* = 245.5 Hz), 163.36, 172.55, 173.11; LC–MS (ESI) (90% H_2_O to 100% MeCN in 10 min, then 100% MeCN to 20 min, DAD 220–600 nm), *t*_R_ = 4.97 min, 99% purity. *m*/*z* [M + H]^+^ calcd for C_21_H_30_FN_4_O_6_, 453.21; found, 453.2. HRMS (ESI) *m*/*z* [M + H]^+^ calcd for C_21_H_30_FN_4_O_6_, 453.2144; found, 453.2137.

#### *tert*-Butyl *N*-[2-[2-[2-[4-[(2,6-Dioxo-3-piperidyl)carbamoyl]-3-fluoroanilino]ethoxy]ethoxy]ethyl]carbamate (**40g**)

This compound was prepared using the General Procedure B (0.5 mmol scale) and acid **37g** (193 mg). The crude product was purified by FC (25 g, 15 μm, gradient from 50 to 100% EtOAc in cyclohexane) to give a colorless solid. Yield 184 mg (74%); mp 76–78 °C; *R*_f_ = 0.32 (EtOAc); ^1^H NMR (500 MHz, DMSO-*d*_6_) δ 1.36 (s, 9H), 1.97–2.15 (m, 2H), 2.49–2.55 (m, 1H), 2.69–2.80 (m, 1H), 3.05 (q, *J* = 6.1 Hz, 2H), 3.24 (q, *J* = 5.6 Hz, 2H), 3.37 (t, *J* = 6.1 Hz, 2H), 3.47–3.58 (m, 6H), 4.65–4.74 (m, 1H), 6.38 (dd, *J* = 2.2, 15.1 Hz, 1H), 6.45–6.55 (m, 2H), 6.70 (d, *J* = 6.1 Hz, 1H), 7.53 (t, *J* = 8.9 Hz, 1H), 7.83 (t, *J* = 7.6 Hz, 1H), 10.79 (s, 1H); ^13^C NMR (126 MHz, DMSO) δ 24.35, 28.36, 31.13, 42.53, 49.88, 68.93, 69.33, 69.63, 69.84, 77.73, 97.48 (d, *J* = 27.5 Hz), 107.90 (d, *J* = 12.7 Hz), 108.34, 131.90 (d, *J* = 4.7 Hz), 153.43 (d, *J* = 12.5 Hz), 158.35 (d, *J* = 658.7 Hz), 162.92, 163.33 (d, *J* = 2.8 Hz), 172.49, 173.07; LC–MS (ESI) (90% H_2_O to 100% MeCN in 10 min, then 100% MeCN to 20 min, DAD 220–600 nm), *t*_R_ = 5.12 min, 99% purity. *m*/*z* [M + H]^+^ calcd for C_23_H_34_FN_4_O_7_, 497.24; found, 497.4. HRMS (ESI) *m*/*z* [M + H]^+^ calcd for C_23_H_34_FN_4_O_7_, 497.2406; found, 497.2406.

#### *tert*-Butyl *N*-[[1-[4-[(2,6-Dioxo-3-piperidyl)carbamoyl]-3-fluorophenyl]-4-piperidyl]methyl]carbamate (**40h**)

This compound was prepared using the General Procedure B (0.5 mmol scale) and acid **37h** (176 mg). The crude product was purified by FC (25 g, 15 μm, gradient from 50 to 100% EtOAc in cyclohexane) to give a colorless solid. Yield 190 mg (80%); mp 190–192 °C; *R*_f_ = 0.66 (EtOAc); ^1^H NMR (500 MHz, DMSO-*d*_6_) δ 1.06–1.20 (m, 2H), 1.37 (s, 9H), 1.55–1.64 (m, 1H), 1.64–1.70 (m, 2H), 1.97–2.05 (m, 1H), 2.05–2.16 (m, 1H), 2.50–2.55 (m, 1H), 2.70–2.86 (m, 5H), 3.86 (d, *J* = 13.3 Hz, 2H), 4.71 (ddd, *J* = 5.3, 7.8, 12.6 Hz, 1H), 6.72 (dd, *J* = 2.4, 15.8 Hz, 1H), 6.78 (dd, *J* = 2.5, 8.9 Hz, 1H), 6.84 (t, *J* = 6.1 Hz, 1H), 7.60 (t, *J* = 9.0 Hz, 1H), 7.96 (t, *J* = 7.4 Hz, 1H), 10.79 (s, 1H); ^13^C NMR (126 MHz, DMSO-*d*_6_) δ 24.29, 28.41, 28.83, 31.11, 36.20, 40.29, 45.41, 47.07, 49.87, 77.53, 100.53 (d, *J* = 28.0 Hz), 109.54 (d, *J* = 12.9 Hz), 109.88, 131.80 (d, *J* = 4.5 Hz), 154.31 (d, *J* = 11.5 Hz), 155.92, 161.65 (d, *J* = 246.1 Hz), 163.16 (d, *J* = 2.8 Hz), 172.39, 173.06; LC–MS (ESI) (90% H_2_O to 100% MeCN in 10 min, then 100% MeCN to 20 min, DAD 220–600 nm), *t*_R_ = 5.78 min, 98% purity. *m*/*z* [M + H]^+^ calcd for C_23_H_32_FN_4_O_5_, 463.24; found, 463.3. HRMS (ESI) *m*/*z* [M + H]^+^ calcd for C_23_H_32_FN_4_O_5_, 463.2351; found, 463.2344.

#### *tert*-Butyl 9-[4-[(2,6-Dioxo-3-piperidyl)carbamoyl]-3-fluorophenyl]-3,9-diazaspiro[5.5]undecane-3-carboxylate (**40j**)

This compound was prepared using the General Procedure B (0.5 mmol scale) and acid **37j** (178 mg). The crude product was purified by FC (25 g, 15 μm, gradient from 50 to 100% EtOAc in cyclohexane) to give a colorless solid. Yield 190 mg (71%); mp 238–240 °C; *R*_f_ = 0.45 (80% EtOAc in cyclohexane); ^1^H NMR (500 MHz, DMSO-*d*_6_) δ 1.06–1.20 (m, 2H), 1.37 (s, 9H), 1.55–1.64 (m, 1H), 1.64–1.70 (m, 2H), 1.97–2.05 (m, 1H), 2.05–2.16 (m, 1H), 2.50–2.55 (m, 1H), 2.70–2.86 (m, 5H), 3.86 (d, *J* = 13.3 Hz, 2H), 4.71 (ddd, *J* = 5.3, 7.8, 12.6 Hz, 1H), 6.72 (dd, *J* = 2.4, 15.8 Hz, 1H), 6.78 (dd, *J* = 2.5, 8.9 Hz, 1H), 6.84 (t, *J* = 6.1 Hz, 1H), 7.60 (t, *J* = 9.0 Hz, 1H), 7.96 (t, *J* = 7.4 Hz, 1H), 10.79 (s, 1H); ^13^C NMR (126 MHz, DMSO-*d*_6_) δ 24.29, 28.41, 28.83, 31.11, 36.20, 40.29, 45.41, 47.07, 49.87, 77.53, 100.53 (d, *J* = 28.0 Hz), 109.54 (d, *J* = 12.9 Hz), 109.88, 131.80 (d, *J* = 4.5 Hz), 154.31 (d, *J* = 11.5 Hz), 155.92, 161.65 (d, *J* = 246.1 Hz), 163.16 (d, *J* = 2.8 Hz), 172.39, 173.06; LC–MS (ESI) (90% H_2_O to 100% MeCN in 10 min, then 100% MeCN to 20 min, DAD 220–600 nm), *t*_R_ = 6.78 min, 94% purity. *m*/*z* [M + H]^+^ calcd for C_26_H_36_FN_4_O_5_, 503.26; found, 503.4. HRMS (ESI) *m*/*z* [M + H]^+^ calcd for C_26_H_36_FN_4_O_5_, 503.2664; found, 503.2657.

#### *tert*-Butyl *N*-[[1-[4-[[4-[(2,6-Dioxo-3-piperidyl)carbamoyl]-3-fluoroanilino]methyl]phenyl]-4-piperidyl]methyl]carbamate (**40k**)

This compound was prepared using the General Procedure B (0.5 mmol scale) and crude acid **37k** (229 mg). The crude product was purified by CC (80% EtOAc in cyclohexane) to give a gray solid. Yield 196 mg (69%); mp 128–130 °C; *R*_f_ = 0.43 (80% EtOAc in cyclohexane); ^1^H NMR (600 MHz, DMSO-*d*_6_) δ 1.12–1.21 (m, 2H), 1.37 (s, 9H), 1.43–1.52 (m, 1H), 1.66 (dd, *J* = 3.6, 13.4 Hz, 2H), 1.95–2.03 (m, 1H), 2.03–2.13 (m, 1H), 2.46–2.53 (m, 1H), 2.53–2.60 (m, 2H), 2.69–2.78 (m, 1H), 2.83 (t, *J* = 6.4 Hz, 2H), 3.59–3.66 (m, 2H), 4.18 (d, *J* = 5.7 Hz, 2H), 4.68 (ddd, *J* = 5.4, 7.7, 12.6 Hz, 1H), 6.32 (dd, *J* = 2.2, 15.0 Hz, 1H), 6.48 (dd, *J* = 2.2, 8.8 Hz, 1H), 6.82–6.90 (m, 3H), 6.98 (t, *J* = 5.8 Hz, 1H), 7.13–7.18 (m, 2H), 7.50 (t, *J* = 8.9 Hz, 1H), 7.83 (t, *J* = 7.6 Hz, 1H), 10.79 (s, 1H); ^13^C NMR (151 MHz, DMSO-*d*_6_) δ 24.35, 28.43, 29.35, 31.14, 36.26, 40.24, 45.57, 45.74, 48.87, 49.86, 77.52, 97.75 (d, *J* = 27.0 Hz), 108.01 (d, *J* = 12.4 Hz), 108.65, 115.97, 128.27, 128.66, 131.81 (d, *J* = 4.8 Hz), 150.57, 153.38 (d, *J* = 12.4 Hz), 155.94, 161.83 (d, *J* = 245.6 Hz), 163.35, 172.50, 173.10; LC–MS (ESI) (90% H_2_O to 100% MeCN in 10 min, then 100% MeCN to 20 min, DAD 220–600 nm), *t*_R_ = 6.78 min, 94% purity. *m*/*z* [M + H]^+^ calcd for C_30_H_39_FN_5_O_5_, 568.29; found, 568.4. HRMS (ESI) *m*/*z* [M + H]^+^ calcd for C_30_H_39_FN_5_O_5_, 568.2930; found, 568.2926.

#### *tert*-Butyl *N*-[[1-[4-[(2,6-Dioxo-3-piperidyl)carbamoyl]-3-methoxy-phenyl]-4-piperidyl]methyl]carbamate (**41h**)

This compound was prepared using the General Procedure B (2.3 mmol scale) and crude acid **38h** (840 mg). The crude product was purified by CC (3% MeOH in CH_2_Cl_2_) to give a gray solid. Yield 370 mg (34%); mp 236–238 °C; *R*_f_ = 0.35 (5% MeOH in CH_2_Cl_2_); ^1^H NMR (400 MHz, DMSO-*d*_*6*_) δ 1.09–1.25 (m, 3H), 1.38 (s, 9H), 1.52–1.64 (m, 1H), 1.69 (d, *J* = 13.1 Hz, 2H), 2.02–2.17 (m, 2H), 2.71–2.81 (m, 3H), 2.84 (t, *J* = 6.3 Hz, 2H), 3.91 (s, 5H), 4.64–4.75 (m, 1H), 6.51 (d, *J* = 2.3 Hz, 1H), 6.58 (dd, *J* = 9.0, 2.2 Hz, 1H), 6.91 (t, *J* = 5.9 Hz, 1H), 7.76 (d, *J* = 8.9 Hz, 1H), 8.42 (d, *J* = 7.0 Hz, 1H), 10.88 (s, 1H). ^13^C NMR (101 MHz, DMSO-*d*_*6*_) δ 24.44, 28.31, 28.97, 31.09, 36.21, 45.36, 47.25, 50.10, 55.84, 77.45, 97.39, 106.72, 109.87, 132.42, 154.48, 155.84, 159.01, 164.39, 172.76, 173.03; UPLC-retention time, 4.96 min; purity 90%. HRMS (ESI) *m*/*z* [M + H]^+^ calcd for C_24_H_35_N_4_O_6_, 475.2551; found, 475.2550.

#### *tert*-Butyl *N*-[[1-[4-[(2,6-Dioxo-3-piperidyl)carbamoyl]phenyl]-4-piperidyl]methyl]carbamate (**42h**)

This compound was prepared using the General Procedure B (0.5 mmol scale) and crude acid **39h** (167 mg). The crude product was purified by FC (25 g, 15 μm, gradient from 60 to 100% EtOAc in cyclohexane) to give a colorless solid. Yield 76 mg (34%); mp 124–126 °C; *R*_f_ = 0.44 (EtOAc); ^1^H NMR (500 MHz, DMSO-*d*_6_) δ 1.09–1.21 (m, 2H), 1.37 (s, 9H), 1.50–1.63 (m, 1H), 1.64–1.71 (m, 2H), 1.89–2.00 (m, 1H), 2.04–2.16 (m, 1H), 2.51–2.57 (m, 1H), 2.68–2.86 (m, 5H), 3.80–3.88 (m, 2H), 4.68–4.77 (m, 1H), 6.86 (t, *J* = 6.0 Hz, 1H), 6.91–6.97 (m, 2H), 7.69–7.75 (m, 2H), 8.41 (d, *J* = 8.3 Hz, 1H), 10.80 (s, 1H); ^13^C NMR (126 MHz, DMSO) δ 24.52, 28.40, 29.00, 31.14, 36.24, 45.48, 47.41, 49.49, 77.50, 113.68, 122.42, 128.81, 153.02, 155.91, 165.91, 172.60, 173.13; LC–MS (ESI) (90% H_2_O to 100% MeCN in 10 min, then 100% MeCN to 20 min, DAD 220–600 nm), *t*_R_ = 5.51 min, 99% purity. *m*/*z* [M + H]^+^ calcd for C_23_H_33_N_4_O_5_, 445.24; found, 445.6. HRMS (ESI) *m*/*z* [M + H]^+^ calcd for C_23_H_33_N_4_O_5_, 445.2445; found, 445.2447.

#### 4-[2-[[2-[(9*S*)-7-(4-Chlorophenyl)-4,5,13-trimethyl-3-thia-1,8,11,12-tetrazatricyclo[8.3.0.02,6]trideca-2(6),4,7,10,12-pentaen-9-yl]acetyl]amino]ethylamino]-*N*-(2,6-dioxo-3-piperidyl)-2-fluorobenzamide (**43a**)

This compound was prepared using the General Procedure E and linker conjugate **40a** (41 mg). The crude product was purified by FC (25 g, 15 μm, gradient from 0 to 10% MeOH in CH_2_Cl_2_) to give a colorless solid. Yield 51 mg (74%); mp 208–210 °C; *R*_f_ = 0.45 (10% MeOH in CH_2_Cl_2_); ^1^H NMR (500 MHz, DMSO-*d*_6_) δ 1.62 (s, 3H), 1.97–2.06 (m, 1H), 2.03–2.15 (m, 1H), 2.40 (s, 3H), 2.50–2.55 (m, 1H), 2.59 (s, 3H), 2.70–2.80 (m, 1H), 3.14–3.39 (m, 6H), 4.52 (t, *J* = 7.1 Hz, 1H), 4.66–4.74 (m, 1H), 6.39 (dd, *J* = 2.2, 15.1 Hz, 1H), 6.48 (dd, *J* = 2.2, 8.8 Hz, 1H), 6.54 (t, *J* = 5.6 Hz, 1H), 7.36–7.47 (m, 4H), 7.52–7.59 (m, 1H), 7.84 (t, *J* = 7.7 Hz, 1H), 8.33 (t, *J* = 5.8 Hz, 1H), 10.79 (s, 1H); ^13^C NMR (126 MHz, DMSO) δ 11.42, 12.81, 14.17, 24.35, 31.13, 37.86, 37.98, 40.30, 42.37, 49.90, 53.98, 97.48 (d, *J* = 27.6 Hz), 108.12 (d, *J* = 12.7 Hz), 108.46, 128.57, 129.74, 130.00, 130.25, 130.86, 131.98 (d, *J* = 4.7 Hz), 132.40, 135.36, 136.88, 149.98, 153.32 (d, *J* = 12.4 Hz), 155.26, 162.00 (d, *J* = 246.1 Hz), 163.23, 163.28 (d, *J* = 2.8 Hz), 170.19, 172.48, 173.07; LC–MS (ESI) (90% H_2_O to 100% MeCN in 10 min, then 100% MeCN to 20 min, DAD 220–600 nm), *t*_R_ = 5.78 min, 99% purity. *m*/*z* [M + H]^+^ calcd for C_33_H_33_ClFN_8_O_4_S, 691.20; found, 691.4. HRMS (ESI) *m*/*z* [M + H]^+^ calcd for C_33_H_33_ClFN_8_O_4_S, 691.2013; found, 691.2012.

#### 4-[4-[[2-[(9S)-7-(4-Chlorophenyl)-4,5,13-trimethyl-3-thia-1,8,11,12-tetrazatricyclo[8.3.0.02,6]trideca-2(6),4,7,10,12-pentaen-9-yl]acetyl]amino]butylamino]-*N*-(2,6-dioxo-3-piperidyl)-2-fluorobenzamide (**43b**)

This compound was prepared using the General Procedure E and linker conjugate **40b** (44 mg). The crude product was purified by FC (25 g, 15 μm, gradient from 0 to 10% MeOH in CH_2_Cl_2_) to give a colorless solid. Yield 63 mg (88%); mp 216–218 °C; *R*_f_ = 0.47 (10% MeOH in CH_2_Cl_2_); ^1^H NMR (600 MHz, DMSO-*d*_6_) δ 1.49–1.64 (m, 7H), 1.96–2.04 (m, 1H), 2.04–2.14 (m, 1H), 2.39 (s, 3H), 2.52 (d, *J* = 3.7 Hz, 1H), 2.58 (s, 3H), 2.70–2.79 (m, 1H), 3.02–3.28 (m, 6H), 4.50 (dd, *J* = 5.9, 8.3 Hz, 1H), 4.66–4.73 (m, 1H), 6.32 (dd, *J* = 2.2, 15.2 Hz, 1H), 6.44 (dd, *J* = 2.2, 8.8 Hz, 1H), 6.53 (t, *J* = 5.5 Hz, 1H), 7.37–7.45 (m, 2H), 7.42–7.47 (m, 2H), 7.53 (t, *J* = 8.9 Hz, 1H), 7.80 (t, *J* = 7.4 Hz, 1H), 8.20 (t, *J* = 5.7 Hz, 1H), 10.80 (s, 1H); ^13^C NMR (151 MHz, DMSO) δ 11.44, 12.82, 14.16, 24.37, 25.97, 27.02, 31.15, 37.87, 38.31, 40.24, 42.37, 49.89, 54.07, 55.05, 97.19 (d, *J* = 28.1 Hz), 107.55 (d, *J* = 13.0 Hz), 108.28, 128.59, 129.72, 129.98, 130.25, 130.84, 131.95 (d, *J* = 4.6 Hz), 132.41, 135.37, 136.90, 149.95, 153.62 (d, *J* = 12.2 Hz), 155.27, 162.05 (d, *J* = 246.2 Hz), 163.16, 163.34 (d, *J* = 2.8 Hz), 169.59, 172.55, 173.11; LC–MS (ESI) (90% H_2_O to 100% MeCN in 10 min, then 100% MeCN to 20 min, DAD 220–600 nm), *t*_R_ = 5.99 min, 99% purity. *m*/*z* [M + H]^+^ calcd for C_35_H_37_ClFN_8_O_4_S, 719.23; found, 719.5. HRMS (ESI) *m*/*z* [M + H]^+^ calcd for C_35_H_37_ClFN_8_O_4_S, 719.2326; found, 719.2323.

#### 4-[6-[[2-[(9*S*)-7-(4-Chlorophenyl)-4,5,13-trimethyl-3-thia-1,8,11,12-tetrazatricyclo[8.3.0.02,6]trideca-2(6),4,7,10,12-pentaen-9-yl]acetyl]amino]hexylamino]-*N*-(2,6-dioxo-3-piperidyl)-2-fluorobenzamide (**43c**)

This compound was prepared using the General Procedure E and linker conjugate **40c** (46 mg). The crude product was purified by FC (25 g, 15 μm, gradient from 0 to 10% MeOH in CH_2_Cl_2_) to give a colorless solid. Yield 54 mg (72%); mp 194–196 °C; *R*_f_ = 0.51 (10% MeOH in CH_2_Cl_2_); ^1^H NMR (600 MHz, DMSO-*d*_6_) δ 1.30–1.40 (m, 4H), 1.41–1.48 (m, 2H), 1.48–1.56 (m, 2H), 1.60 (s, 3H), 1.97–2.04 (m, 1H), 2.04–2.14 (m, 1H), 2.39 (s, 3H), 2.50–2.53 (m, 1H), 2.58 (s, 3H), 2.70–2.79 (m, 1H), 3.02 (q, *J* = 6.6 Hz, 2H), 3.04–3.17 (m, 2H), 3.15–3.27 (m, 2H), 4.50 (dd, *J* = 6.1, 8.1 Hz, 1H), 4.66–4.73 (m, 1H), 6.30 (dd, *J* = 2.2, 15.2 Hz, 1H), 6.43 (dd, *J* = 2.2, 8.8 Hz, 1H), 6.51 (t, *J* = 5.4 Hz, 1H), 7.38–7.43 (m, 2H), 7.47 (d, *J* = 8.6 Hz, 2H), 7.53 (t, *J* = 8.9 Hz, 1H), 7.81 (t, *J* = 7.7 Hz, 1H), 8.15 (t, *J* = 5.7 Hz, 1H), 10.80 (s, 1H); ^13^C NMR (151 MHz, DMSO) δ 11.43, 12.82, 14.17, 24.37, 26.27, 26.39, 28.52, 29.37, 31.16, 37.85, 38.54, 40.24, 42.53, 49.89, 54.09, 59.90, 97.18 (d, *J* = 28.1 Hz), 107.53 (d, *J* = 12.3 Hz), 108.23, 128.60, 129.72, 129.96, 130.24, 130.85, 131.94 (d, *J* = 4.8 Hz), 132.41, 135.39, 136.92, 149.93, 153.62 (d, *J* = 12.2 Hz), 155.28, 162.03 (d, *J* = 245.5 Hz), 163.14, 163.35, 169.49, 172.55, 173.11; LC–MS (ESI) (90% H_2_O to 100% MeCN in 10 min, then 100% MeCN to 20 min, DAD 220–600 nm), *t*_R_ = 6.49 min, 99% purity. *m*/*z* [M + H]^+^ calcd for C_37_H_41_ClFN_8_O_4_S, 747.26; found, 747.5. HRMS (ESI) *m*/*z* [M + H]^+^ calcd for C_37_H_41_ClFN_8_O_4_S, 747.2639; found, 747.2636.

#### 4-[8-[[2-[(9*S*)-7-(4-Chlorophenyl)-4,5,13-trimethyl-3-thia-1,8,11,12-tetrazatricyclo[8.3.0.02,6]trideca-2(6),4,7,10,12-pentaen-9-yl]acetyl]amino]octylamino]-*N*-(2,6-dioxo-3-piperidyl)-2-fluorobenzamide (**43d**)

This compound was prepared using the General Procedure E and linker conjugate **40d** (49 mg). The crude product was purified by FC (25 g, 15 μm, gradient from 0 to 10% MeOH in CH_2_Cl_2_) to give a colorless solid. Yield 28 mg (36%); mp 220–224 °C; *R*_f_ = 0.25 (7% MeOH in CH_2_Cl_2_); ^1^H NMR (600 MHz, DMSO-*d*_6_) δ 1.20–1.38 (m, 9H), 1.39–1.47 (m, 2H), 1.47–1.55 (m, 2H), 1.61 (s, 3H), 1.97–2.04 (m, 1H), 2.04–2.14 (m, 1H), 2.39 (s, 3H), 2.58 (s, 3H), 2.70–2.79 (m, 1H), 2.98–3.27 (m, 6H), 4.49 (dd, *J* = 5.9, 8.2 Hz, 1H), 4.66–4.73 (m, 1H), 6.29 (dd, *J* = 2.2, 15.1 Hz, 1H), 6.42 (dd, *J* = 2.1, 8.7 Hz, 1H), 6.50 (t, *J* = 5.4 Hz, 1H), 7.41 (d, *J* = 8.3 Hz, 2H), 7.47 (d, *J* = 8.4 Hz, 2H), 7.53 (t, *J* = 8.9 Hz, 1H), 7.81 (t, *J* = 7.7 Hz, 1H), 8.13 (t, *J* = 5.7 Hz, 1H), 10.80 (s, 1H); ^13^C NMR (151 MHz, DMSO) δ 11.43, 12.82, 14.18, 24.37, 26.51, 26.70, 28.57, 28.94, 28.99, 29.41, 31.16, 37.85, 38.59, 40.24, 42.59, 49.89, 54.10, 97.16 (d, *J* = 27.4 Hz), 107.53 (d, *J* = 13.0 Hz), 108.23, 128.58, 129.73, 129.95, 130.27, 130.87, 131.93 (d, *J* = 4.6 Hz), 132.41, 135.39, 136.90, 149.93, 153.62 (d, *J* = 12.3 Hz), 155.29, 162.02 (d, *J* = 245.6 Hz), 163.12, 163.36, 169.47, 172.56, 173.11; LC–MS (ESI) (90% H_2_O to 100% MeCN in 10 min, then 100% MeCN to 20 min, DAD 200–600 nm), *t*_R_ = 6.94 min, 98% purity. *m*/*z* [M + H]^+^ calcd for C_39_H_45_ClFN_8_O_4_S, 775.30; found, 775.4. HRMS (ESI) *m*/*z* [M + H]^+^ calcd for C_39_H_45_ClFN_8_O_4_S, 775.2952; found, 775.2936.

#### 4-[10-[[2-[(9*S*)-7-(4-Chlorophenyl)-4,5,13-trimethyl-3-thia-1,8,11,12-tetrazatricyclo[8.3.0.02,6]trideca-2(6),4,7,10,12-pentaen-9-yl]acetyl]amino]decylamino]-*N*-(2,6-dioxo-3-piperidyl)-2-fluorobenzamide (**43e**)

This compound was prepared using the General Procedure E and linker conjugate **40e** (52 mg). The crude product was purified by FC (25 g, 15 μm, gradient from 0 to 8% MeOH in CH_2_Cl_2_) to give a colorless solid. Yield 58 mg (73%); mp 180–182 °C; *R*_f_ = 0.60 (10% MeOH in CH_2_Cl_2_); ^1^H NMR (600 MHz, DMSO-*d*_6_) δ 1.17–1.37 (m, 12H), 1.39–1.46 (m, 2H), 1.47–1.55 (m, 2H), 1.61 (s, 3H), 1.96–2.04 (m, 1H), 2.04–2.14 (m, 1H), 2.39 (s, 3H), 2.50–2.53 (m, 1H), 2.58 (s, 3H), 2.70–2.79 (m, 1H), 2.98–3.19 (m, 5H), 3.24 (dd, *J* = 8.4, 14.9 Hz, 1H), 4.49 (dd, *J* = 5.7, 8.4 Hz, 1H), 4.69 (ddd, *J* = 5.4, 7.3, 12.7 Hz, 1H), 6.29 (dd, *J* = 2.2, 15.1 Hz, 1H), 6.42 (dd, *J* = 2.2, 8.8 Hz, 1H), 6.50 (t, *J* = 5.4 Hz, 1H), 7.41 (d, *J* = 8.7 Hz, 2H), 7.46 (d, *J* = 8.8 Hz, 2H), 7.53 (t, *J* = 8.9 Hz, 1H), 7.81 (t, *J* = 7.7 Hz, 1H), 8.13 (t, *J* = 5.7 Hz, 1H), 10.80 (s, 1H); ^13^C NMR (151 MHz, DMSO) δ 11.43, 12.81, 14.18, 24.37, 26.57, 26.70, 28.56, 29.00, 29.18, 29.44, 31.15, 37.86, 38.60, 40.24, 42.58, 49.89, 54.11, 55.05, 97.15 (d, *J* = 28.4 Hz), 107.51 (d, *J* = 12.3 Hz), 108.23, 128.57, 129.73, 129.94, 130.26, 130.87, 131.93 (d, *J* = 4.5 Hz), 132.42, 135.39, 136.88, 149.92, 153.62 (d, *J* = 12.2 Hz), 155.29, 162.02 (d, *J* = 245.6 Hz), 163.09, 163.35, 169.47, 172.55, 173.11; LC–MS (ESI) (90% H_2_O to 100% MeCN in 10 min, then 100% MeCN to 20 min, DAD 220–600 nm), *t*_R_ = 7.69 min, 97% purity. *m*/*z* [M + H]^+^ calcd for C_41_H_49_ClFN_8_O_4_S, 803.33; found, 803.5. HRMS (ESI) *m*/*z* [M + H]^+^ calcd for C_41_H_49_ClFN_8_O_4_S, 803.3265; found, 803.3261.

#### 4-[2-[2-[[2-[(9*S*)-7-(4-Chlorophenyl)-4,5,13-trimethyl-3-thia-1,8,11,12-tetrazatricyclo[8.3.0.02,6]trideca-2(6),4,7,10,12-pentaen-9-yl]acetyl]amino]ethoxy]ethylamino]-*N*-(2,6-dioxo-3-piperidyl)-2-fluorobenzamide (**43f**)

This compound was prepared using the General Procedure E and linker conjugate **40g** (45 mg). The crude product was purified by FC (25 g, 15 μm, gradient from 0 to 10% MeOH in CH_2_Cl_2_) to give a colorless solid. Yield 68 mg (92%); mp 178–180 °C; *R*_f_ = 0.52 (10% MeOH in CH_2_Cl_2_); ^1^H NMR (500 MHz, DMSO-*d*_6_) δ 1.56 (s, 3H), 1.96–2.15 (m, 2H), 2.38 (s, 3H), 2.50–2.56 (m, 1H), 2.58 (s, 3H), 2.69–2.80 (m, 1H), 3.22 (dd, *J* = 5.3, 10.3 Hz, 3H), 3.22–3.29 (m, 2H), 3.29–3.41 (m, 1H), 3.44–3.54 (m, 2H), 3.54–3.64 (m, 2H), 4.51 (dd, *J* = 6.1, 8.1 Hz, 1H), 4.65–4.74 (m, 1H), 6.36 (dd, *J* = 2.2, 15.1 Hz, 1H), 6.47 (dd, *J* = 2.1, 8.8 Hz, 1H), 6.51 (t, *J* = 5.4 Hz, 1H), 7.38–7.45 (m, 2H), 7.45 (d, *J* = 8.7 Hz, 2H), 7.52 (t, *J* = 8.9 Hz, 1H), 7.81 (t, *J* = 7.7 Hz, 1H), 8.27 (t, *J* = 5.7 Hz, 1H), 10.79 (s, 1H); ^13^C NMR (126 MHz, DMSO) δ 11.42, 12.77, 14.10, 24.35, 31.13, 37.79, 38.59, 40.29, 42.53, 49.89, 54.02, 68.52, 69.35, 97.45 (d, *J* = 27.7 Hz), 107.89 (d, *J* = 12.7 Hz), 108.33, 128.55, 129.68, 129.96, 130.28, 130.80, 131.91 (d, *J* = 4.8 Hz), 132.38, 135.34, 136.94, 149.93, 153.43 (d, *J* = 12.4 Hz), 155.23, 161.96 (d, *J* = 245.9 Hz), 163.19, 163.31 (d, *J* = 3.0 Hz), 170.00, 172.50, 173.07; LC–MS (ESI) (90% H_2_O to 100% MeCN in 10 min, then 100% MeCN to 20 min, DAD 220–600 nm), *t*_R_ = 5.89 min, 99% purity. *m*/*z* [M + H]^+^ calcd for C_35_H_37_ClFN_8_O_5_S, 735.23; found, 735.5. HRMS (ESI) *m*/*z* [M + H]^+^ calcd for C_35_H_37_ClFN_8_O_5_S, 735.2275; found, 735.2272.

#### 4-[2-[2-[2-[[2-[(9*S*)-7-(4-Chlorophenyl)-4,5,13-trimethyl-3-thia-1,8,11,12-tetrazatricyclo[8.3.0.02,6]trideca-2(6),4,7,10,12-pentaen-9-yl]acetyl]amino]ethoxy]ethoxy]ethylamino]-*N*-(2,6-dioxo-3-piperidyl)-2-fluorobenzamide (**43g**)

This compound was prepared using the General Procedure E and linker conjugate **40g** (50 mg). The crude product was purified by FC (25 g, 15 μm, gradient from 0 to 10% MeOH in CH_2_Cl_2_) to give a colorless solid. Yield 67 mg (85%); mp 158–160 °C; *R*_f_ = 0.58 (10% MeOH in CH_2_Cl_2_); ^1^H NMR (600 MHz, DMSO-*d*_6_) δ 1.60 (s, 3H), 1.97–2.04 (m, 1H), 2.04–2.14 (m, 1H), 2.39 (s, 3H), 2.50–2.54 (m, 1H), 2.58 (s, 3H), 2.70–2.79 (m, 1H), 3.18–3.30 (m, 6H), 3.46 (t, *J* = 5.9 Hz, 2H), 3.56 (s, 5H), 3.56 (d, *J* = 11.3 Hz, 1H), 4.50 (dd, *J* = 6.1, 8.0 Hz, 1H), 4.66–4.73 (m, 1H), 6.37 (dd, *J* = 2.3, 15.0 Hz, 1H), 6.47 (dd, *J* = 2.2, 8.7 Hz, 1H), 6.54 (t, *J* = 5.6 Hz, 1H), 7.41 (d, *J* = 8.3 Hz, 2H), 7.47 (d, *J* = 8.4 Hz, 2H), 7.52 (t, *J* = 8.9 Hz, 1H), 7.83 (t, *J* = 7.6 Hz, 1H), 8.25 (t, *J* = 5.7 Hz, 1H), 10.80 (s, 1H); ^13^C NMR (151 MHz, DMSO) δ 11.44, 12.82, 14.18, 24.36, 31.15, 37.70, 38.79, 40.24, 42.54, 49.89, 53.99, 55.05, 68.96, 69.37, 69.77, 69.88, 97.48 (d, *J* = 27.7 Hz), 107.89 (d, *J* = 12.2 Hz), 108.36, 128.60, 129.71, 129.98, 130.30, 130.84, 131.92 (d, *J* = 5.0 Hz), 132.42, 135.37, 136.93, 149.95, 153.44 (d, *J* = 12.2 Hz), 155.26, 161.96 (d, *J* = 246.3 Hz), 163.16, 163.34, 169.85, 172.53, 173.11; LC–MS (ESI) (90% H_2_O to 100% MeCN in 10 min, then 100% MeCN to 20 min, DAD 220–600 nm), *t*_R_ = 5.97 min, 99% purity. *m*/*z* [M + H]^+^ calcd for C_37_H_41_ClFN_8_O_6_S, 779.25; found, 779.4. HRMS (ESI) *m*/*z* [M + H]^+^ calcd for C_37_H_41_ClFN_8_O_6_S, 779.2537; found, 779.2535.

#### 4-[4-[[[2-[(9*S*)-7-(4-Chlorophenyl)-4,5,13-trimethyl-3-thia-1,8,11,12-tetrazatricyclo[8.3.0.02,6]trideca-2(6),4,7,10,12-pentaen-9-yl]acetyl]amino]methyl]-1-piperidyl]-*N*-(2,6-dioxo-3-piperidyl)-2-fluorobenzamide (**43h**)

This compound was prepared using the General Procedure E and linker conjugate **40h** (41 mg). The crude product was purified by FC (25 g, 15 μm, gradient from 2 to 12% MeOH in CH_2_Cl_2_) to give a colorless solid. Yield 52 mg (69%); mp 210–212 °C; *R*_f_ = 0.34 (10% MeOH in CH_2_Cl_2_); ^1^H NMR (500 MHz, DMSO-*d*_6_) δ 1.13–1.28 (m, 4H), 1.62 (s, 3H), 1.71–1.78 (m, 2H), 1.95–2.18 (m, 2H), 2.40 (s, 3H), 2.52–2.55 (m, 1H), 2.59 (s, 3H), 2.70–2.84 (m, 3H), 2.97–3.11 (m, 2H), 3.15–3.23 (m, 1H), 3.88 (d, *J* = 13.0 Hz, 2H), 4.51 (t, *J* = 7.2 Hz, 1H), 4.67–4.76 (m, 1H), 6.73 (d, *J* = 16.0 Hz, 1H), 6.79 (d, *J* = 9.1 Hz, 1H), 7.41 (d, *J* = 8.3 Hz, 2H), 7.47 (d, *J* = 8.3 Hz, 2H), 7.61 (t, *J* = 9.1 Hz, 1H), 7.96 (t, *J* = 7.5 Hz, 1H), 8.21 (d, *J* = 6.2 Hz, 1H), 10.80 (s, 1H); ^13^C NMR (126 MHz, DMSO) δ 11.41, 12.80, 14.16, 24.29, 28.96, 31.11, 35.95, 37.86, 40.20, 44.02, 47.09, 49.88, 54.08, 100.58 (d, *J* = 28.0 Hz), 109.60 (d, *J* = 12.8 Hz), 109.91, 128.60, 129.73, 129.96, 130.19, 130.83, 131.80 (d, *J* = 4.6 Hz), 132.38, 135.37, 136.87, 149.91, 154.35 (d, *J* = 11.2 Hz), 155.26, 161.65 (d, *J* = 246.2 Hz), 163.15, 169.71, 172.39, 173.06; LC–MS (ESI) (90% H_2_O to 100% MeCN in 10 min, then 100% MeCN to 20 min, DAD 220–600 nm), *t*_R_ = 6.16 min, 95% purity. *m*/*z* [M + H]^+^ calcd for C_37_H_39_ClFN_8_O_4_S, 745.25; found, 745.5. HRMS (ESI) *m*/*z* [M + H]^+^ calcd for C_37_H_39_ClFN_8_O_4_S, 745.2482; found, 745.2470.

#### 4-[3-[2-[(9S)-7-(4-Chlorophenyl)-4,5,13-trimethyl-3-thia-1,8,11,12-tetrazatricyclo[8.3.0.02,6]trideca-2(6),4,7,10,12-pentaen-9-yl]acetyl]-3,9-diazaspiro[5.5]undecan-9-yl]-*N*-(2,6-dioxo-3-piperidyl)-2-fluorobenzamide (**43j**)

0 mg). The crude product was purified by FC (25 g, 15 μm, gradient from 0 to 10% MeOH in CH_2_Cl_2_) to give a colorless solid. Yield 67 mg (85%); mp >250 °C; *R*_f_ = 0.52 (10% MeOH in CH_2_Cl_2_); ^1^H NMR (600 MHz, DMSO-*d*_6_) δ 1.34–1.61 (m, 8H), 1.62 (s, 3H), 1.96–2.05 (m, 1H), 2.06–2.16 (m, 1H), 2.41 (s, 3H), 2.50–2.54 (m, 1H), 2.59 (s, 3H), 2.71–2.80 (m, 1H), 3.32–3.41 (m, 5H), 3.43–3.54 (m, 2H), 3.57–3.66 (m, 3H), 4.58 (t, *J* = 6.7 Hz, 1H), 4.72 (ddd, *J* = 5.9, 7.9, 12.7 Hz, 1H), 6.75 (dd, *J* = 2.3, 15.9 Hz, 1H), 6.81 (dd, *J* = 2.4, 9.1 Hz, 1H), 7.41–7.46 (m, 2H), 7.46–7.50 (m, 2H), 7.62 (t, *J* = 9.0 Hz, 1H), 7.98 (t, *J* = 7.4 Hz, 1H), 10.81 (s, 1H); ^13^C NMR (151 MHz, DMSO) δ 11.42, 12.84, 14.17, 29.95, 31.15, 34.22, 34.39, 34.79, 34.93, 35.51, 37.22, 40.24, 41.09, 42.75, 49.89, 54.41, 100.37 (d, *J* = 27.9 Hz), 109.55 (d, *J* = 12.5 Hz), 109.70, 128.62, 129.81, 130.03, 130.30, 130.83, 131.82 (d, *J* = 4.8 Hz), 132.35, 135.34, 136.95, 149.88, 154.41 (d, *J* = 11.1 Hz), 155.49, 161.67 (d, *J* = 245.7 Hz), 162.98, 163.19, 168.04, 172.44, 173.11; LC–MS (ESI) (90% H_2_O to 100% MeCN in 10 min, then 100% MeCN to 20 min, DAD 220–600 nm), *t*_R_ = 6.90 min, 99% purity. *m*/*z* [M + H]^+^ calcd for C_40_H_43_ClFN_8_O_4_S, 727.26; found, 785.5. HRMS (ESI) *m*/*z* [M + H]^+^ calcd for C_40_H_43_ClFN_8_O_4_S, 785.2795; found, 785.2794.

#### 4-[[4-[4-[[[2-[(9*S*)-7-(4-Chlorophenyl)-4,5,13-trimethyl-3-thia-1,8,11,12-tetrazatricyclo[8.3.0.02,6]trideca-2(6),4,7,10,12-pentaen-9-yl]acetyl]amino]methyl]-1-piperidyl]phenyl]methylamino]-*N*-(2,6-dioxo-3-piperidyl)-2-fluorobenzamide (**43k**)

This compound was prepared using the General Procedure E and linker conjugate **40k** (57 mg). The crude product was purified by FC (25 g, 15 μm, gradient from 0 to 5% MeOH in CH_2_Cl_2_) to give a colorless solid. Yield 24 mg (31%); mp 210–214 °C; *R*_f_ = 0.36 (5% MeOH in CH_2_Cl_2_); ^1^H NMR (600 MHz, DMSO-*d*_6_) δ 1.19–1.30 (m, 2H), 1.61 (s, 3H), 1.71–1.77 (m, 2H), 1.96–2.13 (m, 2H), 2.07 (s, 3H), 2.40 (s, 3H), 2.50–2.52 (m, 1H), 2.58 (s, 3H), 2.69–2.79 (m, 1H), 3.04 (dh, *J* = 6.2, 25.6 Hz, 2H), 3.18 (dd, *J* = 5.7, 14.9 Hz, 1H), 3.25–3.30 (m, 1H), 3.60–3.67 (m, 2H), 4.19 (d, *J* = 5.8 Hz, 2H), 4.51 (dd, *J* = 5.6, 8.5 Hz, 1H), 4.65–4.72 (m, 1H), 6.33 (dd, *J* = 2.2, 14.9 Hz, 1H), 6.48 (dd, *J* = 2.2, 8.7 Hz, 1H), 6.88 (d, *J* = 8.4 Hz, 2H), 6.99 (t, *J* = 5.8 Hz, 1H), 7.17 (d, *J* = 8.3 Hz, 2H), 7.41 (d, *J* = 8.4 Hz, 2H), 7.45 (d, *J* = 8.7 Hz, 2H), 7.51 (t, *J* = 8.9 Hz, 1H), 7.83 (t, *J* = 7.5 Hz, 1H), 8.21 (t, *J* = 5.9 Hz, 1H), 10.79 (s, 1H); ^13^C NMR (151 MHz, DMSO) δ 11.44, 12.83, 14.17, 24.35, 29.46, 30.83, 31.14, 36.02, 37.89, 40.24, 44.15, 45.74, 48.89, 48.98, 49.87, 54.13, 97.76 (d, *J* = 27.5 Hz), 108.01 (d, *J* = 12.3 Hz), 108.66, 116.01, 128.26, 128.61, 128.76, 129.75, 129.97, 130.24, 130.87, 131.82 (d, *J* = 4.6 Hz), 132.41, 135.39, 136.89, 149.94, 150.60, 153.39 (d, *J* = 12.7 Hz), 155.29, 161.83 (d, *J* = 245.5 Hz), 163.17, 163.29–163.39 (m), 169.72, 172.50, 173.10; LC–MS (ESI) (90% H_2_O to 100% MeCN in 10 min, then 100% MeCN to 20 min, DAD 220–600 nm), *t*_R_ = 6.88 min, 97% purity. *m*/*z* [M + H]^+^ calcd for C_44_H_46_ClFN_9_O_4_S, 850.31; found, 850.5. HRMS (ESI) *m*/*z* [M + H]^+^ calcd for C_44_H_46_ClFN_9_O_4_S, 850.3061; found, 850.3050.

#### 4-[4-[[[2-[(9*S*)-7-(4-Chlorophenyl)-4,5,13-trimethyl-3-thia-1,8,11,12-tetrazatricyclo[8.3.0.02,6]trideca-2(6),4,7,10,12-pentaen-9-yl]acetyl]amino]methyl]-1-piperidyl]-*N*-(2,6-dioxo-3-piperidyl)-2-methoxybenzamide (**44h**)

This compound was prepared using the General Procedure E and linker conjugate **41h** (66 mg). The crude product was purified by CC (CH_2_Cl_2_/MeOH 15:1) to give a white solid. Yield 60 mg (63%); mp 174–175 °C; *R*_f_ = 0.38 (10% MeOH in CH_2_Cl_2_); ^1^H NMR (400 MHz, DMSO-*d*_*6*_) δ 1.23 (s, 2H), 1.60–1.64 (m, 3H), 1.68 (s, 1H), 1.77 (d, *J* = 12.8 Hz, 2H), 2.01–2.17 (m, 2H), 2.39–2.43 (m, 3H), 2.60 (s, 3H), 2.78 (q, *J* = 11.4 Hz, 3H), 2.96–3.14 (m, 2H), 3.15–3.32 (m, 2H), 3.92 (s, 5H), 4.52 (dd, *J* = 8.5, 5.7 Hz, 1H), 4.63–4.75 (m, 1H), 6.52 (d, *J* = 2.2 Hz, 1H), 6.59 (dd, *J* = 9.0, 2.2 Hz, 1H), 7.34–7.53 (m, 4H), 7.77 (d, *J* = 8.8 Hz, 1H), 8.28 (t, *J* = 5.9 Hz, 1H), 8.43 (d, *J* = 7.0 Hz, 1H), 10.88 (s, 1H); ^13^C NMR (101 MHz, CDCl_3_) δ 11.95, 13.23, 14.51, 25.46, 29.53, 31.48, 36.24, 39.64, 45.00, 48.11, 51.55, 53.56, 54.68, 55.94, 97.44, 107.73, 110.37, 128.85, 129.94, 130.58, 131.03, 131.07, 132.20, 133.43, 136.65, 136.99, 150.06, 155.20, 155.79, 159.51, 164.11, 165.88, 170.81, 171.92, 172.19; UPLC-retention time, 6.37 min; purity 96%. HRMS (ESI) *m*/*z* [M + H]^+^ calcd for C_38_H_42_ClN_8_O_5_S, 757.2682; found, 757.2675.

#### 4-[4-[[[2-[(9*S*)-7-(4-Chlorophenyl)-4,5,13-trimethyl-3-thia-1,8,11,12-tetrazatricyclo[8.3.0.02,6]trideca-2(6),4,7,10,12-pentaen-9-yl]acetyl]amino]methyl]-1-piperidyl]-*N*-(2,6-dioxo-3-piperidyl)benzamide (**45h**)

This compound was prepared using the General Procedure E and linker conjugate **42h** (44 mg). The crude product was purified by FC (25 g, 15 μm, gradient from 0 to 7% MeOH in CH_2_Cl_2_) to give a colorless solid. Yield 52 mg (72%); mp 222–226 °C; *R*_f_ = 0.22 (7% MeOH in CH_2_Cl_2_); ^1^H NMR (600 MHz, DMSO-*d*_6_) δ1.16–1.28 (m, 2H), 1.62 (s, 3H), 1.62–1.71 (m, 1H), 1.76 (d, *J* = 12.7 Hz, 2H), 1.90–1.99 (m, 1H), 2.05–2.15 (m, 1H), 2.40 (s, 3H), 2.50–2.56 (m, 1H), 2.59 (s, 3H), 2.71–2.82 (m, 3H), 2.98–3.11 (m, 2H), 3.16–3.23 (m, 1H), 3.25–3.31 (m, 1H), 3.83–3.88 (m, 2H), 4.51 (dd, *J* = 5.7, 8.4 Hz, 1H), 4.69–4.77 (m, 1H), 6.95 (d, *J* = 8.7 Hz, 2H), 7.42 (d, *J* = 8.5 Hz, 2H), 7.46 (d, *J* = 8.8 Hz, 2H), 7.73 (d, *J* = 8.6 Hz, 2H), 8.23 (t, *J* = 5.9 Hz, 1H), 8.41 (d, *J* = 8.3 Hz, 1H), 10.79 (s, 1H); ^13^C NMR (151 MHz, DMSO) δ 11.44, 12.83, 14.18, 24.56, 29.15, 31.19, 36.04, 37.88, 40.24, 44.12, 47.45, 47.50, 49.50, 54.12, 113.76, 122.49, 128.62, 128.85, 129.75, 129.98, 130.23, 130.86, 132.41, 135.40, 136.90, 149.94, 153.09, 155.29, 163.18, 165.92, 169.73, 172.68, 173.22; LC–MS (ESI) (90% H_2_O to 100% MeCN in 10 min, then 100% MeCN to 20 min, DAD 220–600 nm), *t*_R_ = 5.96 min, 99% purity. *m*/*z* [M + H]^+^ calcd for C_37_H_40_ClN_8_O_4_S, 727.26; found, 727.5. HRMS (ESI) *m*/*z* [M + H]^+^ calcd for C_37_H_40_ClN_8_O_4_S, 727.2576; found, 727.2582.

#### *tert*-Butyl *N*-[[1-(4-Cyanophenyl)-4-piperidyl]methyl]carbamate (**46**)

4-Fluorobenzonitrile (0.61 g, 5.0 mmol), *tert*-butyl (piperidin-4-ylmethyl)carbamate (1.07 g, 5.0 mmol), and DIPEA (1.74 mL, 10 mmol) were dissolved in dry DMSO (50 mL) and heated under an argon atmosphere at 90 °C for 16 h. After cooling, it was diluted with half-saturated NH_4_Cl solution (200 mL) and extracted with EtOAc (2 × 200 mL). The combined organic layers were washed with 5% LiCl solution, half-saturated NH_4_Cl solution, and brine (each 200 mL). The solution was then dried over Na_2_SO_4_, filtered, and concentrated *in vacuo*. The crude product was purified by FC (40 g, 30 μm, gradient from 10 to 50% EtOAc in cyclohexane) to give a colorless solid. Yield 0.81 g (51%); *R*_f_ = 0.65 (50% EtOAc in cyclohexane); mp 136–138 °C; ^1^H NMR (500 MHz, DMSO-*d*_6_) δ 1.05–1.17 (m, 2H), 1.37 (s, 9H), 1.55–1.70 (m, 3H), 2.76–2.85 (m, 4H), 3.85–3.93 (m, 2H), 6.84 (t, *J* = 6.0 Hz, 1H), 6.94–7.01 (m, 2H), 7.48–7.55 (m, 2H); ^13^C NMR (126 MHz, DMSO-*d*_6_) δ 28.40, 28.83, 36.20, 45.36, 46.68, 77.52, 97.39, 114.09, 120.27, 133.44, 153.10, 155.91.; LC–MS (ESI) (90% H_2_O to 100% MeCN in 10 min, then 100% MeCN to 20 min, DAD 220–600 nm), *t*_R_ = 7.15 min, 99% purity. *m*/*z* [M + H]^+^ calcd for C_18_H_26_N_3_O_2_, 316.20; found, 316.2.

#### *tert*-Butyl 2-[4-[(2,6-Dioxo-3-piperidyl)carbamoyl]-3-fluoroanilino]acetate (**47**)

This compound was prepared using the General Procedure D and *tert*-butyl 2-aminoacetate (1.18 g). The crude product was purified by FC (80 g, 15 μm, gradient from 0 to 10% MeOH in CH_2_Cl_2_) to give a colorless solid. This crude intermediate was subjected to the General Procedure B (0.5 mmol scale). The crude product was purified by FC (25 g, 15 μm, gradient from 50 to 100% EtOAc in cyclohexane) to give a light blue solid. Yield 0.15 g (79%); mp 196–200 °C; *R*_f_ = 0.67 (EtOAc); ^1^H NMR (500 MHz, DMSO-*d*_6_) δ 1.41 (s, 9H), 1.96–2.04 (m, 1H), 2.01–2.15 (m, 1H), 2.39–2.55 (m, 1H), 2.75 (ddd, *J* = 5.6, 13.4, 17.4 Hz, 1H), 3.86 (d, *J* = 6.3 Hz, 2H), 4.65–4.74 (m, 1H), 6.34 (dd, *J* = 2.2, 14.6 Hz, 1H), 6.46 (dd, *J* = 2.2, 8.8 Hz, 1H), 6.78 (t, *J* = 6.1 Hz, 1H), 7.54 (t, *J* = 8.8 Hz, 1H), 7.90 (t, *J* = 7.4 Hz, 1H), 10.79 (s, 1H); ^13^C NMR (126 MHz, DMSO) δ 24.32, 27.86, 31.12, 45.12, 49.86, 81.01, 98.06 (d, *J* = 27.5 Hz), 108.53, 108.88 (d, *J* = 12.7 Hz), 131.79 (d, *J* = 4.5 Hz), 153.00 (d, *J* = 12.3 Hz), 161.67 (d, *J* = 246.1 Hz), 163.32, 169.77, 172.43, 173.07; LC–MS (ESI) (90% H_2_O to 100% MeCN in 10 min, then 100% MeCN to 20 min, DAD 220–600 nm), *t*_R_ = 5.08 min, 98% purity. *m*/*z* [M + H]^+^ calcd for C_18_H_23_FN_3_O_5_, 380.16; found, 380.2. HRMS (ESI) *m*/*z* [M + H]^+^ calcd for C_18_H_23_FN_3_O_5_, 380.1616; found, 380.1610.

#### 2-[4-[(2,6-Dioxo-3-piperidyl)carbamoyl]-3-methoxyanilino]acetic Acid (**48**)

To a solution of compound **11d** (1.42 g, 4.53 mmol), glyoxylic acid monohydrate (0.63 g, 6.80 mmol), and sodium acetate (0.74 g, 9.06 mmol) in dry MeOH (60 mL) was added under an argon atmosphere acetic acid (1.09 g, 1.04 mL, 18.12 mmol). The mixture was cooled to 0 °C and NaCNBH_3_ (0.31 g, 4.98 mmol) was added portion-wise. After stirring at 0 °C for 1 h, the mixture was filtered over a pad of silica and washed with 1% AcOH in EtOAc (100 mL). The volatiles were evaporated, EtOAc (100 mL) was added and then washed with brine (100 mL), dried over Na_2_SO_4_, filtered, and concentrated *in vacuo*. The crude product was purified by column chromatography (CH_2_Cl_2_/MeOH 2:1) to give a beige solid. Yield 435 mg (29%); mp 206–209 °C; *R*_f_ = 0.28 (CH_2_Cl_2_/MeOH 2:1); ^1^H NMR (400 MHz, DMSO-*d*_6_) δ 1.96–2.19 (m, 2H), 2.51–2.53 (m, 1H), 2.66–2.81 (m, 1H), 3.38 (d, *J* = 3.8 Hz, 2H), 3.86 (s, 3H), 4.62–4.73 (m, 1H), 5.93 (t, *J* = 4.4 Hz, 1H), 6.19 (dd, *J* = 8.7, 2.0 Hz, 1H), 6.26 (d, *J* = 2.0 Hz, 1H), 7.68 (d, *J* = 8.6 Hz, 1H), 8.35 (d, *J* = 6.9 Hz, 1H), 10.87 (s, 1H); ^13^C NMR (101 MHz, DMSO-*d*_6_) δ 24.56, 31.11, 47.31, 50.09, 55.56, 94.65, 104.45, 107.68, 132.61, 152.63, 159.24, 164.72, 172.89, 173.02; LC–MS (ESI) (90% H_2_O to 100% MeCN in 10 min, then 100% MeCN to 20 min, DAD 220–600 nm), *t*_R_ = 0.40 min, 98% purity. *m*/*z* [M + H]^+^ calcd for C_15_H_18_N_3_O_6_, 336.12; found, 336.1. HRMS (ESI) *m*/*z* [M + H]^+^ calcd for C_15_H_18_N_3_O_6_, 336.1190; found, 336.1188.

#### Preloaded Resin (**49**)

The synthesis was performed according to our previously published protocol,^60^ and starting from the preloaded resin HAIR D (loading 0.674–0.678 mmol/g).^61^

#### *N*-(2,6-Dioxo-3-piperidyl)-2-fluoro-4-[[2-[[8-[4-[[7-(hydroxyamino)-7-oxo-heptyl]carbamoyl]anilino]-8-oxooctyl]amino]-2-oxoethyl]amino]benzamide (**50**)

After swelling of the preloaded resin **49** (560 mg, 0.30 mmol), the Fmoc-deprotection was performed by treatment with 20% piperidine in DMF (2 × 3 mL for 5 min). Afterward, the resin was washed with DMF (5 × 5 mL), MeOH (5 × 5 mL), and DMF (5 × 5 mL). In parallel the free carboxylic acid derivative of **47** (194 mg, 0.30 mmol, obtained in quantitative yield from the corresponding *tert*-butyl ester **47** by treatment with 50% TFA in CH_2_Cl_2_), HATU (229 mg, 0.60 mmol), HOBt × H_2_O (114 mg, 0.60 mmol) and DIPEA (157 μL, 0.89 mmol) were dissolved in DMF (2.5 mL) and stirred for 5 min. The activated carboxylic acid was added to the resin and the mixture was shaken for 18 h. Subsequently, the resin was washed with DMF (5 × 5 mL) and CH_2_Cl_2_ (10 × 5 mL). A TNBS-test (Catalog# T2024, TCI, Tokyo, Japan) and a test cleavage were performed to verify the completion of the amide coupling. PROTAC **50** was cleaved from the resin by treatment with TFA and triisopropylsilane (each 5% in CH_2_Cl_2_) for 1 h and dried in vacuo. For each 60 mg of resin, 1.0 mL of the cleavage cocktail was used. The crude product was dissolved in DMSO/acetone (1:9 (*v*/*v*)) and purified by preparative HPLC to yield **50** as an amorphous white powder. Yield (over 7 steps from HAIR D): 32.3 mg (19%); ^1^H NMR (600 MHz, DMSO-*d*_6_) δ 1.21–1.32 (m, 11H), 1.35–1.43 (m, 2H), 1.46–1.52 (m, 4H), 1.53–1.61 (m, 2H), 1.94 (t, *J* = 7.4 Hz, 2H), 1.97–2.04 (m, 1H), 2.06–2.14 (m, 1H), 2.31 (t, *J* = 7.4 Hz, 2H), 2.51–2.54 (m, 1H)*, 2.75 (ddd, *J* = 13.4, 17.2 Hz, 1H), 3.05–3.10 (m, 2H), 3.19–3.24 (m, 2H), 3.69 (s, 2H), 4.67–4.74 (m, 1H), 6.31 (dd, *J* = 2.2, 14.7 Hz, 1H), 6.45 (dd, *J* = 2.2, 14.7 Hz, 1H), 7.55 (t, *J* = 8.8 Hz, 1H), 7.64 (d, *J* = 8.5 Hz, 2H), 7.77 (d, *J* = 8.6 Hz, 2H), 7.90 (t, *J* = 7.5 Hz, 1H), 7.93 (t, *J* = 5.8 Hz, 1H), 8.26 (t, *J* = 5.6 Hz, 1H), 10.03 (s, 1H), 10.31 (s, 1H), 10.81 (s, 1H), *overlapping with DMSO signal, C–N*H*–OH signal could not be detected due to solvent exchange. ^13^C NMR (151 MHz, DMSO-*d*_6_) δ 24.16, 24.93, 25.06, 26.21, 28.33, 28.47, 28.60, 29.03, 29.06, 30.96, 32.22, 36.41, 38.43, 40.06, 46.03, 49.71, 97.84 (d, *J* = 27.5 Hz), 108.44, 108.52 (d, *J* = 12.6 Hz), 118.07, 127.88, 128.93, 131.64 (d, *J* = 4.6 Hz), 141.69, 152.96 (d, *J* = 12.2 Hz), 161.54 (d, *J* = 246.1 Hz), 163.15 (d, *J* = 2.9 Hz), 165.53, 168.87, 169.08, 171.58, 172.30, 172.93; HPLC (95% A for 5 min equilibration, from 95% A to 95% B in 12 and 15 min isocratic) *t*_R_ = 10.71 min, 99% purity. HRMS (ESI) *m*/*z* [M + H]^+^ calcd for C_36_H_49_FN_7_O_8_, 726.3621; found, 726.3621.

#### *N*-(2,6-Dioxo-3-piperidyl)-4-[[2-[[8-[4-[[7-(hydroxyamino)-7-oxo-heptyl]carbamoyl] anilino]-8-oxooctyl]amino]-2-oxoethyl]amino]-2-methoxybenzamide (**51**)

After swelling of the preloaded resin **49** (350 mg, 0.20 mmol, 1.00 equiv), the Fmoc-deprotection was performed by treatment with 20% piperidine in DMF (2 × 3 mL for 5 min). Afterward, the resin was washed with DMF (5 × 5 mL), MeOH (5 × 5 mL), and DMF (5 × 5 mL). In parallel the free carboxylic acid **48** (138 mg, 0.41 mmol), HATU (156 mg, 0.041 mmol), HOBt × H_2_O (78 mg, 0.41 mmol), EDC × HCl (79 mg, 0.41 mmol) and DIPEA (180 μL, 1.03 mmol) were dissolved in DMF (2.4 mL) and stirred for 5 min. The activated carboxylic acid was added to the resin and the mixture was shaken for 18 h. Subsequently, the resin was washed with DMF (5 × 5 mL) and CH_2_Cl_2_ (10 × 5 mL). A TNBS-test (Catalog# T2024, TCI, Tokyo, Japan) and a test cleavage were performed to verify the completion of the amide coupling. **51** was cleaved from the resin by treatment with TFA and triisopropylsilane (each 5% in CH_2_Cl_2_) for 1 h and dried in vacuo. For each 60 mg of resin, 1.0 mL of the cleavage cocktail was used. The crude product was dissolved in DMSO/acetone (1:9 (*v*/*v*)) and purified by preparative HPLC to yield **51** as amorphous white powder. Yield (over 7 steps from HAIR D): 41.0 mg (28%); ^1^H NMR (600 MHz, DMSO-*d*_6_) δ 1.18–1.34 (m, 11H), 1.36–1.42 (m, 2H), 1.45–1.52 (m, 4H), 1.54–1.60 (m, 2H), 1.94 (t, *J* = 7.4 Hz, 2H), 1.99–2.08 (m, 1H), 2.10–2.17 (m, 1H), 2.30 (t, *J* = 7.4 Hz, 2H), 2.50–2.54 (m, 1H)*, 2.71–2.79 (m, 1H), 3.05–3.10 (m, 2H), 3.19–3.24 (m, 2H), 3.70 (s, 2H), 3.85 (s, 3H), 4.64–4.72 (m, 1H), 6.21 (dd, *J* = 2.1, 8.7z Hz, 1H), 6.25 (d, *J* = 2.1 Hz, 1H), 7.64 (d, *J* = 8.7 Hz, 2H), 7.70 (d, *J* = 8.6 Hz, 1H), 7.77 (d, *J* = 8.7 Hz, 2H), 7.90 (t, *J* = 5.8 Hz, 1H), 8.27 (t, *J* = 5.6 Hz, 1H), 8.36 (d, *J* = 6.9 Hz, 1H), 10.03 (s, 1H), 10.31 (s, 1H), 10.83 (s, 1H), *overlapping with DMSO signal, C–N*H*–OH signal could not be detected due to solvent exchange.; ^13^C NMR (151 MHz, DMSO-*d*_6_) δ 24.46, 24.94, 25.07, 25.46, 26.22, 28.34, 28.49, 28.61, 29.07, 29.09, 31.04, 32.22, 36.41, 38.42, 40.06, 46.20, 50.08, 55.53, 95.00, 104.58, 108.91, 118.07, 127.89, 128.93, 132.52, 141.70, 152.76, 159.02, 164.53, 165.54, 169.08, 169.19, 171.58, 172.72, 172.91; HPLC (95% A for 5 min equilibration, from 95% A to 95% B in 15 and 5 min isocratic) *t*_R_ = 14.46 min; 96% purity. HRMS (ESI) *m*/*z* [M + H]^+^ calcd for C_37_H_52_N_7_O_9_, 737.3821; found, 737.3821.

#### *N*-(2,6-Dioxo-3-piperidyl)pyridine-2-carboxamide (**52**)

This compound was prepared using the General Procedure B (2 mmol scale) and 2-picolinic acid (0.25 g). The crude product was purified by FC (40 g, 15 μm, gradient from 60 to 100% EtOAc in cyclohexane) to give a light blue solid. Yield 0.10 g (22%); mp 186–190 °C; *R*_f_ = 0.39 (EtOAc); ^1^H NMR (500 MHz, DMSO-*d*_6_) δ 1.96–2.06 (m, 1H), 2.15–2.28 (m, 1H), 2.52–2.57 (m, 1H), 2.74–2.85 (m, 1H), 4.74–4.83 (m, 1H), 7.60–7.66 (m, 1H), 7.98–8.04 (m, 1H), 8.06 (d, *J* = 7.6 Hz, 1H), 8.64–8.69 (m, 1H), 9.06 (d, *J* = 8.3 Hz, 1H), 10.85 (s, 1H); ^13^C NMR (126 MHz, DMSO) δ 24.08, 31.10, 49.63, 122.16, 126.89, 137.99, 148.60, 149.60, 163.96, 172.17, 173.02; LC–MS (ESI) (90% H_2_O to 100% MeCN in 10 min, then 100% MeCN to 20 min, DAD 220–600 nm), *t*_R_ = 1.34 min, 99% purity. *m*/*z* [M + H]^+^ calcd for C_11_H_12_N_3_O_3_, 234.09; found, 234.1.

#### 2-Acetamido-*N*-(2,6-dioxo-3-piperidyl)thiophene-3-carboxamide (**53**)

This compound was prepared using the General Procedure B (2 mmol scale) and compound **56** (0.37 g). The crude product was purified by FC (80 g, 30 μm, gradient from 60 to 100% EtOAc in cyclohexane) to give a light blue solid. Yield 69 mg (12%); mp 212–214 °C; *R*_f_ = 0.44 (EtOAc); ^1^H NMR (500 MHz, DMSO-*d*_6_) δ 1.90–1.98 (m, 1H), 2.10 (s, 3H), 2.11–2.23 (m, 1H), 2.50–2.59 (m, 1H), 2.71–2.82 (m, 1H), 4.70 (ddd, *J* = 5.3, 8.1, 13.0 Hz, 1H), 7.73 (d, *J* = 5.4 Hz, 1H), 7.92 (d, *J* = 5.3 Hz, 1H), 8.52 (d, *J* = 8.2 Hz, 1H), 10.86 (s, 1H), 10.86 (s, 1H); ^13^C NMR (126 MHz, DMSO) δ 24.04, 24.23, 31.08, 49.53, 113.06, 122.21, 129.30, 142.75, 163.56, 167.34, 172.01, 173.02; LC–MS (ESI) (90% H_2_O to 100% MeCN in 10 min, then 100% MeCN to 20 min, DAD 220–600 nm), *t*_R_ = 2.61 min, 99% purity. *m*/*z* [M + H]^+^ calcd for C_12_H_14_N_3_O_4_S, 296.07; found, 296.1.

#### *N*-(2,6-Dioxo-3-piperidyl)-2-methylbenzofuran-7-carboxamide (**54**)

This compound was prepared using the General Procedure B (1 mmol scale) and compound **59** (0.29 g). The crude product was purified by FC (40 g, 30 μm, gradient from 60 to 100% EtOAc in cyclohexane) to give a light blue solid. Yield 0.16 g (55%); mp 244–248 °C; *R*_f_ = 0.56 (EtOAc); ^1^H NMR (500 MHz, DMSO-*d*_6_) δ 2.09–2.17 (m, 1H), 2.14–2.25 (m, 1H), 2.52–2.60 (m, 1H), 2.76–2.87 (m, 1H), 4.78–4.87 (m, 1H), 6.67–6.71 (m, 1H), 7.29 (t, *J* = 7.7 Hz, 1H), 7.64–7.73 (m, 2H), 8.50 (d, *J* = 7.6 Hz, 1H), 10.89 (s, 1H); ^13^C NMR (126 MHz, DMSO) δ 13.84, 24.21, 31.08, 50.14, 103.04, 117.74, 122.73, 123.75, 123.79, 129.99, 151.06, 156.24, 163.73, 172.20, 173.00; LC–MS (ESI) (90% H_2_O to 100% MeCN in 10 min, then 100% MeCN to 20 min, DAD 220–600 nm), *t*_R_ = 4.52 min, 98% purity. *m*/*z* [M + H]^+^ calcd for C_15_H_15_N_2_O_4_, 287.10; found, 287.2.

#### Conformational Analyses of CRBN Ligands

For each compound, a 3D structure of the

*S*-stereoisomer was first generated using LigPrep (Schrödinger Suite 2020–2, Schrödinger, LLC, New York, NY, 2020). Structures were then minimized using MacroModel (Schrödinger Suite 2020–2, Schrödinger, LLC, New York, NY, 2020) and subjected to coordinate scan in the OPLS3e force field. The dihedral angle between the phenyl and the amide bond was selected.

### Biochemistry

#### Microscale Thermophoresis

MST measurements and data analysis were performed as previously described using the human thalidomide binding domain (hTBD).^24^ In brief, a 16-point 1:1 dilution series of the compound in DMSO was diluted 1:100 in ddH_2_O and then mixed with protein:reporter stock to final concentrations of 10 μM hTBD and 200 nM BODIPY-uracil. Measurements were performed on a Monolith NT.115 with a Nano BLUE detector (NanoTemper Technologies), using 20% excitation power, MST power set to medium and temperature control at 25 °C. The obtained normalized fluorescence (F_norm_) values at an MST on-time of 20 s were baseline-corrected to the mean of the normalized fluorescence values for the lowest concentration of ligand (ΔF_norm_), plotted against the compound concentration and fitted to a nonlinear four-parameter equation using GraphPad Prism 9. Errors correspond to the symmetrical 95% confidence interval. Conversion of IC_50_ values to *K*_i_ values was performed as described previously.^74^*K*_i_ error values were calculated as the difference between the *K*_i_ and a theoretical *K*_i_ calculated from the boundary of the IC_50_ 95% confidence interval.

#### Crystallization

For structural studies, we employed the previously described crystal soaking system based on the hTBD homolog MsCI4.^44^ In brief, MsCI4 was concentrated to 21 mg/mL and cocrystallized with 3 mM thalidomide at (NH_4_)H_2_PO_4_ concentrations between 0.4 to 0.6 M. For soaking experiments, crystals were transferred to a droplet of fresh reservoir solution spiked with individual compounds to final concentrations of 3.3 mM and 3.3% DMSO. After soaking times of 48 h, crystals were cryoprotected with the addition of 70% sodium malonate and flash-cooled in liquid nitrogen. Diffraction data were collected using an EIGER2 16 M detector (DECTRIS) at the Swiss Light Source beamline X10SA at 100 K. Data were processed and scaled with XDS,^75^ and R_free_ sets imported from PDB 6R18. The structures were solved using difference Fourier methods and PDB 4V2Y as a starting template. Structures were completed using cyclic rounds of refinement in REFMAC5,^76^ and modeling in Coot.^77^ Figures were created using PyMOL (Schrödinger, LLC.). Data collection and refinement statistics are specified in Tables S2 and S3. Coordinates and structure factors were deposited in the Protein Data Bank (PDB) under the accession codes 8OU3 (**8d**), 8OU4 (**11a**), 8OU5 (**11b**), 8OU6 (**11c**), 8OU7 (**11d**), 8OU9 (**11e**), 8OUA (**11f**).

#### UV–Vis-Based Stability Screening

The aqueous stability of compounds in Tables 2 and 3 was determined spectrophotometrically by following the changes in the absorption spectra of the compounds, as described previously.^56,78^ Briefly, the final mixture contained: 10 mM sodium phosphate, pH 7.0, 8.0, or 9.0, and 50 μM of the tested compound. The final concentration of DMSO was 5% (V/V). The mixtures were incubated in 96-well UV-transparent microplates (CLS3635, Corning, USA) without lids at 37 °C using Synergy H4 microplate reader (BioTek Instruments, Inc., USA) and the absorbance spectrum (244–400 nm) was acquired in sweep mode immediately after preparing solutions and after 240 min using. A blank experiment was performed without compound and the obtained baseline was subtracted from each measurement. Compounds with an absorbance maximum of less than 0.2 AU were assigned a low absorbance flag and were not evaluated due to the high experimental error in this assay. For other compounds, the relative absorbance difference between the first time point and 240 min at the most responsive wavelength was calculated. If the relative absorbance difference for the compound in the buffer was above 0.2, the compound was classified as unstable.

### Redox Activity Assays

Assays were performed according to previously optimized procedures in 96-well microplates in assay buffer (50 mM HEPES, 50 mM NaCl, pH 7.5).^49^ Threshold values for activity flags were above 10-fold standard deviation compared to DMSO blanks. All reagent solutions were freshly prepared before performing the experiments.

3-Methyltoxoflavin was used as a control compound. To exclude spectral interference, absorbance at the excitation and emission wavelengths and autofluorescence were determined for all compounds in buffer solution.

#### H_2_DCFDA Assay for the Detection of ROS

First, the probe H_2_DCFDA was dissolved in DMSO and diluted to 500 μM with 0.01 M NaOH. The obtained solution was incubated for 30 min in the dark at room temperature to hydrolyze the ester. To 52.5 μL of assay buffer were added 7.5 μL of 2 mM compound DMSO stock solution, 75 μL of assay buffer (redox-free) or 75 μL of 200 μM TCEP in assay buffer, and 15 μL of 500 μM H_2_DCFDA. The final concentrations were 100 μM compound, 50 μM H_2_DCFDA, 100 μM TCEP, and 5% (V/V) DMSO. The microplate was covered with a lid and incubated for 30 min in the dark at room temperature. Fluorescence intensity was then measured using Synergy H4 microplate reader (BioTek Instruments, Inc., USA) at λ_ex_ = 485 nm and λ_em_ = 535 nm. In a blank experiment, the compound solution was replaced with pure DMSO. The measured fluorescence for each compound was then divided by the blank value.

#### Resazurin Assay for the Detection of Free Radical

Briefly, to 100 μL of assay buffer were added 2 μL of 1, 0.1, or 0.01 mM compound DMSO stock solution and 100 μL of resazurin solution (10 μM resazurin and 200 μM DTT in assay buffer). The final concentrations were 10, 1, or 0.1 μM compound, 5 μM resazurin, 100 μM DTT, and 1% (V/V) DMSO. The microplate was covered with a lid and incubated for 30 min in the dark at room temperature. Fluorescence intensity was then measured using Synergy H4 microplate reader (BioTek Instruments, Inc., USA) at λ_ex_ = 560 nm and λ_em_ = 590 nm. In a blank experiment, the compound solution was replaced with pure DMSO. The measured fluorescence for each compound was then divided by the blank value.

### Determination of Physicochemical Properties

#### log D_7.4_ Measurements

The determination of the log *D*_7.4_ values was performed by a chromatographic method as described previously.^79^ Briefly, the system was calibrated by plotting the retention times of six different drugs (atenolol, metoprolol, labetalol, diltiazem, triphenylene, permethrin) versus their literature known log *D*_7.4_ values to obtain a calibration line (*R*^2^ ≥ 0.95). Subsequently, the mean retention times of the analytes were taken to calculate their log *D*_7.4_ values with aid of the calibration line.

#### Plasma Protein Binding Studies

CHIRALPAK HSA 50 × 3 mm, 5 μm column with the literature known %PPB values (converted into log *K* values) of the following drugs: warfarin, ketoprofen, budesonide, nizatidine, indomethacin, acetylsalicylic acid, carbamazepine, piroxicam, nicardipine, and cimetidine. Samples were dissolved in MeCN/DMSO 9:1 to achieve a final concentration of 0.5 mg/mL. The mobile phase A was 50 mM NH_4_Ac adjusted to pH 7.4 with ammonia, while mobile phase B was *i*PrOH. The flow rate was set to 1.0 mL/min, the UV detector was set to 254 nm, and the column temperature was kept at 30 °C. After injecting 3 μL of the sample, a linear gradient from 100% A to 30% *i*PrOH in 5.4 min was applied. From 5.4 to 18 min, 30% *i*PrOH was kept, followed by switching back to 100% A in 1.0 min and a re-equilibration time of 6 min. With the aid of the calibration line (*R*^2^ ≥ 0.92), the log *K* values of new substances were calculated and converted to their %PPB values.

#### IAM Chromatography

Drug–membrane interactions were assessed and characterized by a high-throughput HPLC method on an IAM column that consists of monolayers of phospholipids covalently bound to silica particles.^80^ In detail, the column was a Regis IAM.PC.DD2 column (100 × 4.6 mm, 10 μm, 300 Å) equipped with a guard cartridge. The column oven was set to 25 °C. Mobile phase A was 50 mM NH_4_Ac adjusted to pH 7.4 with ammonia, while mobile phase B was MeCN. The retention times were measured with a gradient of 0 to 95% MeCN from 0 to 6 min, which was kept at 95% until 6.5 min, then dropped to 0% from 6.5 to 7 min, and finally kept at 0% until 9 min. The mobile phase flow rate was 1.5 mL/min. For the conversion of gradient retention times to chromatographic hydrophobicity index values referring to IAM chromatography (CHI_IAM_ values), a calibration was performed by plotting the retention times of an IAM standard solution of paracetamol, acetanilide, acetophenone, propiophenone, butyrophenone, valerophenone and octanophenone against their literature known CHI values.^80^

#### Compound Solubility

Compound solubility was determined by the shake flask method. About 1 mg of the analyte was weighed into a Eppendorff tube and shaken at 500 rpm and 25 °C for 1 h with 1 mL buffer pH 6.8 (25 mM PBS). After 1 h, the suspensions were centrifuged at 15.000 rpm for 10 min, aliquots of 450 μL of the supernatants were removed, and diluted with MeCN (50 μL) to prevent precipitation of the dissolved compounds. Accordingly, the observed solubility result was multiplied by 500/450 to account for the MeCN addition in the test filtrate. The concentrations of the compounds were determined by HPLC with UV detection at 270 nm unless indicated otherwise. Eluent A consisted of 0.1% TFA in water, eluent B consisted of 0.1% TFA in MeCN. Compounds were eluted in gradient mode on a Poroshell 120 EC-C18 3 × 50 mm, 2.7 μm column within 12 min and starting at 20% B. The injection volume was 20 μL and the flow rate was 0.7 mL/min. The calibration curve was obtained by plotting the peak areas vs diluted concentrations of reference solutions (at least 6 data points). The analyte was accurately weighted and a DMSO stock solution of 1 mg/mL was prepared. Diluted MeCN solutions between 1 and 100 μg/mL were prepared and analyzed by HPLC. The values represent the mean of at least two independent experiments.

#### Compound Stability Measurements

The HPLC-based assay was performed by analogy with our previously reported method.^14^ In brief, 5 mM compound solutions in MeCN were mixed with the indicated buffer (20:80 v/v), incubated at 37 °C, and the internal standard CST530 was used. At the time points 0, 24, and 48 h, sample solutions were injected to HPLC and compound stability was calculated as the percentage of initial compound after integrating the area under curve (AUC).

#### Compound Stability in Human Plasma

The determination of the *in vitro* plasma stability was performed by a chromatographic method as described before.^14^ In brief, the human plasma was diluted to 80% with 0.05 M PBS (pH 7.4). Test compounds were prepared as 2 mM stock solutions in DMSO. Preheated (37 °C) plasma (570 μL) was mixed with compound solution (30 μL) to yield a final concentration of 100 μM with a DMSO content of 5%. Each plasma sample was divided into aliquots of 100 μL that were incubated at 37 °C. At each stability time point (0, 15, 30, 60, and 120 min), the samples were quenched by the addition of MeOH (200 μL) containing internal standard and 2% AcOH. After quenching, samples were vortexed for 1 min, stored at 0 °C for at least 15 min to complete precipitation, and then centrifuged at 15000 rpm at 4 °C for 2 min. The clear supernatants were filtered through a 0.45 μm PTFE membrane filter, and the filtrates were analyzed by HPLC. For each test compound injection, the response ratio (test peak area/internal standard peak area) was calculated and converted to percentage test compound remaining. The % test compound remaining is plotted versus time. The values represent the mean of three independent experiments. Procaine hydrochloride was used as a positive control compound to demonstrate sufficient activity of the plasma. The *in vitro* plasma half-life (*t*_1/2_) was calculated using the expression *t*_1/2_ = 0.693/*b*, where *b* is the slope found in the linear fit of the natural logarithm of the fraction remaining of the parent compound versus incubation time.

#### Metabolic Stability in Human Liver Microsomes

Assays were performed in 96-deep-well-plates (“incubation plate”). Experiments were conducted on a horizontal shaker with a fitted heating block (“Thermomixer”, Eppendorf). Test and reference items stock solutions were prepared in DMSO or ACN, respectively. The test item stock solutions were further diluted in DMSO/H_2_O (1:1, v/v), and the verapamil stock solution was diluted in ACN to obtain working solutions of 100-fold higher strength than the intended final test concentration in experimental incubations (1 μM in the presence of 0.5% DMSO for test items, 1% ACN for verapamil). The assay was performed using human liver microsomes at 0.5 mg/mL. The incubation solutions (final volume: 700 μL) consisted of 350 μL of a microsomal suspension (1 mg/mL protein; i.e., final 0.5 mg/mL in incubation) in reaction buffer (potassium phosphate buffer 50 mM, pH 7.4, supplemented with 3 mM MgCl_2_ and 1 mM EDTA), 273 μL reaction buffer, and 7 μL of test item or positive control working solution, respectively. Components were pipetted into the respective wells of the “incubation plate” in the order given above and prewarmed on a horizontal shaker equipped with a fitted heating block. For the test item, two wells were prepared as reaction wells, and two wells were prepared as negative control wells. The experiment was initiated by the addition of 70 μL of a 10 mM NADPH solution in reaction buffer to the prewarmed (37 °C) microsomes/buffer/test item mix to facilitate Phase I metabolism. 70 μL samples were removed from the incubations after 0, 10, 30, and 60 min and transferred to the “quenching plate” for sample preparation containing ACN supplemented with the internal standards (ISTD, 1 μM diazepam, 1 μM griseofulvin, and 10 μM diclofenac). Verapamil was used as high clearance positive control in order to demonstrate the microsomal CYP enzyme activity. Incubations containing verapamil were run in parallel with test item incubations. Samples were drawn after 0 and 30 min. Microsomal metabolic activity was assessed in terms of verapamil turnover, i.e., loss of verapamil. Negative controls using microsome without NADPH were run in parallel to experimental incubations to verify that any apparent loss of test item in the assay incubation was due to metabolism. For negative controls, 70 μL assay buffer instead of 70 μL NADPH solution was added to the negative control incubations. All incubations were run in duplicates (*n* = 2). All incubations were stopped and precipitated by addition of 2 volumes of ACN containing the internal standards. After vigorously shaking (10 s) the samples were centrifuged (2200*g*) for 5 min at room temperature. Aliquots of the particle-free supernatants were diluted with an equal volume of H_2_O and subsequently subjected to LC/MS analysis.

#### Molecular Descriptor Calculations

Predicted values for the topological polar surface area (TPSA) were calculated using MarvinSketch 17.28.0 (ChemAxon).

### Cell Biology

#### Cell Culture and Cell Treatment

Cell lines were obtained from the American Type Culture Collection (ATCC) and Deutsche Sammlung von Mikroorganismen und Zellkulturen (DSMZ). Cells were cultured in RPMI-1640 (Thermo Scientific) or Dulbecco’s Modified Eagle’s Medium (DMEM, Thermo Scientific) supplemented with 10% fetal bovine serum (FBS; Life Technologies) and 1% penicillin/streptomycin (Life Technologies). The multiple myeloma cell line MM.1S was additionally supplemented with 1 mM pyruvate. The HuH6 cell line was maintained in DMEM. Cells were maintained in a 37 °C humidified incubator with 5% CO_2_ and regularly tested for mycoplasma. Cells (3 × 10^6^ cells/mL) were seeded in cell culture plates or flasks and treated with the respective compounds or vehicle (DMSO) for the indicated time.

#### Immunoblotting

Cells were washed and lysed in Pierce IP lysis buffer supplemented protease and phosphatase inhibitors. Protein concentration was determined by Pierce BCA Protein Assay Kit (Catalog# 23225, Thermo Fisher Scientific Inc., Waltham, MA, USA) according to the manufacturer’s guidelines. Samples were denatured by Laemmli 2 × concentrate (Catalog# S3401-10VL, Sigma-Aldrich, St. Louis, MO, USA), and Precision Plus Kaleidoscope Protein Unstained Standard was used as molecular weight marker (Catalog# 1610375, Bio-Rad, Hercules, CA, USA). SDS-PAGE was performed with 20 μg protein per sample using Mini-PROTEAN Tetra Cell System (Catalog# 1658001EDU Bio-Rad Hercules, CA, USA). Following separation, the proteins were transferred using Trans-Blot Turbo transfer System (Catalog# 1704150, Bio-Rad) onto PVDF membranes. The membranes were washed with TBST and blocked in 5% BSA solution for 1 h at room temperature. Subsequently, the membranes were incubated with anti-BRD4 (Catalog# 13440S, Cell Signaling Technology, Denver, MA, USA), anti-BRD3 (Catalog# PA5-40885, Invitrogen, USA), anti-BRD2 (Catalog# 5848S, Cell Signaling Technology), anti-GSPT1 (Catalog# 14980, Cell Signaling Technology), anti-IKZF3 (Catalog# 15103, Cell Signaling Technology), anti-IKZF1 (Catalog# 14859S, Cell Signaling Technology), anti-CK1a (Catalog# sc6477, Santa Cruz, USA), anti-SALL4 (Catalog# ab57577, Abcam, USA), anti-α-Tubulin (Catalog# T5168, Sigma, USA), and ß-actin (HRP) (Catalog# ab20272, Abcam, USA) antibody solutions in 1:1000–1:5000 dilutions at 4 °C overnight. Incubation with HRP-conjugated secondary antirabbit HRP-conjugated antibody (Catalog# 7074, Cell Signaling Technology) was performed in a 5% Milk solution for 1 h at room temperature. Chemiluminescence signal was detected using Immobilon Western Chemiluminescent HRP Substrate (Millipore) and imaged using LAS 4000× (Fuji). Quantification of blots was performed using ImageJ software. HDAC6 immunoblotting was performed as follows. Cell lysis was performed with Cell Extraction Buffer (10 mM Tris, pH 7.4, 100 mM NaCl, 1 mM EDTA, 1 mM EGTA, 1 mM NaF, 20 mM Na_4_P_2_O_7_, 2 mM Na_3_VO_4_, 1% TritonX-100, 10% glycerol, 0.1% SDS, 0.5% deoxycholate; Catalog# FNN0011, Thermo Fisher Scientific Inc., Waltham, MA, USA) and addition of Halt Protease Inhibitor Cocktail (100×) (Catalog# 78429, Life Technologies GmbH, Carlsbad, CA, USA) and phenylmethanesulfonyl fluoride (Catalog# 10837091001, Sigma-Aldrich, St. Louis, MO, USA) according to the manufacturer’s protocol. Protein content was determined by Pierce BCA Protein Assay Kit (Catalog# 23225, Thermo Fisher Scientific Inc., Waltham, MA, USA) according to the manufacturer’s guidelines. Samples were denatured by Laemmli 2× concentrate (Catalog# S3401-10VL, Sigma-Aldrich, St. Louis, MO, USA), and Precision Plus Protein Unstained Standard was used as molecular weight marker (Catalog# 1610363, Bio-Rad, Hercules, CA, USA). SDS-PAGE was performed with 10% Mini-PROTEAN TGX Stain-Free Gel (Catalog# 458035, Bio-Rad, Hercules, CA, USA) at 200 V for 50 min (Catalog# 458035, Bio-Rad). Afterward, proteins were transferred with the Trans-Blot Turbo Transfer System (Catalog# 1704150, Bio-Rad, Hercules, CA, USA) to Immobilon-FL PVDF membranes (Catalog# IPFL00005, Millipore Merck, Burlington, MA, USA) at 1.0 A for 30 min and incubated with 5% milk-powder solution for 1 h at room temperature under slight agitation. Subsequently, the membranes were incubated with anti-BRD4 (Catalog# 13440S, Cell Signaling Technology, Denver, MA, USA), anti-HDAC1 (Catalog# 5356S, Cell Signaling Technology, Denver, MA, USA), anti-HDAC6 (Catalog# 7558S, Cell Signaling Technology, Denver, MA, USA), anti-GAPDH (Catalog# T0004, Affinity Biosciences, Cincinnati, OH, USA) and ß-actin (HRP) (Catalog# ab20272, Abcam, USA) antibody solutions in 1:1000–1:20000 dilutions at 4 °C overnight. Incubation with HRP-conjugated secondary antimouse (Catalog# sc-516102, Santa Cruz, Dallas, TX, USA) and antirabbit (Catalog# HAF008, R&D Systems, Inc., Minneapolis, MN, USA) antibody solution was performed for 1.5 h, and membranes were developed with clarity western ECL substrate (Catalog# 1705061, Bio-Rad, Hercules, CA, USA). A ChemiDoc XRS+ System (Catalog# 1708265, Bio-Rad, Hercules, CA, USA) was used for detection and Image Lab Software 6.1 (Bio-Rad, Hercules, CA, USA) for analyses. Immunoblots were performed in three independent experiments.

#### IKZF3 Reporter System

HEK293T cells were seeded in 6-well plate in 2 mL of DMEM (10% FCS, 1% Pen-Strep) and incubated at 37 °C overnight. The following day, the transfection mix containing 1 μg Aiolis in Artichoke (Addgene Plasmid #74452), 1 μg psPAX2 (Addgene Plasmid #12260), 200 ng pMD2.G (Addgene Plasmid #12259), 6 μL TranslT-LT1 Transfection Reagent (Mirus MIR2300), and 250 mL Opti-MEM (ThermoFischer 31985062) was prepared and incubated at room temperature for 30 min. The transfection mix was added dropwise to the cells followed by incubation at 37 °C overnight. Virus was harvested after 36 h using a syringe containing a 0.45 μm filter. Molt4 and MM1.S cells were transduced in 24 well plates using desired number of cells and virus with Polybrene (Sigma-Aldrich TR-1003) at a final concentration of 8 μg/mL. Spinfection was performed at 1000 RCF for 1 h at 32 °C. After 48 h, the cells were selected using 1 μg/mL puromycin for 72 h. For the IKZF3 reporter assay, MOLT4 and MM.1S cells were seeded in 96 well plates and treated with the mentioned compounds for 4 or 24 h. Cells were washed with PBS and resuspended in DAPI/PBS solution. Cells were measured on Cytoflex (Beckman Coulter) flow cytometer. Live-single cells expressing mCherry were gated for FITC and histograms displaying Counts were plotted and quantified.

## Supporting Information Available

^1^H-^13^C HMBC NMR spectrum, torsion angle scans, correlation between physiochemical properties, cell staining, dose-response curves (Figures S1–S10); syntheses (Scheme S1); sub van der Waals distances, X-ray data, crystal data, (Tables S1–S3); synthetic procedures for compounds **55**–**59**, selected ^1^H and ^13^C NMR spectra, LC/MS data, HPLC trace (PDF)
Molecular formula strings (CSV)

## Accession Codes

Atomic coordinates and structure factors have been deposited in the Protein Data Bank. Accession codes of the hTBD homolog MsCI4 in complex with benzamides are as follows: **8d** (PDB 8OU3), **11a** (PDB 8OU4), **11b** (PDB 8OU5), **11c** (PDB 8OU6), **11d** (PDB 8OU7), **11e** (PDB 8OU9), **11f** (PDB 8OUA).

## ABBREVIATIONS USED

BRD4: bromodomain-containing protein 4
CC: column chromatography
CHI: chromatographic hydrophobicity index
CK1α: casein kinase 1α
CRBN: cereblon E3 ligase
DMEM: Dulbecco’s Modified Eagle’s Medium
FC: flash chromatography
FRET: Förster resonance energy transfer
GAPDH: glyceraldehyde 3-phosphate
GSPT1: G1 to S Phase Transition 1 protein
HBD: hydrogen bond donor
H_2_DCFDA: 2′,7′-dichloro-dihydrofluorescein diacetate
HDAC: histone deacetylase
HOESY: heteronuclear Overhauser effect spectroscopy
hTBD: human CRBN thalidomide binding domain
IMiDs: immunomodulatory drugs
IMHB: intramolecular hydrogen bond
LLE: lipophilic ligand efficiency
MG: molecular glue
MST: microscale thermophoresis
NOE: nuclear Overhauser effect
PEG: polyethylene glycol
POI: protein of interest
PPB: plasma protein binding
PROTACs: proteolysis-targeting chimeras
ROS: reactive oxygen species
SALL4: spalt-like transcription factor 4
S_N_Ar: nucleophilic aromatic substitution
TCEP: tris(2-carboxyethyl)phosphine
TPD: targeted protein degradation
USP7: ubiquitin-specific protease 7
