# Supplementary material for: Leveraging Ligand Affinity and Properties: Discovery of Novel Benzamide-Type Cereblon Binders for the Design of PROTACs
Source: J Med Chem. 2023 Oct 30;66(21):14513–43. doi: 10.1021/acs.jmedchem.3c00851 (PMC10641816; doi:10.1021/acs.jmedchem.3c00851)
Supplement: Supplementary file 1 — jm3c00851_si_001.pdf [file jm3c00851_si_001.pdf]

SUPPORTING INFORMATION

Leveraging Ligand Affinity and Properties: Discovery of Novel Benzamide-Type Cereblon Binders for the Design of PROTACs

Christian Steinebach,[a,#] Aleša Bricelj,[b,#] Arunima Murgai,[c,#] Izidor Sosič,[b] Luca Bischof,[d] Yuen Lam Dora Ng,[c] Christopher Heim,[d] Samuel Maiwald,[d] Matic Proj,[b] Rabea Voget,[a] Felix Feller,[a] Janez Košmrlj,[e] Valeriia Sapozhnikova,[c,f,g] Annika Schmidt,[c] Maximilian Rudolf Zuleeg,[c] Patricia Lemnitzer,[c] Philipp Mertins,[f,h] Finn K. Hansen,[a] Michael Gütschow,[a] Jan Krönke,[c,g,]* and Marcus D. Hartmann[d,i,]*

[a] Pharmaceutical Institute, University of Bonn, D-53121 Bonn, Germany

[b] Faculty of Pharmacy, University of Ljubljana, SI-1000 Ljubljana, Slovenia

[c] Department of Hematology, Oncology, and Cancer Immunology, Charité - Universitätsmedizin Berlin, corporate member of Freie Universität Berlin, Humboldt-Universität zu Berlin, D-12203 Berlin, Germany

[d] Max Planck Institute for Biology Tübingen, D-72076 Tübingen, Germany

[e] Faculty of Chemistry and Chemical Technology, University of Ljubljana, SI 1000 Ljubljana, Slovenia

[f] Max Delbrück Center for Molecular Medicine, D-13125 Berlin, Germany

[g] German Cancer Consortium (DKTK), Partner Site Berlin, DKFZ, D-69120 Heidelberg, Germany

[h] Berlin Institute of Health, D-10178 Berlin, Germany

[i] Interfaculty Institute of Biochemistry, University of Tübingen, D-72076 Tübingen, Germany

# These authors contributed equally

Corresponding Authors

* Email: jan.kroenke@charite.de (J.Kr.);

* Email: marcus.hartmann@tuebingen.mpg.de (M.D.H.)

Table of Content

Supporting Figures, Schemes, and Tables 3

Figure S1. Section of the ^1H-^{13}C HMBC NMR spectrum of **14** in DMSO-d_6. 3

Figure S2. Torsion angle scans of compounds **8c**, **8d**, and **11a-f**. 4

Figure S3. Correlation between physiochemical properties of **8c**, **8d** and **11** 5

Figure S4. Neosubstrate modulation by benzamides **11** and established IMiDs. 6

Figure S5. Effects of linker-connected CRBN ligands **15-23** on neosubstrates 6

Figure S6. Evaluation of BRD4 PROTACs **43-45** in MV4;11 cells. 7

Figure S7. Concentration dependent analysis of BRD4 degradation by PROTACs. 8

Figure S8. Additional physiochemical properties of selected compounds. 9

Figure S9. Proteins regulated by PROTAC **44h**. 10

Figure S10. Dose-response curves for patent structures **52-54** 10

Scheme S1. Synthesis of comparators **52-54**. 11

Table S1. Sub-van der Waals distances observed for the crystallized compounds. 11

Table S2. X-ray data collection and processing. 10

Table S3. X-ray structure solution and refinement. 11

Synthetic Procedures for Compounds 55-59 12

Materials and Methods Proteomics 14

Selected NMR and LC/MS Data 16

References 30

Supporting Figures, Schemes, and Tables

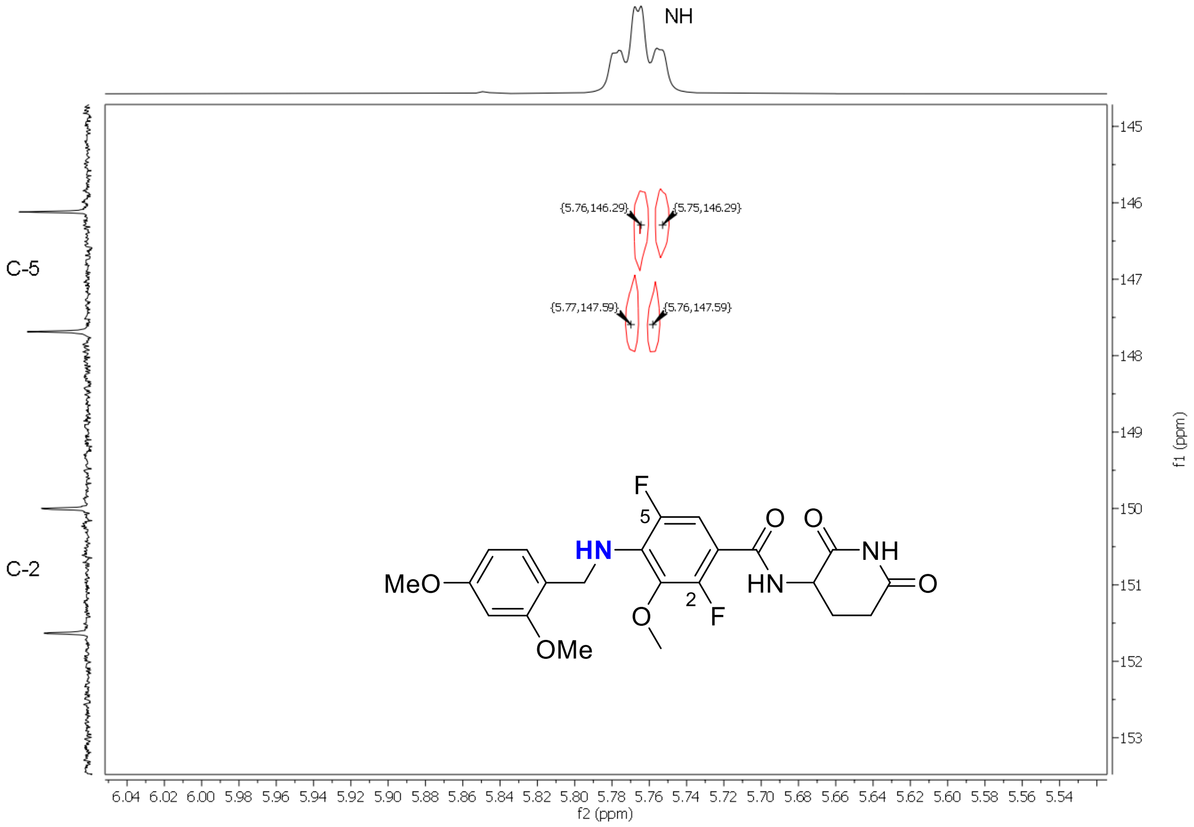

Figure S1. Section of the ^{1}H-^{13}C HMBC NMR spectrum of **14** in DMSO-d_6.

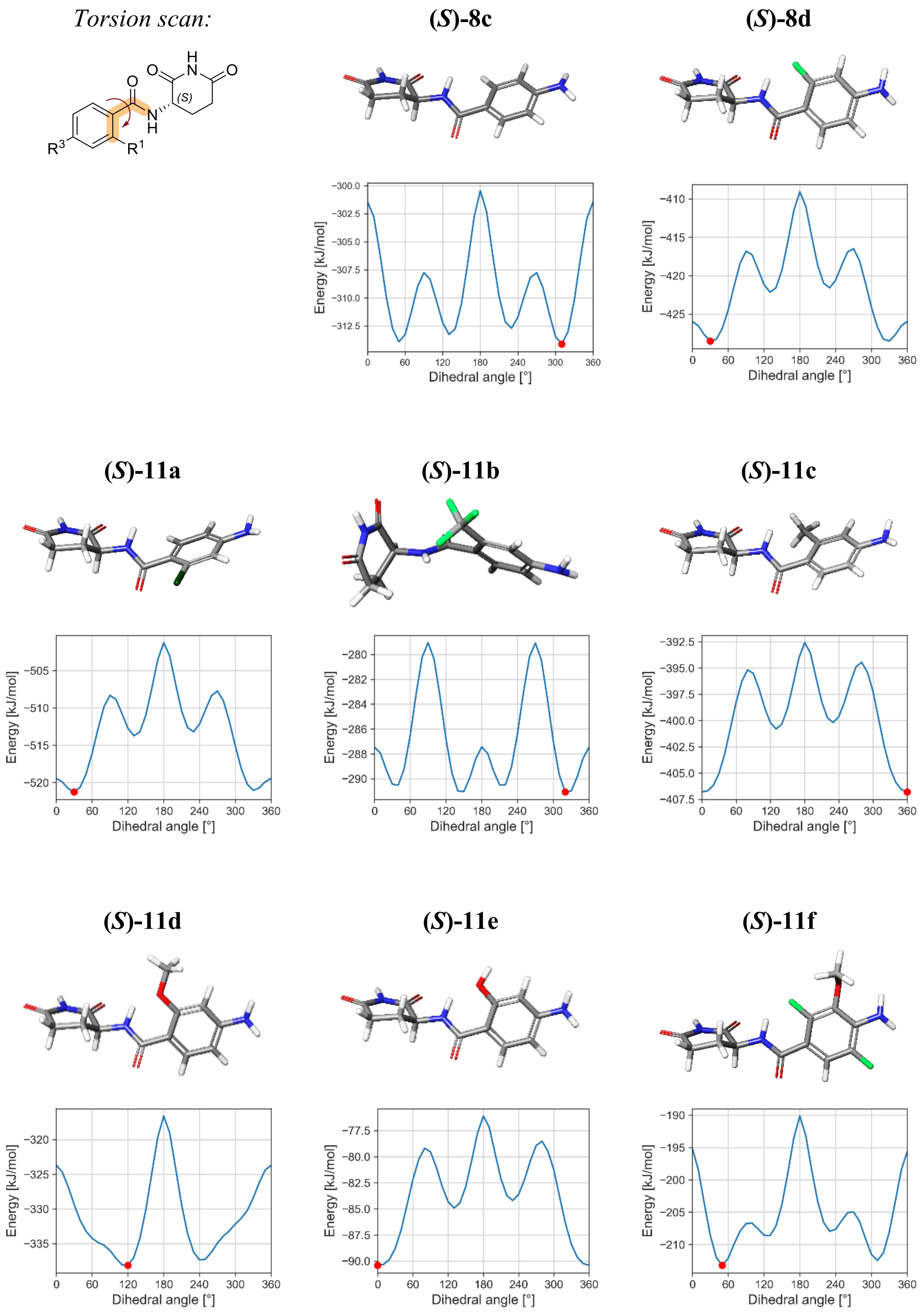

Figure S2. Torsion angle scans of compounds **8c**, **8d**, and **11a-f**. For simplification, only the *S*-configurated compound was considered. Red dot denotes global minimum.

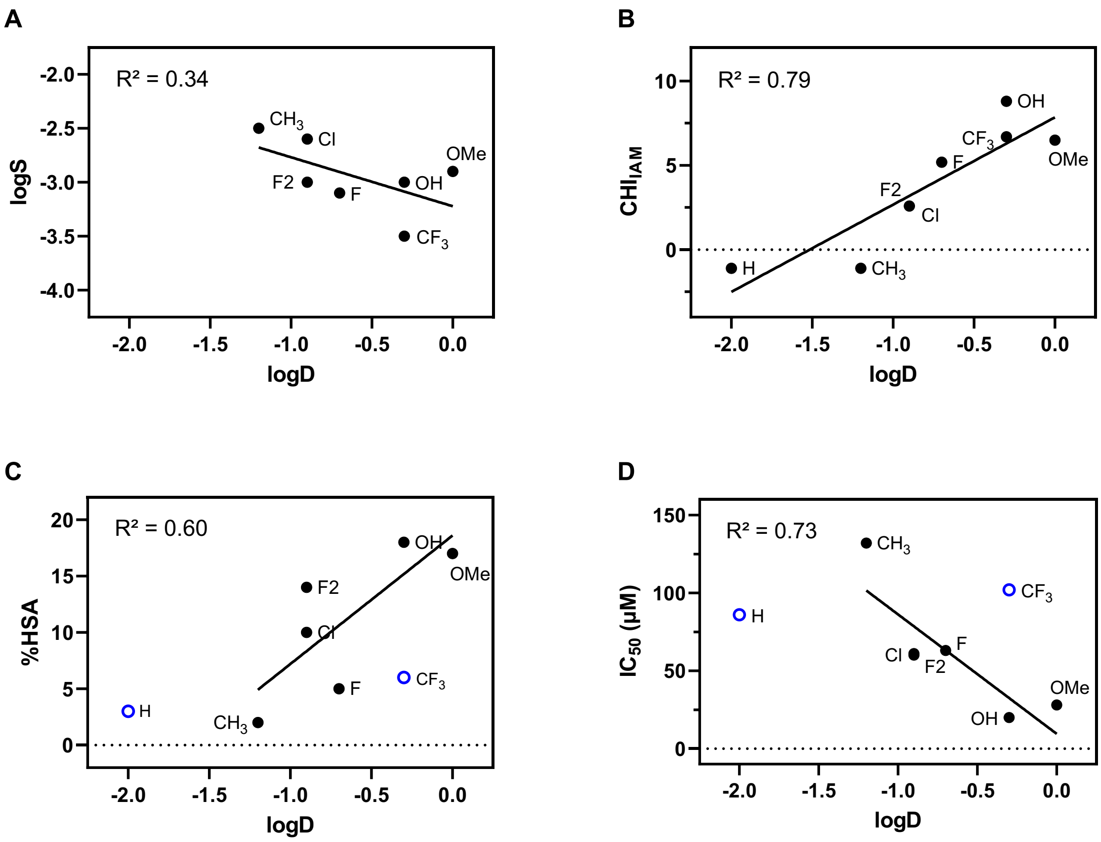

Figure S3. Correlation between physiochemical properties of **8c**, **8d** and **11** (for structures, see also Figure S2). (A) Logarithm of the solubility measured in mol/L at *p*H 6.8 by an HPLC-based method. (B) Chromatographic hydrophobicity index values referring to IAM chromatography (CHI_{IAM} values), an estimate for drug-membrane interactions and permeability. (C) Plasma protein binding, experimentally determined percentage of compound bound to human serum albumin. Compounds with blue circles were not considered in the linear regression. (D) Inhibitory concentration referring to a competitive MST experiment using the hTBD and our previously described reporter molecule.[1] Compounds with blue circles were not considered in the linear regression.

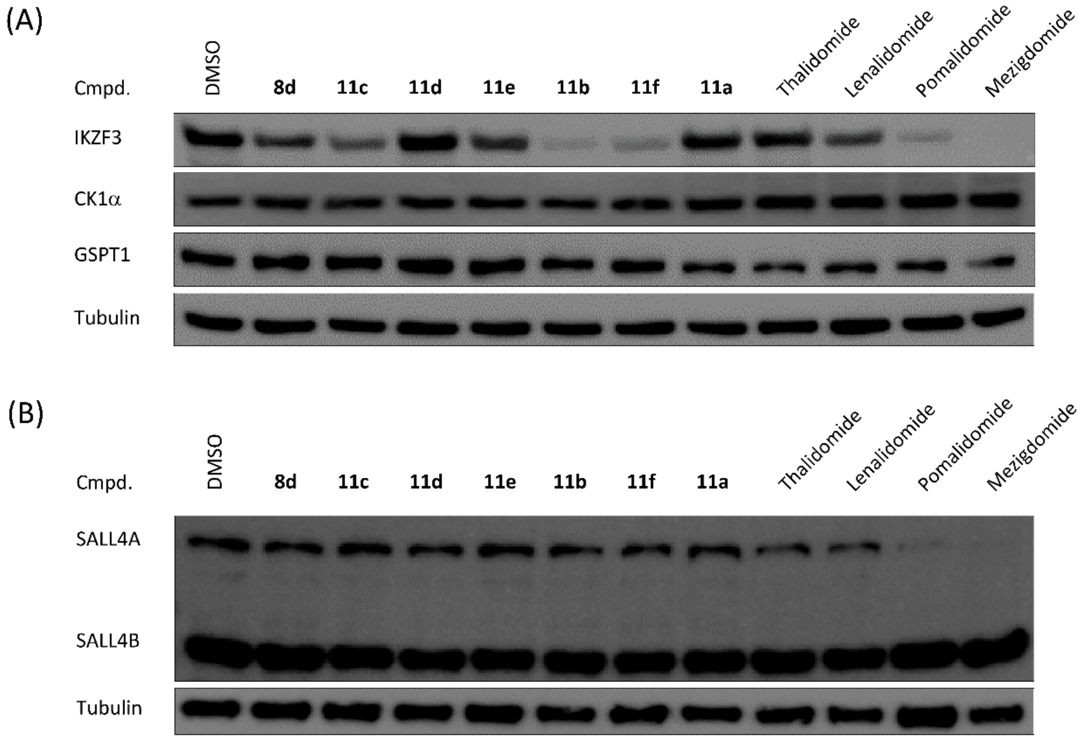

Figure S4. Neosubstrate modulation by benzamides **11** and established IMiDs. (A) MM.1S cells were treated with 0.1 μM of each compound for 16 h before lysis and blotting for IKZF3, GSPT1, and CK1α. (B) HuH6 cells were treated with 0.1 μM of compound for 16 h before lysis and blotting for SALL4.

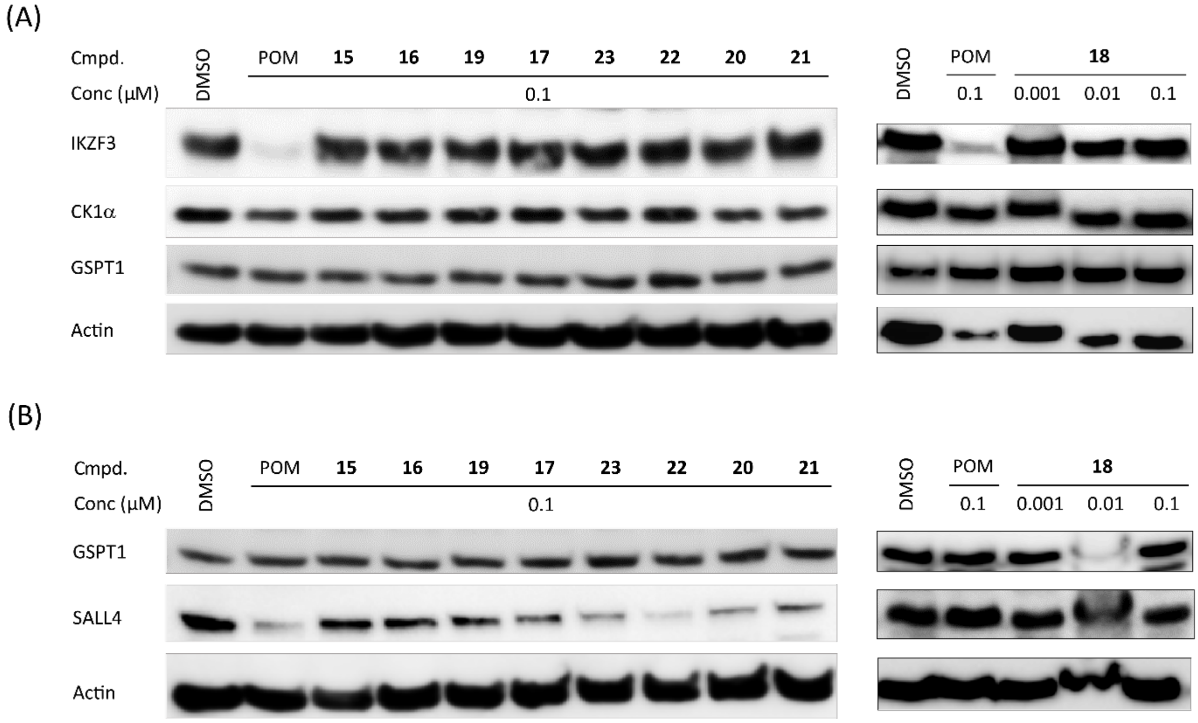

Figure S5. Effects of linker-connected CRBN ligands **15-23** on neosubstrates. (A) MM.1S cells were treated with 0.1 μM of compound for 16 h before lysis and blotting for IKZF3, CK1α, and GSPT1. (B) HuH6 cells were treated with 0.1 μM of compound for 16 h before blotting for GSPT1 and SALL4.

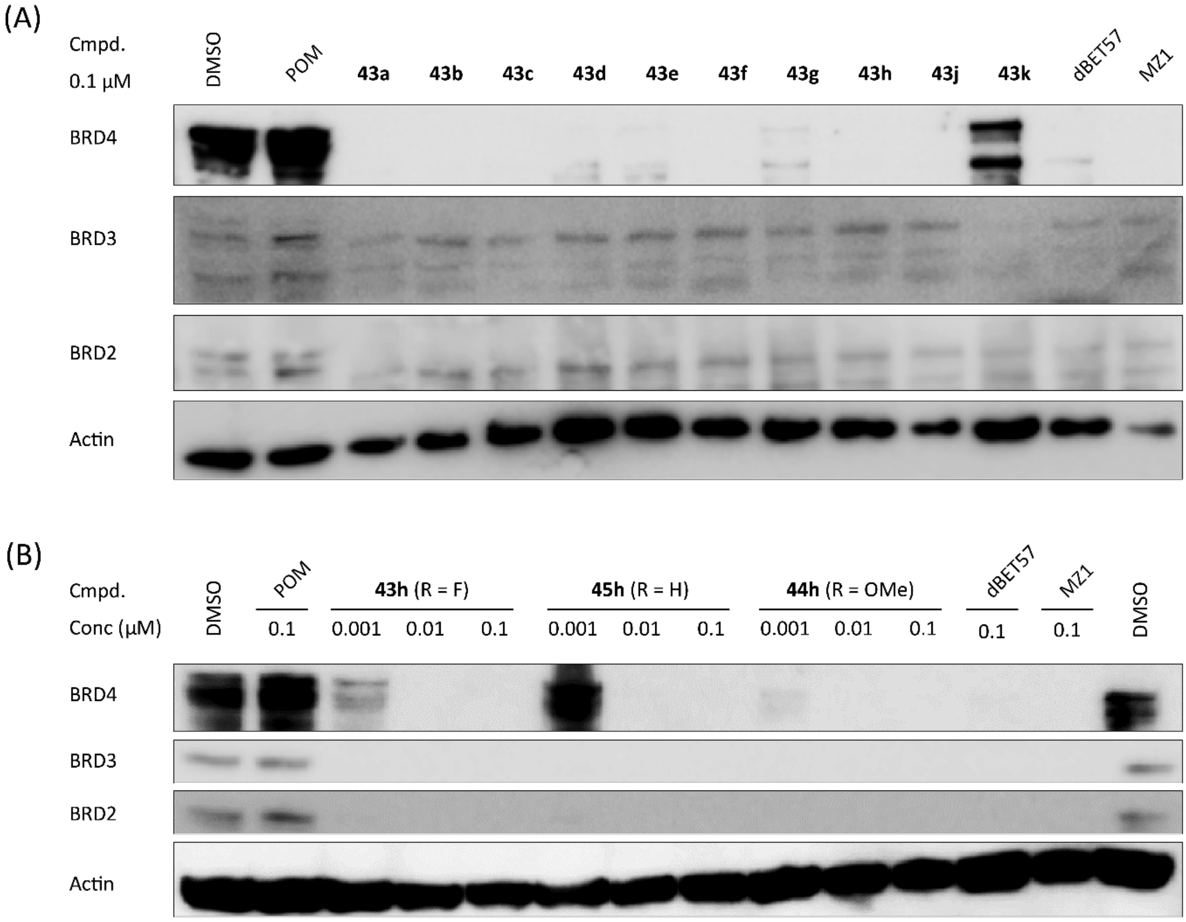

Figure S6. Evaluation of BRD4 PROTACs **43-45** in MV4;11 cells. (A) Western blot analyses of BRD4, BRD3, BRD2, and actin protein levels in MV4;11 cells treated with PROTACs **43** or the CRBN-recruiting reference dBET57 or the VHL-recruiting reference MZ1 for 24 h at 0.1 µM. (B) Western blot analyses of the homologous series **43h**, **44h**, and **45h** at three different concentrations after treatment of MV4;11 cells for 24 h.

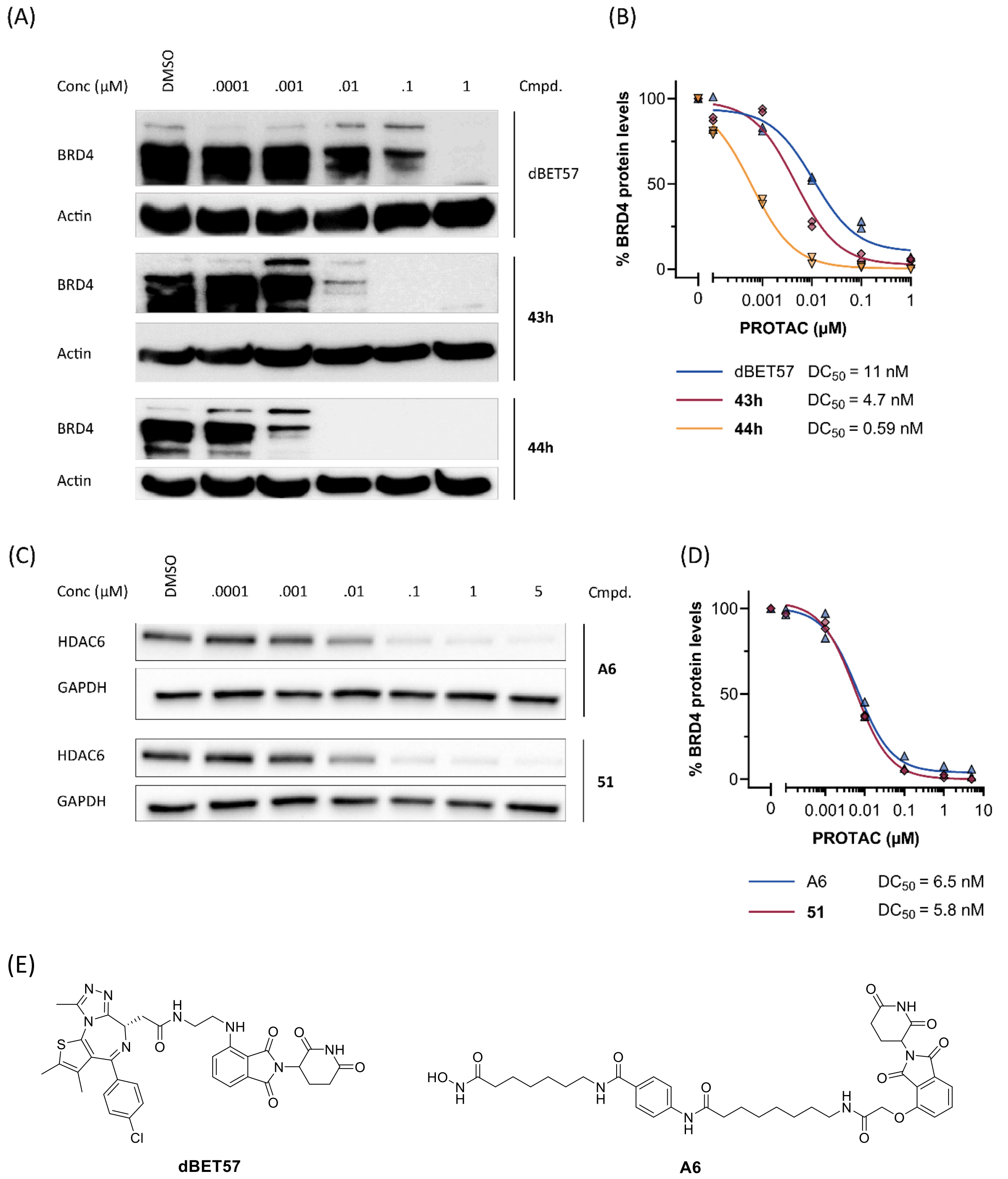

Figure S7. Concentration dependent analysis of BRD4 degradation by PROTACs. (A) PROTACs dBET57, **43h**, and **44h** induce BRD4 degradation in a dose-dependent manner. MOLT4 cells were treated with PROTACs at indicated concentrations for 24 h. (B) Quantification of (A) and calculation of the DC_{50} values from repeats (n = 2). (C) PROTACs A6 and **51** induce HDAC6 degradation in a dose-dependent manner. MM.1S cells were treated with PROTACs at indicated concentrations for 24 h. (D) Quantification of (C) and calculation of the DC_{50} values from repeats (n = 2). Representative blots and graphs are shown.(E) Chemical structures of the reference PROTACs dBET57 and A6.

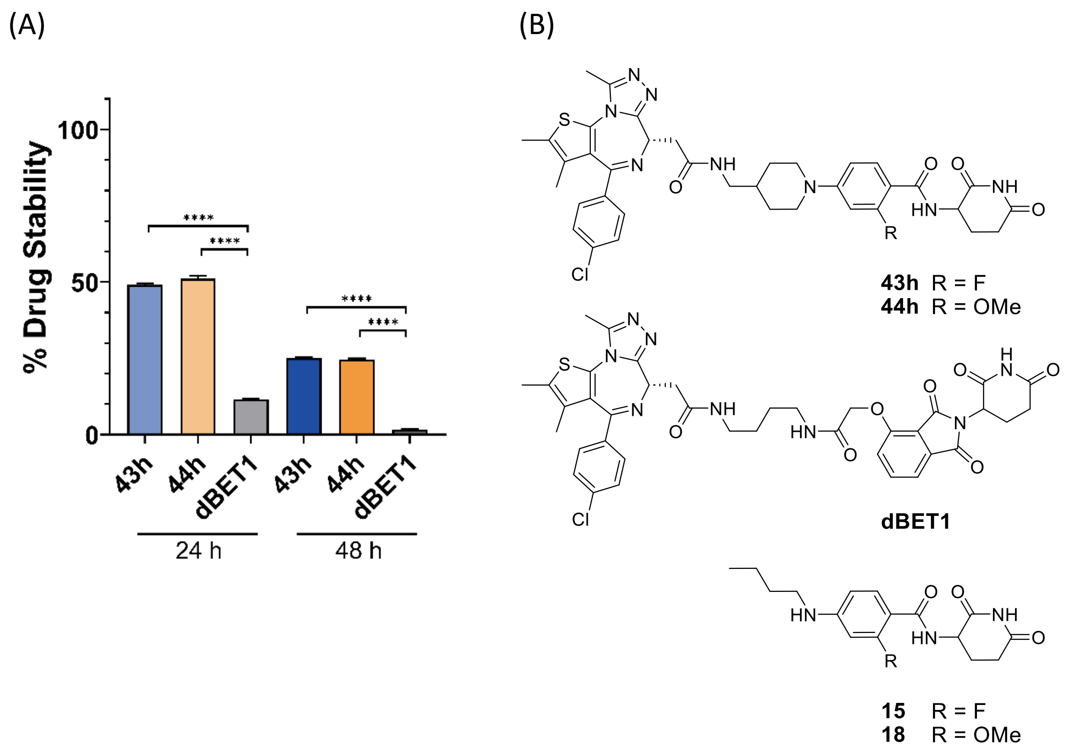

(C)	**43h**	**15**	**18**
Molecular weight (g/mol)	745	321	333
Solubility @ *p*H 6.8 (µg/mL)	1.1	81	7.7
Human microsomal stability ($t_{1/2}$ in min)	8.1	>60	>60
Human plasma protein binding (%fu)	7	15	10
Human plasma stability ($t_{1/2}$ in min)	>120	>120	>120
pDC$_{50}$ (MOLT4, 24 h)	8.3	--	--
D$_{max}$ (MOLT4, 24 h)	>99	--	--

Figure S8. Additional physiochemical properties of selected compounds. (A) PROTAC stability data in PBS buffer *p*H 7.4. Acetonitrile solutions of the PROTACs were mixed with 50 mM PBS buffer and incubated for 24 or 48 hours at 37 °C. Subsequently, aliquots were analysed by HPLC and normalized to initial values at t = 0 h. (B) Chemical structures of selected PROTACs and CRBN ligands along with physicochemical property profiles (C).

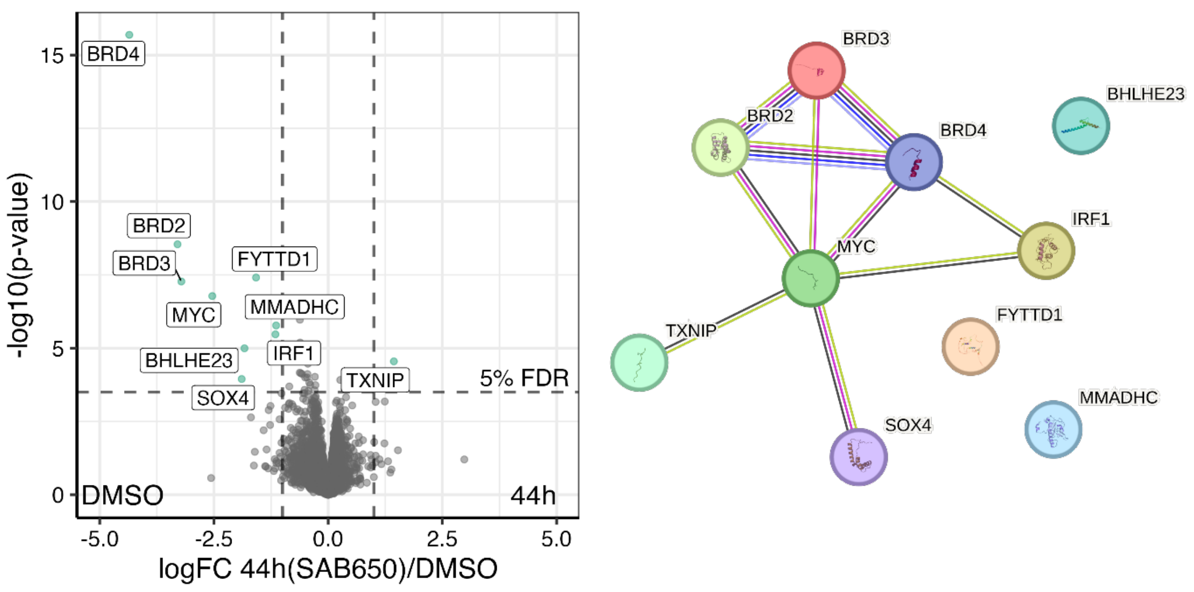

Figure S9. Proteins regulated by PROTAC **44h** which includes **44h** targets and downstream effects. Most regulated proteins are connected to MYC as indicated in the network graph obtained from STRING database searches.

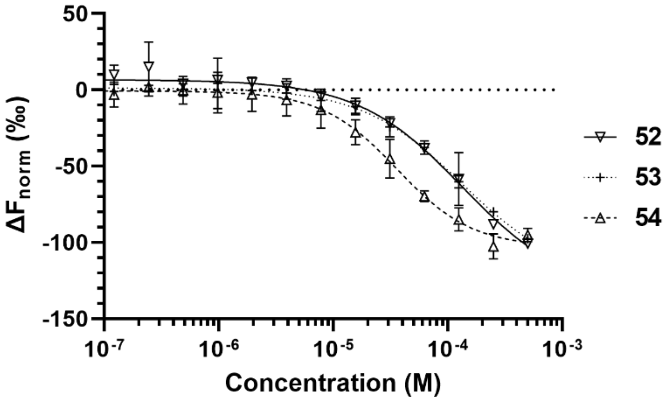

Figure S10. Dose-response curves for patent structures **52-54** obtained in competitive MST measurements with BODIPY-uracil and hTBD. Data is shown as mean ± s.d. (n=3).

Scheme S1. Synthesis of comparators **52-54**.

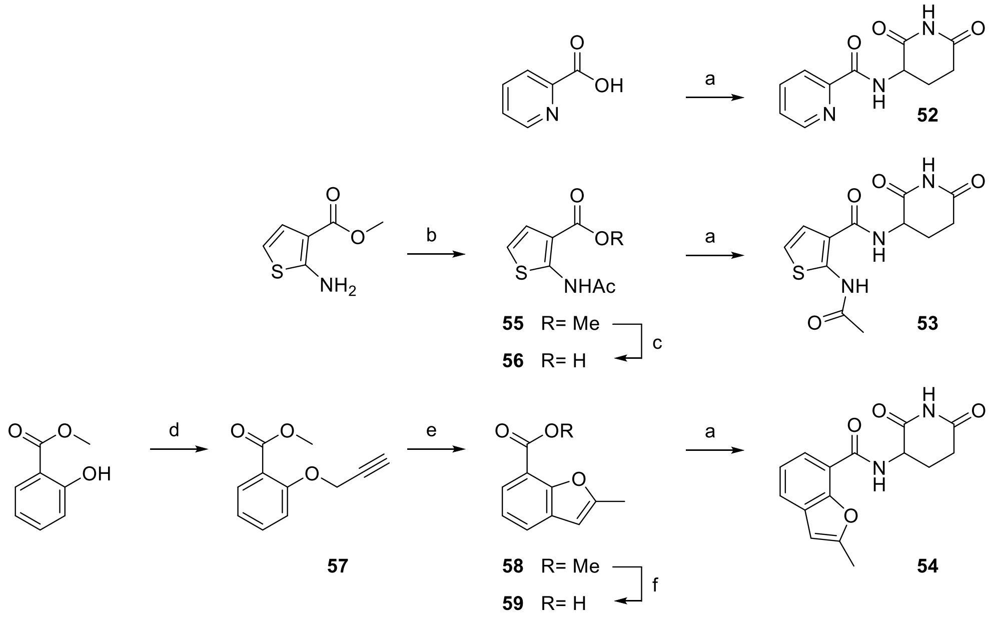

[a]Reagents and conditions: (a) EDC × HCl, HOBt × H_2O, 3-aminopiperidine-2,6-dione hydrochloride, DIPEA, DMF, rt, 16 h, 12–55%; (b) Ac_2O, 140 °C, 30 min, 61%; (c) KOH, MeOH, H_2O, 50 °C, 16 h, 89%; (d) propargyl bromide, Cs_2CO_3, DMF, rt, 16 h, 88%; (e) CsF, *N*,*N*-diethyl aniline, 200 °C, 3 h, 15%; (f) 2M NaOH (aq), MeOH, THF, 40 °C, 6 h, 76%.

Cmpd	Chain A	Chain B	Chain C	VDW distance
**8d**	n/a	1.9 Å	n/a	F···H sum: 2.67 Å
**11a**	1.9 Å	1.7 Å	n/a	Cl···H sum: 2.95 Å
**11c**	1.3 Å	1.7 Å	n/a	H···H sum: 2.4 Å
**11d**	1.9 Å	2.0 Å	1.9 Å	O···H sum: 2.72 Å
**11e**	1.9 Å	1.9 Å	n/a	O···H sum: 2.72 Å
**11f**	1.9 Å	1.8 Å	n/a	F···H sum: 2.67 Å

Table S1. Sub-van der Waals distances observed for the crystallized compounds. The observed distances between the indicated atoms in the respective protein chain (A, B, or C) in the asymmetric unit of the co-crystal structures are given. n/a: no compound bound in the respective protein chain.

	MsCI4-**8d**	MsCI4-**11a**	MsCI4-**11b**	MsCI4-**11c**	MsCI4-**11d**	MsCI4-**11e**	MsCI4-**11f**
Wavelength (Å)	1.00	1.00	1.00	1.00	1.00	1.00	1.00
Space group	P 2_1 2_1 2_1	P 2_1 2_1 2_1	P 2_1 2_1 2_1	P 2_1 2_1 2_1	P 2_1 2_1 2_1	P 2_1 2_1 2_1	P 2_1 2_1 2_1
a, b, c (Å)	56.79, 59.52, 88.15	56.51, 59.54, 88.19	56.84, 59.57, 88.48	56.99, 59.13, 87.14	56.41, 59.55, 88.41	56.33, 59.74, 88.31	56.42, 59.47, 88.15
α, β, γ (°)	90, 90, 90	90, 90, 90	90, 90, 90	90, 90, 90	90, 90, 90	90, 90, 90	90, 90, 90
Resolution range (Å)	35.42 - 1.47 (1.52 - 1.47)	40.99 - 2.25 (2.33 - 2.25)	49.42 - 2.30 (2.38 - 2.30)	37.12 - 1.84 (1.91 - 1.84)	40.95 - 1.70 (1.76 - 1.70)	37.17 - 1.75 (1.81 - 1.75)	47.52 - 1.85 (1.92 - 1.85)
Total No. of reflections	664940 (109801)	188951 (30600)	179890 (28972)	340486 (54241)	430577 (61914)	312122 (50046)	300966 (28943)
No. of unique reflections	51535 (8199)	14648 (2311)	13895 (2193)	26237 (4157)	33503 (5334)	30740 (4878)	25046 (3258)
Completeness (%)	99.97 (99.88)	99.84 (99.44)	99.97 (100.00)	99.97 (100.00)	99.98 (100.00)	99.96 (99.93)	96.37 (74.23)
$CC_{1/2}$	99.9 (66.4)	99.8 (63.1)	99.7 (58.0)	99.9 (61.8)	99.9 (61.9)	99.8 (56.9)	99.8 (58.8)
$\langle I/\sigma(I) \rangle$	15.28 (1.09)	10.68 (1.26)	10.48 (1.09)	16.62 (1.19)	14.74 (1.21)	11.33 (1.27)	11.62 (1.00)
R_{meas}	7.8 (200.4)	16.2 (196.6)	18.2 (255.7)	8.1 (198.1)	8.8 (177.0)	12.5 (166.4)	13.1 (159.8)
Overall B factor from Wilson plot ($Å^2$)	25.33	53.08	55.36	40.11	31.65	28.12	34.42

Table S2. X-ray data collection and processing. Values in parentheses correspond to the outer resolution shell.

	MsCI4-**8d**	MsCI4-**11a**	MsCI4-**11b**	MsCI4-**11c**	MsCI4-**11d**	MsCI4-**11e**	MsCI4-**11f**
Resolution range (Å)	35.42 - 1.47 (1.52 - 1.47)	40.99 - 2.25 (2.33 - 2.25)	49.42 - 2.30 (2.38 - 2.30)	37.12 - 1.84 (1.91 - 1.84)	40.95 - 1.70 (1.76 - 1.70)	37.17 - 1.75 (1.81 - 1.75)	47.52 - 1.85 (1.92 - 1.85)
Completeness (%)	99.97 (99.88)	99.84 (99.44)	99.97 (100.00)	99.97 (100.00)	99.98 (100.00)	99.96 (99.93)	96.37 (74.23)
Final R_{cryst}	0.19 (0.34)	0.20 (0.34)	0.18 (0.33)	0.18 (0.33)	0.17 (0.34)	0.18 (0.33)	0.18 (0.35)
Final R_{free}	0.22 (0.34)	0.27 (0.31)	0.26 (0.36)	0.21 (0.35)	0.21 (0.36)	0.22 (0.30)	0.23 (0.36)
No. of non-H atoms							
Total	2473	2267	2308	2126	2475	2562	2503
Protein	2288	2208	2237	2010	2325	2412	2373
Ligand	75	41	47	61	63	60	52
Solvent	110	18	24	55	87	90	78
R.m.s. deviations							
Bond lengths (Å)	0.011	0.008	0.016	0.010	0.009	0.009	0.009
Angles (°)	1.57	1.30	2.15	1.48	1.47	1.46	1.38
Average B factors (Å^2)							
Overall	23.1	44.2	38.2	35.2	28.6	35.5	29.5
Protein	22.0	43.9	37.7	34.1	27.8	35.0	28.7
Ligand	34.2	53.7	50.2	53.8	31.3	39.0	39.9
Solvent	38.2	61.9	60.0	54.6	47.8	45.4	49.0
Ramachandran plot statistics							
Most favoured (%)	97.56	98.58	96.80	99.61	97.60	97.7	98.3
Allowed (%)	2.44	1.42	2.85	0.39	2.41	2.30	1.68
Ligand bound in chain(s)	A, B, C	A, B	A, B	A, B	A, B, C	A, B, C	A, B

Table S3. X-ray structure solution and refinement. Values in parentheses correspond to the outer resolution shell.

Synthetic Procedures for Compounds 55-59

*Methyl 2-acetamidothiophene-3-carboxylate (**55**).* Methyl 2-aminothiophene-3-carboxylate (1.57 g, 10 mmol) was dissolved in Ac_2O (5 mL) and heated to 135 °C for 10 min in a flask open to air. After cooling, it was poured onto H_2O (100 mL) and EtOAc (100 mL), and the mixture was vigorously stirred at rt for 1 h. The organic layer was separated and washed with brine (50 mL), dried over Na_2SO_4, filtered, and concentrated *in vacuo*. The crude product was purified by FC (80 g, 30 μm, gradient from 20 to 50% EtOAc/cyclohexane) to give a colorless solid. Yield: 1.21 g (61%); mp 94-96 °C, lit. mp 96-97 °C;[2] R_f = 0.68 (60% EtOAc in cyclohexane); ^{1}H NMR (500 MHz, DMSO-d_6) δ 2.16 (s, 3H), 3.83 (s, 3H), 7.86 (d, J = 5.4 Hz, 1H), 7.89 – 7.94 (m, 1H), 9.96 (s, 1H); ^{13}C NMR (126 MHz, DMSO-d_6) δ 24.10, 52.13, 110.22, 122.35, 132.95, 143.98, 163.49, 167.75; LC-MS (ESI) (90% H_2O to 100% MeCN in 10 min, then 100% MeCN to 20 min, DAD 220-600 nm), t_R = 4.67 min, 99% purity, m/z $[M + H]^+$ calcd for $C_8H_{10}NO_3S$, 200.04; found, 200.1.

*2-Acetamidothiophene-3-carboxylic acid (**56**).* Compound **55** (1.20 g, 6.0 mmol) was dissolved in MeOH (20 mL), and KOH (0.60 g, 10.8 mmol) in H_2O (10 mL) was added. The mixture was stirred at 50 °C for 16 h. After cooling, it was diluted with H_2O (100 mL), and the aqueous layer was washed with EtOAc (100 mL). Subsequently, the aqueous solution was acidified with 2N HCl until *p*H = 2, and it was extracted with EtOAc (2 × 50 mL). The combined organic layers were washed with brine (50 mL), dried over Na_2SO_4, filtered, and concentrated *in vacuo* to give a colorless solid. Yield: 0.99 g (89%); mp 222 °C (dec); R_f = 0.45 (10% MeOH/CH_2Cl_2); ^{1}H NMR (600 MHz, DMSO-d_6) δ 2.14 (s, 3H), 7.80 (d, J = 5.4 Hz, 1H), 7.92 (d, J = 5.4 Hz, 1H), 10.11 (s, 1H), 13.37 (s, 1H); ^{13}C NMR (151 MHz, DMSO-d_6) δ 24.26, 111.40, 122.17, 132.24, 143.73, 164.97, 167.63; LC-MS (ESI) (90% H_2O to 100% MeCN in 10 min, then 100% MeCN to 20 min, DAD 220-600 nm), t_R = 0.31 min, 99% purity, m/z $[M + H]^+$ calcd for $C_7H_8NO_3S$, 186.02; found, 185.9.

*Methyl 2-prop-2-ynoxybenzoate (**57**).* This compound was synthesized as reported previously.[3] In brief, methyl salicylate (3.04 g, 20 mmol) and Cs_2CO_3 (6.52 g, 24 mmol) were suspended in dry DMF (20 mL) and stirred at rt for 10 min. Subsequently, propargyl bromide (9.2 mol/L solution in toluene, 2.8 mL, 26 mmol) was added, and the mixture was stirred at rt for 16 h. It was then quenched with H_2O (100 mL) and extracted with EtOAc (2 × 100 mL). The combined organic layers were washed with 5% LiCl solution and brine (100 mL), dried over Na_2SO_4, filtered, and concentrated *in vacuo*. The crude product was purified by FC (120 g, 50 μm, gradient from 0 to 40% EtOAc/cyclohexane) to give a colorless oil. Yield: 3.36 g

(88%); R_f = 0.54 (30% EtOAc in cyclohexane); ^{1}H NMR (500 MHz, DMSO-d_6) δ 3.57 (t, J = 2.4 Hz, 1H), 3.78 (s, 3H), 4.87 (d, J = 2.5 Hz, 2H), 7.01 – 7.09 (m, 1H), 7.21 (d, J = 8.3 Hz, 1H), 7.50 – 7.57 (m, 1H), 7.65 (dd, J = 1.8, 7.6 Hz, 1H); ^{13}C NMR (126 MHz, DMSO-d_6) δ 52.00, 56.35, 78.63, 79.02, 114.45, 121.07, 121.10, 130.79, 133.27, 156.10, 166.12; LC-MS (ESI) (90% H_2O to 100% MeCN in 10 min, then 100% MeCN to 20 min, DAD 220-600 nm), t_R = 5.33 min, 99% purity, m/z [M + H]$^+$ calcd for $C_{11}H_{11}O_3$, 191.07; found, 191.1.

*Methyl 2-methylbenzofuran-7-carboxylate (**58**)*. This compound was synthesized as reported previously.[3] Compound **57** (1.90 g, 10 mmol) was dissolved in *N*,*N*-diethylaniline (10 mL) and cesium fluoride (1.97 g, 13 mmol) was added. The mixture was stirred at 200 °C for 3 h. After cooling, the brown mixture was directly loaded onto a silica gel column equilibrated with *n*-hexanes. The product was then eluated from the column with 20% EtOAc in *n*-hexanes. Product-enriched fractions were pooled and subjected to FC (80 g, 30 μm, gradient from 0 to 20% EtOAc/*n*-hexanes) to give a yellow oil. Yield: 0.29 g (15%); R_f = 0.60 (20% EtOAc in cyclohexane); ^{1}H NMR (500 MHz, DMSO-d_6) δ 2.48 (s, 3H), 3.90 (s, 3H), 6.68 (d, J = 1.2 Hz, 1H), 7.29 (t, J = 7.7 Hz, 1H), 7.72 – 7.82 (m, 2H); ^{13}C NMR (126 MHz, DMSO-d_6) δ 13.88, 52.16, 102.87, 113.99, 122.58, 125.21, 125.56, 130.80, 152.36, 156.78, 164.78; LC-MS (ESI) (90% H_2O to 100% MeCN in 10 min, then 100% MeCN to 20 min, DAD 220-600 nm), t_R = 6.20 min, 98% purity, m/z [M + H]$^+$ calcd for $C_{11}H_{11}O_3$, 191.07; found, 191.1.

*2-Methylbenzofuran-7-carboxylic acid (**59**)*. Compound **58** (285 mg, 1.5 mmol) was dissolved in THF (3 mL) and MeOH (0.5 mL), after which aqueous 2N NaOH (1.5 mL) was added. The mixture was stirred at 40 °C for 6 h. It was diluted with H_2O (50 mL), acidified with 2N HCl until *p*H = 2, and it was extracted with EtOAc (2 × 50 mL). The combined organic layers were washed with brine (50 mL), dried over Na_2SO_4, filtered, and concentrated *in vacuo* to give a colorless solid, which was used in the next step without further purification. Yield: 0.20 g (76%); R_f = 0.46 (EtOAc).

Materials and Methods Proteomics

Sample Preparation. Cell pellets were lysed with 8M urea lysis buffer for 15 min at 4 °C (8M urea, 50 mM Tris *p*H 8, 150 mM NaCl, 1 mM 2-chloroacetamide), supplemented with protease inhibitors (2 µg/ml aprotinin, 10 µg/ml leupeptin, 1 mM phenylmethylsulfonyl fluoride) as described before.[4] Lysates were then clarified by centrifugation (20,000g, 15 min, 4°C). Disulfide bonds were reduced (5 mM dithiothreitol for 1 h) and alkylated (40 mM chloroacetamide for 45 min in the dark). Afterwards samples were diluted 1 : 4 with 50 mM Tris–HCl *p*H 8 and digested using sequencing grade LysC (Wako Chemicals) for 2 h in weight-to-weight ratio of 1 : 50. Finally sequencing grade trypsin (Promega) was added at a weight-to-weight ratio of 1 : 50, and digestion was carried out overnight. Samples were acidified with formic acid (FA) followed by centrifugation (20,000g, 15 min). The supernatant was further processed using Sep-Pak C18 cc Cartridges (Waters) for desalting.

Mass Spectrometry. For the LC/MS analysis, 1 µg of desalted peptides was utilized for each sample. Peptide were separated on a Vanquish Neo System (Thermo Fisher) with a gradient lasting 106 min and a flow rate of 250 µL/min. The mobile phase B was gradually increased from 4% to 20% over the first 67 min, then to 30% over the next 20 min, followed by 60% for 10 min, 90% for 5 min, and finally 0% for 2 min. MS data was acquired on an Exploris 480 (Thermo Fisher) using data-independent acquisition (DIA) mode. Full scans were obtained at a resolution of 120,000, scanning a range of 350–1650 *m*/*z*. The maximum injection time (IT) was set at 20 ms, with an automatic gain control (AGC) target value of 3e6. Subsequent to the full scan, narrow isolation windows were used, covering the range of 375-1430 *m*/*z* with isolation windows ranging from 14 to 440 acquired at 30,000 resolution. The fixed first mass was set at 200 *m*/*z*, with an AGC target value of 300e6 (3000%), and a maximum IT of 54 ms. The normalized collision energy was set in stepped mode at 26%, 29%, and 32%. Dynamic exclusion was employed for 30 s, and ions with charge states of 1, 6, or higher were excluded from fragmentation.

Proteomics Data Analysis. Raw data was searched using DIA-NN 1.8.1 software against the human UniProt reference proteome.[5] Library-free mode was used, with the in silico FASTA digest parameter enabled. The peptide length range was set to 7-30, and the precursor charge range was set to 1-4. The *m*/*z* range for precursors was 340-1650, and for fragment ions, it was 200-1800. The 'match between runs' parameter was enabled. LFQ protein intensities from the DIA-NN pg output table were log2 transformed, filtered for valid values (> 70%) and contaminants. The resulting intensities were median normalized, and missing values were

imputed from a normal distribution with a downshift (-1.8 SD from the mean and the distribution width is 0.3 SD). Comparative analysis of experimental groups was conducted using a two-sided moderated two-sample t-test. The resulting p-values were corrected using the Benjamini-Hochberg method. The data analysis was performed using R (4.3.1).

Selected NMR and LC/MS Data

^{1}H and ^{13}C NMR spectrum of compound **8d**

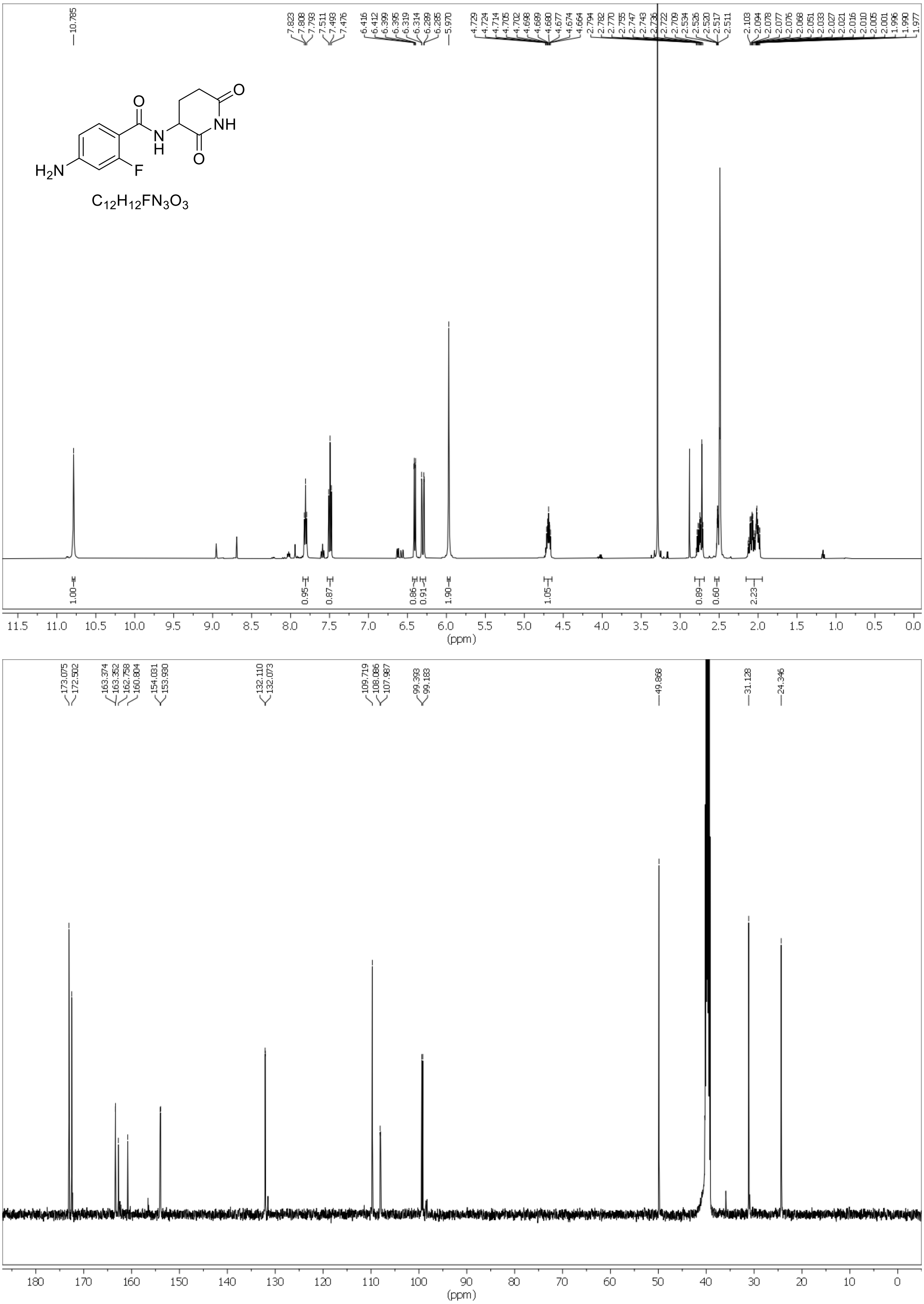

^{1}H and ^{13}C NMR spectrum of compound **11a**

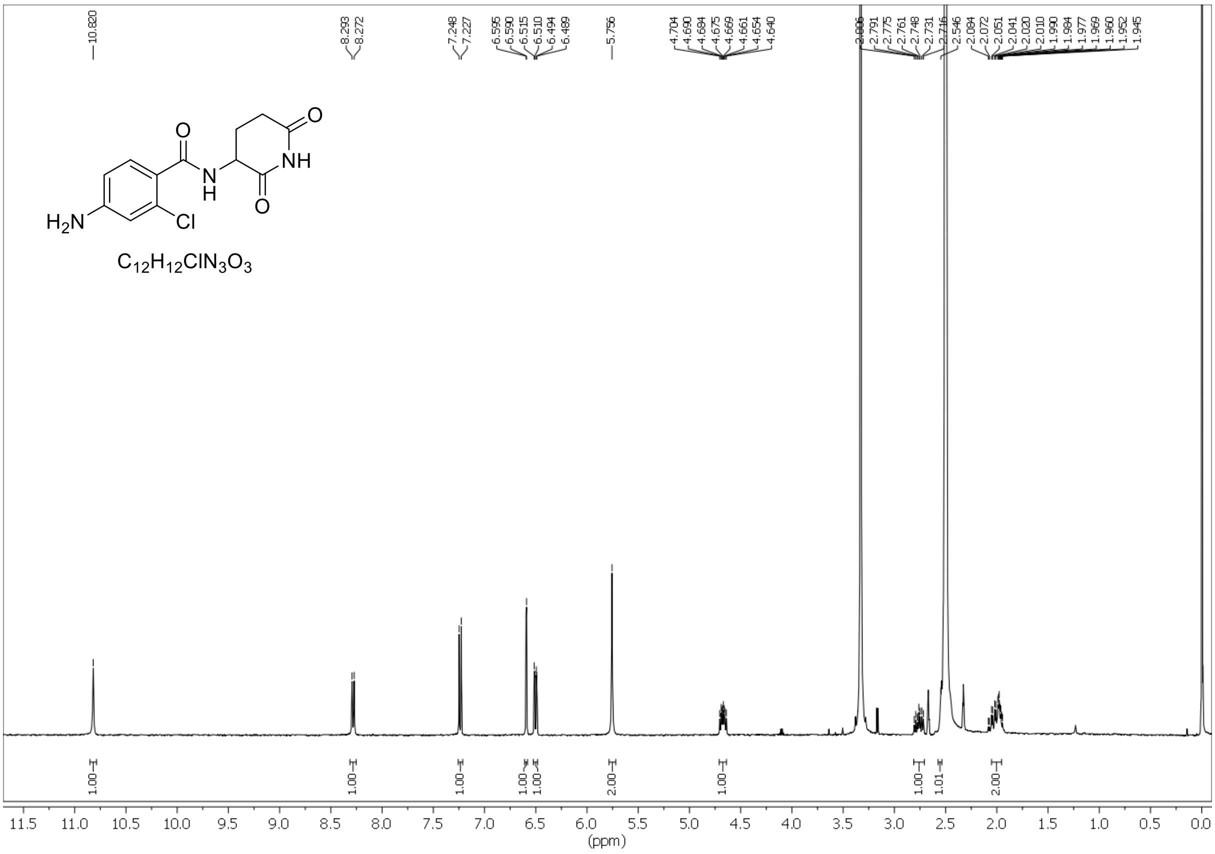

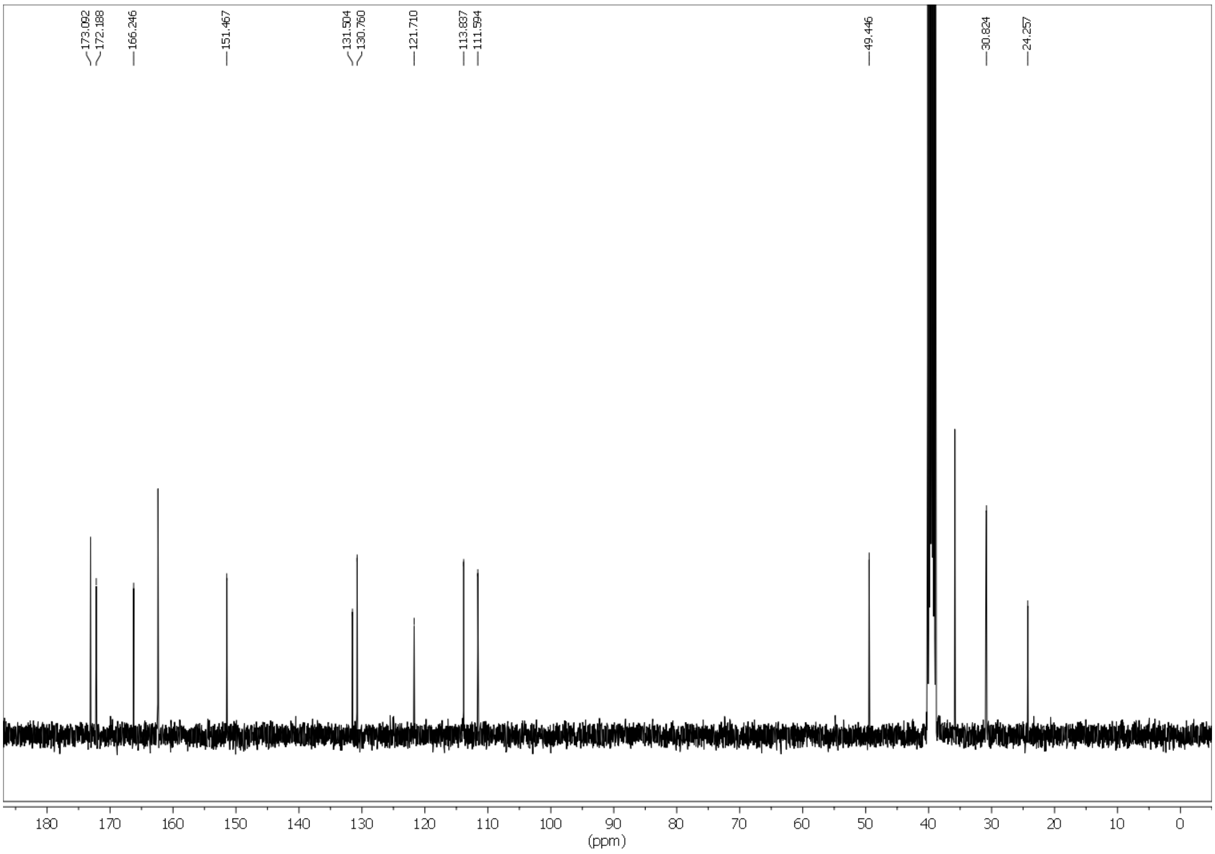

^{1}H and ^{13}C NMR spectrum of compound **11b**

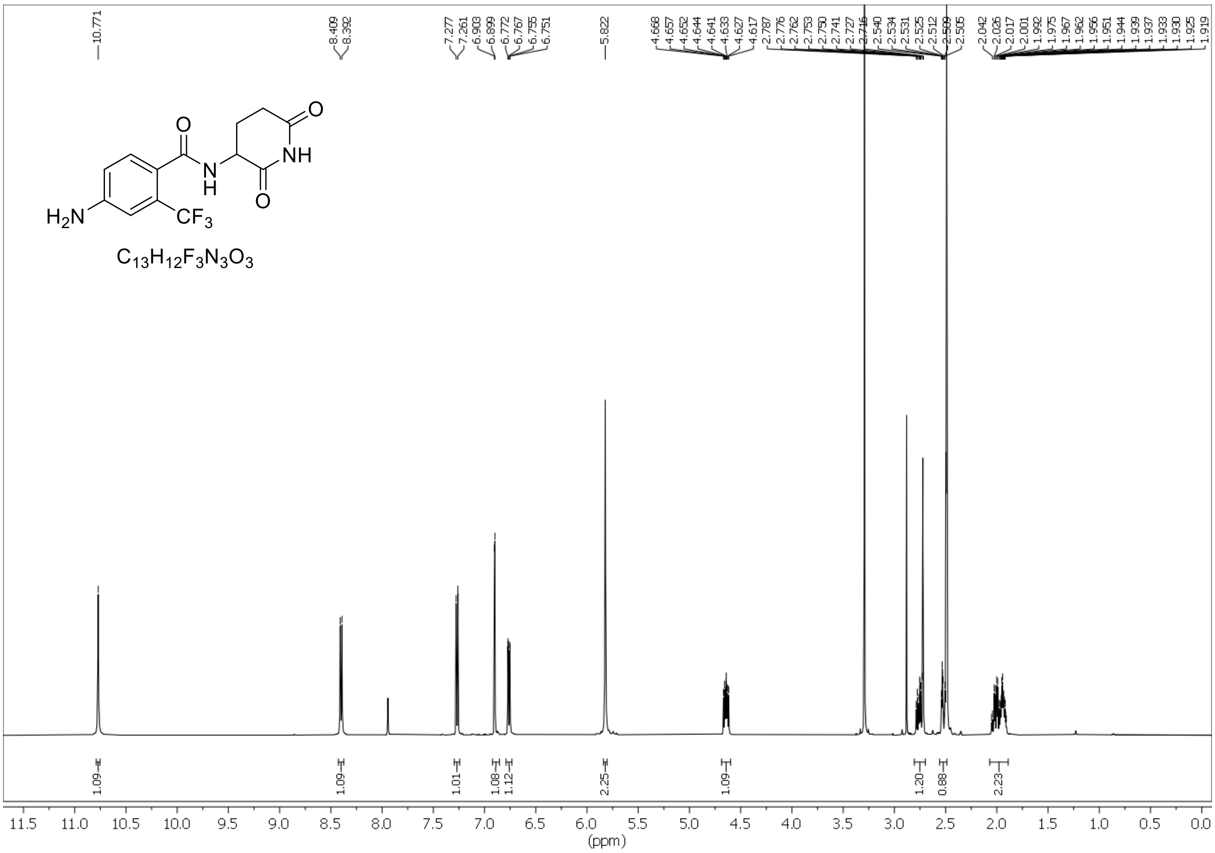

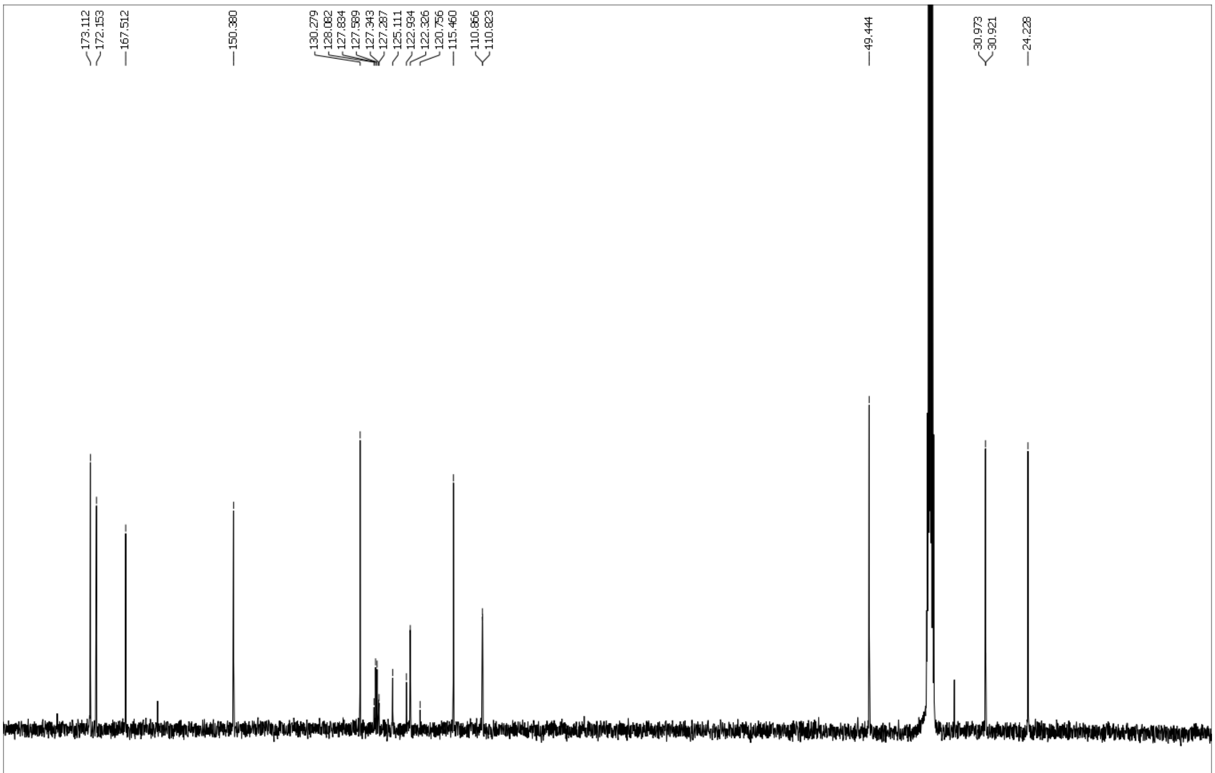

^{1}H and ^{13}C NMR spectrum of compound **11c**

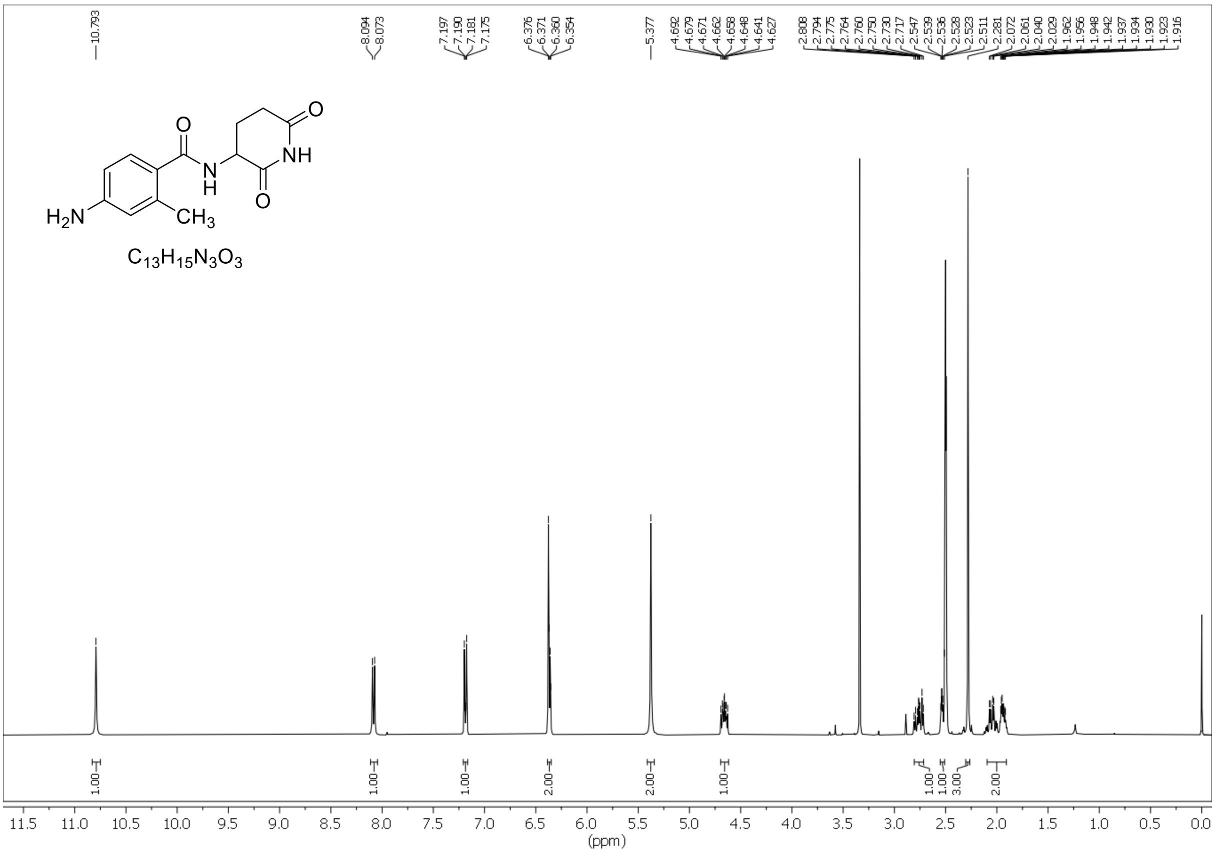

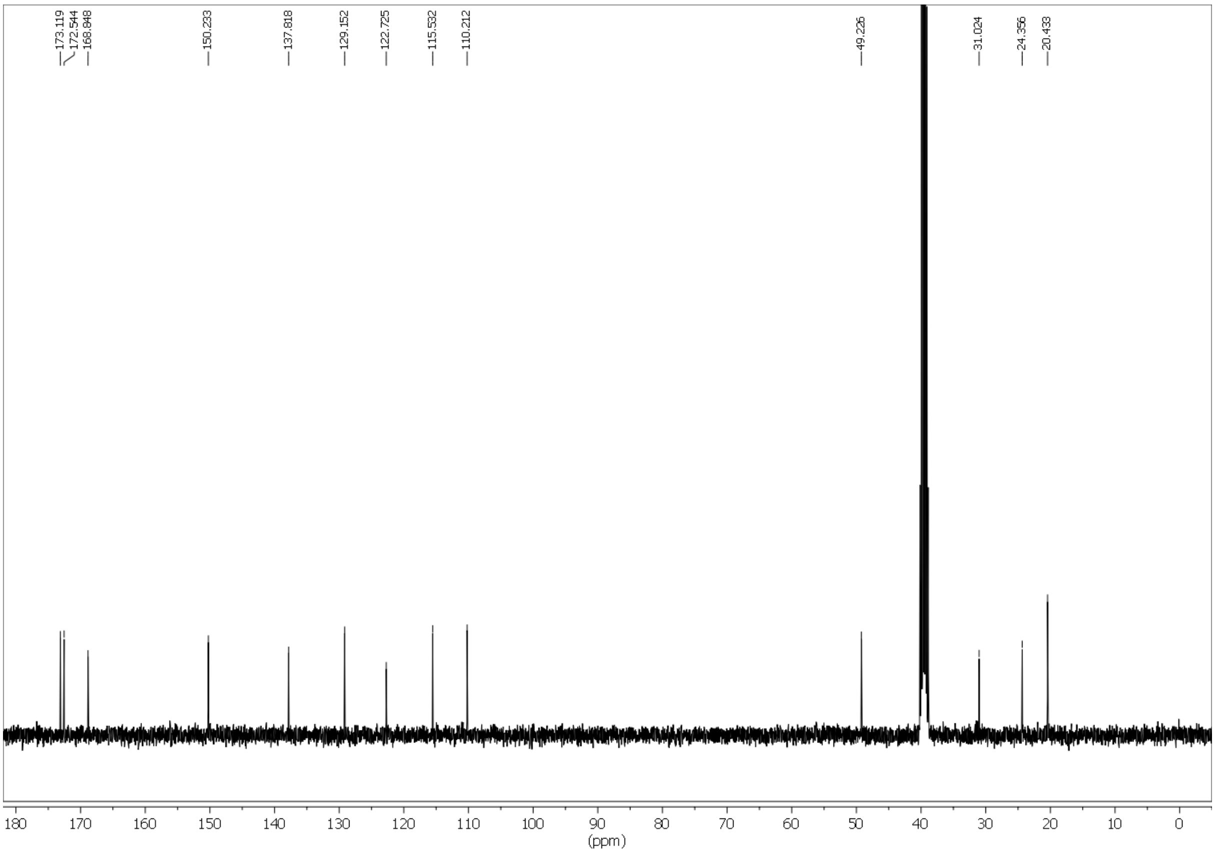

^{1}H and ^{13}C NMR spectrum of compound **11d**

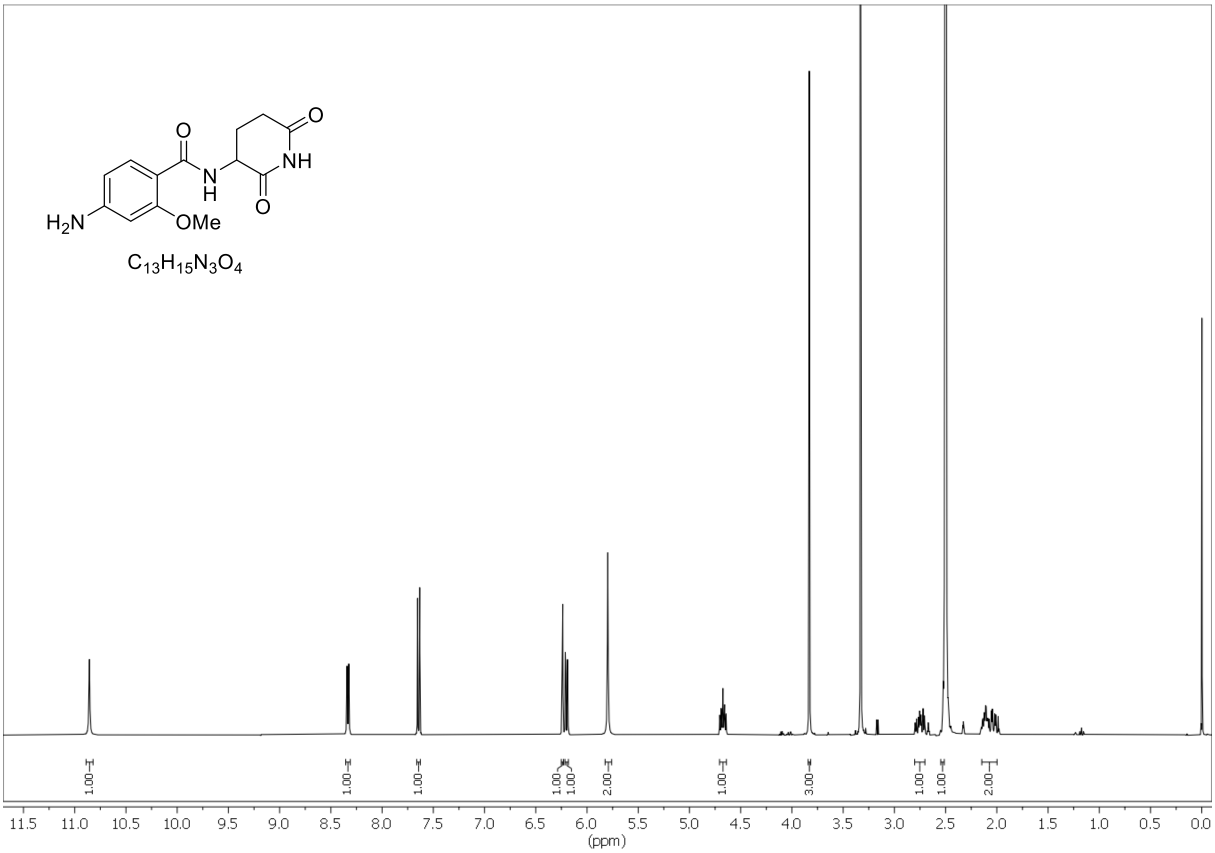

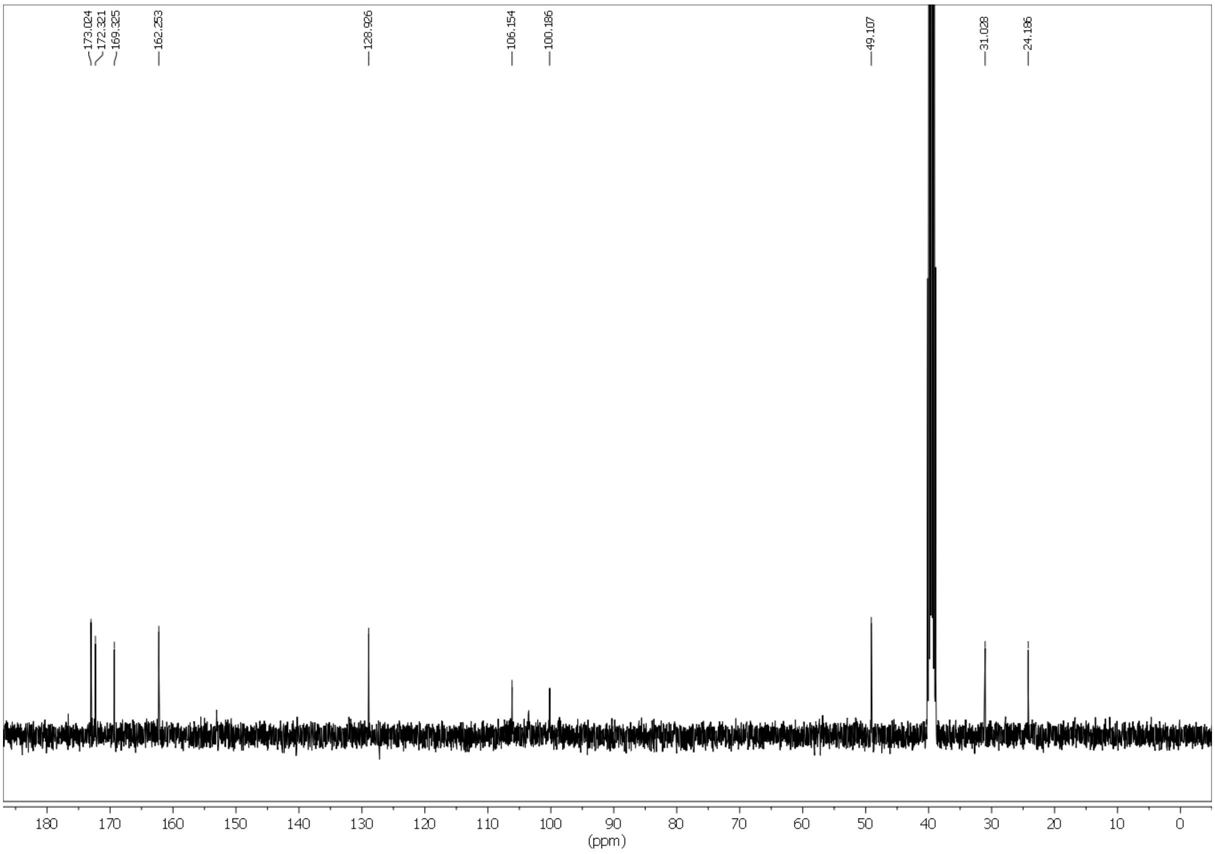

^{1}H and ^{13}C NMR spectrum of compound **11e**

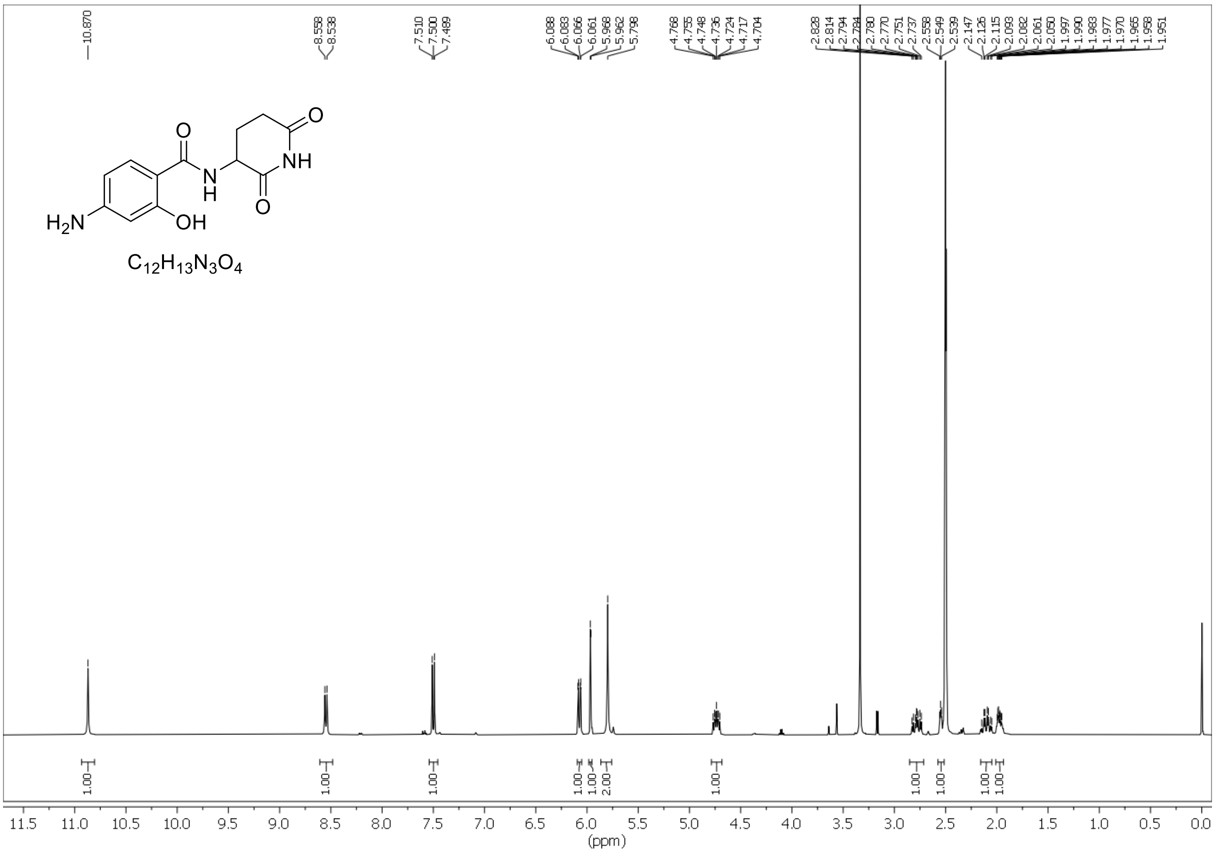

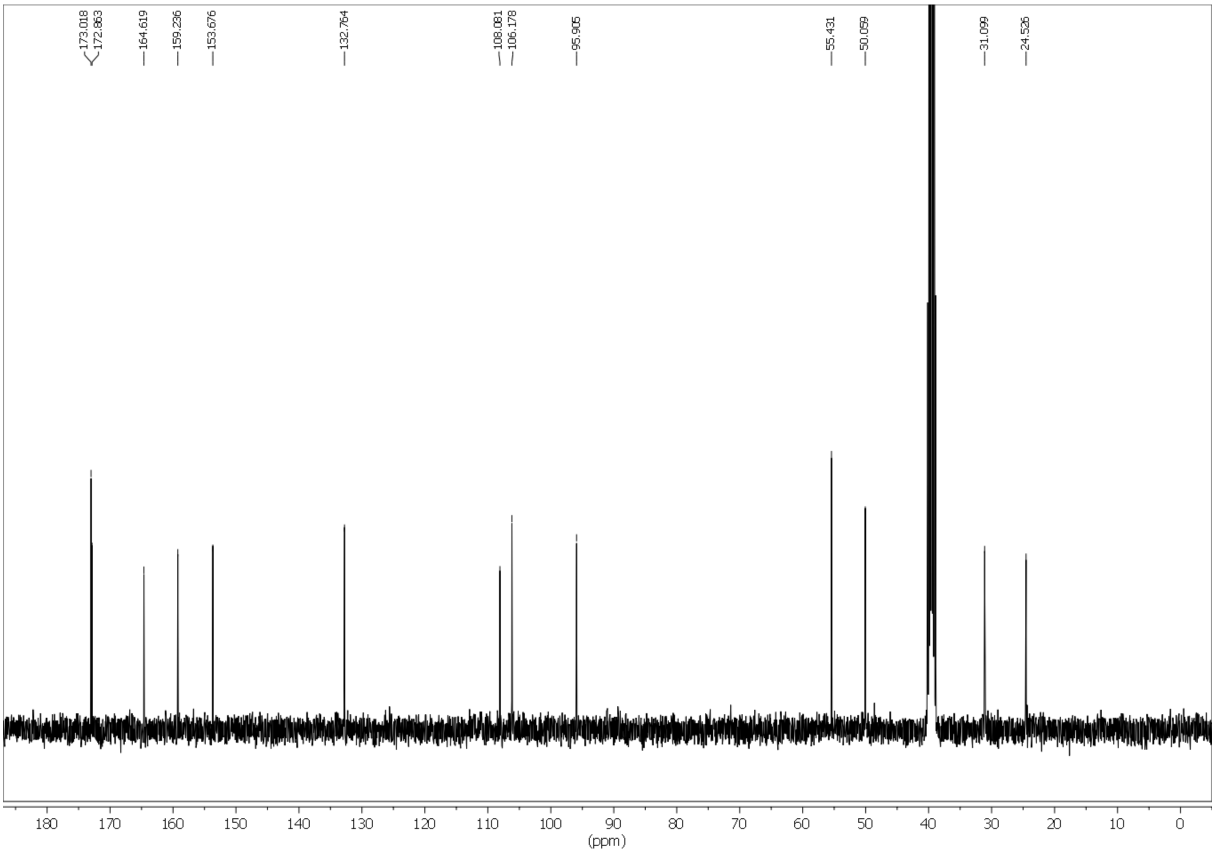

^{1}H and ^{13}C NMR spectrum of compound **11f**

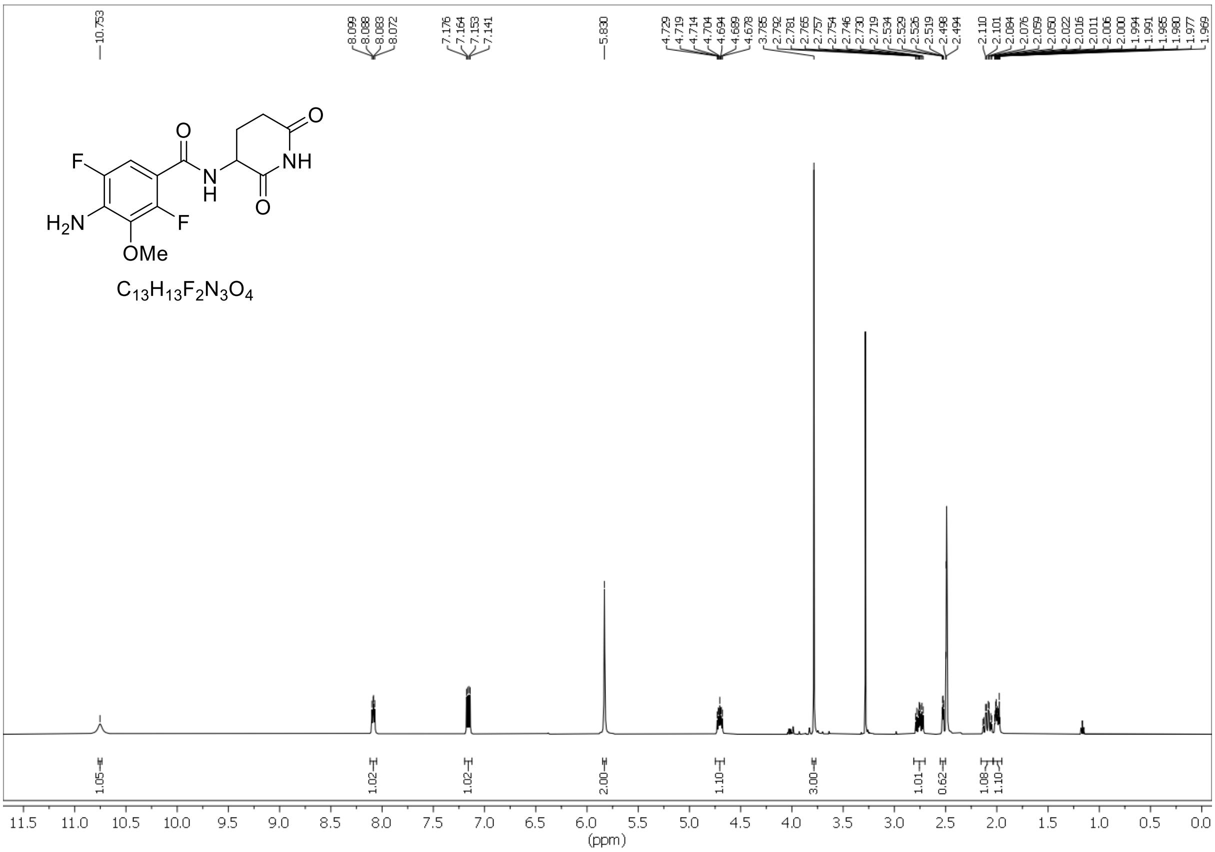

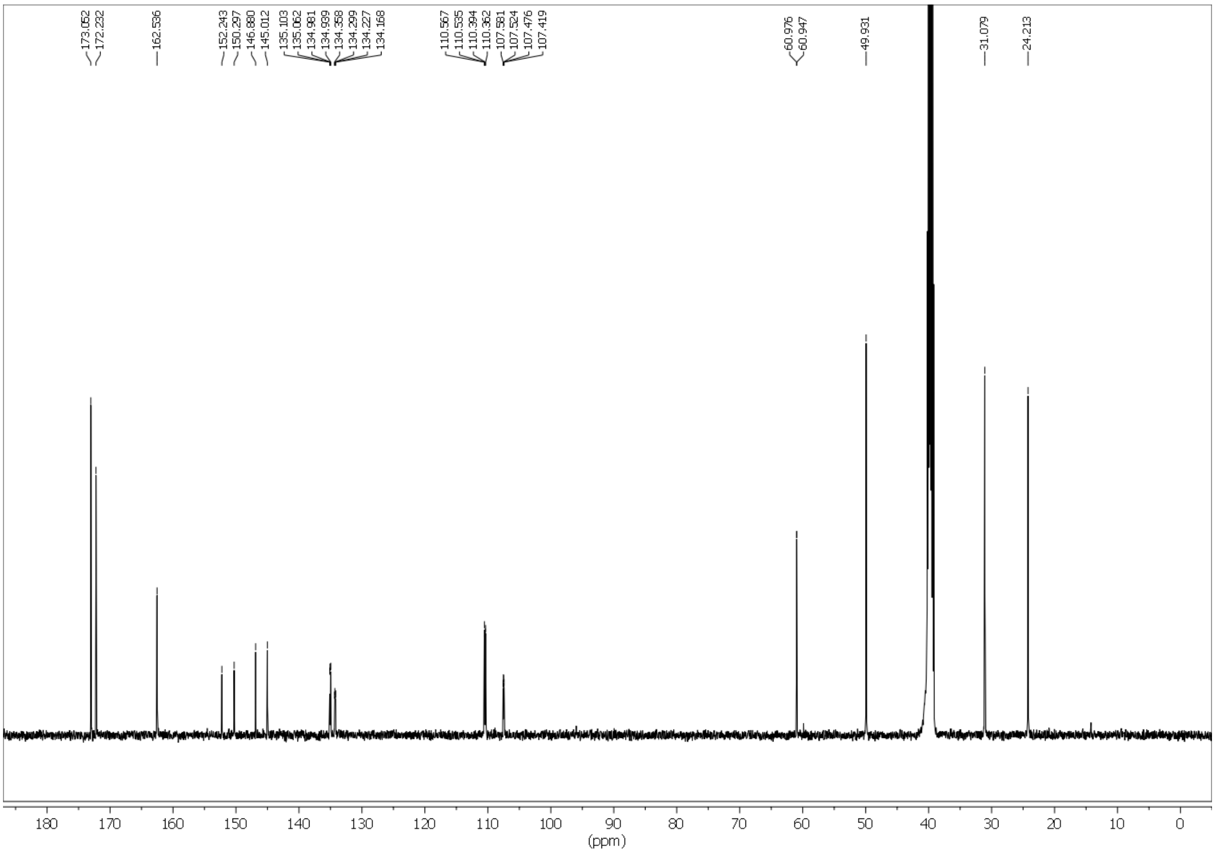

^{1}H and ^{13}C NMR spectrum of compound **40h**

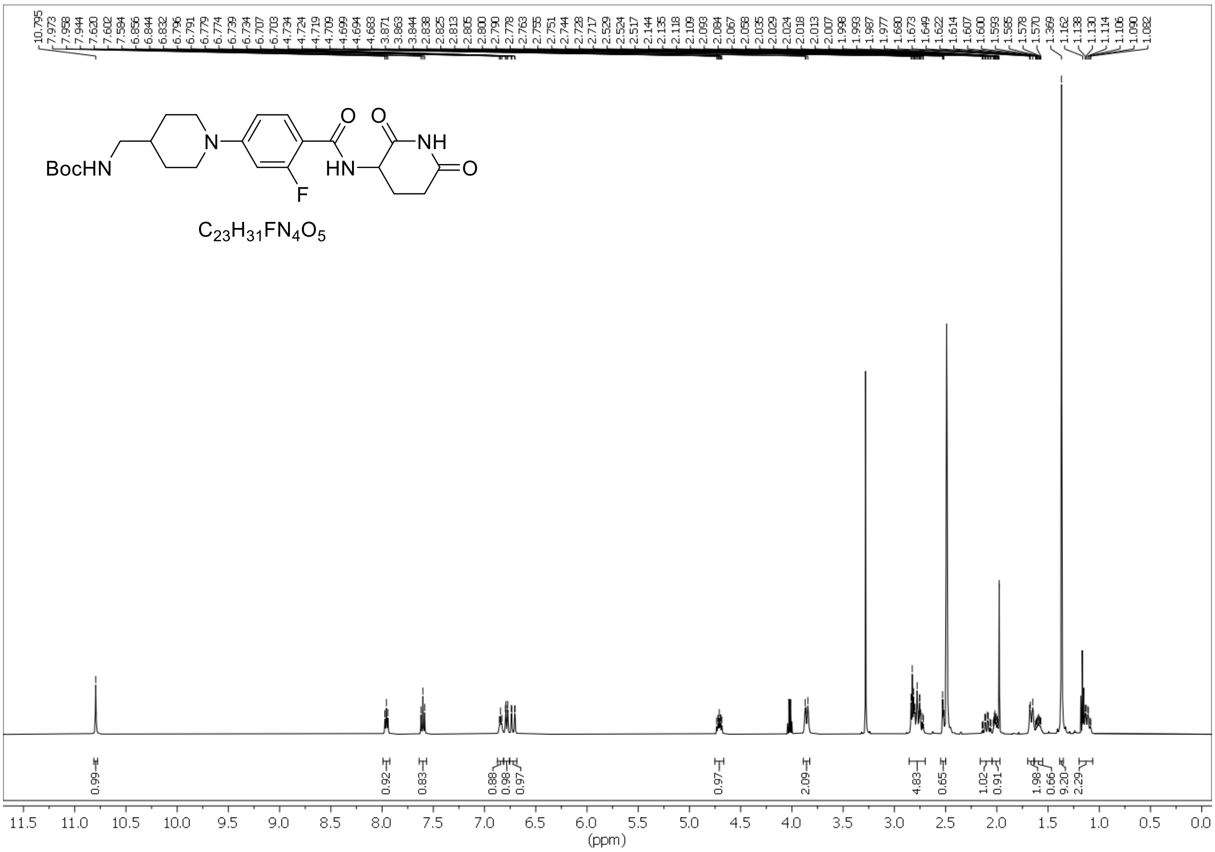

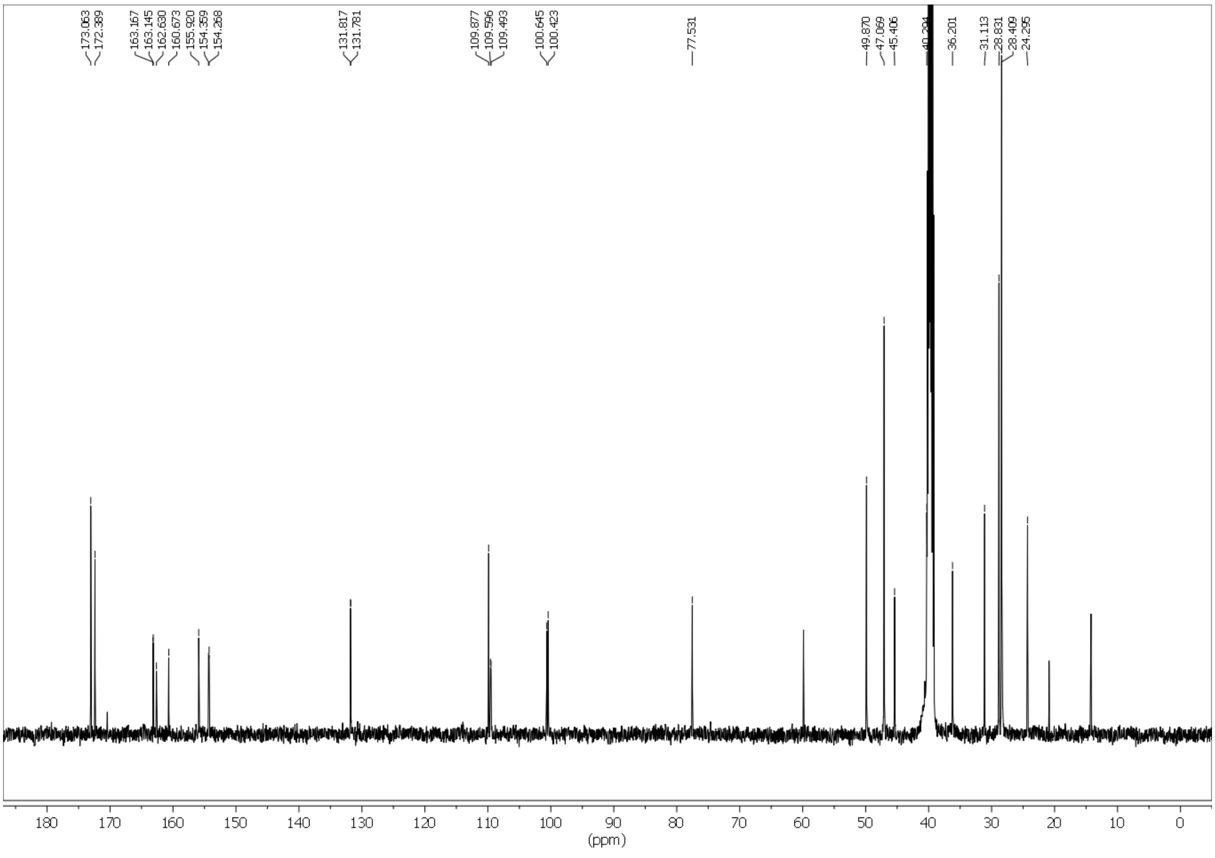

^{1}H and ^{13}C NMR spectrum of compound **41h**

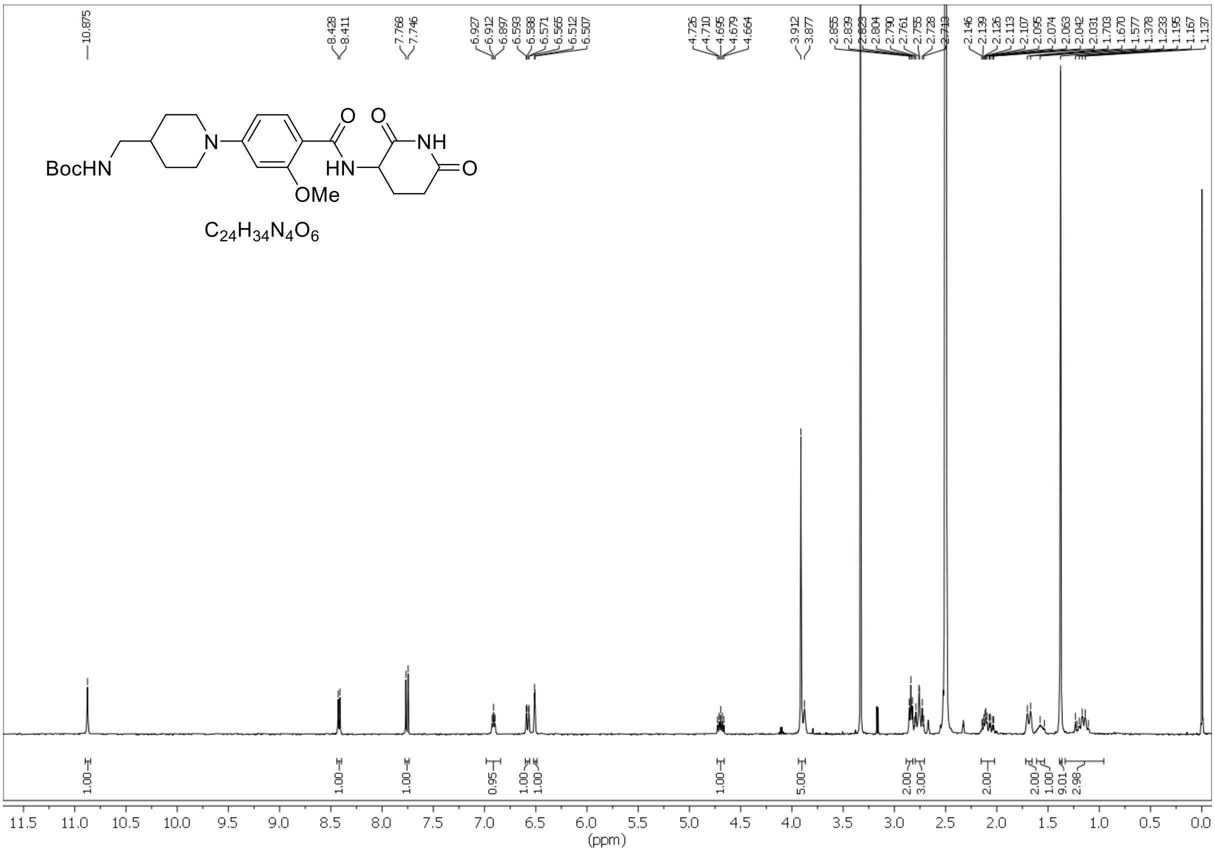

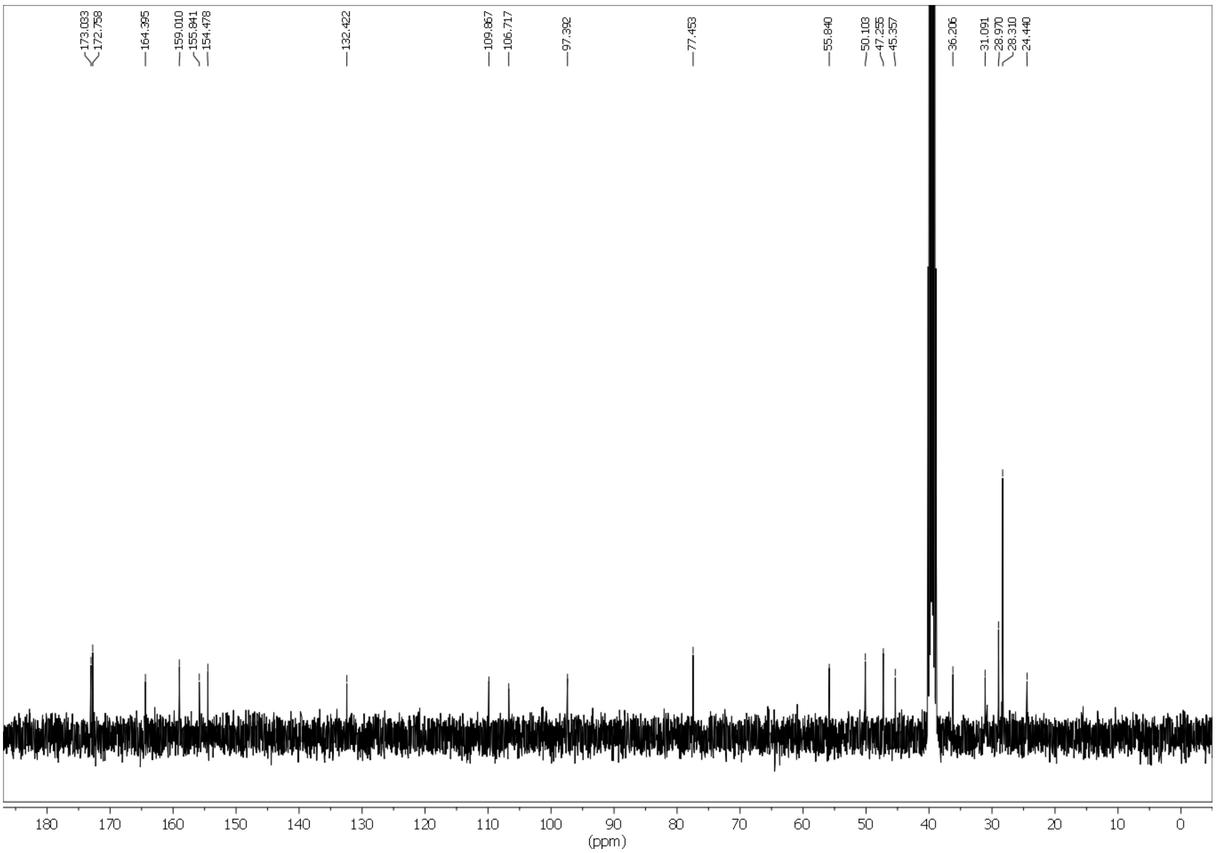

^{1}H and ^{13}C NMR spectrum of compound **43h**

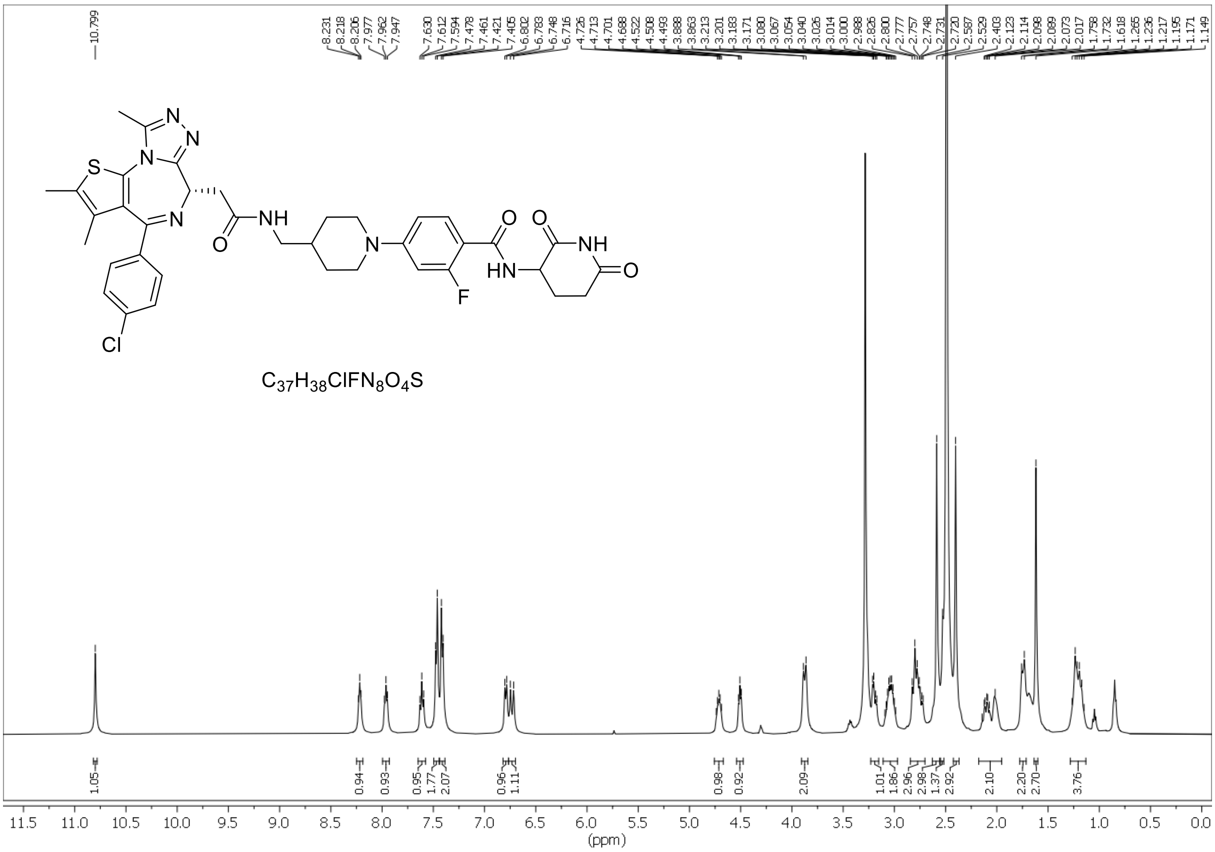

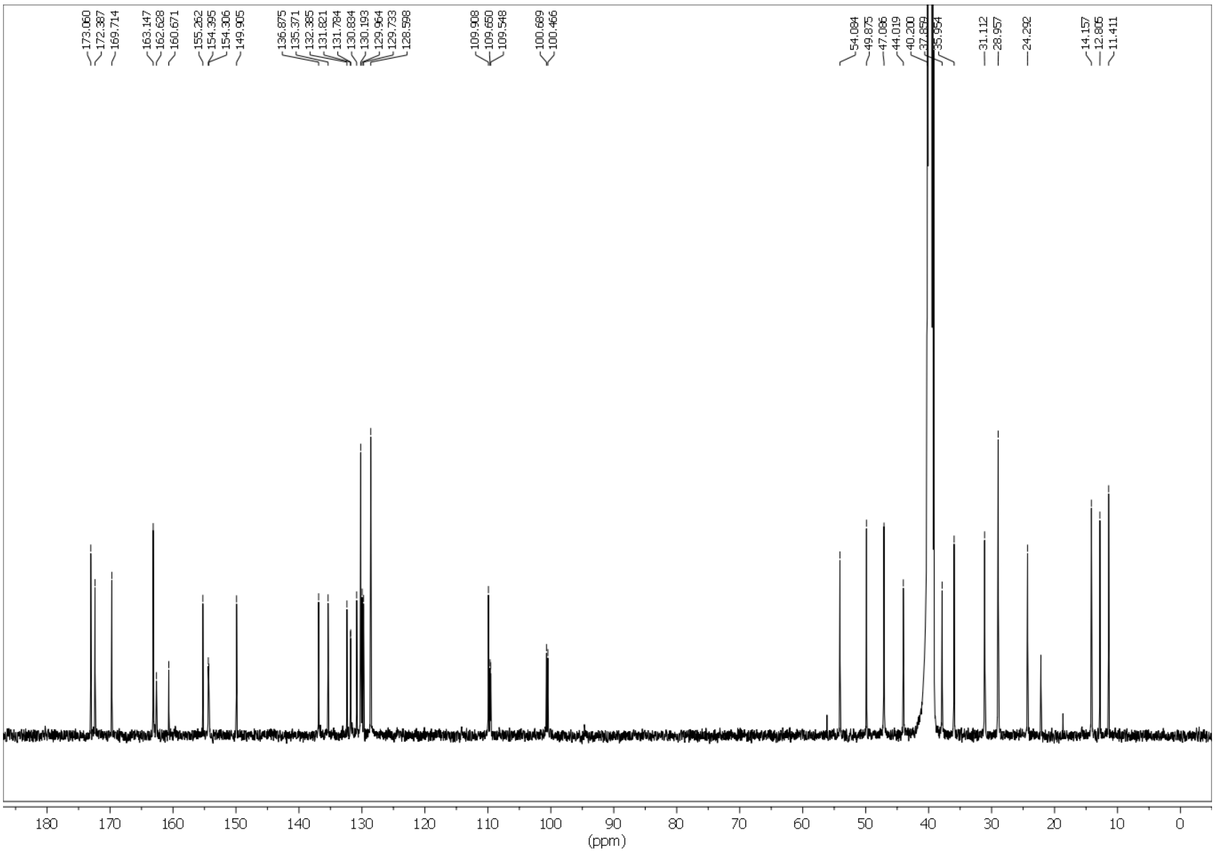

^{1}H and ^{13}C NMR spectrum of compound **44h**

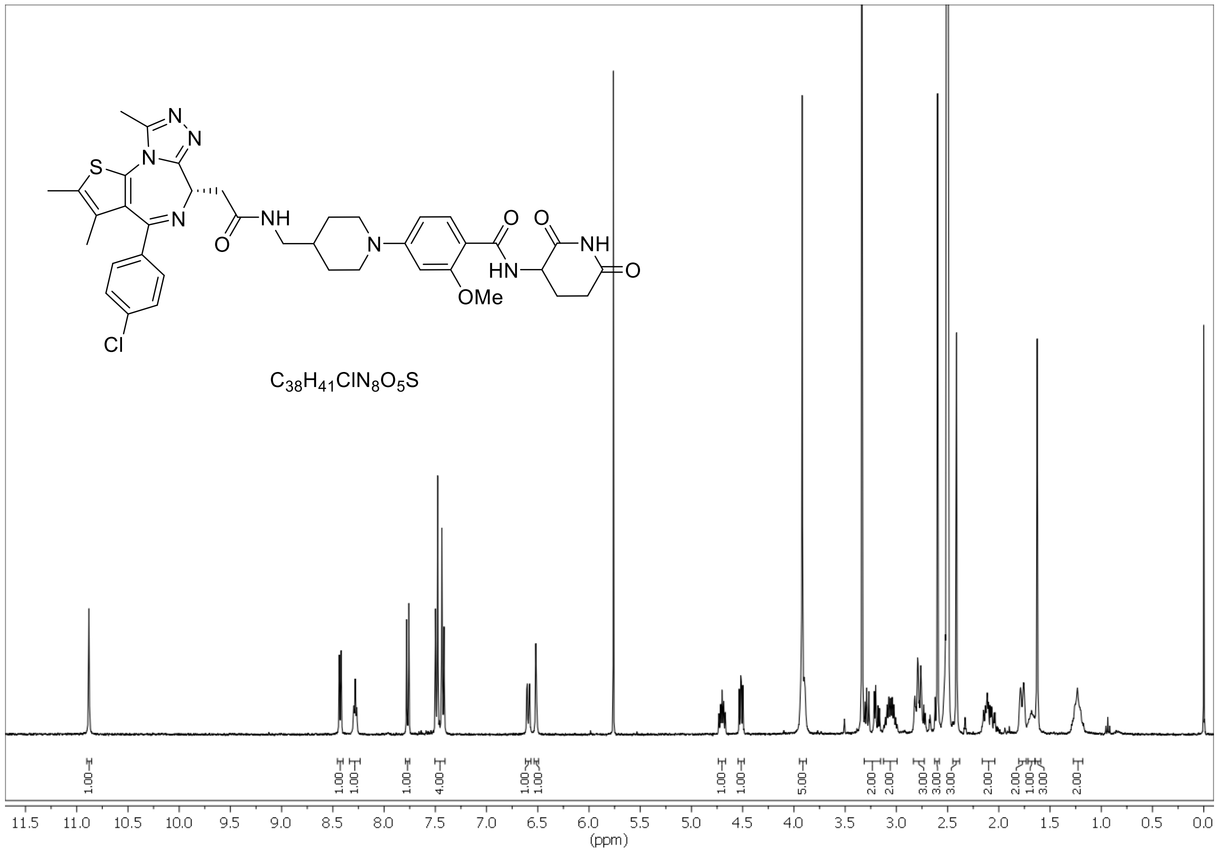

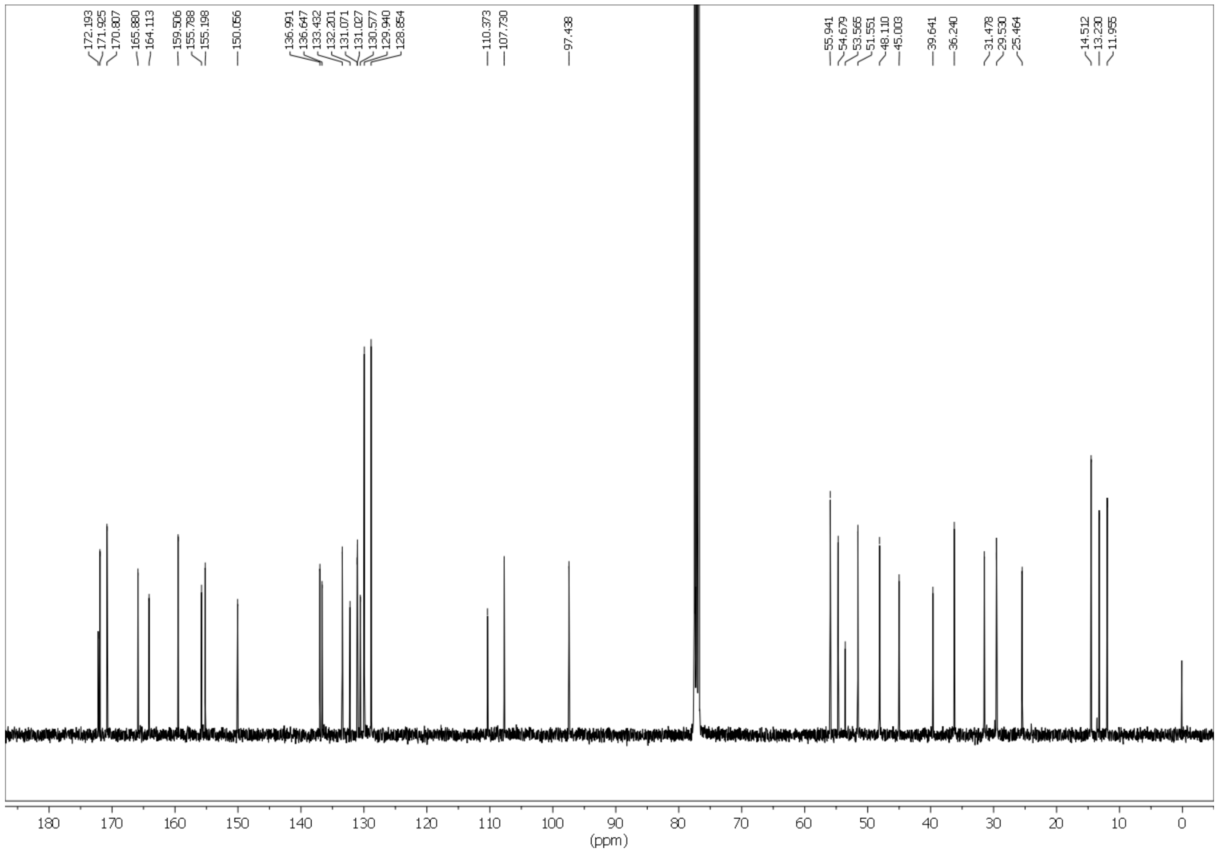

LC/MS traces of compound **43h** (CST991)

**Sequence Name:**	SingleSample		
**Data file:**	CST991_ACN.dx	**Project Name:**	Single Quad
**Sample name:**	CST991_ACN	**Operator :**	SYSTEM
**Instrument:**	Single Quad	**Acquired on:**	2022-01-03 15:44:02+01:00
**Inj. volume:**	2.000	**Location:**	P2-C4
**Acq. method:**	00-new_wasser-acn_standard_100-1500_BD-20min.amx		
**Processing method:**	MS_standard_plot.pmx		

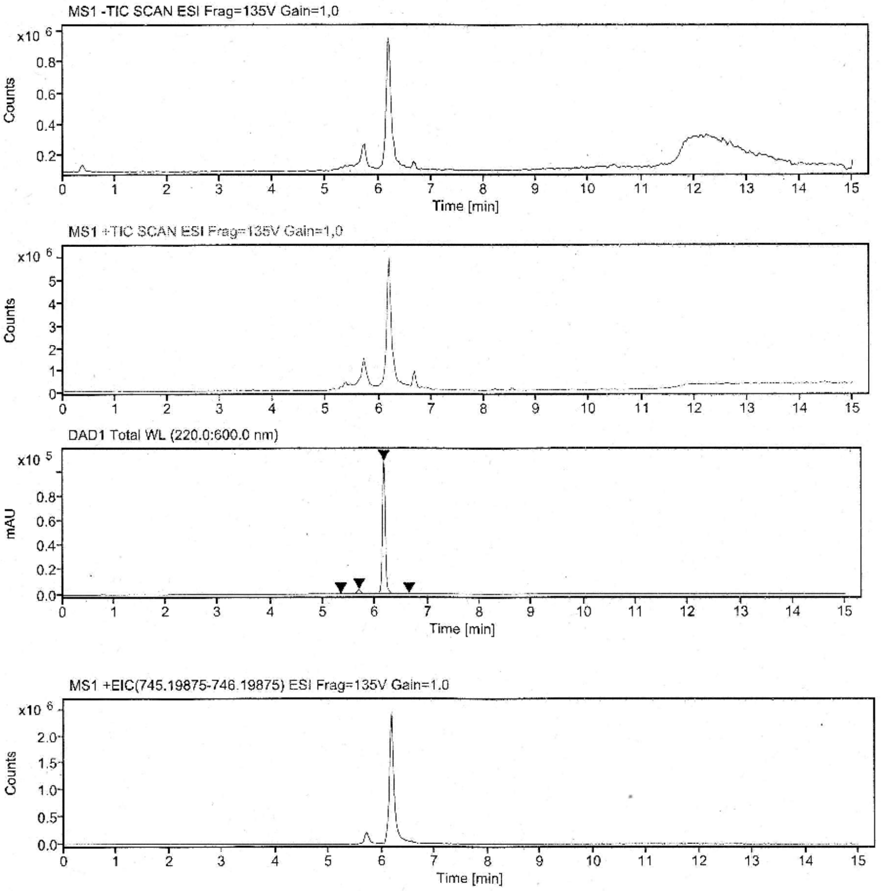

Signal: DAD1 Total WL (220.0:600.0 nm)

RT [min]	Peak MS Base Peak m/z	Area	Area%	Max Peak%	Height
5.331		609.5820	0.1409	0.148	103.957
5.687	743.500	17904.9675	4.1382	4.333	3630.566
6.163	743.600	413248.2074	95.5091	100.000	107974.656
6.641		916.5494	0.2118	0.222	253.116
	Sum	432679.3063			

D:\CDSProjects\Default\0-standardplot+EIC.rdl [Rev. 4464] Printed: 2022-01-04 06:45:36+01:00 Page 1 of 2

Signal: MS1 +TIC SCAN ESI Frag=135V Gain=1,0

RT [min]	Peak MS Base Peak m/z	Area	Area%	Max Peak%	Height
5.718	743.500	6280027.1531	13.8687	16.102	1093226.155
6.195	745.500	39001871.5958	86.1313	100.000	5725015.454
	Sum	45281898.7488			

Signal: MS1 -TIC SCAN ESI Frag=135V Gain=1,0

RT [min]	Peak MS Base Peak m/z	Area	Area%	Max Peak%	Height
5.723	759.400	802148.4247	11.1822	12.590	141676.158
6.194	743.600	6371311.8426	88.8178	100.000	863219.107
	Sum	7173460.2673			

MS1 +TIC SCAN ESI Frag=135V Gain=1,0RT:5.7177241474218min (Subtracted)

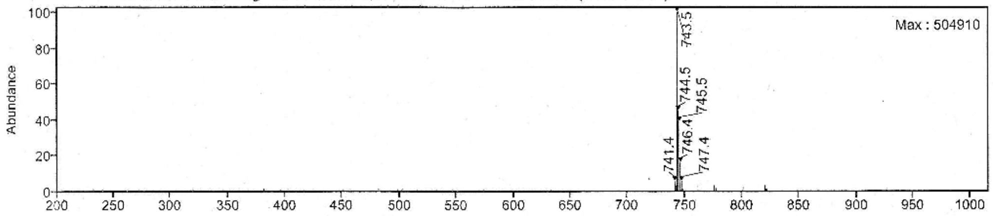

MS1 -TIC SCAN ESI Frag=135V Gain=1,0RT:5.72299352218386min (Subtracted)

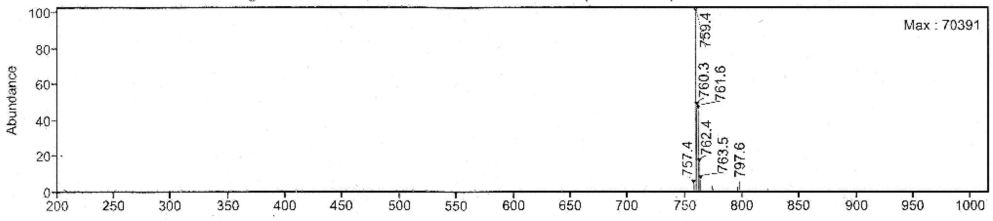

MS1 -TIC SCAN ESI Frag=135V Gain=1,0RT:6.1939283102925min (Subtracted)

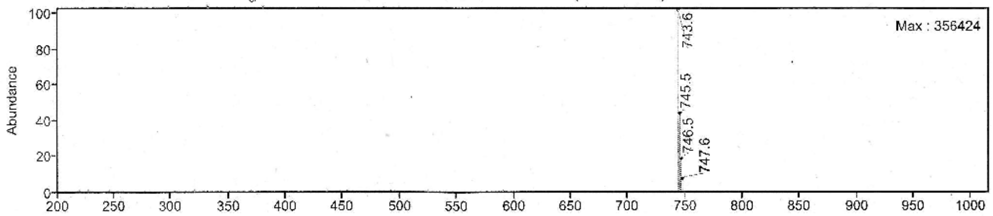

MS1 +TIC SCAN ESI Frag=135V Gain=1,0RT:6.19494772465955min (Subtracted)

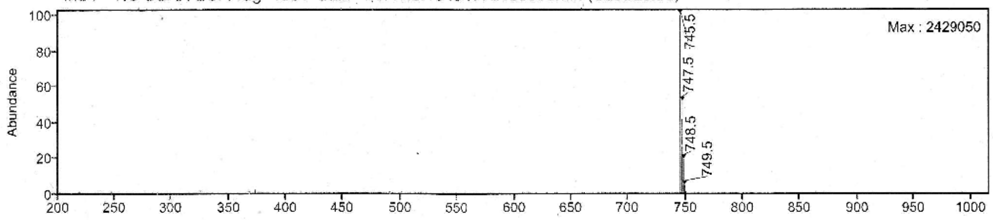

HPLC trace of compound **44h** (SAB650)

Chromatogram and Results

Injection Details

Injection Name:	**SAB 650**	*Run Time (min):*	**14,50**
Vial Number:	**BA3**	*Injection Volume:*	**5,00**
Injection Type:	**Unknown**	*Channel:*	**UV_VIS_3**
Calibration Level:		*Wavelength:*	**254**
Instrument Method:	**Purity_PROTAC**	*Bandwidth:*	**16**
Processing Method:	**Quantitative**	*Dilution Factor:*	**1,0000**
Injection Date/Time:	**30.jan..23 18:54**	*Sample Weight:*	**1,0000**

Chromatogram

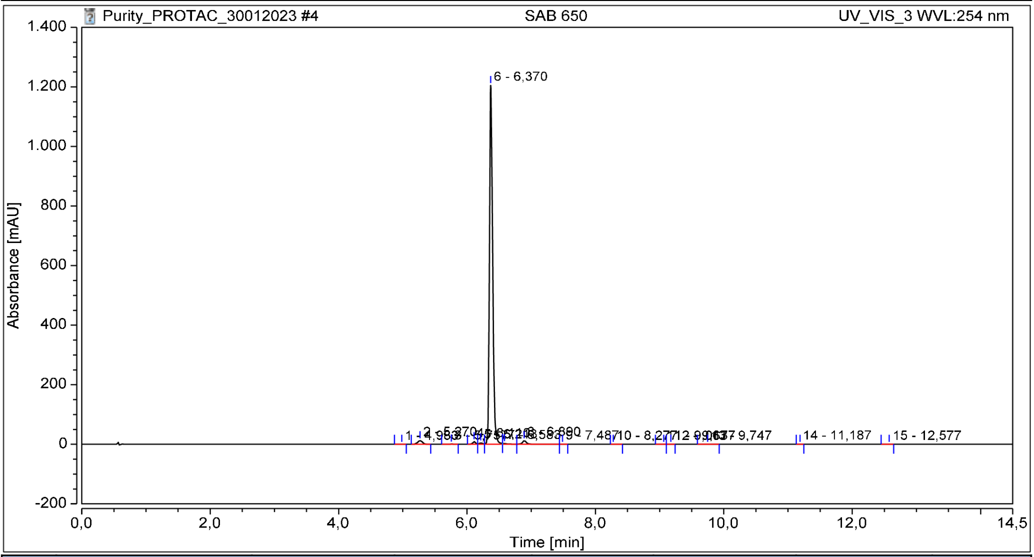

Integration Results

No.	Peak Name	Retention Time min	Area mAU*min	Height mAU	Relative Area %	Relative Height %	Amount n.a.
1		4,983	0,009	0,128	0,01	0,01	n.a.
2		5,270	1,080	12,193	1,49	0,98	n.a.
3		5,757	0,063	0,743	0,09	0,06	n.a.
4		6,110	0,467	8,825	0,64	0,71	n.a.
5		6,213	0,294	4,786	0,41	0,38	n.a.
6		6,370	69,239	1204,739	95,59	96,62	n.a.
7		6,583	0,246	2,431	0,34	0,19	n.a.
8		6,890	0,980	12,182	1,35	0,98	n.a.
9		7,487	0,010	0,168	0,01	0,01	n.a.
10		8,277	0,003	0,061	0,00	0,00	n.a.
11		9,063	0,006	0,065	0,01	0,01	n.a.
12		9,177	0,004	0,071	0,01	0,01	n.a.
13		9,747	0,013	0,105	0,02	0,01	n.a.
14		11,187	0,012	0,294	0,02	0,02	n.a.
15		12,577	0,006	0,103	0,01	0,01	n.a.
**Total:**			**72,433**	**1246,892**	**100,00**	**100,00**	

Chromeleon (c) Dionex
Version 7.2.9.11323

References

(1) Maiwald, S.; Heim, C.; Hernandez Alvarez, B.; Hartmann, M. D. Sweet and Blind Spots in E3 Ligase Ligand Space Revealed by a Thermophoresis-Based Assay. *ACS Med. Chem. Lett.* **2021**, *12* (1), 74–81. https://doi.org/10.1021/acsmedchemlett.0c00440.

(2) Archer, S.; Seyed-Mozaffari, A.; Simon, E. J.; Gioannini, T. L. Opioid Ligands Related to Tifluadom. *Eur. J. Med. Chem.* **1989**, *24* (6), 569–572. https://doi.org/10.1016/0223-5234(89)90022-6.

(3) Cherney, E. C.; Zhang, L.; Lo, J.; Huynh, T.; Wei, D.; Ahuja, V.; Quesnelle, C.; Schieven, G. L.; Futran, A.; Locke, G. A.; Lin, Z.; Monereau, L.; Chaudhry, C.; Blum, J.; Li, S.; Fereshteh, M.; Li-Wang, B.; Gangwar, S.; Pan, C.; Chong, C.; Zhu, X.; Posy, S. L.; Sack, J. S.; Zhang, P.; Ruzanov, M.; Harner, M.; Akhtar, F.; Schroeder, G. M.; Vite, G.; Fink, B. Discovery of Non-Nucleotide Small-Molecule STING Agonists *via* Chemotype Hybridization. *J. Med. Chem.* **2022**, *65* (4), 3518–3538. https://doi.org/10.1021/acs.jmedchem.1c01986.

(4) Mertins, P.; Tang, L. C.; Krug, K.; Clark, D. J.; Gritsenko, M. A.; Chen, L.; Clauser, K. R.; Clauss, T. R.; Shah, P.; Gillette, M. A.; Petyuk, V. A.; Thomas, S. N.; Mani, D. R.; Mundt, F.; Moore, R. J.; Hu, Y.; Zhao, R.; Schnaubelt, M.; Keshishian, H.; Monroe, M. E.; Zhang, Z.; Udeshi, N. D.; Mani, D.; Davies, S. R.; Townsend, R. R.; Chan, D. W.; Smith, R. D.; Zhang, H.; Liu, T.; Carr, S. A. Reproducible Workflow for Multiplexed Deep-Scale Proteome and Phosphoproteome Analysis of Tumor Tissues by Liquid Chromatography–Mass Spectrometry. *Nat. Protoc.* **2018**, *13* (7), 1632–1661. https://doi.org/10.1038/s41596-018-0006-9.

(5) Demichev, V.; Messner, C. B.; Vernardis, S. I.; Lilley, K. S.; Ralser, M. DIA-NN: Neural Networks and Interference Correction Enable Deep Proteome Coverage in High Throughput. *Nat. Methods* **2020**, *17* (1), 41–44. https://doi.org/10.1038/s41592-019-0638-x.
